# Cooperative reactivity of halomethanes and -silanes at an A-frame complex: transannular addition *versus* bridging tetrylenes

**DOI:** 10.1039/d6sc00337k

**Published:** 2026-04-15

**Authors:** Max Passargus, Celine Nieuwland, Merle Arrowsmith, F. Matthias Bickelhaupt, Holger Braunschweig

**Affiliations:** a Institute for Inorganic Chemistry, Julius-Maximilians-Universität Würzburg Am Hubland 97074 Würzburg Germany; b Institute for Sustainable Chemistry & Catalysis with Boron, Julius-Maximilians-Universität Würzburg Am Hubland 97074 Würzburg Germany; c Department of Chemistry and Pharmaceutical Sciences, Amsterdam Institute of Molecular and Life Sciences (AIMMS), Vrije Universiteit Amsterdam De Boelelaan 1108 1081 HZ Amsterdam The Netherlands F.M.Bickelhaupt@vu.nl; d Institute for Molecules and Materials, Radboud University Heyendaalseweg 135 6525 AJ Nijmegen The Netherlands; e Department of Chemical Sciences, University of Johannesburg Auckland Park Johannesburg 2006 South Africa

## Abstract

With CH_2_X_2_ (X = Cl, Br, I) and CCl_4_ the diplatinum(0) complex [(µ-dmpm)_2_Pt_2_(nbe)_2_] (dmpm = bis(dimethylphosphino)methane, nbe = norbornene) undergoes facile cooperative addition of one C–X bond at each Pt centre to yield the methylene-bridged diplatinum(ii) A-frame complexes [(µ-dmpm)_2_Pt_2_X_2_(µ-CY_2_)] (Y=H, Cl). In contrast, reactions with Me_4−*n*_SiX_*n*_ (X = Cl, *n* = 1–3; X = I, *n* = 1) lead preferentially to transannular oxidative additions of a single Si–X bond over the two metal centres, yielding the complexes [(µ-dmpm)_2_{PtX}{Pt(SiMe_4−*n*_X_*n*−1_)}]. In CH_2_Cl_2_ [(µ-dmpm)_2_{PtCl}{Pt(SiCl_3_)}] undergoes rearrangement to the silylene-bridged [(µ-dmpm)_2_Pt_2_Cl_2_(µ-SiCl_2_)], while in CH_2_Br_2_ oxidation of the platinum centres to Pt(ii), Cl–Br exchange, and the insertion of a CH_2_Br_2_-derived methylene unit into the Pt–Si bond are observed. Quantum-chemical calculations provide insights into the differences in reactivity between the halomethanes and -silanes.

## Abstract 2

Dihalomethanes and CCl_4_ directly add across [(µ-dmpm)_2_Pt_2_] (dmdm = (Me_2_P)_2_CH_2_) to generate methylene-bridged A-frame complexes, whereas SiMeCl_3_ and SiCl_4_ first undergo transannular Si–Cl addition, before rearranging to silylene-bridged A-frames.

## Graphical abstract

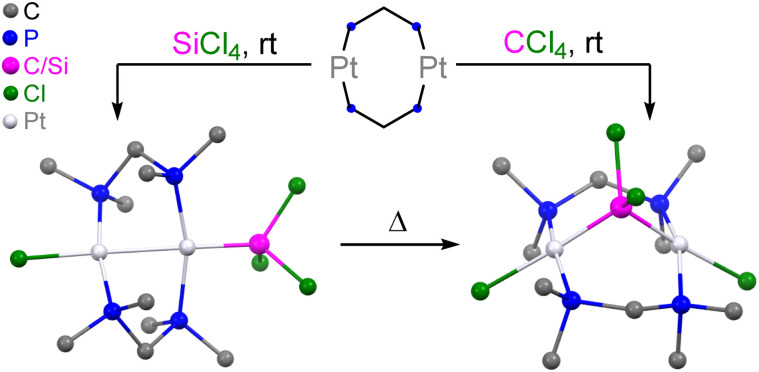

## Introduction

The oxidative addition of polar carbon–halogen bonds to electron-rich metal complexes is a key step in many examples of transition metal (TM) catalysis.^1^ While palladium has been the metal of choice for the vast majority of catalytic cross-coupling processes involving the oxidative addition of organohalides,^2^ more expensive platinum(0) catalysts have found applications in some specialised cross-coupling reactions, including the coupling of polyfluoroarylimines with ZnMe_2_,^3^ thiolates with (hetero)aryl iodides,^4^ hydrazones with allyl halides,^5^ or hydrosilanes with iodoalkanes.^6^

**Fig. 1:**
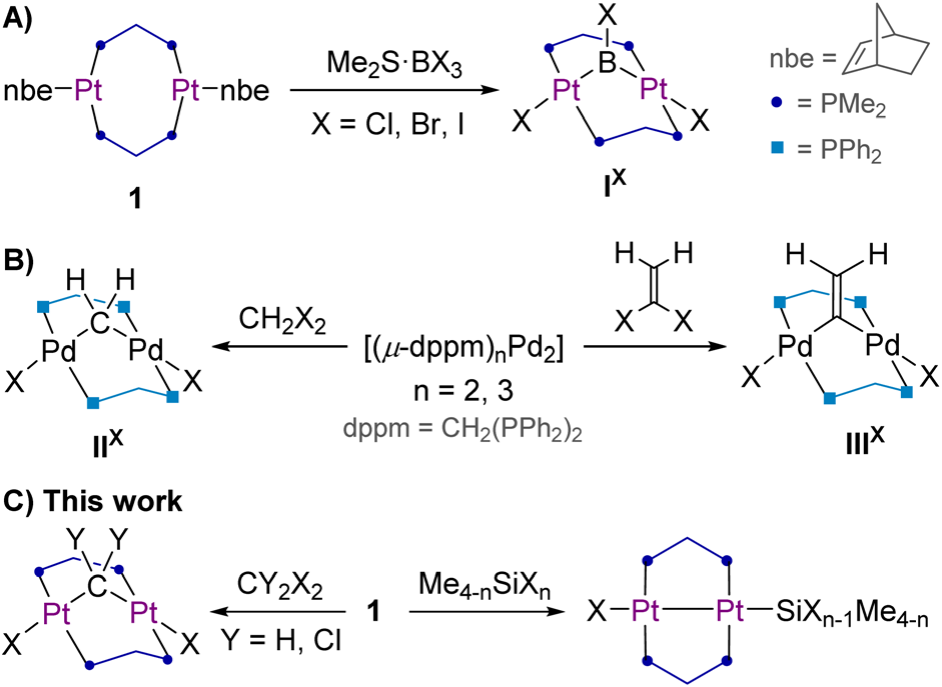
Cooperative addition of (A) trihaloboranes at the diplatinum(0) complex 1, (B) organodihalides at a dipalladium(0) complex, and (C) di- and tetrahalomethanes and heavier group 14 halides at 1.

Whereas the oxidative addition of carbon–halide bonds and their heavier group 14 congeners at mononuclear Pt(0) complexes has been studied in detail,^7,8^ studies of E–X (E = group 14 element, X = halide) bond activations by dinuclear platinum complexes, which may offer unique cooperative reactivity and catalytic enhancement,^9^ remain rare. Our group has studied the oxidative addition of (di)boron halides to the diplatinum(0) complex [(µ-dmpm)_2_Pt_2_(nbe)_2_] (1, dmpm = bis(dimethylphosphino)methane, nbe = norbornene),^10–12^ which offers a flexible platform for the cooperative oxidative addition of B–X bonds at both Pt centres, generating so-called A-frame complexes^13^ with a bridging (di)boranediyl apex ligand, like I^X^ (Fig. 1A). A number of cooperative activations of organodihalides by similar dppm-bridged (dppm = bis(diphenylphosphino)methane) dinickel(0) and dipalladium(0) systems have been reported, affording a range of organodiyl-bridged A-frame complexes, like the methylene- or vinylidene-bridged complexes II^X^ and III^X^.^14^ To our knowledge, however, there have been no reports of cooperative activation of organodihalides, let alone heavier group 14 dihalides, by dinuclear group 10 systems.

In this contribution, we present our investigation into the oxidative addition of di- and tetrahalomethanes at 1 and compare it to that of their heavier group 14 counterparts, underpinning the experimental results by DFT calculations on the origin of the differences in reactivity.

## Results and discussion

### Synthesis of methylene-bridged diplatinum A-frame complexes

**Scheme 1:**
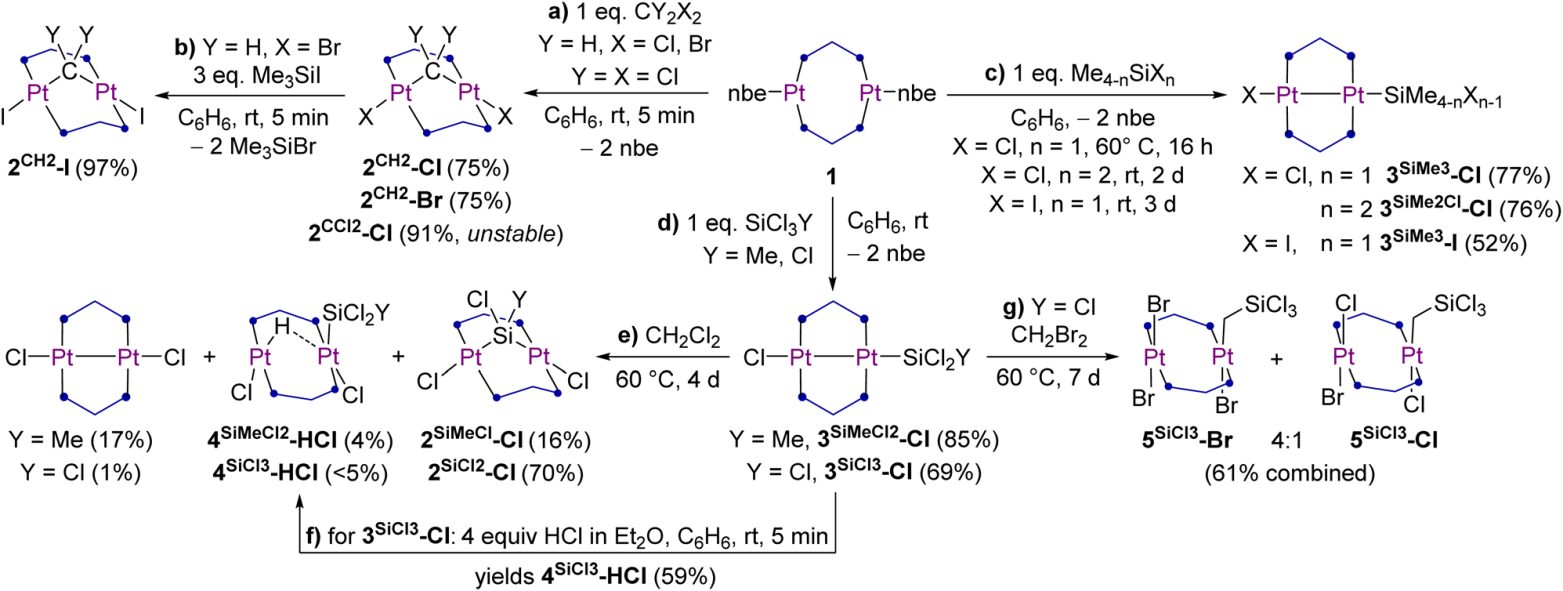
Reactivity of 1 towards halomethanes and halosilanes.

Equimolar reactions of 1 with CH_2_X_2_ (X = Cl, Br) in benzene at room temperature (rt) for 5 min yielded the methylene-bridged platinum(ii) A-frame complexes [(µ-dmpm)_2_Pt_2_X_2_(µ-CH_2_)] (2^CH2^-X) as yellow solids in high isolated yield (75% each, Scheme 1a). To avoid the use of light-sensitive and potentially mutagenic CH_2_I_2_, the brown complex 2^CH2^-I was synthesised quantitatively by treating 2^CH2^-Br with an excess of trimethylsilyl iodide (Scheme 1b). It is noteworthy that all three complexes 2^CH2^-X decompose both in solution and when subjected to vacuum to the diplatinum(i) complexes [(µ-dmpm)_2_Pt_2_X_2_], as has also been observed for boranediyl-bridged diplatinum A-frames under comparable conditions.^10–12^ While we are yet to determine the fate of the lost methylene fragment in these decomposition reactions, its spontaneous release from the A-frame complexes 2^CH2^-X could potentially be harnessed for metal-mediated C–C bond forming reactions in the presence of suitable substrates, such as azoalkanes, alkenes, alkynes and nucleophilic heterocycles.^15^ Coupled with a reductive step to reduce the by-product [(µ-dmpm)_2_Pt_2_X_2_] back to a diplatinum(0) complex, such processes could even be made catalytic.

While the addition of CCl_4_ to 1 also led to the instant formation of the corresponding, orange-coloured dichloromethylene-bridged A-frame complex 2^CCl2^-Cl in near-quantitative yield, the latter was only partially characterised as it decomposed rapidly in solution to a range of unidentified complexes.

The ^1^H NMR spectra of 2^CH2^-X show a quintet (^2^*J*_H–P_ ≈ 9 Hz) with ^195^Pt satellites (^1^*J*_H–Pt_ = 55–58 Hz) for the protons of the bridging methylene, which is shifted upfield with increasing halide electronegativity (*δ* = 0.83 (2^CH2^-Cl), 1.07 (2^CH2^-Br), 1.47 (2^CH2^-I) ppm), as in the series of analogous dipalladium A-frames II^X^.^14^ A similar multiplet (*δ*_1H_ = 1.08 ppm, ^2^*J*_H–P_ ≈ 9 Hz, ^1^*J*_H–Pt_ ≈ 54 Hz) was observed for the analogous [(µ-dppm)_2_Pt_2_Cl_2_(µ-CH_2_)], synthesised in 1985 from the reaction of highly explosive diazomethane with the cationic diplatinum complex [(µ-dppm)_2_{Pt(PPh_3_)}{PtH}]^+^, and subsequent recrystallisation from CH_2_Cl_2_.^16^ The chemical shift of the corresponding high-field ^13^C{^1^H} NMR multiplet shows a similar trend (*δ* = −14.5 (2^CH2^-Cl), −7.6 (2^CH2^-Br), 4.5 (2^CH2^-I) ppm), the ^1^*J*_C–Pt_ coupling remaining relatively unchanged (^1^*J*_C–Pt_ = 613–617 Hz) over the entire series. As expected, the methylene ^13^C NMR resonance of 2^CCl2^-Cl is shifted to much lower field at 20.3 ppm. Conversely, the ^31^P{^1^H} NMR singlet of 2^CH2^-X, with its typical higher-order A-frame satellite splitting pattern (^1^*J*_P–Pt_ ≈ 2980–3070 Hz, ^3^*J*_P–Pt_ ≈ 70 Hz), is shifted upfield upon descending the halide group (from *δ* = −7.3 (2^CH2^-Cl) to −13.2 (2^CH2^-I) ppm), as expected with decreasing halide electronegativity (Table 1). The ^31^P{^1^H} NMR resonance of 2^CCl2^-Cl appears at −8.7 ppm, between those of 2^CH2^-Cl and 2^CH2^-Br, and displays a much lower ^1^*J*_P–Pt_ coupling of 2750 Hz but significantly higher ^3^*J*_P–Pt_ coupling of 150 Hz. An upfield shift is also observed for the ^195^Pt{^1^H} NMR triplet of 2^CH2^-X upon going down the halide group (from *δ* = −4267 ppm (2^CH2^-Cl) to −4645 (2^CH2^-I) ppm), concomitant with a decrease in their ^1^*J*_Pt–P_ coupling constants (from ^1^*J*_Pt–P_ = 3081 (2^CH2^-Cl) to 2981 (2^CH2^-I) Hz). The ^195^Pt{^1^H} NMR triplet of 2^CCl2^-Cl was detected by ^1^H–^195^Pt HMQC at −4270 ppm, close to that of 2^CH2^-Cl. The analysis of the ^195^Pt satellite pattern of the ^31^P{^1^H} NMR multiplets of 2^CH2^-X (see Fig. S2 in the SI)^17^ enables the determination of the ^2^*J*_Pt–Pt_ coupling constants, which decrease from 842 Hz in 2^CH2^-Cl to 754 Hz in 2^CH2^-I, and are similar to that of I^Cl^ (^2^*J*_Pt–Pt_ = 879 Hz).^11^ We have shown that the value of the ^2^*J*_Pt–Pt_ constant in the boranediyl-bridged complexes [(µ-dmpm)_2_Pt_2_X_2_(µ-BY)] (X = halide; Y = halide, amino, organo) decreases as the Pt⋯Pt distance increases, which is also the case in the methylene-bridged A-frame series 2^CH2^-X (*vide infra*).

**Table 1:** ^31^P and ^195^Pt NMR shifts (ppm) and coupling constants (Hz) for the complexes presented herein

	^31^P_P1_	^31^P_P2_	^1^ *J*_P1–Pt1_	^1^ *J*_P2–Pt2_	^*n*^ *J*_P1–Pt2_	^*n*^ *J*_P2–Pt1_	^195^Pt_Pt1_	^195^Pt_Pt2_	^*n*^ *J*_Pt–Pt_
2^CH2^-Cl	−7.3	—	3072	—	73^a^	—	−4267	—	842^b^
2^CH2^-Br	−9.6	—	3036	—	71^a^	—	−4397	—	831^b^
2^CH2^-I	−13.2	—	2982	—	68^a^	—	−4645	—	754^b^
2^Cl2^-Cl	−8.7	—	2750	—	150	—	−4270	—	n.d.^d^
2^SiMeCl^-Cl	−0.1^e^	−2.8^f^	3261	3114	261^a^	203^a^	−4519	—	n.d.
2^SiCl2^-Cl	1.2	—	3098	—	242^a^	—	−4446	—	297^b^
3^SiMe3^-Cl	−15.6	−25.7	3156	2671	122^b^	48^b^	−4317	−4798	3060^c^
3^SiMe3^-I	−21.4	−26.5	3132	2656	116^b^	57^b^	−4686	−4755	n.d.
3^SiMe2Cl^-Cl	−17.5	−26.4	3035	2581	138^b^	52^b^	−4309	−4748	3269^c^
3^SiMeCl2^-Cl	−17.7	−26.0	2930	2475	154^b^	57^b^	−4329	−4752	3738^c^
3^SiCl3^-Cl	−19.4	−26.7	2815	2434	154^b^	62^b^	−4305	−4791	4163^c^
5^SiCl3^-Br	−20.7	−15.0	2420	2702	n.d.	n.d.	−4408	−4421	—
5^SiCl3^-Cl	−16.7	−9.6	2420	2720	n.d.	n.d.	−4367	−4411	—

a *n* = 3.

b *n* = 2.

c *n* = 1.

d n.d. = not determined due to lack of spectral resolution.

e P1/P3.

f P2/P4.

**Fig. 2:**
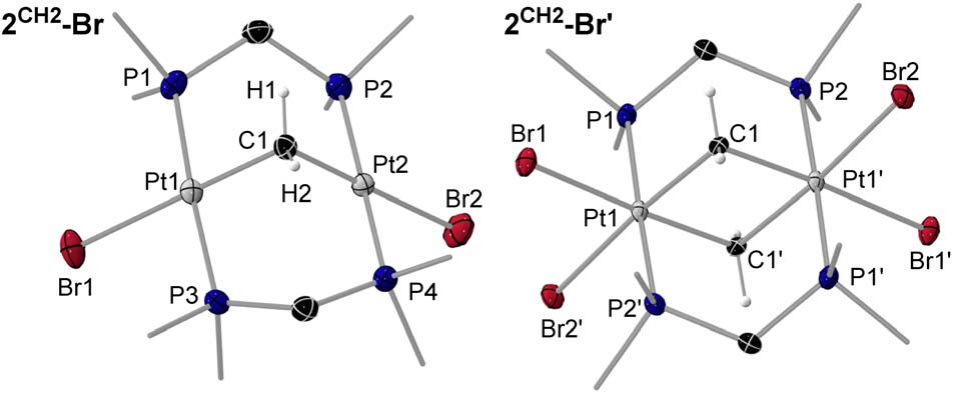
SCXRD-derived solid-state structures of 2^CH2^-Br and 2^CH2^-Br′. Atomic displacement ellipsoids are set at 50%. Ellipsoids of methyl groups and hydrogen atoms are omitted for clarity, except for the protons of the bridging methylene units.

The formation of 2^CH2^-Cl and 2^CH2^-Br was accompanied by the precipitation of a yellow solid, which in the case of the reaction with CH_2_Br_2_ was identified by single-crystal X-ray diffraction (SCXRD) analysis as the bis(methylene)-bridged diplatinum(iv) complex [(µ-dmpm)_2_Pt_2_Br_4_(µ-CH_2_)_2_] (2^CH2^-Br′) (Fig. 2), in which one CH_2_Br_2_ molecule has added on each side of the (µ-dmpm)_2_Pt_2_ core. Unfortunately, the reaction of 1 with a large excess of CH_2_Br_2_ did not yield 2^CH2^-Br′ more selectively. Although further characterisation of these side-products was marred by their insolubility in all common organic solvents, it is noteworthy that 2^CH2^-Br′ is a unique example of a dinuclear group 10 complex bridged by two methylene units.

Crystals of 2^CH2^-Cl, 2^CH2^-Br and 2^CH2^-I were also analysed by SCXRD (Fig. 2 and Table 2, see Fig. S67 and S70 in the SI for 2^CH2^-Cl and 2^CH2^-I). The degree of A-frame distortion in these complexes is quantified by their |P–M–M–P|_*cis*_ and |P–M–M–P|_*trans*_ torsion angles (M = metal centre), which amount to 0° and 180°, respectively, in the ideal A-frame structure. Complexes 2^CH2^-X all show a relatively small degree of A-frame distortion (|P–Pt–Pt–P|_*cis*_ ≤ 19°; |P–Pt–Pt–P|_*trans*_ ≥ 163°), while the centrosymmetric structure of 2^CH2^-Br′ provides a near-ideal A-frame (|P–Pt–Pt–P|_*cis*_ 1.19(5)°; |P–Pt–Pt–P|_*trans*_ 180°). The distortion in 2^CH2^-X is higher than in their haloboranediyl-bridged counterparts I^X^ (|P–Pt–Pt–P|_*cis*_ ≤ 7°; |P–Pt–Pt–P|_*trans*_ ≥ 173°), as the more acute Pt–C–Pt angle (2^CH2^-X avg. 100°; I^X^ Pt–B–Pt 111–126°) enforces a shorter Pt⋯Pt distance (2^CH2^-X avg. 3.15 Å; I^X^ 3.25–3.47 Å).^10^ Moreover, the ^2^*J*_Pt–Pt_ coupling constant decreases (from 842 (2^CH2^-Cl) to 754 (2^CH2^-I) Hz) in line with the increasing Pt⋯Pt distance (from 3.1475(5) (2^CH2^-Cl) to 3.1745(5) (2^CH2^-I) Å). The Pt–C bond lengths in 2^CH2^-X are all similar (avg. 2.06 Å) and consistent with those previously reported for a similar methylene-bridged complex [(µ-dppm)_2_Pt_2_Cl_2_(µ-CH_2_)] (2.008(13) Å).^18^

**Table 2:** Selected bond lengths (Å), bond angles (°) and torsion angles (°) of the complexes characterised by SCXRD

	P–Pt	Pt–X	Pt⋯Pt	Pt1–E	Pt2–E	Pt–E–Pt	|P–Pt–Pt–P|_*cis*_	|P–Pt–Pt–P|_*trans*_
2^CH2^-Cl	2.267(2)–2.294(2)	2.4445(19), 2.4351(19)^a^	3.1475(5)	2.060(7)^d^	2.054(7)^d^	99.8(3)^d^	12.01(7), 18.61(7)	163.02(7), 166.36(7)
2^CH2^-Br	2.2764(17)–2.2864(17)	2.5606(7), 2.5606(7)^b^	3.1604(4)	2.055(6)^d^	2.051(6)^d^	100.7(2)^d^	6.80(6), 13.54(6)	169.69(6), 169.98(6)
2^CH2^-Br′	2.3419(16), 2.3417(16)	2.6099(12), 2.5811(8)^b^	3.1816(10)	2.079(5)^d^	2.077(5)^d^	99.9(2)^d^	1.19(5)	180
2^CH2^-I	2.279(3), 2.288(3)	2.7358(7), 2.7129(6)^c^	3.1745(5)	2.057(9)^d^	2.063(8)^d^	100.8(3)^d^	7.7(2), 9.2(1)	170.4(2), 172.6(1)
2^SiCl2^-Cl	2.2928(16)–2.3054(17)	2.4486(16), 2.4366(16)^a^	3.5682(6)	2.2610(17)^e^	2.2541(17)^e^	104.42(7)^e^	2.28(6), 7.87(6)	161.05(6), 171.20(6)
3^SiMe3^-I	2.2542(19)–2.2714(19)	2.7402(6)^c^	2.7320(5)	—	2.357(2)^e^	—	52.27(7), 54.80(7)	125.15(7), 127.78(7)
3^SiMe2Cl^-Cl	2.2501(19)–2.2796(17)	2.4419(18)^a^	2.6921(7)	—	2.3316(18)^e^	—	53.68(6), 55.83(6)	125.23(6), 125.26(6)
3^SiMeCl2^-Cl	2.2562(19)–2.2798(18)	2.4402(17)^a^	2.6897(6)	—	2.2960(18)^e^	—	52.67(6), 54.89(6)	126.19(7), 126.24(6)
3^SiCl3^-Cl	2.2576(9)–2.2897(8)	2.4195(9)^a^	2.66556(18)	—	2.2933(9)^e^	—	53.14(3), 55.79(3)	125.39(3), 125.67(3)
4^SiCl3^-HCl	2.278(3)–2.330(3)	2.420(3), 2.397(3)^a^	3.1734(9)	—	2.253(3)^e^	—	14.2(1), 28.4(1)	157.5(1), 159.9(1)
5^SiCl3^-Br	2.2959(16)–2.3073(17)	2.4403(7)–2.4968(7)^b^	3.400(1)	2.097(6)^d^	—	—	12.21(7), 12.61(7)	163.88(7), 171.30(7)

a X = Cl.

b X = Br.

c X = I.

d E = C.

e E = Si.

### Transannular oxidative addition of methylhalosilanes

There are no literature precedents for the oxidative addition of heavier group 14 halides at dinuclear platinum complexes. The reaction of 1 with Me_4−*n*_SiCl_*n*_ (*n* = 1–4) provided the transannular oxidative addition products [(µ-dmpm)_2_{PtX}{Pt(SiMe_4−*n*_X_*n*−1_)}] (X = Cl, *n* = 1: 3^SiMe3^-Cl (brown), *n* = 2: 3^SiMe2Cl^-Cl (beige), *n* = 3: 3^SiMeCl2^-Cl (yellow), *n* = 4: 3^SiCl3^-Cl (yellow); X = I, *n* = 1: 3^SiMe3^-I (orange)), in which one Si–X bond has formally added across the central Pt_2_ unit (Scheme 1c and d).††In order to study silicon–halide bond activation, methylhalosilanes were chosen instead of dihydrodihalosilanes (SiH_2_X_2_) as the Si–H bond is significantly weaker than the Si–X bond,^53^ resulting in preferential Si–H activation in test reactions with SiHCl_3_ and Me_2_SiHCl. Except for the reaction with Me_3_SiCl, which required heating at 60 °C for 16 h, all these reactions proceeded at rt. After workup, the resulting silyldiplatinum complexes were isolated in moderate to excellent yields (69–84%). Unlike the A-frame complexes 2^CY2^-X the silyldiplatinum complexes 3^SiMe(3–*n*)X*n*^-X (*n* = 0–3) were stable in solution over longer periods of time. While mononuclear silyl platinum(ii) complexes are key intermediates in the catalytic addition of Si–E (E = H, Si, B) bonds to alkenes and alkynes, as well in the silylation of aryl halides,^19^ the use of bimetallic complexes or of halosilane precursors in such transformations have not yet been has not been studied.

The ^29^Si{^1^H} NMR spectra of 3^SiMe3^-Cl and 3^SiMe3^-I display a triplet of triplets (^2^*J*_Si–P_ ≈ 10 Hz, ^3^*J*_Si–P_ ≈ 2–4 Hz) with ^195^Pt satellites, while 3^SiMeCl2^-Cl and 3^SiCl3^-Cl only show coupling to the adjacent phosphines.‡‡The ^29^Si{^1^H} NMR signal for 3^SiCl3^-Cl was too weak to be detected. As expected, the ^29^Si{^1^H} NMR resonances of the chloro derivatives are progressively deshielded (from *δ* = −11 to 45 ppm) as the number of chlorides increases at the silicon centre. The ^1^*J*_Si–Pt_ and ^2^*J*_Si–Pt_ coupling constants increase with the number of halides at silicon, from 990 and 273 Hz, respectively, in 3^SiMe2Cl^-Cl to 1629 and 624 Hz, respectively, in 3^SiCl3^-Cl. The same trends have been observed for the ^29^Si{^1^H} NMR chemical shifts and ^1^*J*_Si–Pt_ constants in the mononuclear disilylplatinum complexes [(Et_3_P)_2_Pt(SiMe_3−*n*_Cl_*n*_)_2_] (*n* = 0–2). The ^31^P{^1^H} NMR spectra of 3^SiMe(3–^*^n^*^)X^*^n^*-X reflect the asymmetry of the complexes, showing two higher-order multiplets with complex satellite patterns around −26 ppm for the P_2_*Pt*Si nuclei and −15 to −19 ppm for the P_2_*Pt*Cl nuclei, or −21 ppm for the P_2_*Pt*I nucleus. The influence of the *trans*-silyl ligand in the chloro derivatives induces a small but non-negligible upfield shift in the P_2_*Pt*Cl resonance as the degree of chlorination of the silyl ligand increases, as also observed in [(Et_3_P)_2_Pt(SiMe_3−*n*_Cl_*n*_)Cl] (*n* = 0–2).^20^ While the *Pt*Si centres in 3^SiMe(^*^3^*^–^*^n^*^)X^*^n^*-X all display a similar ^195^Pt{^1^H} NMR shift, between −4740 and −4800 ppm, the resonance of the *Pt*I centre in 3^SiMe3^-I (*δ* = −4686 ppm) is significantly upfield-shifted compared to that of the *Pt*Cl centre in the chloro derivatives (*δ* ≈ −4300 to −4330 ppm), as already observed in 2^CH2^-X. In the chloro derivatives the ^1^*J*_Pt–Pt_ coupling constants increase from 3060 Hz to 4163 Hz, both with the degree of chlorination of the silyl ligand and a shortening of the Pt–Pt bond length (*vide infra*). These ^1^*J*_Pt–Pt_ coupling constants are relatively small compared to other unsymmetrical bis(diphosphine)-bridged diplatinum(i) complexes displaying Pt–Pt bonding, such as the stannyl analogue of 3^SiCl3^-Cl, [(µ-dppm)_2_{PtCl}{Pt(SnCl_3_)}] (obtained from the reaction of [(µ-dppm)_2_Pt_2_Cl_2_] with SnCl_2_, ^1^*J*_Pt–Pt_ = 8200 Hz),^21^ [(µ-dppm)_2_Pt_2_Cl_2_] (^1^*J*_Pt–Pt_ = 8146 Hz),^22^ or our [(µ-dmpm^tab^)(µ-dmpm)Pt_2_Br_2_] complexes (dmpm^tab^ = tetrazaborolyl-substituted dmpm ligand, ^1^*J*_Pt–Pt_ = 6670–8510 Hz).^23^

**Fig. 3:**
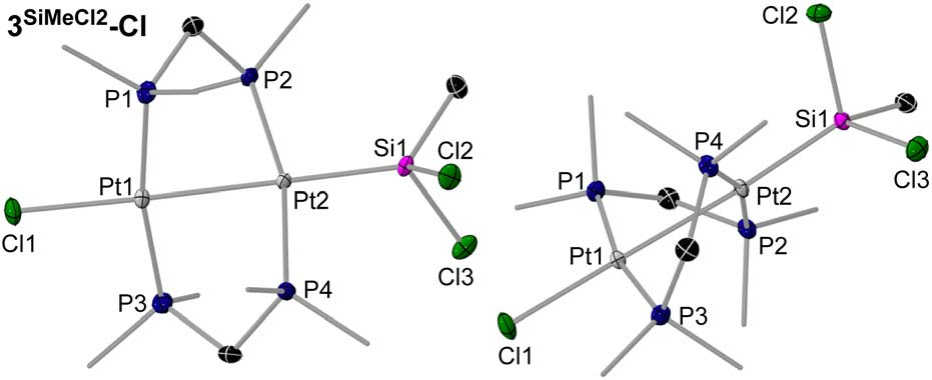
Two views of the SCXRD-derived solid-state structures of 3^SiMeCl2^-Cl, highlighting its twisted framework. Atomic displacement ellipsoids are set at 50%. Ellipsoids of phosphine-bound methyl groups and hydrogen atoms are omitted for clarity.

**Fig. 4:**
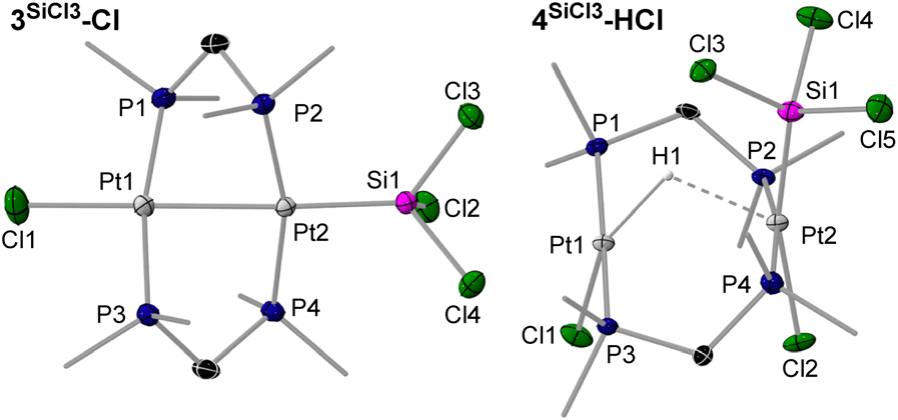
SCXRD-derived solid-state structures of 3^SiCl3^-Cl and 4^SiCl3^-HCl. Atomic displacement ellipsoids are set at 50%. Ellipsoids of methyl groups and hydrogen atoms are omitted for clarity, except for the bridging hydride in 4^SiCl3^-HCl.

Colourless crystals of 3^SiMe2Cl^-Cl, and yellow crystals of 3^SiMe3^-I, 3^SiMeCl2^-Cl and 3^SiCl3^-Cl, provided suitable data for SCXRD analysis (Fig. 3 and 4, left, and Table 2; see Fig. S71 and S72 in the SI for 3^SiMe3^-I and 3^SiMe2Cl^-Cl). As exemplified in the right-hand view of complex 3^SiMeCl2^-Cl in Fig. 3, the (µ-dmpm)_2_Pt_2_ frameworks of these complexes are highly distorted (|P–Pt–Pt–P|_*cis*_ 52–56°, |P–Pt–Pt–P|_*trans*_ 125–128°). Indeed, a CCDC search shows these are the most distorted [(µ-CH_2_{PR_2_}_2_)_2_Pt_2_XY] complexes that have been reported, beside the twisted isomers of our recent [(µ-dmpm^tab^)(µ-dmpm)Pt_2_Br_2_] complexes (|P–Pt–Pt–P|_*cis*_ 49–52°, |P–Pt–Pt–P|_*trans*_ 126–132°),^23^ and [(µ-dmpm)_2_Pt_2_Br_2_] (|P–Pt–Pt–P|_*cis*_ 49°, |P–Pt–Pt–P|_*trans*_ 124–136°).^10^ The Pt–Pt bond lengths range from 2.66556(18) Å in 3^SiCl3^-Cl to 2.7320(5) Å in 3^SiMe3^-I, in the typical range for neutral [{µ-CH_2_(PR_2_)_2_}_2_Pt_2_XY] complexes (2.62–2.71 Å).^10,24^ They also decrease with the degree of chlorination of the silyl ligand, as more electron density is pulled out of the Pt–Pt bond. Similarly, the Pt–Si distance also decreases with the degree of silyl chlorination, from 2.2933(9) in 3^SiCl3^-Cl to 2.357(2) Å in 3^SiMe3^-I. Similar Pt–Si bond lengths are found in mononuclear, unchelated [*trans*-(R_3_P)_2_PtX(SiR′_3_)] complexes (2.32–2.39 Å).^25^

Transannular oxidative additions like these, with concomitant metal–metal bond formation, were first reported by Schmidbaur in 1975 at a cyclometallated dinuclear Au(ii) complex using elemental halogens and methyl iodide.^26^ Other examples of transannular oxidative additions of group 14 halides have been reported for dinuclear Au(i), Au(ii), Ir(i) and Rh(i) complexes,^27–29^ but remain limited to alkyl halides. Similarly, Brown and Puddephatt reported the oxidative addition of alkyl iodides at [(µ-dppm)_3_Pt_2_], which yielded the ionic Pt(i)_2_ complexes [(µ-dppm)_2_{(η^1^-dppm)Pt}{PtR}]I, in which a terminally coordinated dppm ligand takes the place of the iodide.^30^

### Solvent-dependent reactivity of 3^SiMeCl2^-Cl and 3^SiCl3^-Cl

During the full NMR-spectroscopic characterisation of both 3^SiMeCl2^-Cl and 3^SiCl3^-Cl in CD_2_Cl_2_, the appearance of two new products was observed after several hours at rt (Scheme 1e). The minor one was identified by SCXRD as 4^SiMeCl2^-HCl and 4^SiCl3^-HCl, respectively, the product of the transannular addition of HCl, possibly derived from traces of HCl present from the degradation of the dichloromethane solvent through either hydrolysis, photolysis or Pt-catalysed oxidation with trace oxygen.^31^ The major one was identified as the rearrangement product 2^SiMeCl^-Cl and 2^SiCl2^-Cl, respectively, *i.e.* the corresponding silylene-bridged A-frame complex. Complexes 4^SiMeCl2^-HCl and 4^SiCl3^-HCl were systematically detected in small amounts (<5%) in dichloromethane solutions of 3^SiMeCl2^-Cl and 3^SiCl3^-Cl, respectively. For full characterisation 4^SiCl3^-HCl was synthesised independently by the addition of 1 equiv. HCl in Et_2_O to 3^SiCl3^-Cl (Scheme 1f). In the ^31^P{^1^H} NMR spectra, 4^SiMeCl2^-HCl and 4^SiCl3^-HCl appear as two multiplets around −7 to −9 ppm (*P*_2_PtHCl) and −14 ppm (*P*_2_PtSiCl), and in the ^195^Pt{^1^H} NMR spectra as two multiplets around −4600 and −4700 ppm, as also confirmed by ^1^H–^195^Pt{^1^H} HSQC experiments. The ^1^H NMR spectrum of 4^SiCl3^-HCl shows a highly shielded Pt*H* triplet at −15.8 ppm (^2^*J*_H–P_ = 13.2 Hz), with satellites to the two Pt nuclei (^1^*J*_H–Pt_ = 1193 Hz, ^3^*J*_H–Pt_ = 22.8 Hz). These values are similar to those of the mononuclear [*trans*-(Me_3_P)_2_PtHCl], which shows a Pt*H* triplet at −16.2 ppm (^2^*J*_H–P_ = 17 Hz), with a slightly larger coupling to the adjacent Pt nucleus (^1^*J*_H–Pt_ = 1308 Hz).^32^

The solid-state structure of 4^SiCl3^-HCl confirms the transannular oxidative addition of HCl at 3^SiCl3^-Cl (Fig. 4, right). The Pt⋯Pt distance of 3.1734(9) Å is similar to that of the A-frame complexes 2^CH2^-X, thus precluding any Pt–Pt bonding. As a result, the (µ-dmpm)_2_Pt_2_ framework is much less distorted than in 3^SiCl3^-Cl (|P–Pt–Pt–P|_*cis*_ ≤ 28.4(1)°, |P–Pt–Pt–P|_*trans*_ ≥ 157.5(1)°). The bridging hydride, located in the inverse Fourier map and freely refined, bridges very unsymmetrically between the Pt centres (Pt1–H1 1.87(11), Pt2⋯H1 2.23(11) Å). To our knowledge, 4^SiCl3^-HCl is the first example of a neutral hydridodiplatinum(ii) complex, the only reported cationic one being the symmetrical complex [(µ-dmpm)_2_Pt_2_Me_2_(µ-H)]^+^, with a significantly shorter Pt⋯Pt distance 2.932(1) Å, the hydride not having been located in that structure.^33^

Whereas 3^SiCl3^-Cl fully rearranged to the µ-silylene A-frame complex 2^SiCl2^-Cl over 4 days at 60 °C in CH_2_Cl_2_, enabling its isolation in 70% yield, the rearrangement of 3^SiMeCl2^-Cl to 2^SiMeCl^-Cl remained incomplete, reaching only 17%, and was accompanied by the formation of the decomposition product [(µ-dmpm)_2_Pt_2_Cl_2_] (Scheme 1e). Like its methylene-bridged analogues, 2^SiCl2^-Cl proved unstable in solution, decomposing spontaneously to [(µ-dmpm)_2_Pt_2_Cl_2_] with release of the:SiCl_2_ unit, the exact fate of which was not ascertained. As such, 2^SiCl2^-Cl may provide an interesting starting point for studying platinum-mediated or -catalysed silylene transfer reactions, which are usually performed using silirane precursors.^34^

**Fig. 5:**
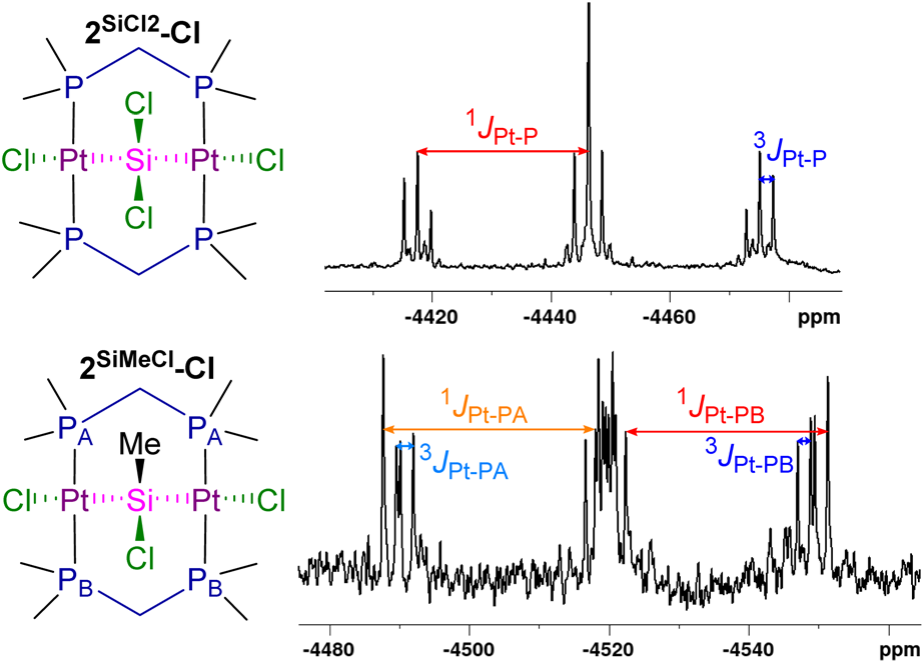
^195^Pt{^1^H} spectra of 2^SiCl2^-Cl and 2^SiMeCl^-Cl.

Complex 2^SiCl2^-Cl shows a ^29^Si NMR triplet with Pt satellites at 22.5 ppm (^2^*J*_Si–P_ = 10.2 Hz, ^1^*J*_Si–Pt_ = 1559 Hz), *ca.* 20 ppm upfield-shifted compared to the terminal silyl resonance of 3^SiMeCl2^-Cl (*δ* = 44.6 ppm). The ^31^P{^1^H} NMR singlet of 2^SiCl2^-Cl at 1.2 ppm is *ca.* 10 ppm upfield-shifted from that of its dichloromethylene-bridged analogue 2^CCl2^-Cl (*δ* = −8.7 ppm), as the silylene is more electron-donating than the methylene ligand. Both the ^1^*J*_P–Pt_ (3098 (2^SiCl2^-Cl), 2750 (2^CCl2^-Cl) Hz) and ^3^*J*_P–Pt_ (242 (2^SiCl2^-Cl), 150 (2^CCl2^-Cl) Hz) coupling constants increase significantly upon changing from the CCl_2_ to the SiCl_2_ bridge. Interestingly, complex 2^SiMeCl^-Cl displays two 1 : 1 ^31^P{^1^H} NMR triplets at −0.1 and −2.8 ppm, rather than a singlet. This is owed to the asymmetry generated by the SiMeCl bridge, as the methyl and chloride substituents each point to one of the dmpm ligands, thereby creating slightly different magnetic environments. The ^195^Pt NMR triplet of 2^SiCl2^-Cl at −4446 ppm is also upfield-shifted from 2^CCl2^-Cl (*δ* = −4270 ppm), while its ^3^*J*_Pt–Pt_ coupling constant is substantially lower than that of all the methylene-bridged A-frames (^3^*J*_Pt–Pt_ = 297 (2^SiCl2^-Cl), 750–842 (2^SiCH2^-X) Hz),§§The ^2^*J*_Pt–Pt_ coupling constant of 2^CCl2^-Cl could not be determined due to poor spectral resolution. in line with the larger Pt⋯Pt distance in 2^SiCl2^-Cl (*vide infra*). For 2^SiMeCl^-Cl the asymmetry generated by the SiMeCl bridge results in a dddd resonance at −4519 ppm in the ^195^Pt{^1^H} NMR spectrum, with two distinct ^1^*J*_Pt–P_ (3261, 3114 Hz) and ^3^*J*_Pt–P_ (261, 203 Hz) coupling constants to each of the dmpm ligands (Fig. 5).

**Fig. 6:**
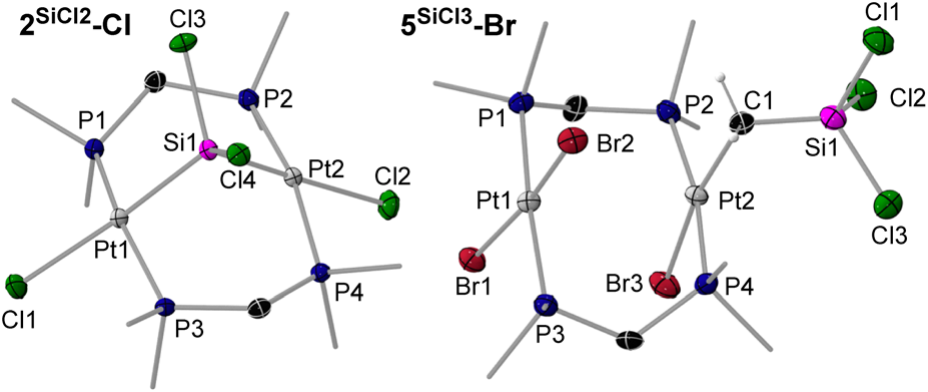
SCXRD-derived solid-state structures of 2^SiCl2^-Cl and 5^SiCl3^-Br. Atomic displacement ellipsoids are set at 50%. Ellipsoids of methyl groups and hydrogen atoms are omitted for clarity, except for the methylene protons in 5^SiCl3^-Br.

Green crystals of 2^SiCl2^-Cl were analysed by SCXRD (Fig. 6, left). The much larger covalent radius of the silicon compared to the carbon bridge (*r*_Si_ = 1.11 Å, *r*_C_ = 0.76 Å) induces a significant widening of the Pt⋯Pt distance (3.5682(6) Å) compared to 2^CH2^-Cl (3.1475(5) Å), which in turn releases the strain of the (µ-dmpm)_2_Pt_2_ framework, resulting in a nearly ideal A-frame structure (|P–Pt–Pt–P|_*cis*_ ≤ 7.87(6), |P–Pt–Pt–P|_*trans*_ ≥ 161.05(6)°). The Pt–Si bonds in 2^SiCl2^-Cl (2.2610(17), 2.2541(17) Å) are significantly shorter than in the silyl complexes 3^SiMe(3–^*^n^*^)X^*^n^*-X (2.2933(9)–2.357(2) Å), or other silylene-bridged diplatinum complexes with square-planar Pt coordination environments (2.2998(11)–2.412(2) Å).^35,36^ This shortening is likely due to the strain imposed by the A-frame and the electron-withdrawing effect of the chlorides *trans* to the silylene.

While there are numerous examples of silylene-bridged di- and triplatinum(0) complexes, usually synthesised by dehydrogenation of dihydrosilanes or the direct addition of stable silylenes to the Pt(0) precursors,^37^ our silylene-bridged A-frames are the first examples of µ-silylene diplatinum(ii) complexes, obtained by twofold Si–X oxidative addition at two Pt(0) centres. There are, however, a couple of examples of homo- and heterobimetallic µ-silylene platinum(ii) complexes obtained by the oxidative addition of the Si–H bond of a hydrosilyl ligand to a Pt centre.^36^ In these cases, the bridging SiR_2_ ligand should rather be viewed as a triplet silanediyl ligand with electron-sharing Pt–Si bonds, rather than a singlet silylene with Si→Pt donor–acceptor bonding. The same observation has been made for our BY-bridged A-frame complexes, which should be regarded as boranediyl-rather than borylene-bridged complexes.

While in CH_2_Cl_2_ complex 3^SiCl3^-Cl rearranges to 2^SiCl2^-Cl, in CH_2_Br_2_ the unsymmetrical silyl(trihalo)diplatinum(ii) complexes 5^SiCl3^-Br and 5^SiCl3^-Cl are formed, in which a unique twofold oxidative addition of CH_2_Br_2_, concomitant with methylene insertion into the Pt–Si bond, and in the case of 5^SiCl3^-Br an additional Cl–Br exchange, have taken place. The two products were formed in a 4 : 1 mixture, as determined by NMR spectroscopy, and could not be separated by fractional crystallisation (Scheme 1g). Both species were detected by HRMS, and 5^SiCl3^-Br was additionally characterised by SCXRD (*vide infra*). In the ^1^H NMR spectrum complexes 5^SiCl3^-Br and 5^SiCl3^-Cl show a distinctive P_2_PtC*H*_2_ 2H triplet at 1.74 and 2.35 ppm (^2^*J*_H–P_ = 8.5 Hz), respectively, corresponding to a shielded ^13^C{^1^H} NMR Pt*C*H_2_ multiplet at −1.2 ppm. In the ^29^Si{^1^H} NMR spectrum both compounds overlap at 2.5 ppm. The ^31^P{^1^H} NMR spectrum shows two triplets for each complex, reflecting their asymmetry, one set at −15.0 (*P*_2_PtBr(CH_2_SiCl_3_)) and −20.7 (*P*_2_PtBr_2_) ppm for 5^SiCl3^-Br (^2^*J*_P–P_ = 11.8 Hz), and one at −9.6 (*P*_2_PtCl(CH_2_SiCl_3_)) and −16.7 (*P*_2_PtBrCl) ppm for 5^SiCl3^-Cl (^2^*J*_P–P_ = 15.2 Hz), with ^1^*J*_P–Pt_ coupling constants of *ca.* 2400 Hz for the *P*_2_PtX_2_ moiety and *ca.* 2700 Hz for the *P*_2_PtX(CH_2_SiCl_3_) moiety. These assignments are based on comparison with the ^31^P NMR data of the mononuclear complexes *trans*-[(Me_3_P)_2_PtX_2_] (X = Cl, *δ* = −15.8 ppm, ^1^*J*_P–Pt_ = 2386 Hz; X = Br, *δ* = −21.5 ppm, ^1^*J*_P–Pt_ = 2324 Hz),^38^ and *trans*-[(Me_3_P)_2_Pt(CH_2_SiMe_3_)Cl] (*δ* = −14.6 ppm, ^1^*J*_P–Pt_ = 2765 Hz).^39^ The orientation of the Cl and Br ligands at the first Pt centre in 5^SiCl3^-Cl relative to the CH_2_SiCl_3_ ligand of the second Pt centre remains unclear, as no single crystals of 5^SiCl3^-Cl could be obtained.

The solid-state structure of 5^SiCl3^-Br (Fig. 6, right) shows two near-parallel square-planar Pt centres (Pt1 ∠ Pt2 *ca.* 3°) bridged by two dmpm ligands, one bearing two bromides and the other a bromide and a CH_2_SiCl_3_ ligand. The Pt⋯Pt distance of 3.400(1) Å is significantly shorter than in the related complex *trans*-[(µ-dmpm)_2_Pt_2_I_4_] (Pt⋯Pt 3.3477(6) Å).^11^ The Pt2–C1 bond length of 2.091(6) Å is similar to that of the related *trans*-[(Me_2_PhP)_2_PtCl(CH_2_SiMe_3_)] (2.079(14) Å).^40^

Insertion reactions of unsaturated hydrocarbons into Pt–Si bonds represent a key step in the Pt-catalysed hydrosilylation of alkenes and alkynes.^41^ To our knowledge, however, there have been no reports of methylene insertions into a Pt–Si bond. Puddephatt and coworkers have studied a similar insertion of methylene into the Pt–aryl bond of a cycloneophylplatinum(ii) complex upon addition of CH_2_X_2_ (X = Cl, Br, I), which was calculated to proceed *via* a radical mechanism.^42^ Others have reported ionic reaction mechanisms of chloromethyl platinum(ii) complexes involving the displacement of the chloride anion by various Lewis bases (LB) and formation of new Pt–CH_2_–LB linkages (E = PR_3_, SR_2_, NR_n_).^43^

**Scheme 2:**
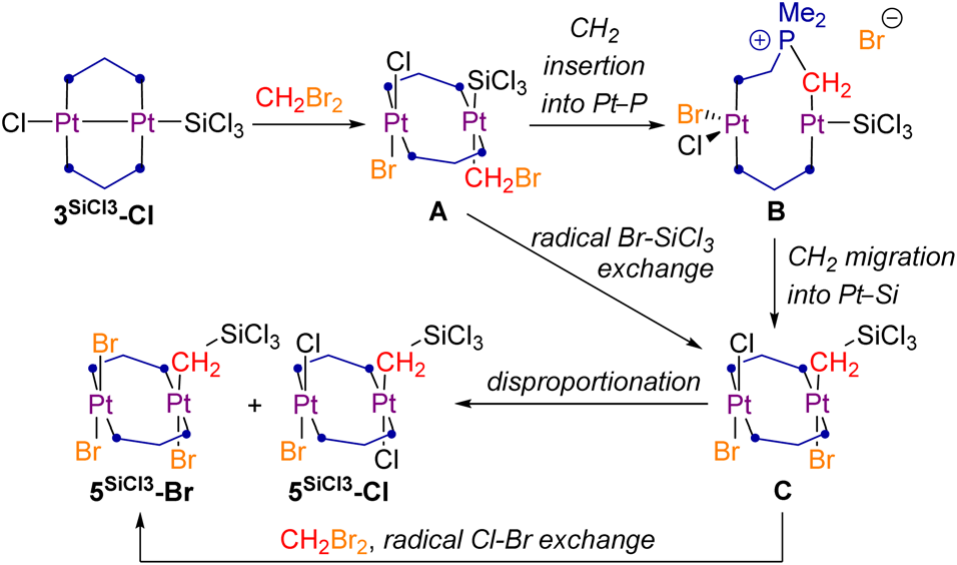
Possible mechanisms for the formation of 5^SiCl3^-X from 3^SiCl3^-Cl and CH_2_Br_2_.

Based on these studies, it is possible that the formation of 5^SiCl3^-Cl proceeds first *via* a formal transannular, likely radical oxidative addition of Br˙ and ˙CH_2_Br, yielding the unstable *trans*-(bromomethyl)silyl complex A (Scheme 2). Subsequent insertion of the CH_2_ unit into one of the Pt–P bonds and displacement of the bromide anion yields the phosphonium ylide B (or its dicationic Pt–Pt-bonded analogue if the second bromide also gets displaced). Complex B then undergoes CH_2_ migration into the Pt–Si bond, with concomitant reformation of the Pt–P bond and bromide addition to Pt2, yielding the silyl(trihalo)diplatinum(ii) complex C. Alternatively, a radical Br–SiCl_3_ exchange at A could lead directly to C. Finally, C undergoes radical or ionic disproportionation to 5^SiCl3^-Cl and 5^SiCl3^-Br, or radical Cl–Br exchange to yield the observed excess of 5^SiCl3^-Br.

### Comparative computational study of the reactivity of 1 with CH_2_Cl_2_, CCl_4_ and SiCl_4_

In order to understand the divergent reactivity of 1 towards dihalomethanes and polychlorosilanes, quantum-chemical calculations using density functional theory (DFT) were undertaken. All computations were performed at the ZORA^44^-BLYP^45^-D3(BJ)^46^/TZ2P^47^ level of theory, using the conductor-like screening model (COSMO)^48^ to simulate solvation in benzene (see the full computational details in the SI). This level of theory has been proven to be accurate for studying bond activation by group 10 metals and metal–metal interactions, both in previous reports,^49,50^ as well as in the additional performance test carried out in the current work (see details in the SI). The optimised structures of 2^CH2^-Cl and 3^SiCl3^-Cl at this level of theory are in good agreement with the experimental SCXRD-derived structures, notwithstanding small discrepancies attributable to the fact that the computed structures are optimised in solution rather than the solid state (see Fig. S78 in the SI).

**Fig. 7:**
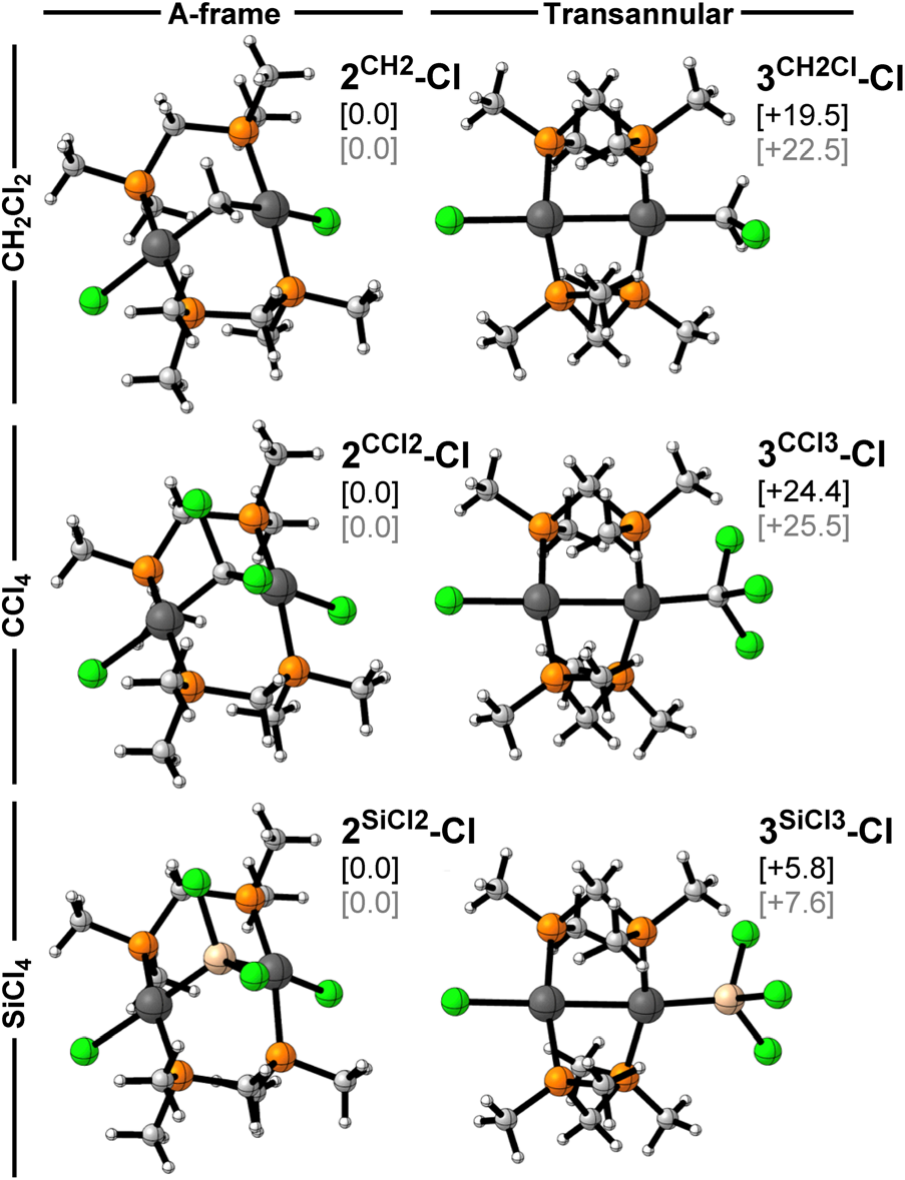
Ball-and-stick structures, relative Gibbs free energies Δ*G* (in black, kcal mol^−1^), and relative electronic energies Δ*E* (in grey, kcal mol^−1^) of the optimised A-frame (left) and transannular oxidative addition (right) products of CH_2_Cl_2_, CCl_4_, and SiCl_4_ with complex 1, calculated at the COSMO(benzene)-ZORA-BLYP-D3(BJ)/TZ2P level of theory. Atom colours: H = white; C = grey; P = orange; Cl = green; Si = beige; Pt = anthracite.

In order to identify the origin of the divergent reactivity of 1 towards CY_2_Cl_2_ (Y = H, Cl) and SiCl_4_, the relative stability of the two possible isomeric products, *i.e.* the methylene/silylene-bridged A-frame, and the product of transannular C/Si–Cl addition, was first investigated. The optimised geometries and relative energies are presented in Fig. 7. In all three cases the A-frame complex is the most stable species and thus corresponds to the thermodynamic product. This aligns with the experimentally observed eventual rearrangement of 3^SiCl3^-Cl to 2^SiCl2^-Cl upon prolonged heating in CH_2_Cl_2_. It is noteworthy that the energetic preference for the A-frame is significantly more pronounced for the halomethanes (Δ*G* ≥ 19.5 kcal mol^−1^) than the halosilane (Δ*G* = 5.8 kcal mol^−1^), which is consistent with the sole observation of the A-frame product in the former systems.

**Fig. 8:**
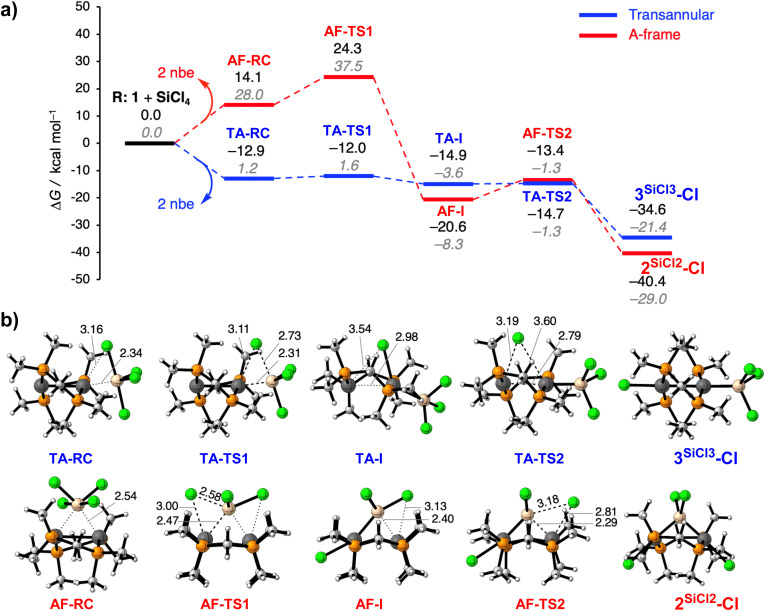
(a) Reaction profiles of the transannular addition (TA, blue pathway) and direct A-frame formation (AF, red pathway) of SiCl_4_ with 1. Relative Gibbs free energies Δ*G* (in kcal mol^−1^) and electronic energies Δ*E* (in grey italics). (b) Corresponding stationary point structures with relevant bond lengths (in Å). All computations were carried out at the COSMO(benzene)-ZORA-BLYP-D3(BJ)/TZ2P level of theory. R = reactants; RC = reactant complex; TS = transition state; I = intermediate. Note that the fourth chlorine atom of SiCl_4_ appears hidden in the orientation of AF-TS1, AF-I, and AF-TS2.

Although the A-frame product is thermodynamically favoured, all halosilane substrates yield the transannular diplatinum(i) complex at rt, which is therefore the kinetic product, associated with the lowest activation barrier in the selectivity-determining step. This is confirmed by our DFT calculations, which reveal two distinct two-step reaction mechanisms, one for the transannular oxidative addition of SiCl_4_ to 1, yielding 3^SiCl3^-Cl (TA pathway in blue, Fig. 8a), and the other for the direct twofold oxidative addition, yielding the A-frame 2^SiCl2^-Cl (AF pathway in red, Fig. 8a). In both cases SiCl_4_ first coordinates to 1 with release of the nbe ligands to form a hypervalent reactant complex (RC), a typical feature of silicon chemistry that cannot be accessed by the halocarbon analogues.^51^ This can occur either on one side of the diplatinum complex, yielding the transannular RC (TA-RC), or in the centre above the Pt⋯Pt unit, yielding the A-frame RC (AF-RC, Fig. 8b). The nature of the [SiCl_4_⋯1] interaction in both reactant complexes was assessed in more detail using quantitative energy decomposition analysis (see Table S1 in the SI).^52^ The results show that the electrostatic and orbital interactions are the main stabilising bonding components in both complexes and of similar magnitude. The main difference lies in the higher steric Pauli repulsion experienced by AF-RC as the SiCl_4_ molecule sits atop the centre of 1, rather than on one side. As a result, the formation of TA-RC is exergonic by Δ*G*_1_ = −12.9 kcal mol^−1^, whereas that of AF-RC is endergonic by Δ*G*_1_ = +14.1 kcal mol^−1^. Note that CCl_4_ and CH_2_Cl_2_ cannot form reactant complexes of this nature because the central carbon atom cannot become hypervalent, and the C–Cl bonds are relatively shorter and too rigid to allow this degree of bond angle deformation. Instead, CCl_4_ and CH_2_Cl_2_ form a reactant complex by coordinating on top of 1 through one of the Cl atoms (see Fig. S79 in the SI), thereby favouring an AF pathway.

Once the RC is formed, both pathways proceed with the selectivity-determining step, the oxidative addition of one Si–Cl bond at one of the Pt centres (Fig. 8a). In the corresponding transition-state (TS) structure of the AF pathway, AF-TS1, the SiCl_4_ moiety is highly deformed, resulting in a very high overall first activation barrier of Δ*G*_1_^‡^ = +24.3 kcal mol^−1^. In contrast, the TS structure of the first oxidative addition of the TA pathway, TA-TS1, strongly resembles that of TA-RC, resulting in a very low first activation barrier of Δ*G*_1_^‡^ = +0.9 kcal mol^−1^. In both cases an intermediate mixed-valence Pt(ii)–Pt(0) complex is formed, in which the Pt(0) centre remains dicoordinate and the Pt(ii) centre displays a distorted square-planar geometry, with either the silyl (AF-I) or the chloride (TA-I) ligand positioned above the Pt⋯Pt unit. While the formation of AF-I from AF-RC is highly exergonic (Δ*G*_2_ = −34.7 kcal mol^−1^), that of TA-I from TA-RC is only mildly so (Δ*G*_2_ = −2.0 kcal mol^−1^), making the latter process actually reversible. While the AF pathway is thus highly thermodynamically favoured, the TA pathway is kinetically much more accessible, and therefore always preferred.

In the AF pathway AF-I then undergoes a second Si–Cl bond addition at the remaining Pt(0) centre *via*AF-TS2 yielding the silylene-bridged 2^SiCl2^-Cl, with a much lower barrier than for the first addition (Δ*G*_2_^‡^ = +7.2 kcal mol^−1^). In the TA pathway the transfer of the second chloride to the second Pt centre, which yields 3^SiCl3^-Cl, is virtually barrierless (Δ*G*_2_^‡^ = +0.2 kcal mol^−1^). Consequently, the TA pathway is kinetically favoured for both reaction steps. Formation of the thermodynamically favoured silylene-bridged A-frame 2^SiCl2^-Cl could only be achieved by heating 3^SiCl3^-Cl in CH_2_Cl_2_ for four days (see Scheme 1e). The kinetic preference for the TA product is expected to be even more pronounced for the methylsilyl derivatives, as the larger methyl groups would render the formation of AF-RC and AF-TS1 even less favourable. In addition, the non-coordinating and larger methyl groups likely decrease, or even invert, the thermodynamic preference for the AF relative to the TA pathway, as additional steric clashes between the silane and Pt complex methyl groups would induce even larger Pauli repulsion in the AF reactant complexes and transition states (see SI Table S1). As a result, only 3^SiCl3^-Cl fully rearranges to 2^SiCl2^-Cl, whereas 3^SiMeCl2^-Cl rearranges only partially, and 3^SiMe2Cl^-Cl and 3^SiMe3^-Cl do not.

## Conclusion

We demonstrated in this work the unique cooperative reactivity that a dinuclear platinum complex, [(µ-dmpm)_2_Pt_2_(nbe)_2_] (1), exhibits towards the double activation of C–X and Si–X bonds of CH_2_X_2_ (X = Cl, Br, I), CCl_4_ and Me_4−*n*_SiX_*n*_ (X = Cl, *n* = 1–4; X = I, *n* = 1), respectively. Upon reaction with di- and tetrahalomethanes, complex 1 exclusively forms methylene-bridged A-frame structures (2^CY2^-X), while halosilanes undergo transannular oxidative addition reactions in which one Si–X bond is added across the two metal centres, leading to the formation of unsymmetrical silyldiplatinum(i) complexes (3^SiMe(3−^*^n^*^)X^*^n^*-X). Upon heating in dichloromethane the transannular addition product of 1 and SiCl_4_, complex 3^SiCl3^-Cl, fully rearranges to the corresponding silylene-bridged A-frame complex 2^SiCl2^-Cl, while its MeSiCl_3_-derived analogue, 3^SiMeCl2^-Cl, undergoes only partial rearrangement. In contrast, heating in dibromomethane results in a highly unusual insertion of a CH_2_Br_2_-derived methylene unit into the Pt–Si bond (5^SiCl3^-X).

DFT computations reveal that the methylene/silylene-bridged A-frame complexes are always the thermodynamic products, albeit with a much stronger preference for the A-frames in the halomethane-based reactions. For SiCl_4_ the direct two-step formation of the A-frame complex 2^SiCl2^-Cl has a much higher activation barrier than the pathway leading to the kinetic transannular addition product 3^SiCl3^-Cl. The propensity towards rearrangement of the transannular addition product 3^SiMe(3−^*^n^*^)Cl^*^n^*-Cl to the A-frame is dictated by the number of methyl groups, as these clash with the dmpm methyl groups during the rearrangement.

The divergent reaction pathways described herein highlight the potential of bimetallic complexes in tuning cooperative bond activations, which could be integrated into new methylene/silylene transfer or silylation reactions. Further investigations to broaden the substrate scope and ultimately harness the cooperativity of these diplatinum complexes in catalytic transformations are underway in our laboratory.

## Author contributions

M. P. designed the study, carried out the experimental work and wrote the SI. C. N. carried out and wrote the computational contributions. M. A. wrote the manuscript. H. B. and M. B. provided supervision and funding.

## Conflicts of interest

The authors declare no conflict of interest.

## Supplementary Material

SC-017-D6SC00337K-s001

SC-017-D6SC00337K-s002

## Data Availability

CCDC 2406490 (2^CH2^-Cl), 2406494 (2^CH2^-Br), 2406498 (2^CH2^-I), 2406509 (2^CH2^-Br′), 2406550 (3^SiMe3^-I), 2406556 (3^SiMe2Cl^-Cl), 2406558 (3^SiMeCl2^-Cl), 2406564 (3^SiCl3^-Cl), 2505905 (4^SiCl3^-HCl), 2406843 (2^SiCl2^-Cl) and 2406901 (5^SiCl3^-Br) contain the supplementary crystallographic data for this paper.^54*a*–*k*^ The data supporting this article have been included as part of the supplementary information (SI). Supplementary information: methods, synthetic procedures, NMR spectra, X-ray crystallographic and computational details. See DOI: https://doi.org/10.1039/d6sc00337k.
