# Supplementary material for: Cooperative reactivity of halomethanes and -silanes at an A-frame complex: transannular addition versus bridging tetrylenes
Source: Chem Sci. 2026 Apr 15;17(21):10547–58. doi: 10.1039/d6sc00337k (PMC13085931; doi:10.1039/d6sc00337k)
Supplement: SC-017-D6SC00337K-s001 [file SC-017-D6SC00337K-s001.pdf]

Supplementary Information (SI) for Chemical Science.
This journal is © The Royal Society of Chemistry 2026

Supporting Information for:

Cooperative Reactivity of Halomethanes and -silanes at an A-Frame Complex: Transannular Addition versus Bridging Tetrylenes

Max Passargus,[a,b] Celine Nieuwland,[c] Merle Arrowsmith,[a,b] F. Matthias Bickelhaupt,*[c,d,e] and Holger Braunschweig*[a,b]

[a] *Institute for Inorganic Chemistry, Julius-Maximilians-Universität Würzburg, Am Hubland, 97074 Würzburg, Germany.* [b] *Institute for Sustainable Chemistry & Catalysis with Boron, Julius-Maximilians-Universität Würzburg, Am Hubland, 97074 Würzburg, Germany.* [c] *Department of Chemistry and Pharmaceutical Sciences, Amsterdam Institute of Molecular and Life Sciences (AIMMS), Vrije Universiteit Amsterdam, De Boelelaan 1108, 1081 HZ Amsterdam, The Netherlands.* [d] *Institute for Molecules and Materials, Radboud University, Heyendaalseweg 135, 6525 AJ Nijmegen, The Netherlands.* [e] *Department of Chemical Sciences, University of Johannesburg, Auckland Park, Johannesburg 2006, South Africa.*

Contents

Methods and materials 2

Synthetic procedures 3

NMR spectra of new compounds 11

Crystallographic data 75

Computational details 87

References 114

Methods and materials

All manipulations were performed under an atmosphere of dry argon or in vacuo using standard Schlenk line or glovebox techniques. Deuterated solvents were dried over molecular sieves and degassed by three freeze-pump-thaw cycles before use. All other solvents were distilled and degassed from appropriate drying agents. Solvents (both deuterated and non-deuterated) were stored under argon over activated 4 Å molecular sieves. NMR spectra were acquired on a Bruker Avance 500 NMR spectrometer (^{1}H and $^{1}H\{^{31}P\}$: 500.1 MHz, $^{11}B\{^{1}H\}$: 160.5 MHz, $^{13}C\{^{1}H\}$: 125.8 MHz, $^{31}P\{^{1}H\}$: 202.5 MHz, $^{29}Si\{^{1}H\}$: 99.4 MHz, $^{195}Pt\{^{1}H\}$: 107.5 MHz), Bruker Avance 400 NMR spectrometer ($^{11}B\{^{1}H\}$: 128.4 MHz, $^{31}P\{^{1}H\}$: 162.2 MHz) and Bruker Avance 600 NMR spectrometer (^{1}H and $^{1}H\{^{31}P\}$: 600.2 MHz, $^{11}B\{^{1}H\}$: 192.6 MHz, $^{13}C\{^{1}H\}$: 150.9 MHz, $^{31}P\{^{1}H\}$: 243.0 MHz, $^{29}Si\{^{1}H\}$: 119.2 MHz, $^{195}Pt\{^{11}B, ^{1}H\}$: 129.0 MHz). Chemical shifts (δ) are given in ppm and internally referenced to the carbon nuclei ($^{13}C\{^{1}H\}$) or residual protons (^{1}H) of the solvent. The $^{11}B\{^{1}H\}$, $^{31}P\{^{1}H\}$, $^{29}Si\{^{1}H\}$ and $^{195}Pt\{^{11}B, ^{1}H\}$ spectra were referenced to [$BF_3 \cdot OEt_2$], 85% H_3PO_4, $SiMe_4$ and $Na_2[PtCl_6]$, respectively, as external standards. NMR multiplicities are given as s (singlet), t (triplet), tt (triplet of triplets), ttt (triplet of triplets of triplets), quint (quintet), m (multiplet), app (apparent), br (broad), n_{+sat} (n + satellites). Microanalyses (C, H, N) were performed on an Elementar vario MICRO cube elemental analyzer. High-resolution mass spectrometry (HRMS) data were obtained from a Thermo Scientific Exactive Plus spectrometer. Solvents and reagents were purchased from Sigma Aldrich, Alfa Aesar, Fluorochem or TCI and transferred to the glovebox for use. Compound **1** was synthesised following a literature procedure.[1]

Synthetic procedures

General remarks

The isolated complexes systematically slowly decomposed under reduced pressure or in solution, independent of the choice of solvent. The major decomposition product was always the corresponding dihalodiplatinum(I) complex [(μ–dmpm)$_2$Pt$_2$X$_2$] (X = Cl: δ_{31P} = –19.3 (m$_{+sat}$, $^1J_{P–Pt}$ = 2654 Hz) ppm; δ_{195Pt} = 4766 (s$_{+sat}$) pm);[2] X = Br: δ_{31P} = –23.4 (m$_{+sat}$, $^1J_{P–Pt}$ = 2640 Hz) ppm;[1] X = I: δ_{31P} = –31.4 (m$_{+sat}$, $^1J_{P–Pt}$ = 2258 Hz) ppm[3]). The fate of the extruded (in)organic fragments could not be determined as the other byproduct(s) precipitated as amorphous colourless solids, which proved insoluble in all solvents.

Synthesis of 2^{CH2}-Cl

In a vial (5 mL), complex **1** (20.0 mg, 23.5 µmol) was dissolved in benzene (0.7 mL) and one equiv. of dichloromethane (DCM, 2.00 mg, 23.5 µmol) was added. The mixture was stirred for 5 min at rt, resulting in a yellow suspension. After evaporating the volatiles at ambient pressure, the residual solid was washed with pentane (3 x 2 mL) and benzene (2 x 2 mL). Recrystallisation from DCM/pentane yielded complex **2^{CH2}-Cl** as a yellow solid (13.1 mg, 17.5 µmol, 75%). ^{1}H NMR (500.1 MHz, CD$_2$Cl$_2$, 298 K): δ = 2.62–2.52 (m, 2H, P$_2$CH_2), 1.85–1.68 (m, 2H, P$_2$CH_2), 1.71 (s$_{+sat}$, $^3J_{H–Pt}$ = 27.5 Hz, 12H, PCH_3), 1.66 (s$_{+sat}$, $^3J_{H–Pt}$ = 33.9 Hz, 12H, PCH_3), 0.87 (quint$_{+sat}$, $^3J_{H–P}$ = 9.0 Hz, $^2J_{H–Pt}$ = 57.8 Hz, 2H, Pt$_2$CH_2) ppm. ^{13}C{^{1}H} NMR (125.8 MHz, CD$_2$Cl$_2$, 298 K): δ = 22.0 (m, P$_2$$CH_2$), 15.2 (m, P$CH_3$), 13.8 (m, P$CH_3$), –14.5 (quint$_{+sat}$, $^2J_{C–P}$ = 3.9 Hz, $^1J_{C–Pt}$ = 613 Hz, Pt$_2$$CH_2$) ppm. ^{31}P{^{1}H} NMR (202.5 MHz, CD$_2$Cl$_2$, 298 K): δ = –7.3 (s$_{+sat}$, $^1J_{P–Pt}$ = 3072 Hz, $^3J_{P–Pt}$ = 73 Hz, $^2J_{Pt-Pt}$ = 842 Hz) ppm. ^{195}Pt{^{1}H} NMR (107.5 MHz, CD$_2$Cl$_2$, 298 K): δ = –4267 (tt$_{+sat}$, $^1J_{Pt–P}$ = 3072 Hz, $^3J_{Pt–P}$ = 73 Hz, $^2J_{Pt-Pt}$ = 842 Hz) ppm. Elemental analysis (%) calculated for [C$_{11}$H$_{30}$Cl$_2$P$_4$Pt$_2$] (M$_w$ = 747.3): C 17.68, H 4.05; found: C 17.99, H 3.99.

Synthesis of 2^{CH2}-Br

In a vial (5 mL), complex **1** (20.0 mg, 23.5 µmol) was dissolved in benzene (0.7 mL), then one equiv. dibromomethane (DBM, 4.09 mg, 23.5 µmol) was added. The mixture was stirred for 5 min at rt, resulting in a yellow suspension. After evaporating the volatiles at ambient pressure, the residual solid was washed with pentane (3 x 2 mL) and benzene (2 x 2 mL). Recrystallisation from DCM/pentane yielded complex **2^{CH2}-Br** as a yellow solid (18.3 mg, 21.9 µmol, 75%). *Note: the synthesis systematically yielded a yellow byproduct, which proved*

*insoluble in all common organic solvents, and was identified by single-crystal X-ray diffraction (SCXRD) analysis as the product of the twofold addition of DMB across both Pt centers, complex **2^{CH2}-Br'**. The reaction of **1** with an excess of DBM did not result in improved yields of **2^{CH2}-Br'**.* ^{1}H NMR (600.2 MHz, CD_2Cl_2, 298 K): δ = 2.67–2.59 (m, 2H, P_2CH_2), 1.80 (s_{+sat}, $^{3}J_{H–Pt}$ = 27.6 Hz, 12H, PCH_3), 1.77–1.71 (m, 2H, P_2CH_2), 1.69 (s_{+sat}, $^{3}J_{H–Pt}$ = 33.6 Hz, 12H, PCH_3), 1.07 ($quint_{+sat}$, $^{3}J_{H–P}$ = 9.1 Hz, $^{2}J_{H–Pt}$ = 56.7 Hz, 2H, Pt_2CH_2) ppm. ^{13}C{^{1}H} NMR (150.9 MHz, CD_2Cl_2, 298 K): δ = 21.1 (m, P_2CH_2), 15.6 (m, PCH_3), 15.5 (m, PCH_3), –7.6 ($quint_{+sat}$, $^{2}J_{C–P}$ = 3.7 Hz, $^{1}J_{C–Pt}$ = 617 Hz, Pt_2CH_2) ppm. ^{31}P{^{1}H} NMR (243.0 MHz, CD_2Cl_2, 298 K): δ = –9.6 (s_{+sat}, $^{1}J_{P–Pt}$ = 3036 Hz, $^{3}J_{P–Pt}$ = 71 Hz, $^{2}J_{Pt-Pt}$ = 831 Hz) ppm. ^{195}Pt{^{1}H} NMR (129.0 MHz, CD_2Cl_2, 298 K): δ = –4397 (tt_{+sat}, $^{1}J_{P–Pt}$ = 3036 Hz, $^{3}J_{P–Pt}$ = 71 Hz, $^{2}J_{Pt-Pt}$ = 831 Hz) ppm. Elemental analysis (%) calculated for [$C_{11}H_{30}Br_2P_4Pt_2$] (M_w = 836.2): C 15.80, H 3.62; found: C 15.95, H 3.47.

Synthesis of 2^{CH2}-I

In a vial (5 mL), complex **2^{CH2}-Br** (20.0 mg, 23.9 µmol) was suspended in benzene (0.7 mL), then three equiv. of trimethylsilyl iodide (TMSI, 14.4 mg, 71.8 µmol) were added. The resulting mixture was stirred for 5 min at rt, resulting in a red suspension. After evaporating the volatiles at ambient pressure, the residual solid was washed with pentane (3 x 2 mL) and benzene (2 x 2 mL). Recrystallisation from DCM/pentane afforded complex **2^{CH2}-I** as a brown solid (21.6 mg, 23.2 µmol, 97%). *Note: this synthetic route to **2^{CH2}-I** proved more reliable than the reaction of **1** with one equiv. CH_2I_2.* ^{1}H NMR (500.1 MHz, CD_2Cl_2, 298 K): δ = 2.78–2.68 (m, 2H, P_2CH_2), 1.94 (s_{+sat}, $^{3}J_{H–Pt}$ = 28.5 Hz, 12H, PCH_3), 1.76–1.63 (m, 2H, P_2CH_2), 1.74 (s_{+sat}, $^{3}J_{H–Pt}$ = 33.5 Hz, 12H, PCH_3), 1.47 ($quint_{+sat}$, $^{3}J_{H–P}$ = 9.2 Hz, $^{2}J_{H–Pt}$ = 55.0 Hz, 2H, Pt_2CH_2) ppm. ^{13}C{^{1}H} NMR (125.8 MHz, CD_2Cl_2, 298 K): δ = 19.5 (m, P_2CH_2), 19.1 (m, PCH_3), 16.2 (m, PCH_3), 4.5 (quint, $^{2}J_{C–P}$ = 3.9 Hz, Pt_2CH_2) ppm. ^{31}P{^{1}H} NMR (202.5 MHz, CD_2Cl_2, 298 K): δ = –13.2 (s_{+sat}, $^{1}J_{P–Pt}$ = 2982 Hz, $^{3}J_{P–Pt}$ = 68 Hz, $^{2}J_{Pt-Pt}$ = 754 Hz) ppm. ^{195}Pt{^{1}H} NMR (107.5 MHz, CD_2Cl_2, 298 K): δ = –4645 (tt_{+sat}, $^{1}J_{Pt–P}$ = 298 Hz, $^{3}J_{Pt–P}$ = 68 Hz, $^{2}J_{Pt-Pt}$ = 754 Hz) ppm. Elemental analysis (%) calculated for [$C_{11}H_{30}I_2P_4Pt_2$] (M_w = 930.2): C 14.20, H 3.25; found: C 14.23, H 3.00.

Synthesis of 2^{CCl2}-Cl

In a vial (5 mL), complex **1** (20.0 mg, 23.5 µmol) was dissolved in benzene (0.7 mL) and one equiv. of tetrachloromethane (2.28 µL, 23.5 µmol) was added. The mixture was stirred for 5 min at rt, resulting in an orange suspension. After evaporating the volatiles at ambient pressure,

the residual solid was washed with pentane (3 x 2 mL) and benzene (2 x 2 mL), yielding **2^{CCl2}-Cl** as an orange solid (17.4 mg, 21.3 μmol, 91%). *Note: **2^{CCl2}-Cl** is only sparingly soluble in $CDCl_3$ and undergoes complete decomposition within two days in solution. Owing to its rapid degradation, it could not be fully characterised.* ^{1}H NMR (500.1 MHz, CD_2Cl_2, 298 K): δ = 3.68–3.47 (m, 2H, P_2CH_2), 1.97 (s$_{+sat}$, $^3J_{H–Pt}$ = 40.0 Hz, 12H, PCH_3), 1.86–1.78 (m, 2H, P_2CH_2), 1.82 (s$_{+sat}$, $^3J_{H–Pt}$ = 27.2 Hz, 12H, PCH_3) ppm. ^{13}C{^{1}H} NMR (125.8 MHz, CD_2Cl_2, 298 K): δ = 20.3 (m, P_2CCl_2), 13.0 (m, PCH_3), 11.8 (m, PCH_3) ppm. ^{31}P{^{1}H} NMR (202.5 MHz, $CDCl_3$, 298 K): δ = –8.7 (s$_{+sat}$, $^1J_{P–Pt}$ = 2750 Hz, $^3J_{P–Pt}$ = 150 Hz) ppm. ^{195}Pt{^{1}H} NMR (107.5 MHz, CD_2Cl_2, 298 K): δ = –4013 (t, $^1J_{P–Pt}$ = 2750 Hz, *detected by ^{1}H-^{195}Pt HMQC*) ppm.

Synthesis of 3^{SiMe3}-Cl

In a vial (5 mL), complex **1** (20.0 mg, 23.5 μmol) was dissolved in benzene (0.7 mL), then one equiv. of chlorotrimethylsilane (TMSCl, 2.55 mg, 23.5 μmol) was added. The resulting mixture was heated to 60 °C for 16 h. After evaporating the volatiles at ambient pressure, the residual solid was washed with pentane (3 x 2 mL). Recrystallisation from 1,2-difluorobenzene (*o*-DFB)/pentane afforded complex **3^{SiMe3}-Cl** as a brownish solid (14.4 mg, 18.2 μmol, 77%). ^{1}H NMR (500.1 MHz, C_6D_6, 298 K): δ = 2.36–2.14 (m, 4H, P_2CH_2), 1.50 (t$_{+sat}$, $^2J_{H–P}$ = 3.1 Hz, $^3J_{H–Pt}$ = 32.9 Hz, 12H, PCH_3), 1.35 (t$_{+sat}$, $^2J_{H–P}$ = 3.1 Hz, $^3J_{H–Pt}$ = 32.4 Hz, 12H, PCH_3), 0.45 (s$_{+sat}$, $^2J_{H–Si}$ = 9.5 Hz, 9H, $SiCH_3$) ppm. ^{13}C{^{1}H} NMR (125.8 MHz, C_6D_6, 298 K): δ = 44.7 (t, $^1J_{C–P}$ = 19.2 Hz, P_2CH_2), 19.9 (app. tt, $^1J_{C–P}$ = 18.5 Hz, $^3J_{C–P}$ = 3.3 Hz, PCH_3), 15.2 (app. tt, $^1J_{C–P}$ = 17.0 Hz, $^3J_{C–P}$ = 2.7 Hz, PCH_3), 9.2 (t$_{+sat}$, $^3J_{C–P}$ = 3.2 Hz, $^1J_{C–Si}$ = 61.7 Hz, $SiCH_3$) ppm. ^{29}Si{^{1}H} NMR (99.4 MHz, C_6D_6, 298 K): δ = –10.9 (tt$_{+sat}$, $^2J_{Si–P}$ = 10.2 Hz, $^3J_{Si–P}$ = 2.4 Hz, $^1J_{Si–Pt}$ = 994 Hz, $^2J_{Si–Pt}$ = 273 Hz, *Si*CH$_3$) ppm. ^{31}P{^{1}H} NMR (202.5 MHz, C_6D_6, 298 K): δ = –15.6 (m$_{+sat}$, $^1J_{P–Pt}$ = 3160 Hz, $^2J_{P–Pt}$ = 48 Hz, 2P, *P*$_2$PtCl), –25.7 (m$_{+sat}$, $^1J_{P–Pt}$ = 2671 Hz, $^2J_{P–Pt}$ = 122 Hz, 2P, *P*$_2$PtSi) ppm. ^{195}Pt{^{1}H} NMR (107.5 MHz, C_6D_6, 298 K): δ = –4317 (tt$_{+sat}$, $^1J_{Pt–P}$ = 3160 Hz, $^2J_{Pt–P}$ = 122 Hz, $^1J_{Pt–Pt}$ = 3060 Hz, 1Pt, P$_2$*Pt*Cl), –4798 (tt$_{+sat}$, $^1J_{Pt–P}$ = 2671 Hz, $^2J_{Pt–P}$ = 48 Hz, $^1J_{Pt–Pt}$ = 3060 Hz, 1Pt, P$_2$*Pt*Si) ppm. Elemental analysis (%) calculated for [$C_{13}H_{37}ClP_4Pt_2Si$] (M_w = 771.0): C 20.25, H 4.84, found: C 20.19, H 4.69.

Synthesis of 3^{SiMe3}-I

In a vial (5 mL), complex **1** (20.0 mg, 23.5 μmol) was dissolved in benzene (0.7 mL), then one equiv. of TMSI (4.70 mg, 23.5 μmol) was added. The resulting mixture was stirred for 3 d at rt. After evaporating the volatiles at ambient pressure, the residual solid was washed with

pentane (3 x 2 mL). Recrystallisation from benzene/pentane yielded complex **3^{SiMe3}-I** as an orange solid (10.5 mg, 12.2 µmol, 52%). ^{1}H NMR (500.1 MHz, C_6D_6, 298 K): δ = 2.38–2.17 (m, 4H, P_2CH_2), 1.53 (t_{+sat}, $^2J_{H-P}$ = 3.1 Hz, $^3J_{H-Pt}$ = 32.9 Hz, 12H, PCH_3), 1.43 (t_{+sat}, $^2J_{H-P}$ = 3.1 Hz, $^3J_{H-Pt}$ = 32.2 Hz, 12H, PCH_3), 0.44 (s_{+sat}, $^2J_{H-Si}$ = 10.1 Hz, 9H, $SiCH_3$) ppm. $^{13}C\{^1H\}$ NMR (125.8 MHz, C_6D_6, 298 K): δ = 44.2 (m, P_2CH_2), 19.2 (app. tt, $^1J_{C-P}$ = 18.8 Hz, $^3J_{C-P}$ = 3.6 Hz, PCH_3), 18.7 (app. tt, $^1J_{C-P}$ = 17.5 Hz, $^3J_{C-P}$ = 2.8 Hz, PCH_3), 9.2 (t_{+sat}, $^3J_{C-P}$ = 3.5 Hz, $^1J_{C-Si}$ = 63.5 Hz, $SiCH_3$) ppm. $^{29}Si\{^1H\}$ NMR (99.4 MHz, C_6D_6, 298 K): δ = –10.1 (tt_{+sat}, $^2J_{Si-P}$ = 9.5 Hz, $^3J_{Si-P}$ = 4.1 Hz, $^1J_{Si-Pt}$ = 994 Hz, $^2J_{Si-Pt}$ = 286 Hz, *Si*CH_3) ppm. $^{31}P\{^1H\}$ NMR (202.5 MHz, C_6D_6, 298 K): δ = –21.4 (m_{+sat}, $^1J_{P-Pt}$ = 3132 Hz, $^2J_{P-Pt}$ = 57 Hz, 2P, P_2PtI), –26.5 (m_{+sat}, $^1J_{P-Pt}$ = 2656 Hz, $^2J_{P-Pt}$ = 116 Hz, 2P, P_2PtSi) ppm. $^{195}Pt\{^1H\}$ NMR (107.5 MHz, C_6D_6, 298 K): δ = –4686 (tt_{+sat}, $^1J_{Pt-P}$ = 3153 Hz, $^2J_{Pt-P}$ = 116 Hz, 1Pt, P_2*Pt*I), –4755 (tt_{+sat}, $^1J_{Pt-P}$ = 2675 Hz, $^2J_{Pt-P}$ = 57 Hz, 1Pt, P_2*Pt*Si) ppm. Elemental analysis (%) calculated for [$C_{13}H_{37}IP_4Pt_2Si\cdot(C_6H_6)_{0.1}$] ($M_w$ = 870.3): C 18.65, H 4.30, found: C 18.77, H 4.35.

Synthesis of $3^{SiMe2Cl}$-Cl

In a vial (5 mL), complex **1** (20.0 mg, 23.5 µmol) was dissolved in benzene (0.7 mL), then one equiv. of dichlorodimethylsilane (3.03 mg, 23.5 µmol) was added. The resulting mixture was stirred for 2 d at rt. After evaporating the volatiles at ambient pressure, the residual solid was washed with pentane (3 x 2 mL). Recrystallisation from benzene/pentane yielded **$3^{SiMe2Cl}$-Cl** as an off-white solid (14.2 mg, 17.9 µmol, 76%). ^{1}H NMR (600.2 MHz, C_6D_6, 298 K): δ = 2.33–2.15 (m, 4H, P_2CH_2), 1.55 (t_{+sat}, $^2J_{H-P}$ = 3.0 Hz, $^3J_{H-Pt}$ = 32.2 Hz, 12H, PCH_3), 1.32 (t_{+sat}, $^2J_{H-P}$ = 3.0 Hz, $^3J_{H-Pt}$ = 31.4 Hz, 12H, PCH_3), 0.83 (s_{+sat}, $^2J_{H-Si}$ = 7.9 Hz, 6H, $SiCH_3$) ppm. $^{13}C\{^1H\}$ NMR (150.9 MHz, C_6D_6, 298 K): δ = 44.2 (t, $^1J_{C-P}$ = 18.8 Hz, P_2CH_2), 19.7 (m, PCH_3), 15.2 (m, PCH_3), 13.8 (t, $^3J_{C-P}$ = 2.1 Hz, $SiCH_3$) ppm. $^{29}Si\{^1H\}$ NMR (119.2 MHz, C_6D_6, 298 K): δ = 38.5 (t_{+sat}, $^2J_{Si-P}$ = 12 Hz, $^1J_{Si-Pt}$ = 1269 Hz, $^2J_{Si-Pt}$ = 398 Hz, *Si*CH_3) ppm. $^{31}P\{^1H\}$ NMR (243.0 MHz, C_6D_6, 298 K): δ = –17.5 (m_{+sat}, $^1J_{P-Pt}$ = 3035 Hz, 2P, P_2PtCl), –26.4 (m_{+sat}, $^1J_{P-Pt}$ = 2581 Hz, $^2J_{P-Pt}$ = 138 Hz, 2P, P_2PtSi) ppm. $^{195}Pt\{^1H\}$ NMR (129.0 MHz, C_6D_6, 298 K): δ = –4309 (tt_{+sat}, $^1J_{Pt-P}$ = 3035 Hz, $^2J_{Pt-P}$ = 138 Hz, $^1J_{Pt-Pt}$ = 3269 Hz, 1Pt, P_2*Pt*Cl), –4748 (tt_{+sat}, $^1J_{Pt-P}$ = 2581 Hz, $^2J_{Pt-P}$ = 52 Hz, $^1J_{Pt-Pt}$ = 3269 Hz, 1Pt, P_2*Pt*Si) ppm. Elemental analysis (%) calculated for [$C_{12}H_{34}Cl_2P_4Pt_2Si\cdot(C_6H_6)_{0.3}$] ($M_w$ = 814.9): C 20.34, H 4.43, found: C 20.37, H 4.36. LIFDI-HRMS (toluene, *m/z*) calculated for $C_{12}H_{34}Cl_2P_4Pt_2Si$ = [**$3^{SiMe2Cl}$-Cl** + H]$^+$: 791.0104; found: 791.0101.

Synthesis of $3^{SiMeCl2}$-Cl

In a vial (5 mL), complex 1 (20.0 mg, 23.5 μmol) was dissolved in benzene (0.7 mL), then one equiv. of trichloromethylsilane (3.51 mg, 23.5 μmol) was added. The mixture was stirred for 5 min at rt, resulting in a yellow suspension. After evaporating the volatiles at ambient pressure, the residual solid was washed with pentane (3 x 2 mL). Recrystallisation from *o*-DFB/pentane afforded complex **$3^{SiMeCl2}$-Cl** as a yellow solid (15.6 mg, 19.7 μmol, 84%). ^{1}H NMR (500.1 MHz, CD_2Cl_2, 298 K): δ = 3.18–2.98 (m, 4H, P_2CH_2), 2.00 (t_{+sat}, $^{2}J_{H–P}$ = 3.1 Hz, $^{3}J_{H–Pt}$ = 31.4 Hz, 12H, PCH_3), 1.59 (t_{+sat}, $^{2}J_{H–P}$ = 3.2 Hz, $^{3}J_{H–Pt}$ = 31.3 Hz, 12H, PCH_3), 0.89 (s_{+sat}, $^{2}J_{H–Si}$ = 9.6 Hz, 3H, $SiCH_3$) ppm. ^{13}C{^{1}H} NMR (125.8 MHz, CD_2Cl_2, 298 K): δ = 44.4 (t, $^{1}J_{C–P}$ = 19.1 Hz, P_2CH_2), 20.1 (br m, PCH_3), 18.8 (s_{+sat}, $^{1}J_{C–Si}$ = 54.8 Hz, $SiCH_3$), 15.6 (br m, PCH_3) ppm. ^{29}Si{^{1}H} NMR (99.4 MHz, CD_2Cl_2, 298 K): δ = 44.6 (t_{+sat}, $^{2}J_{Si–P}$ = 14 Hz, $^{1}J_{Si–Pt}$ = 1629 Hz, $^{2}J_{Si–Pt}$ = 624 Hz, $SiCH_3$) ppm. ^{31}P{^{1}H} NMR (202.5 MHz, CD_2Cl_2, 298 K): δ = –17.7 (m_{+sat}, $^{1}J_{P–Pt}$ = 2930 Hz, $^{2}J_{P–Pt}$ = 57 Hz, 2P, P_2PtCl), –26.0 (m_{+sat}, $^{1}J_{P–Pt}$ = 2475 Hz, $^{2}J_{P–Pt}$ = 154 Hz, 2P, P_2PtSi) ppm. ^{195}Pt{^{1}H} NMR (107.5 MHz, CD_2Cl_2, 298 K): δ = –4329 (tt_{+sat}, $^{1}J_{Pt–P}$ = 2930 Hz, $^{2}J_{Pt–P}$ = 154 Hz, $^{1}J_{Pt–Pt}$ = 3738 Hz, 1Pt, P_2*Pt*Cl), –4752 (tt_{+sat}, $^{1}J_{Pt–P}$ = 2475 Hz, $^{2}J_{Pt–P}$ = 57 Hz, $^{1}J_{Pt–Pt}$ = 3738 Hz, 1Pt, P_2*Pt*Si) ppm. Elemental analysis (%) calculated for [$C_{11}H_{31}Cl_3P_4Pt_2Si\cdot(C_6H_6)_{0.5}$] ($M_w$ = 850.9): C 19.76, H 4.03, found: C 19.73, H 4.08. LIFDI-HRMS (toluene, *m/z*) calculated for $C_{11}H_{31}Cl_3P_4Pt_2Si$ = [**$3^{SiMeCl2}$-Cl**]$^+$: 811.9471; found: 811.9467. *Note: The NMR spectra of **$3^{SiMeCl2}$-Cl** show traces (<3%) of [(μ–dmpm)$_2${(Cl)PtH}{(Cl)PtSiMeCl$_2$}] (**$4^{SiMeCl2}$-HCl**, identified by comparison with **4^{SiCl3}-HCl**, which was characterised by SCXRD), presumably generated by the oxidative addition of trace HCl present in the CD_2Cl_2 solution to one of the platinum centers (δ_{31P} = –7.2 (m_{+sat}, $^{1}J_{Pt–P}$ = 930 Hz), –13.8 (m_{+sat}) ppm; δ_{195Pt} = –4600 (weak t, $^{1}J_{Pt–P}$ = 2668 Hz) and –4695 (weak m) ppm). **$4^{SiMeCl2}$-HCl** was not detected by HRMS. At 60 °C **$3^{SiMeCl2}$-Cl** partially (16%) rearranges to the silylene-bridged A-frame complex **2^{SiMeCl}-Cl** (δ_{31P} = –0.1 (m_{+sat}, $^{1}J_{Pt–P}$ = 3261 Hz, $^{3}J_{Pt–P}$ = 261 Hz), –2.8 (m_{+sat}, $^{1}J_{Pt–P}$ = 3114 Hz, $^{3}J_{Pt–P}$ = 203 Hz ppm; δ_{195Pt} = –4519 (dddd, $^{1}J_{Pt–P}$ = 3261, 3114 Hz, $^{3}J_{Pt–P}$ = 261, 203 Hz) ppm).*

Synthesis of 3^{SiCl3}-Cl

In a vial (5 mL), complex **1** (20.0 mg, 23.5 μmol) was dissolved in benzene (0.7 mL), then one equiv. of silicon tetrachloride (3.99 mg, 23.5 μmol) was added. The resulting mixture was stirred for 5 min at rt, resulting in a yellow suspension. After evaporating the volatiles at ambient pressure, the residual solid was washed with pentane (3 x 2 mL) and benzene (2 x 2 mL). Recrystallisation from benzene/pentane afforded complex **3^{SiCl3}-Cl** as a yellow solid (13.5 mg, 16.2 μmol, 69%). ^{1}H NMR (500.1 MHz, CD_2Cl_2, 298 K): δ = 3.22–3.01 (m,

4H, P_2CH_2), 2.04 (t_{+sat}, $^2J_{H-P}$ = 3.1 Hz, $^3J_{H-Pt}$ = 30.8 Hz, 12H, PCH_3), 1.61 (t_{+sat}, $^2J_{H-P}$ = 3.2 Hz, $^3J_{H-Pt}$ = 30.8 Hz, 12H, PCH_3) ppm. $^{13}C\{^1H\}$ NMR (125.8 MHz, CD_2Cl_2, 298 K): δ = 44.4 (t, $^1J_{C-P}$ = 18.7 Hz, P_2CH_2), 19.8 (br m, PCH_3), 15.3 (br m, PCH_3) ppm. $^{29}Si\{^1H\}$ NMR (99.4 MHz, CD_2Cl_2, 298 K): δ = *not detected.* $^{31}P\{^1H\}$ NMR (202.5 MHz, CD_2Cl_2, 298 K): δ = –19.4 (m_{+sat}, $^1J_{P-Pt}$ = 2815 Hz, 2P, P_2PtCl), –26.7 (m_{+sat}, $^1J_{P-Pt}$ = 2434 Hz, 2P, P_2PtSi) ppm. $^{195}Pt\{^1H\}$ NMR (107.5 MHz, CD_2Cl_2, 298 K): δ = –4305 (tt_{+sat}, $^1J_{Pt-P}$ = 2815 Hz, $^2J_{Pt-P}$ = 154 Hz, $^1J_{Pt-Pt}$ = 4163 Hz, 1Pt, P_2PtCl), –4791 (tt_{+sat}, $^1J_{Pt-P}$ = 2434 Hz, $^2J_{Pt-P}$ = 62 Hz, $^1J_{Pt-Pt}$ = 4163 Hz, 1Pt, P_2PtSi) ppm. LIFDI-HRMS (DCM, *m/z*) calculated for $C_{10}H_{28}Cl_4P_4Pt_2Si$ = [**3^{SiCl3}-Cl**]$^+$: 831.8919; found: 831.8912. *Note: The NMR spectra of **3^{SiCl3}-Cl** show traces (<5%) of [(μ–dmpm)$_2${(Cl)PtH}{(Cl)PtSiCl$_3$}] (**4^{SiCl3}-HCl**, identified by SCXRD), presumably generated by the oxidative addition of trace HCl present in the CD_2Cl_2 solution to one of the platinum centers. **4^{SiCl3}-HCl** was not detected by HRMS (δ_{31P} = –8.9 (m_{+sat}, $^1J_{Pt-P}$ = 1476 Hz), –13.7 (m_{+sat}) ppm; δ_{195Pt} = –4537 (weak t, $^1J_{Pt-P}$ = 2486 Hz), –4617 (weak m) ppm). Furthermore, **3^{SiCl3}-Cl** slowly rearranges in DCM to the silylene-bridged complex **2^{SiCl2}-Cl** (vide infra).*

Independent synthesis of 4^{SiCl3}-HCl

3^{SiCl3}-Cl (20.0 mg, 24.0 μmol, 1.00 equiv.) was dissolved in benzene (0.7 mL) and a 2 M HCl solution in Et_2O (37.4 mg, 100 μmol, 4.2 equiv.) was added. The reaction mixture was stirred at rt for 5 min, resulting in a yellow suspension. After slow evaporation of the solvent at rt, the yellow residue was washed with pentane (3 x 2 mL). Recrystallisation by diffusion of pentane into a saturated DCM solution at rt yielded **4^{SiCl3}-HCl** as a pale-yellow crystalline solid (13.4 mg, 15.4 μmol, 59%). ^1H NMR (500.1 MHz, CD_2Cl_2, 298 K): δ = 3.20–2.66 (m, 4H, P_2CH_2), 1.93 (app. t_{+sat}, $^2J_{H-P}$ = 3.8 Hz, $^3J_{H-Pt}$ = 26.2 Hz, 12H, PCH_3), 1.73 (app. t_{+sat}, $^2J_{H-P}$ = 3.5 Hz, $^3J_{H-Pt}$ = 33.5 Hz, 12H, PCH_3), –15.8 (t_{+sat}, $^2J_{H-P}$ = 13.2 Hz, $^1J_{H-Pt}$ = 1193 Hz, $^3J_{H-Pt}$ = 22.8 Hz, 1H, Pt*H*) ppm. $^{13}C\{^1H\}$ NMR (125.8 MHz, CD_2Cl_2, 298 K): δ = 35.1 (tt, $^1J_{C-P}$ = 17.7 Hz, $^3J_{C-P}$ = 13.7 Hz, P_2CH_2), 17.5 (br s, PCH_3), 14.8 (t, $^1J_{C-P}$ = 18.6 Hz, PCH_3) ppm. $^{29}Si\{^1H\}$ NMR (99.4 MHz, CD_2Cl_2, 298 K): δ = –16.4 (t_{+sat}, $^2J_{Si-P}$ = 13.3 Hz, $^1J_{Si-Pt}$ = 3138 Hz, $PtSiCl_3$) ppm. $^{31}P\{^1H\}$ NMR (202.5 MHz, CD_2Cl_2, 298 K): δ = –8.9 (m_{+sat}, $^1J_{P-Pt}$ = 2490 Hz, $^3J_{P-Pt}$ = 73.5 Hz, 2P, P_2PtH), –13.6 (m_{+sat}, $^1J_{P-Pt}$ = 2750 Hz, 2P, $P_2PtSiCl_3$) ppm. $^{195}Pt\{^1H\}$ NMR (107.5 MHz, CD_2Cl_2, 298 K): δ = –4537 (t, $^1J_{P-Pt} \approx$ 2490 Hz, Pt, *Pt*H), –4624 (t, $^1J_{P-Pt} \approx$ 2768 Hz, Pt, $PtSiCl_3$) ppm. LIFDI-HRMS (DCM, *m/z*) calculated for $C_{10}H_{28}Cl_5P_4Pt_2Si$ = [**4^{SiCl3}-HCl** – H]$^+$: 866.8603; found: 866.8602.

Synthesis of 2^{SiCl2}-Cl

In a vial (5 mL), complex **6** (20.0 mg, 24.0 µmol) was dissolved in DCM (0.7 mL) and the mixture heated at 60 °C for 4 d. After evaporating the volatiles at ambient pressure, the residual solid was washed with pentane (3 x 2 mL) and benzene (2 x 2 mL). Recrystallisation from DCM/pentane afforded complex **2^{SiCl2}-Cl** as a yellow solid (14.0 mg, 16.8 µmol, 70%). ^{1}H NMR (500.1 MHz, CD_2Cl_2, 298 K): δ = 3.24–3.14 (m, 2H, P_2CH_2), 1.91–1.85 (m, 2H, P_2CH_2), 1.79 (t_{+sat}, $^{2}J_{H–P}$ = 3.7 Hz, $^{3}J_{H–Pt}$ = 26.4 Hz, 12H, PCH_3), 1.71 (t_{+sat}, $^{2}J_{H–P}$ = 3.2 Hz, $^{3}J_{H–Pt}$ = 28.5 Hz, 12H, PCH_3) ppm. ^{13}C{^{1}H} NMR (125.8 MHz, CD_2Cl_2, 298 K): δ = 27.2 (tt_{+sat}, $^{1}J_{C–P}$ = 16.5 Hz, $^{2}J_{C–Pt}$ = 99.8 Hz, P_2CH_2), 16.2 (br m, PCH_3), 14.6 (br m, PCH_3) ppm. ^{29}Si{^{1}H} NMR (99.4 MHz, CD_2Cl_2, 298 K): δ = 22.5 (t_{+sat}, $^{2}J_{Si–P}$ = 10.2 Hz, $^{1}J_{Si–Pt}$ = 1559 Hz, $SiCl_2$) ppm. ^{31}P{^{1}H} NMR (202.5 MHz, CD_2Cl_2, 298 K): δ = 1.2 (s_{+sat}, $^{1}J_{P–Pt}$ = 3098 Hz, $^{3}J_{P–Pt}$ = 242 Hz, $^{2}J_{Pt–Pt}$ = 283 Hz) ppm. ^{195}Pt{^{1}H} NMR (107.5 MHz, CD_2Cl_2, 298 K): δ = –4446 (tt, $^{1}J_{Pt–P}$ = 3098 Hz, $^{3}J_{Pt–P}$ = 242 Hz) ppm. Elemental analysis (%) calculated for [$C_{10}H_{28}Cl_4P_4Pt_2Si\cdot(C_6H_6)_{0.1}$] ($M_w$ = 840.1): C 15.15, H 3.43, found: C 15.22, H 3.45.

Synthesis of 5^{SiCl3}-Br and 5^{SiCl3}-Cl

In a vial (5 mL), complex **3^{SiCl3}-Cl** (20.0 mg, 24.0 µmol) was dissolved in DBM (0.7 mL) and the solution heated at 60 °C for 7 d. After evaporating the volatiles at ambient pressure, the resulting residue was washed with pentane (3 x 2 mL) and benzene (2 x 2 mL). Recrystallisation of the residue from DCM/pentane yielded an inseparable 80:20 mixture of complexes **5^{SiCl3}-Br** and **5^{SiCl3}-Cl** as a yellow solid (15.3 mg, 14.6 µmol, 61% combined yield). **5^{SiCl3}-Br** (80%): ^{1}H NMR (500.1 MHz, C_6D_6, 298 K): δ = 3.53–3.41 (m, 2H, P_2CH_2), 1.74 (t_{+sat}, $^{2}J_{H–P}$ = 8.5 Hz, 2H, $PtCH_2$), 1.60 (t_{+sat}, $^{2}J_{H–P}$ = 3.3 Hz, $^{3}J_{H–Pt}$ = 25.4 Hz, 6H, PCH_3), 1.39 (t_{+sat}, $^{2}J_{H–P}$ = 3.5 Hz, $^{3}J_{H–Pt}$ = 21.0 Hz, 6H, PCH_3), 1.24 (t_{+sat}, $^{2}J_{H–P}$ = 3.7 Hz, $^{3}J_{H–Pt}$ = 20.2 Hz, 6H, PCH_3), 1.21–1.15 (m, 2H, P_2CH_2), 1.10 (t_{+sat}, $^{2}J_{H–P}$ = 3.2 Hz, $^{3}J_{H–Pt}$ = 25.4 Hz, 6H, PCH_3) ppm. ^{13}C{^{1}H} NMR (125.8 MHz, C_6D_6, 298 K): δ = 34.5 (t_{+sat}, $^{1}J_{C–P}$ = 14.5 Hz, P_2CH_2), 15.0 (app. tt, $^{1}J_{C–P}$ = 18.8 Hz, $^{3}J_{C–P}$ = 4.5 Hz, PCH_3), 14.2 (app. tt, $^{1}J_{C–P}$ = 19.7 Hz, $^{3}J_{C–P}$ = 4.2 Hz, PCH_3), 12.9 (app. t, $^{1}J_{C–P}$ = 18.7 Hz, PCH_3), 10.2 (app. t, $^{1}J_{C–P}$ = 17.0 Hz, PCH_3), –2.9 (t, $^{2}J_{C–P}$ = 5.6 Hz, $PtCH_2$) ppm. ^{29}Si{^{1}H} NMR (99.4 MHz, C_6D_6, 298 K): δ = 2.5 (s, $SiCl_3$) ppm. ^{31}P{^{1}H} NMR (202.5 MHz, C_6D_6, 298 K): δ = –15.0 (t_{+sat}, $^{2}J_{P-P}$ = 11.8 Hz, $^{1}J_{P–Pt}$ = 2702 Hz, 2P, $P_2Pt(Br)SiCl_3$), –20.7 (t_{+sat}, $^{2}J_{P-P}$ = 11.8 Hz, $^{1}J_{P–Pt}$ = 2420 Hz, 2P, P_2PtBr_2) ppm. ^{195}Pt{^{1}H} NMR (107.5 MHz, C_6D_6, 298 K): δ = –4408 (t, $^{1}J_{Pt–P}$ = 2420 Hz, Pt, P_2PtBr_2), –4421 (t, $^{1}J_{Pt–P}$ = 2702 Hz, Pt, $P_2Pt(Br)SiCl_3$), ppm. **5^{SiCl3}-Cl** (20%): ^{1}H NMR (500.1 MHz, C_6D_6, 298 K): δ = 2.35 (t_{+sat}, $^{2}J_{H–P}$ = 8.5 Hz, 2H, $PtCH_2$), 1.70 (t, $^{2}J_{H–P}$ = 3.4 Hz, 6H, PCH_3), 1.62 (m, 6H,

PCH_3), 1.53 (t, $^2J_{H–P}$ = 3.1 Hz, 6H, PCH_3), 1.45 (m, 6H, PCH_3) ppm. *Note: The resonance for the P_2CH_2 group was not detected.* ^{13}C{^{1}H} NMR (125.8 MHz, C_6D_6, 298 K): δ = 14.8 (m, PCH_3), 14.7 (m, PCH_3), 13.2 (m, PCH_3), 12.1 (m, PCH_3), –1.2 (m, PtCH_2) ppm. *Note: The P_2CH_2 resonance was not detected.* ^{29}Si{^{1}H} NMR (99.4 MHz, C_6D_6, 298 K): δ = 2.5 (s, *Si*Cl_3) ppm. ^{31}P{^{1}H} NMR (202.5 MHz, C_6D_6, 298 K): δ = –9.6 (t_{+sat}, $^2J_{P\text{-}P}$ = 15.2 Hz, $^1J_{Pt–P}$ = 2720 Hz, 2P, P_2Pt(Cl)CH_2SiCl_3), –16.7 (t_{+sat}, $^2J_{P\text{-}P}$ = 15.2 Hz, 2P, P_2PtCl_2) ppm. ^{195}Pt{^{1}H} NMR (107.5 MHz, C_6D_6, 298 K): δ = –4367 (t, $^1J_{Pt–P}$ ≈ 2420 Hz, Pt, P_2*Pt*Cl_2), –4411 (t, $^1J_{Pt–P}$ ≈ 2770 Hz, Pt, P_2*Pt*(Cl)$SiCl_3$) ppm. *Note: the ^{195}Pt NMR resonances and $^1J_{Pt–P}$ coupling constants of **5^{SiCl3}-Cl** were identified by ^{1}H-^{195}Pt HMBC.* LIFDI-HRMS (DCM, *m/z*) calculated for $C_{11}H_{30}Br_2Cl_3P_4Pt_2Si$ = [**5^{SiCl3}-Br** – Br]$^+$: 970.7739; found: 970.7723; for $C_{11}H_{30}BrCl_4P_4Pt_2Si$ = [**5^{SiCl3}-Cl** – Cl]$^+$: 924.8253; found: 924.8246.

NMR spectra of new compounds

General remarks

As the complexes decomposed when exposed to a vacuum, they had to be dried at ambient pressure. As a result, most NMR spectra exhibit resonances of residual crystallisation solvents, including benzene, *o*-DFB, pentane, DCM or hexanes, marked with *. The ubiquitous decomposition products [(μ–dmpm)$_2$Pt$_2$X$_2$] are marked with ♦. Other, unidentified byproducts are marked with **?**

Analysis of ^{29}Si{^{1}H} NMR spectra

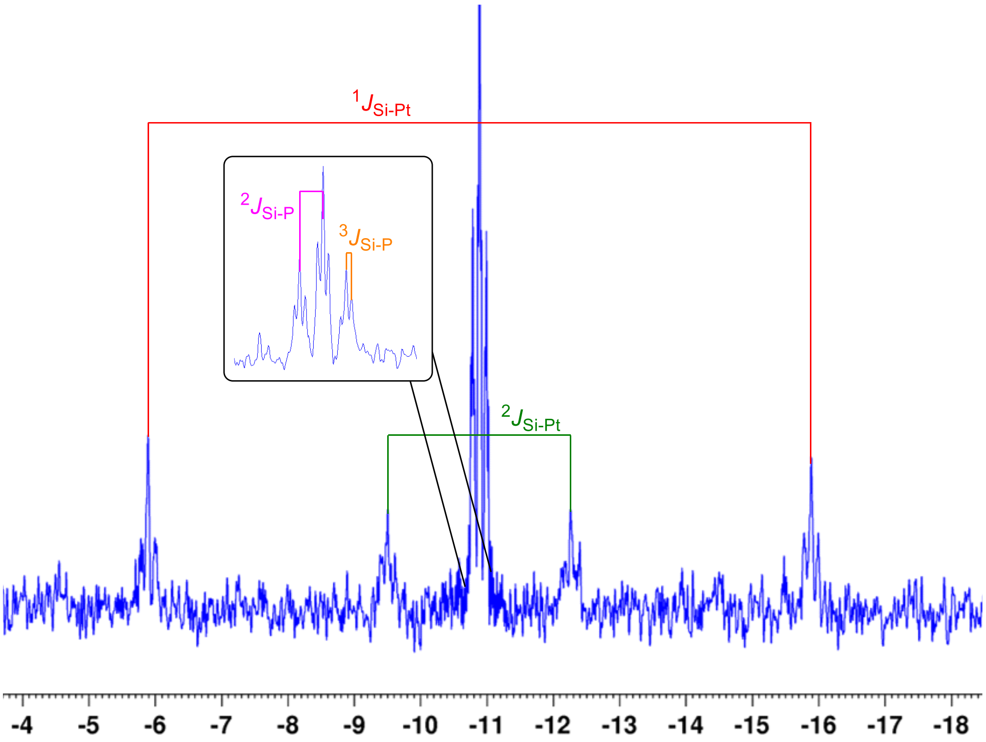

Figure S1. Typical ^{29}Si{^{1}H} spectrum of an unsymmetrical [(μ-dmpm)$_2${PtX}{PtSiR$_3$}] complex, showing the main couplings.

Analysis of $^{31}P\{^1H\}$ NMR spectra[4]

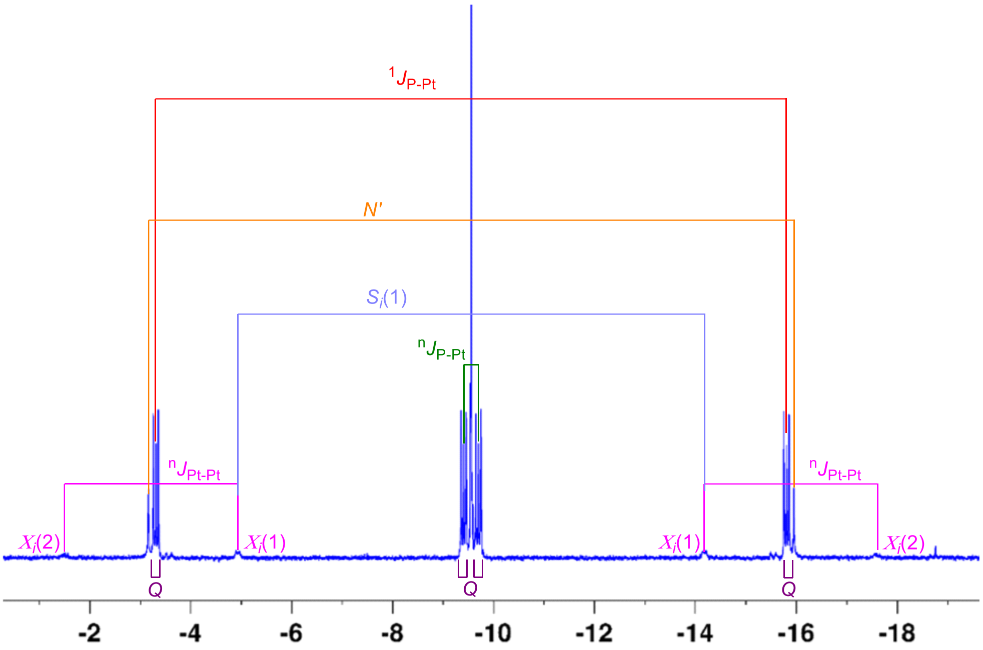

Figure S2. Typical $^{31}P\{^1H\}$ spectrum of a symmetrical A-frame complex, showing the main couplings. $N' = {}^1J_{P\text{-}Pt} + {}^nJ_{P\text{-}Pt}$. $Q = \Sigma({}^nJ_{P\text{-}P})$ (*note:* $^nJ_{P\text{-}P}$ *may be negative*). Second way of calculating $^nJ_{Pt\text{-}Pt}$, when the $X_i(2)$ lines are not visible: $^nJ_{Pt\text{-}Pt} = (L'^2 - S_i(1)^2)/(2S_i(1))$, where $L' = {}^1J_{P\text{-}Pt} - {}^nJ_{P\text{-}Pt}$.

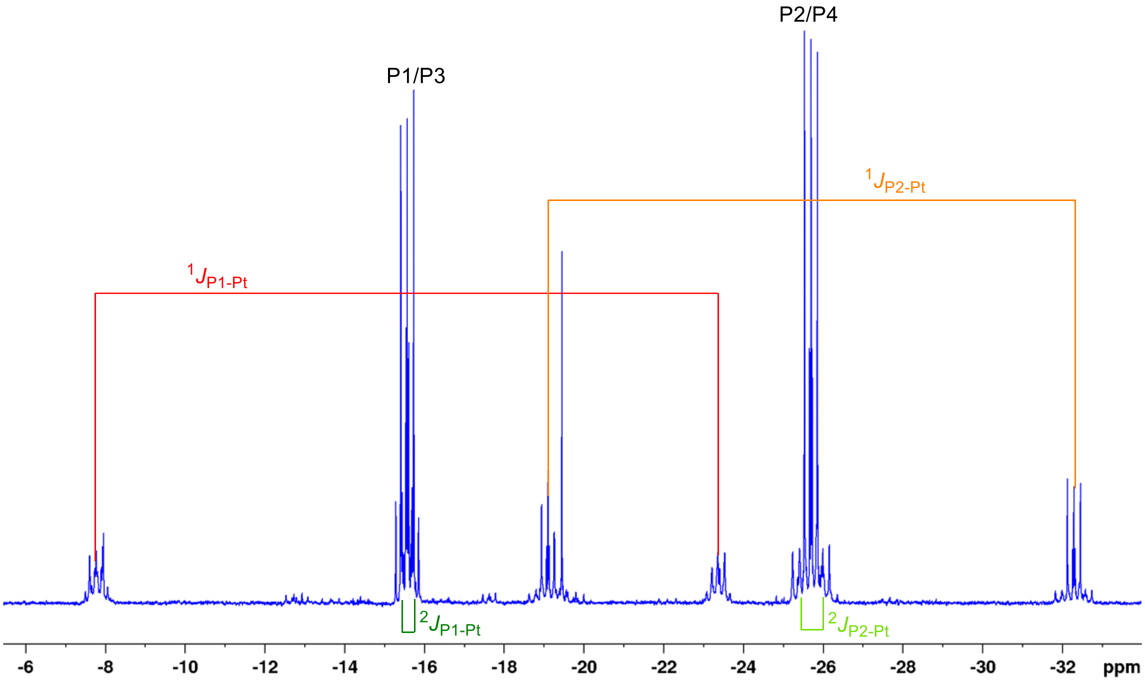

Figure S3. Typical $^{31}P\{^1H\}$ spectrum of an unsymmetrical (μ-dmpm)$_2$Pt$_2$ complex, showing the main couplings. *Note: despite the close proximity of the two resonances, no higher-order coupling patterns are observed.*

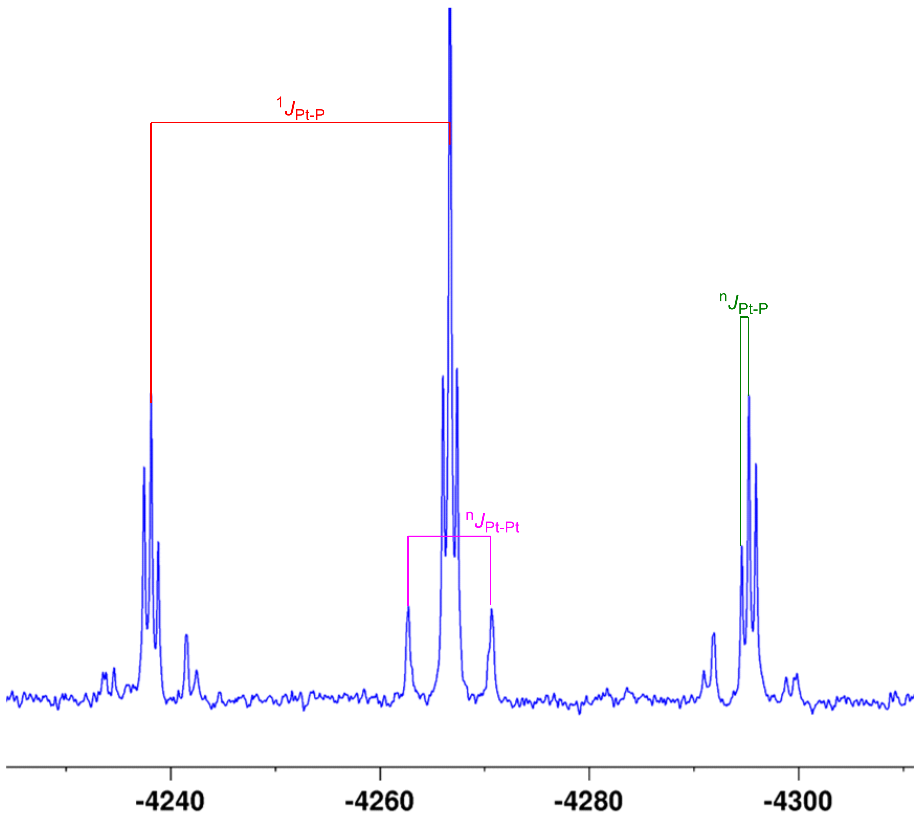

Figure S4. Typical $^{195}Pt\{^1H\}$ spectrum of an A-frame complex, showing the main couplings.

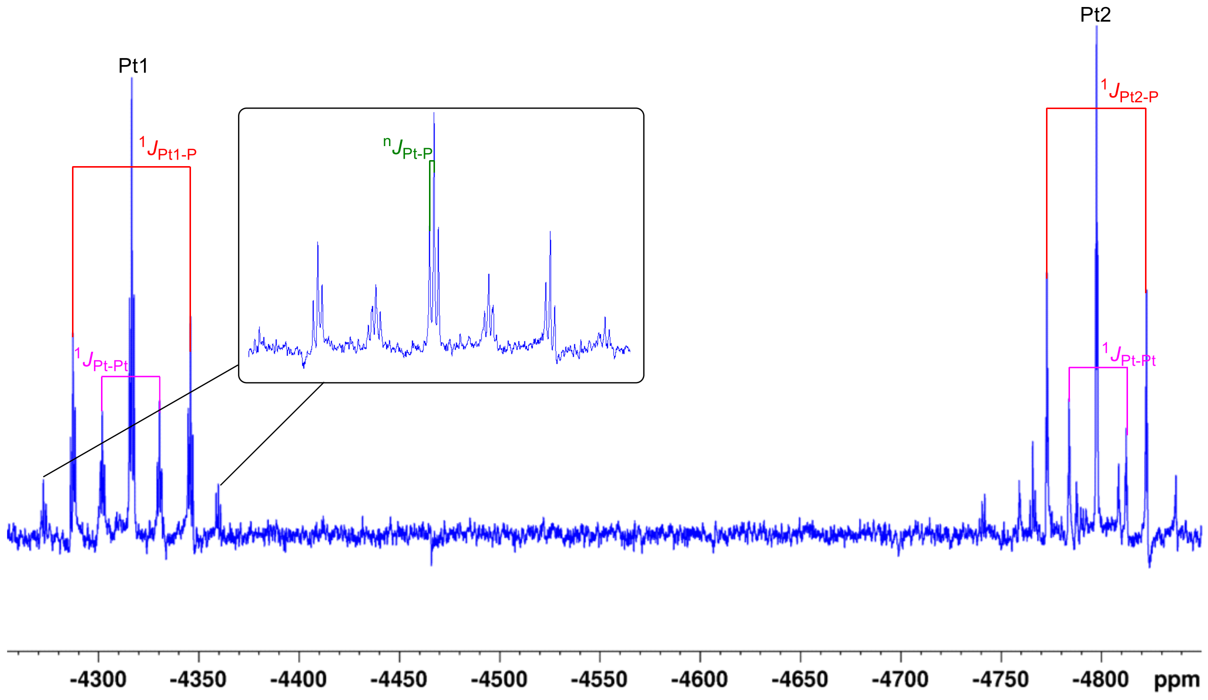

Figure S5. Typical $^{195}Pt\{^1H\}$ spectrum of an unsymmetrical (μ-dmpm)$_2$Pt$_2$ complex, showing the main couplings.

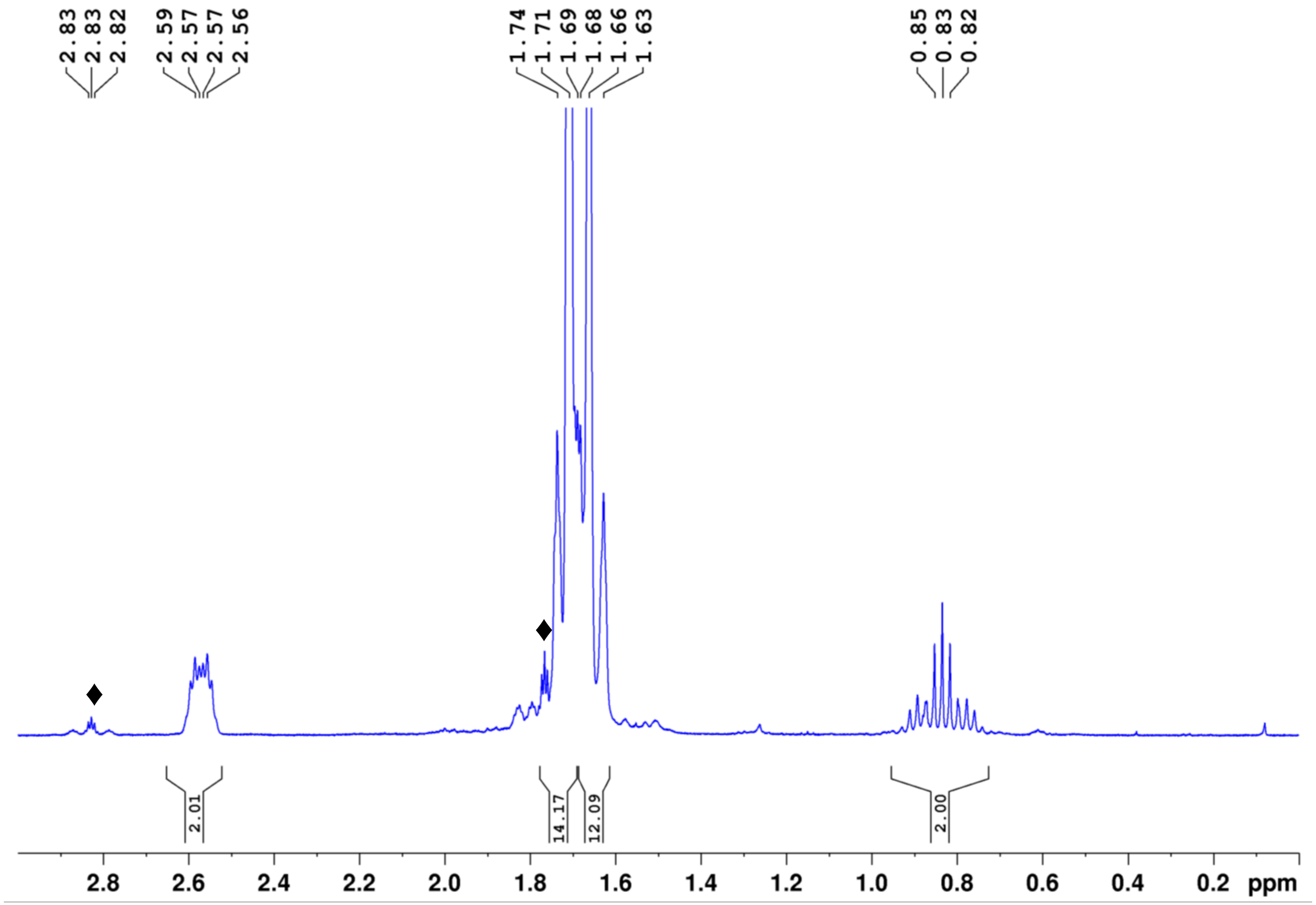

Figure S6. ^{1}H NMR spectrum of **2^{CH2}-Cl** in CD_2Cl_2. The additional resonances marked ♦ correspond to the decomposition product [(μ–dmpm)$_2$Pt$_2$Cl$_2$].

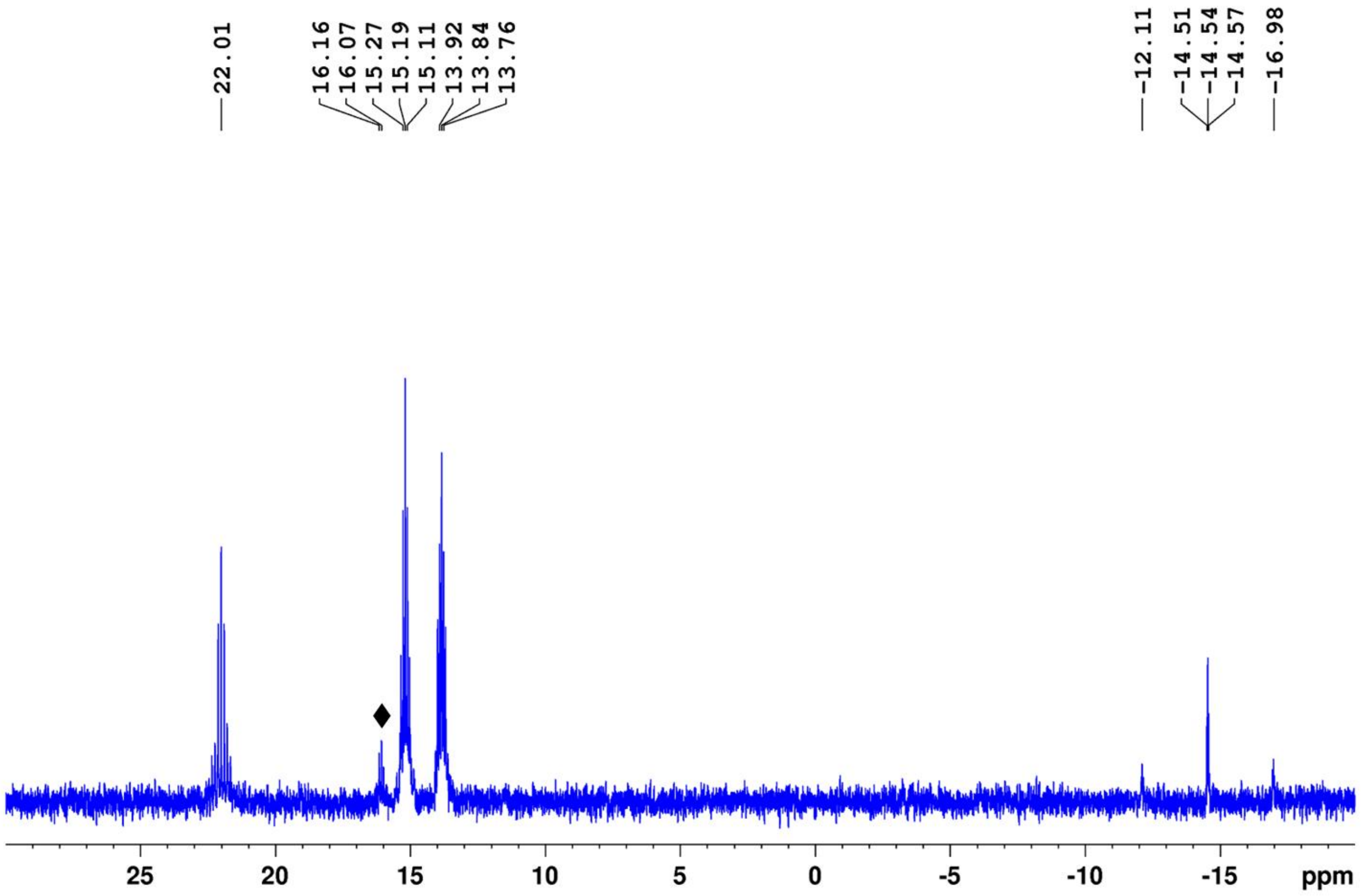

Figure S7. $^{13}C\{^{1}H\}$ NMR spectrum of **2^CH2^-Cl** in CD_2Cl_2. The additional resonance marked ♦ corresponds to the decomposition product [(μ–dmpm)$_2$Pt$_2$Cl$_2$].

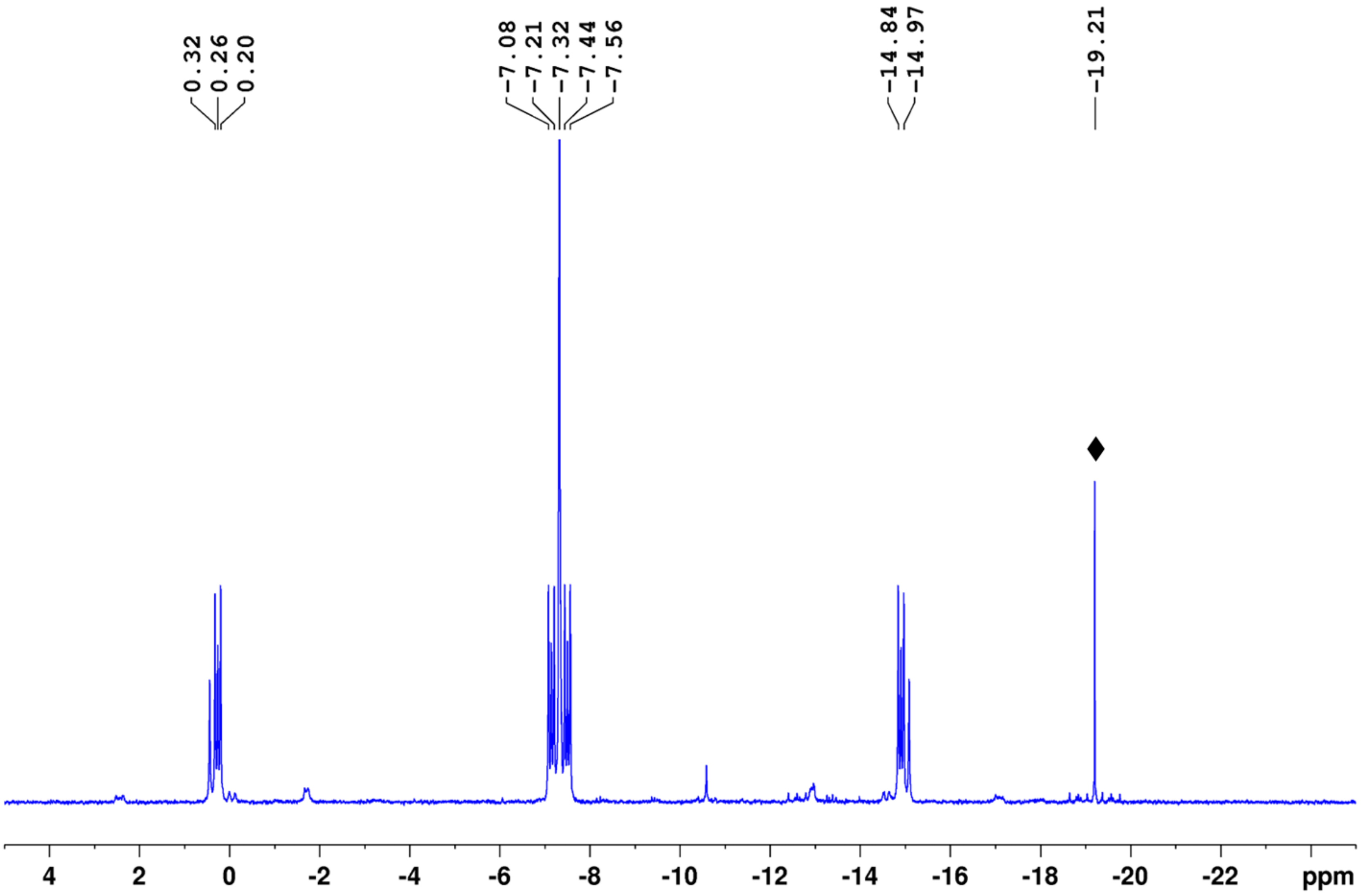

Figure S8. $^{31}P\{^{1}H\}$ NMR spectrum of **2^{CH2}-Cl** in CD_2Cl_2. The additional resonances marked ♦ correspond to the decomposition product [(μ–dmpm)$_2$Pt$_2$Cl$_2$] (ca. 5%).

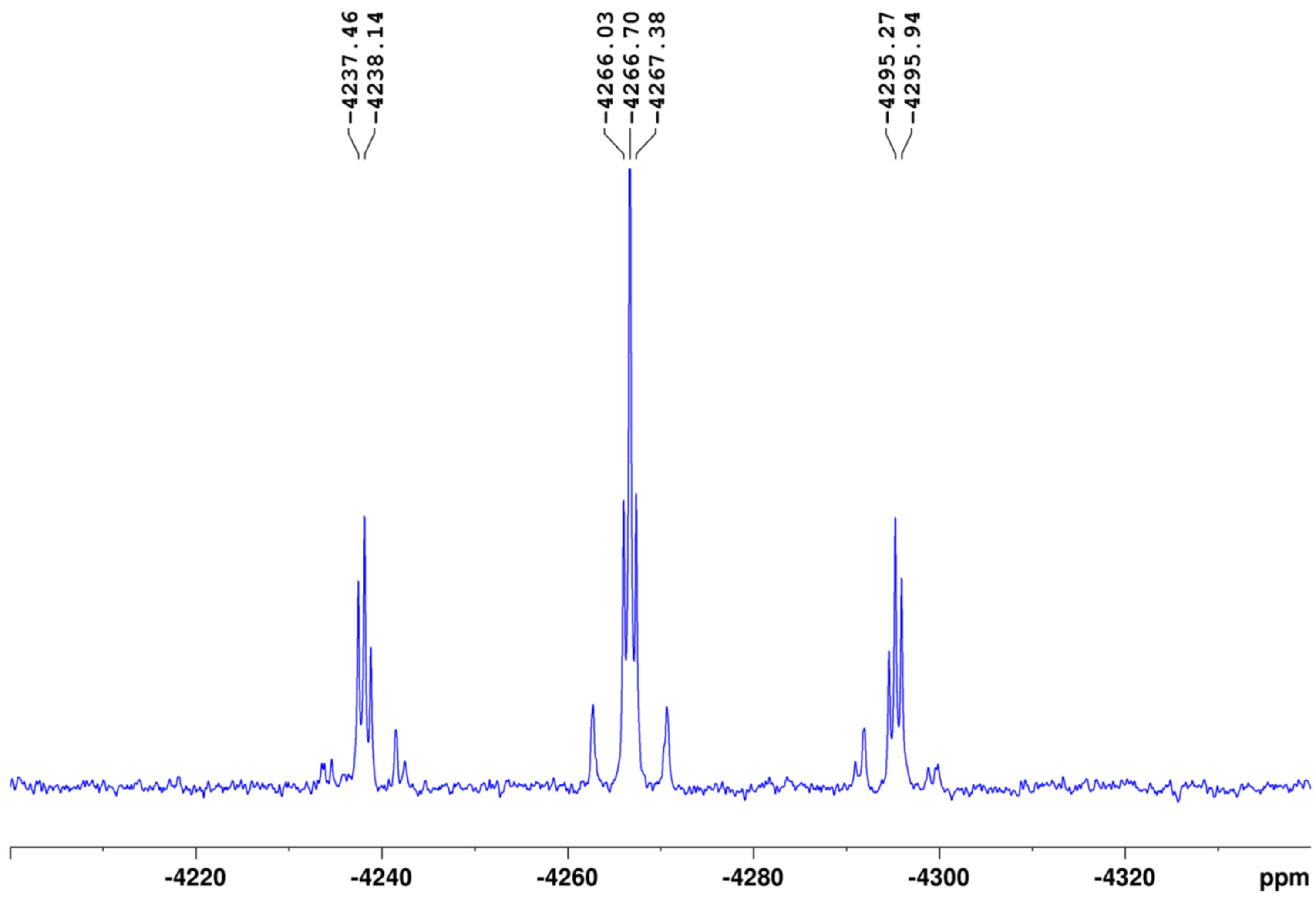

Figure S9. $^{195}Pt\{^{1}H\}$ NMR spectrum of $\mathbf{2^{CH2}}$**-Cl** in CD_2Cl_2.

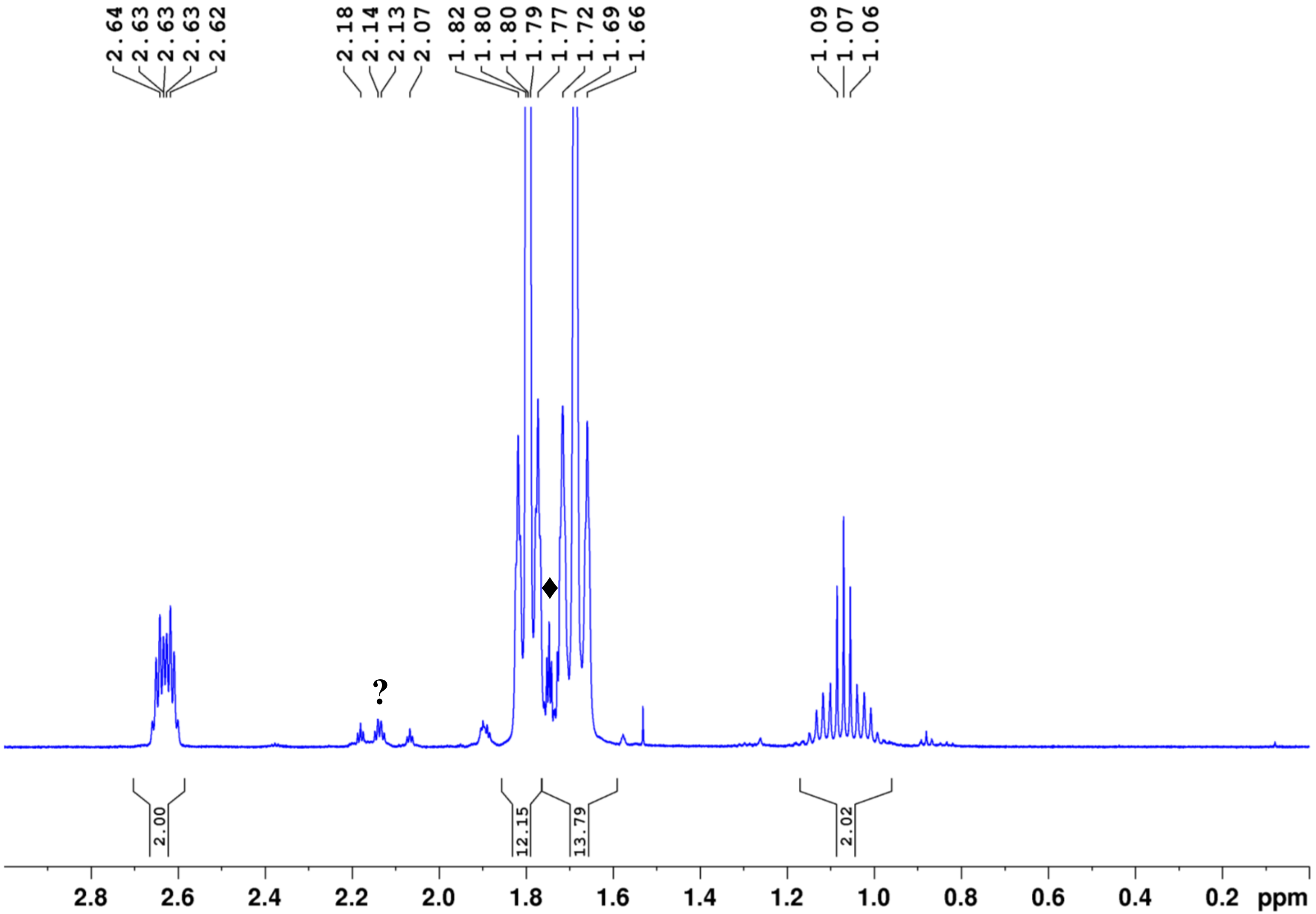

Figure S10. ^{1}H NMR spectrum of $\mathbf{2^{CH2}}$**-Br** in CD_2Cl_2. The additional resonances marked ♦ correspond to the decomposition product $[(\mu\text{–dmpm})_2Pt_2Cl_2]$, those marked **?** to an unknown byproduct.

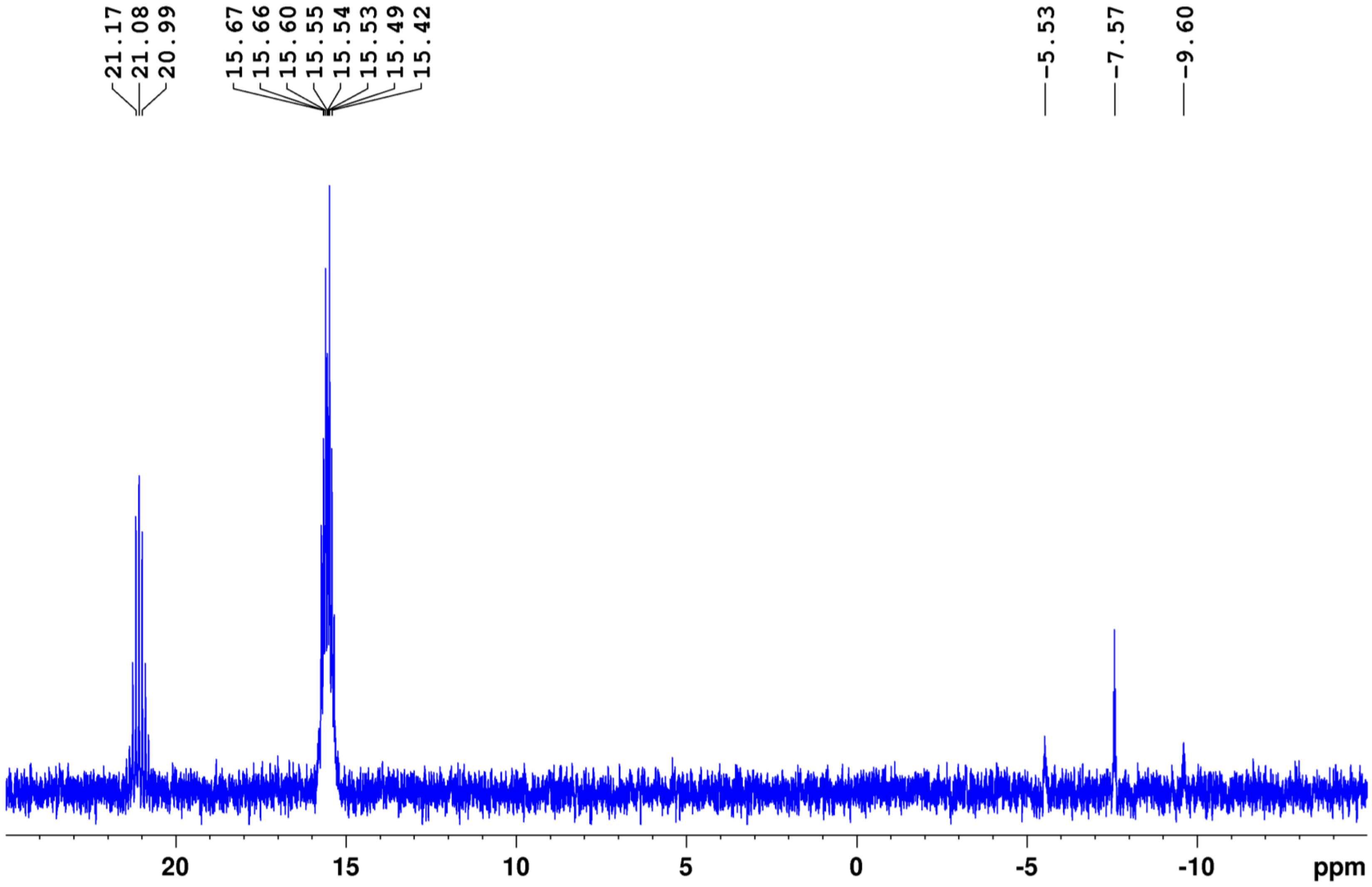

Figure S11. $^{13}C\{^{1}H\}$ NMR spectrum of $\mathbf{2^{CH2}}$**-Br** in CD_2Cl_2.

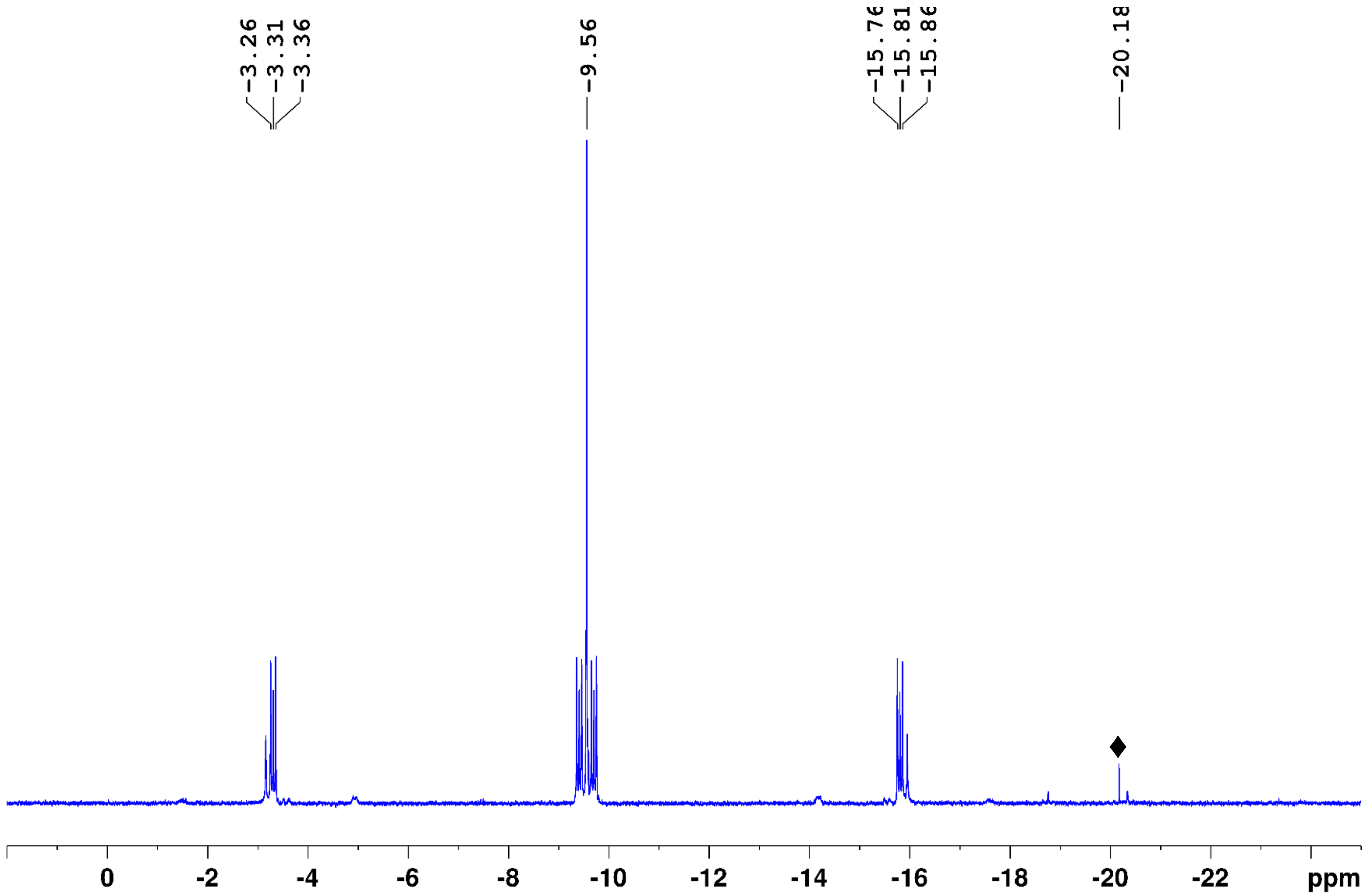

Figure S12. $^{31}P\{^{1}H\}$ NMR spectrum of **2^{CH2}-Br** in CD_2Cl_2. The additional resonances marked ♦ correspond to the decomposition product [(*μ*–dmpm)$_2$Pt$_2$Br$_2$] (ca. 0.5%).

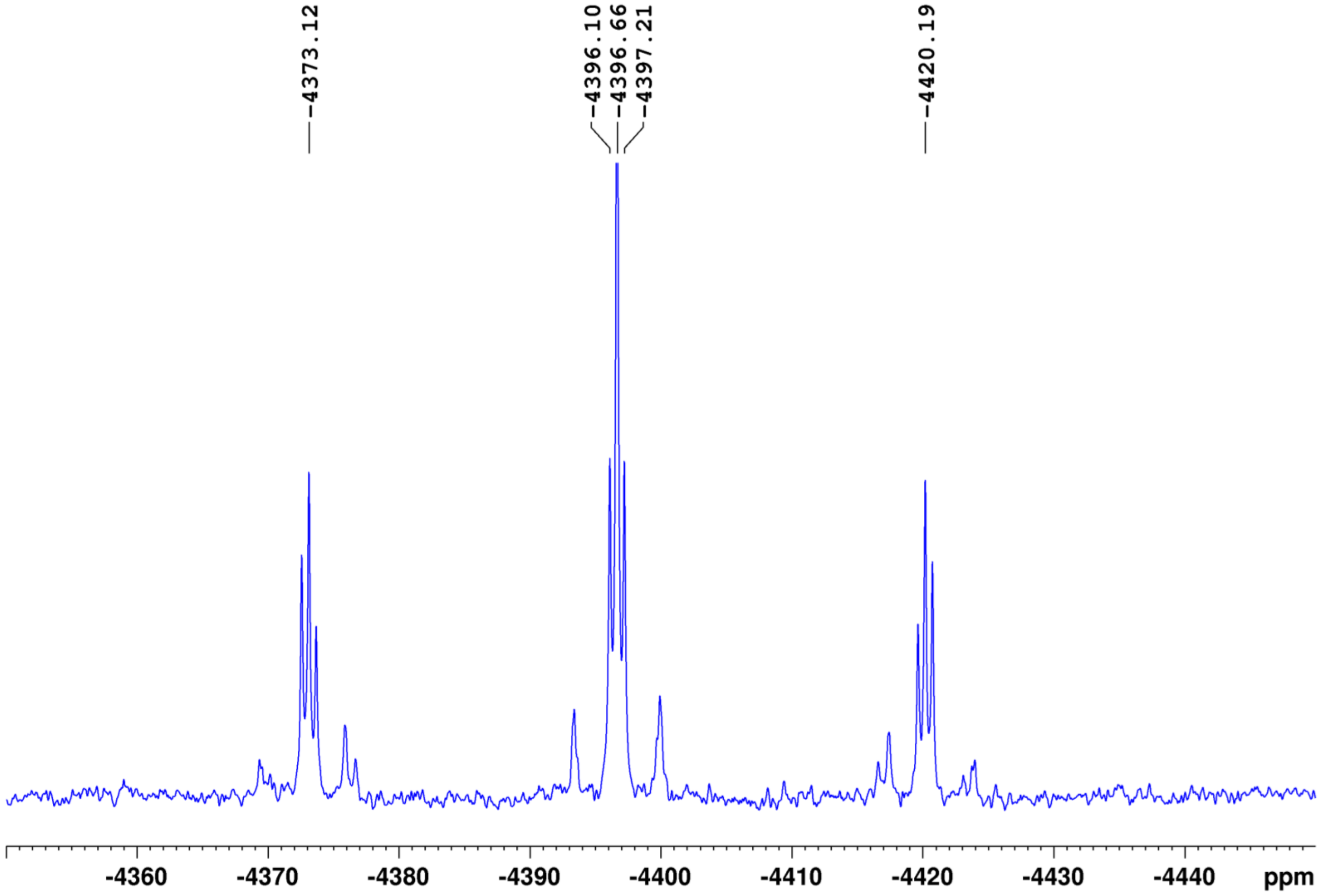

Figure S13. $^{195}Pt\{^{1}H\}$ NMR spectrum of $\mathbf{2^{CH2}}$**-Br** in CD_2Cl_2.

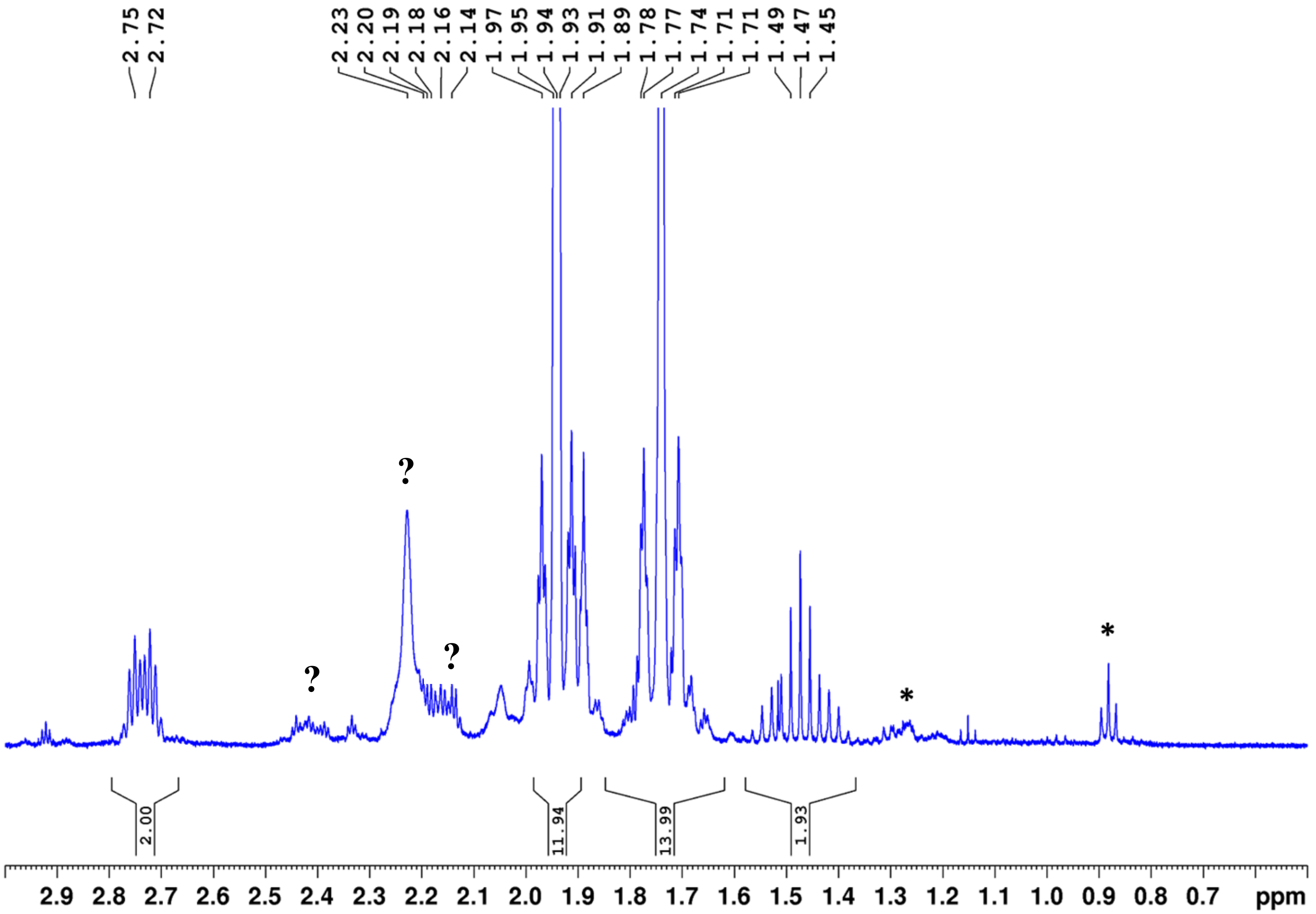

Figure S14. ^{1}H NMR spectrum of $\mathbf{2^{CH2}}$**-I** in CD_2Cl_2. The additional resonances marked **?** correspond to an unknown Pt complex, those marked * to residual pentane.

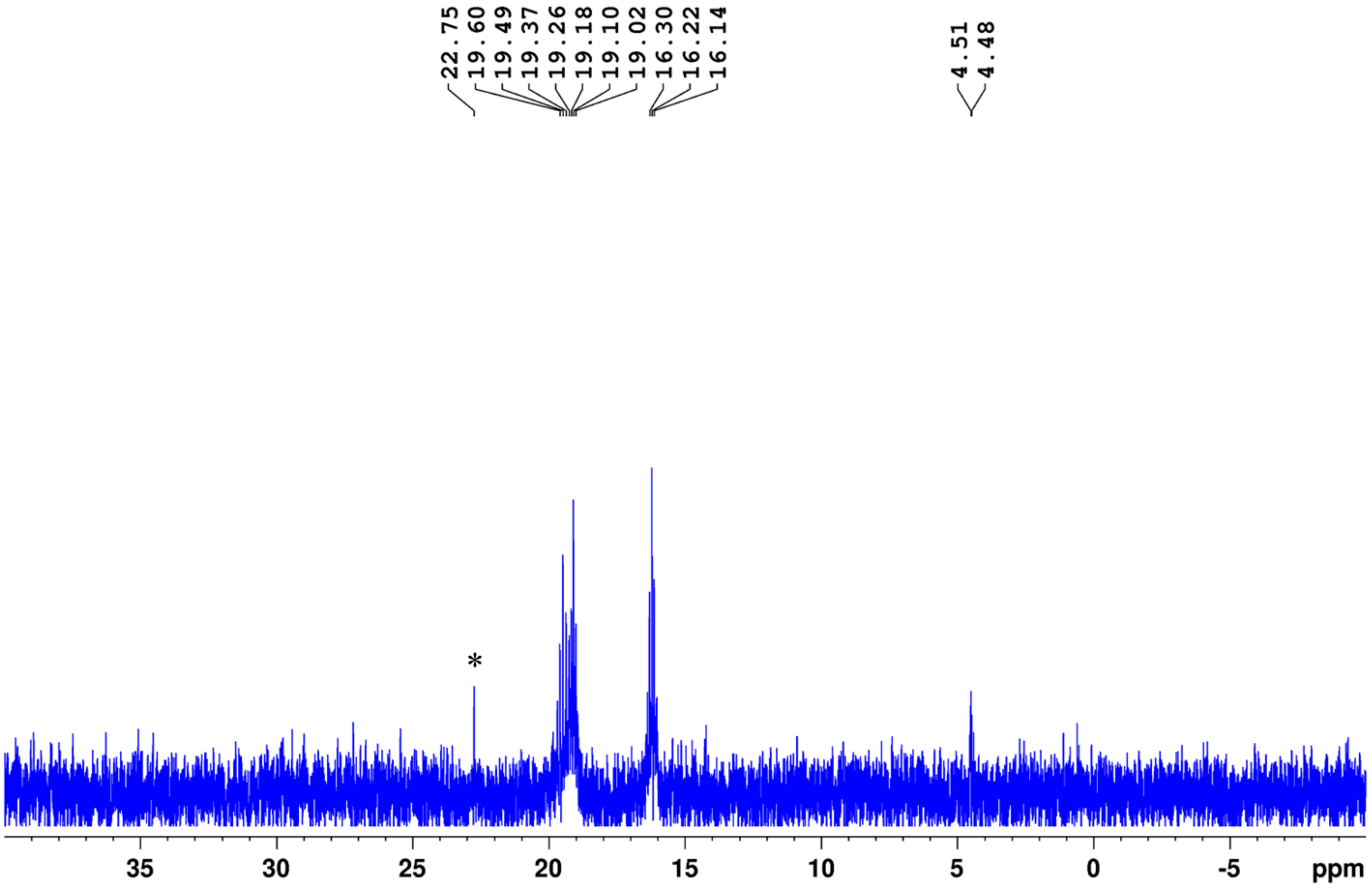

Figure S15. $^{13}C\{^1H\}$ NMR spectrum of $\mathbf{2^{CH2}}$**-I** in CD_2Cl_2. The additional resonance marked * corresponds to residual pentane.

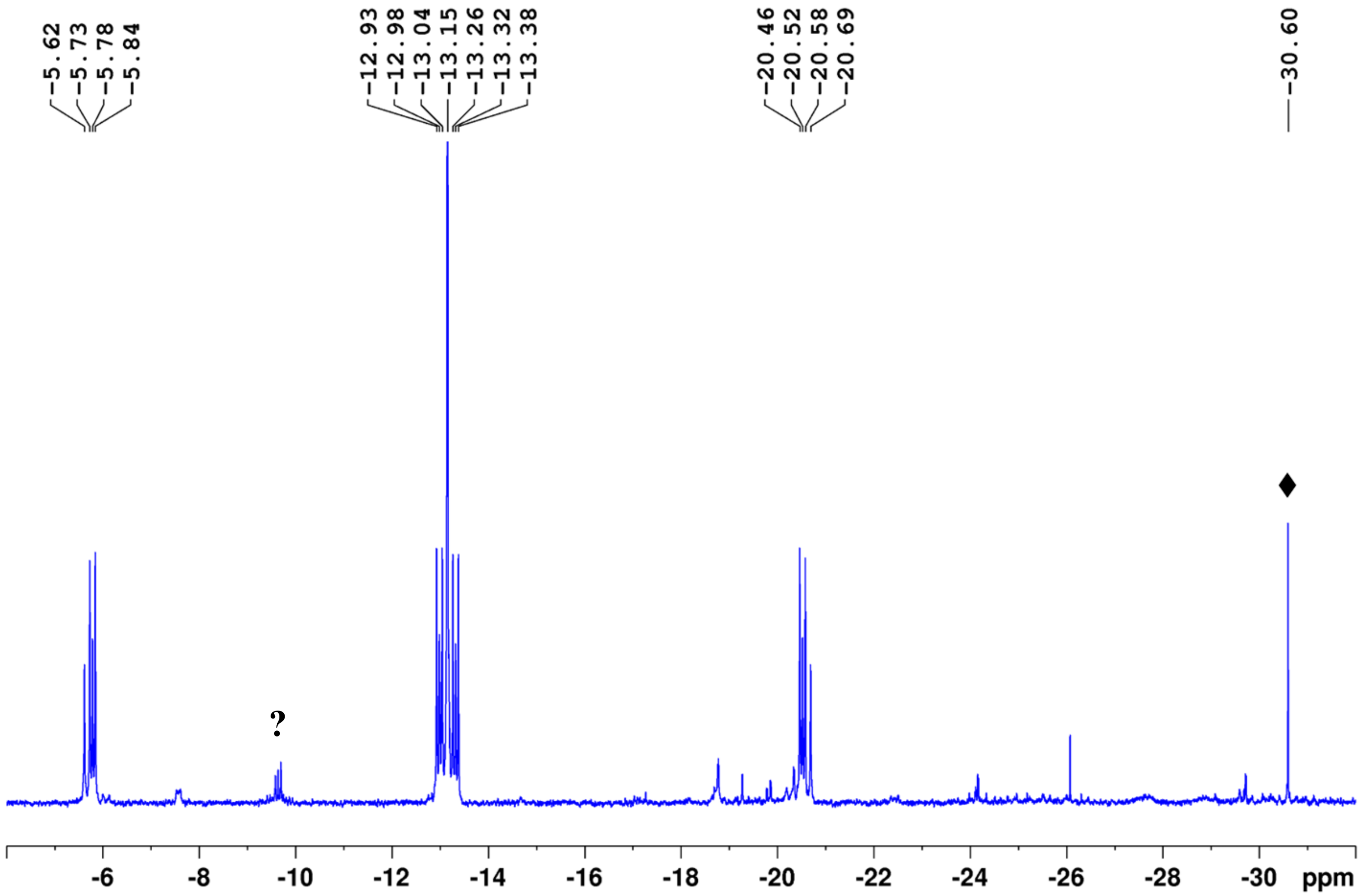

Figure S16. $^{31}P\{^1H\}$ NMR spectrum of $\mathbf{2^{CH2}}$**-I** in CD_2Cl_2. The additional resonance marked ♦ corresponds to the decomposition product [(*μ*–dmpm)$_2$Pt$_2$I$_2$] (ca. 3%), that marked **?** to an unknown byproduct (ca. 3%).

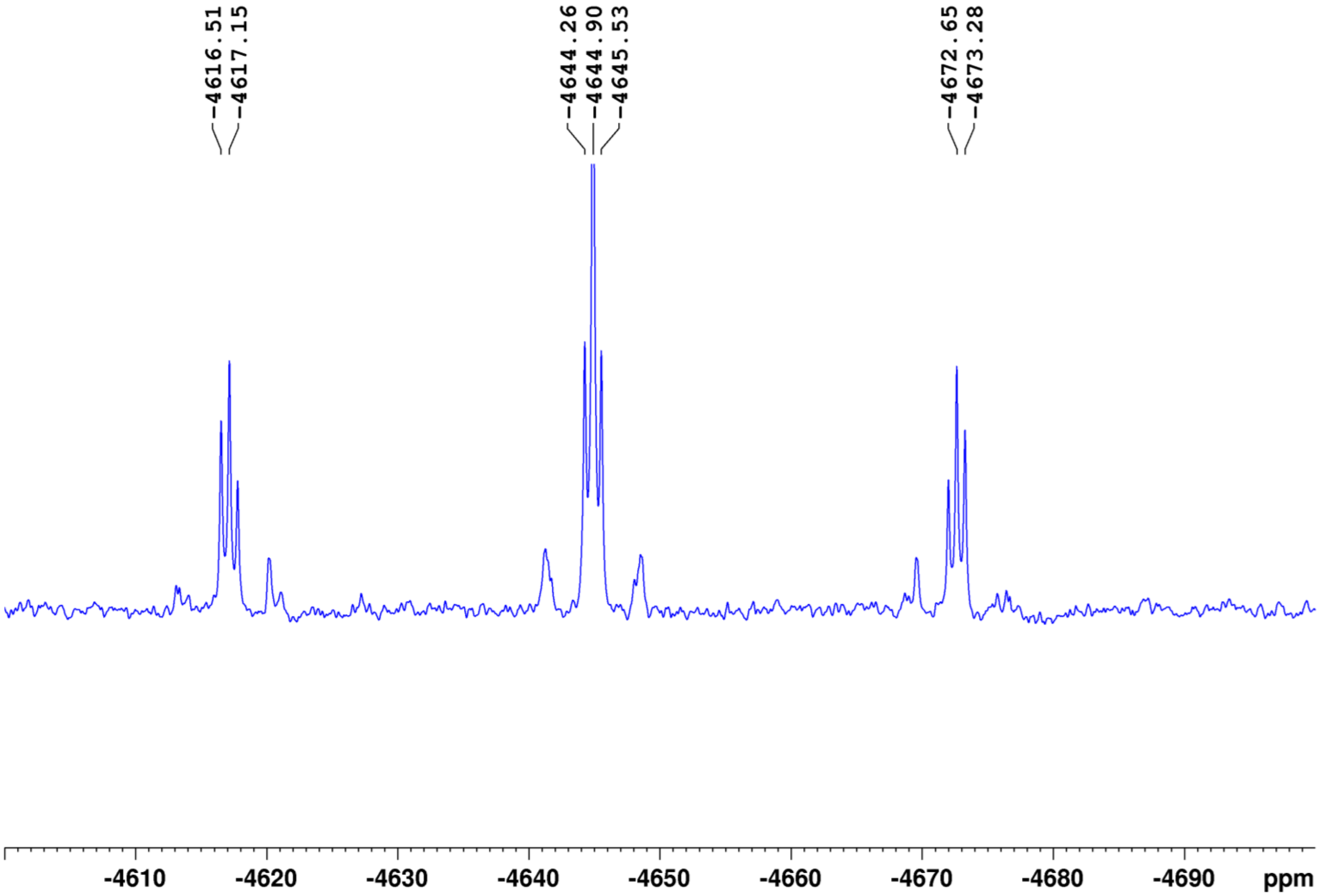

Figure S17. $^{195}Pt\{^{1}H\}$ NMR spectrum of $\mathbf{2^{CH2}}$**-I** in CD_2Cl_2.

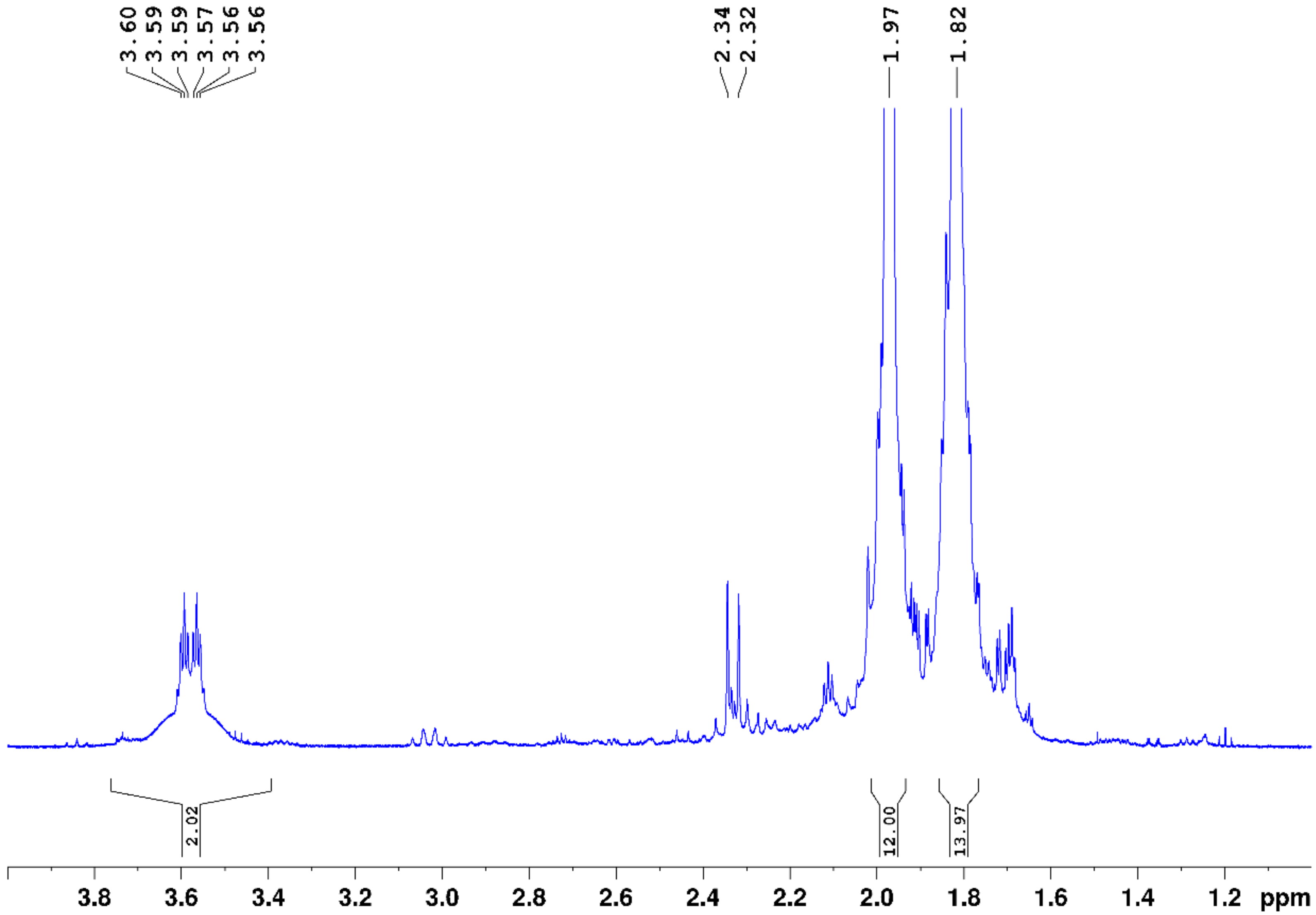

Figure S18. ^{1}H NMR spectrum of **2^{CCl2}-Cl** in $CDCl_3$, showing small amounts of decomposition (ca. 5%) after 30 min at rt.

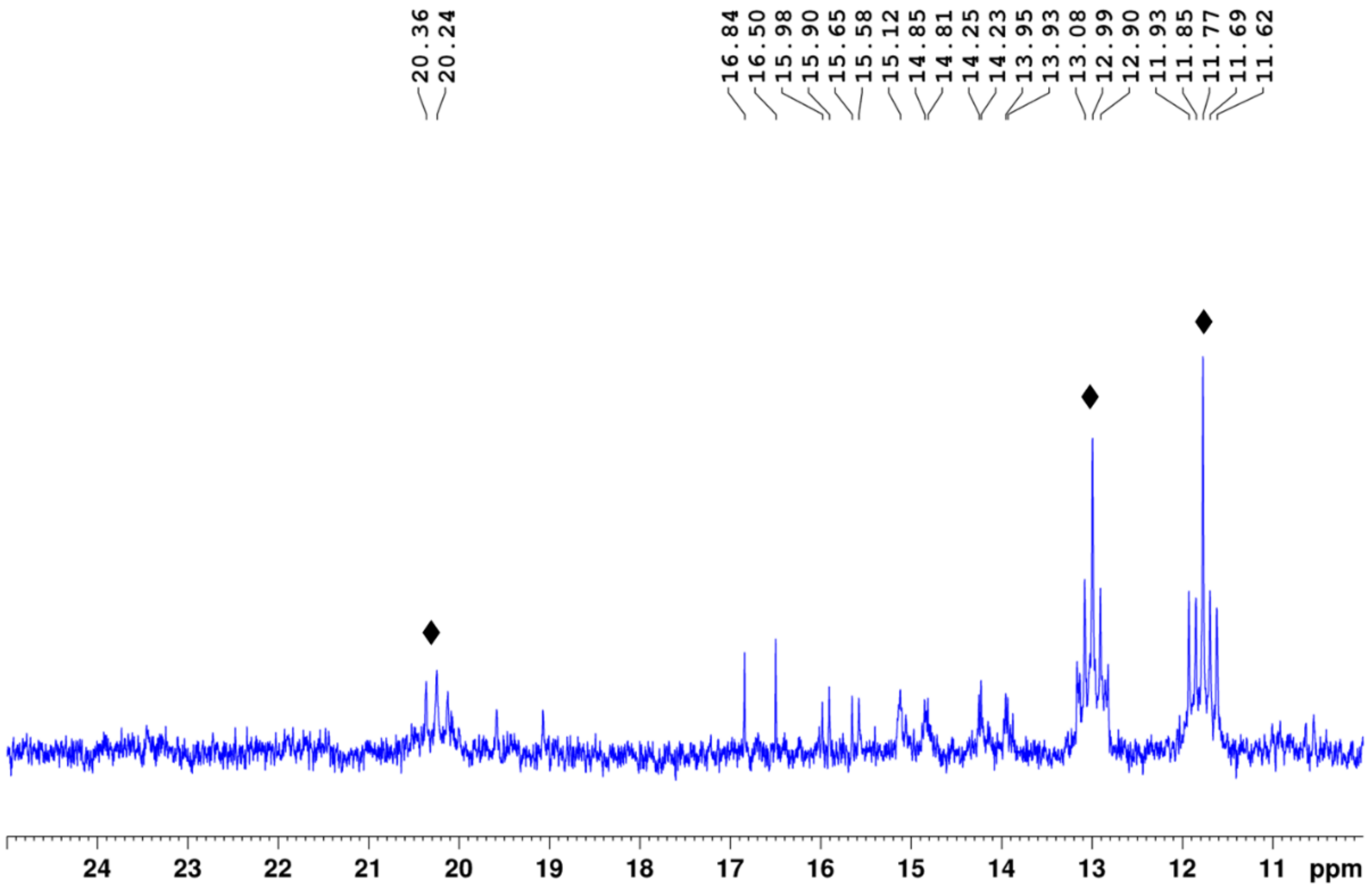

Figure S19. ^{13}C {^{1}H} NMR spectrum of **2^{CCl2}-Cl** (♦) in $CDCl_3$ after 5 h at rt, already showing significant amounts of decomposition.

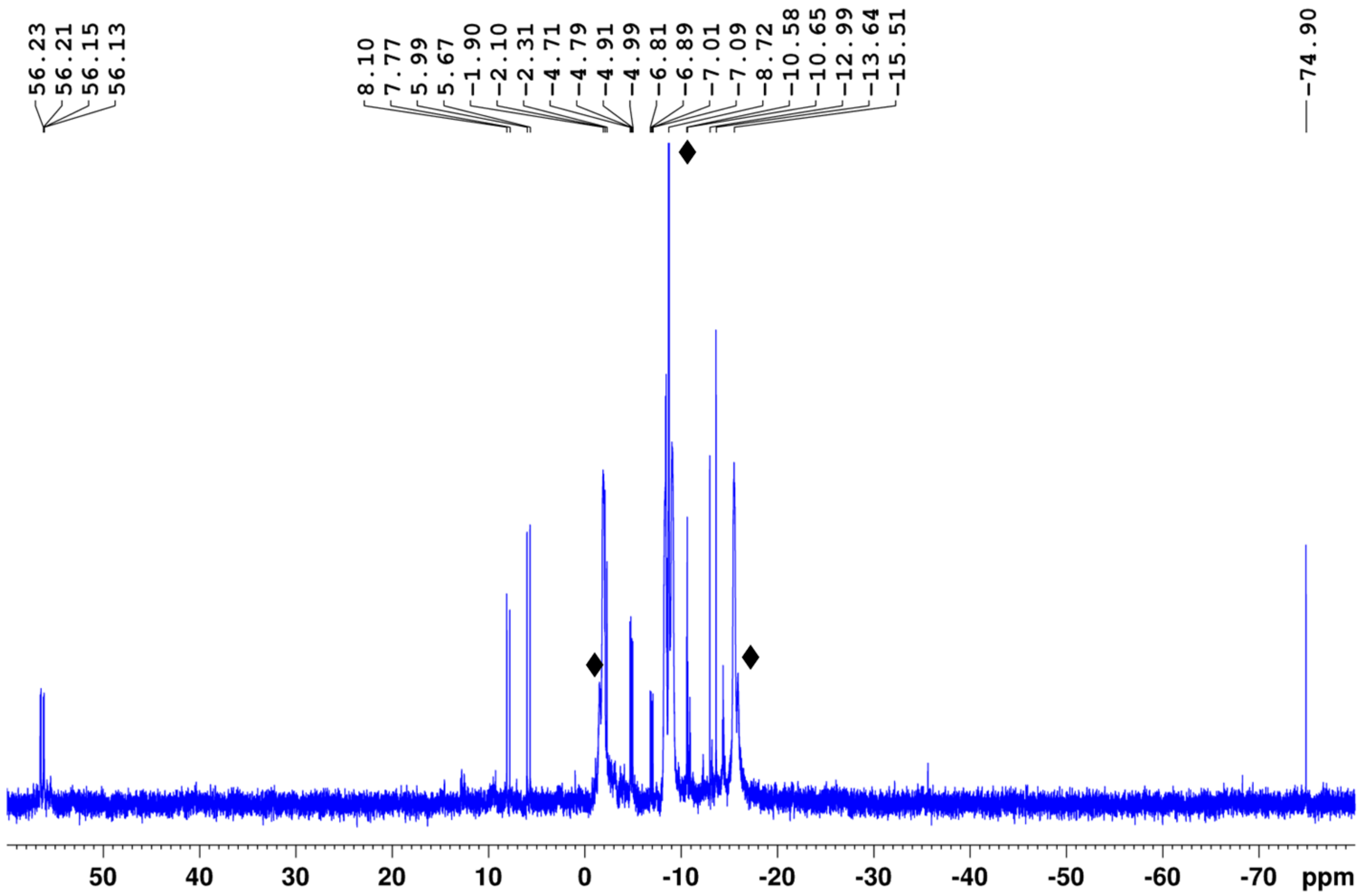

Figure S20. $^{31}P\{^1H\}$ NMR spectrum of $\mathbf{2^{CCl2}}$**-Cl** (♦) in $CDCl_3$ after 1 h at rt, already showing ca. 13% of decomposition

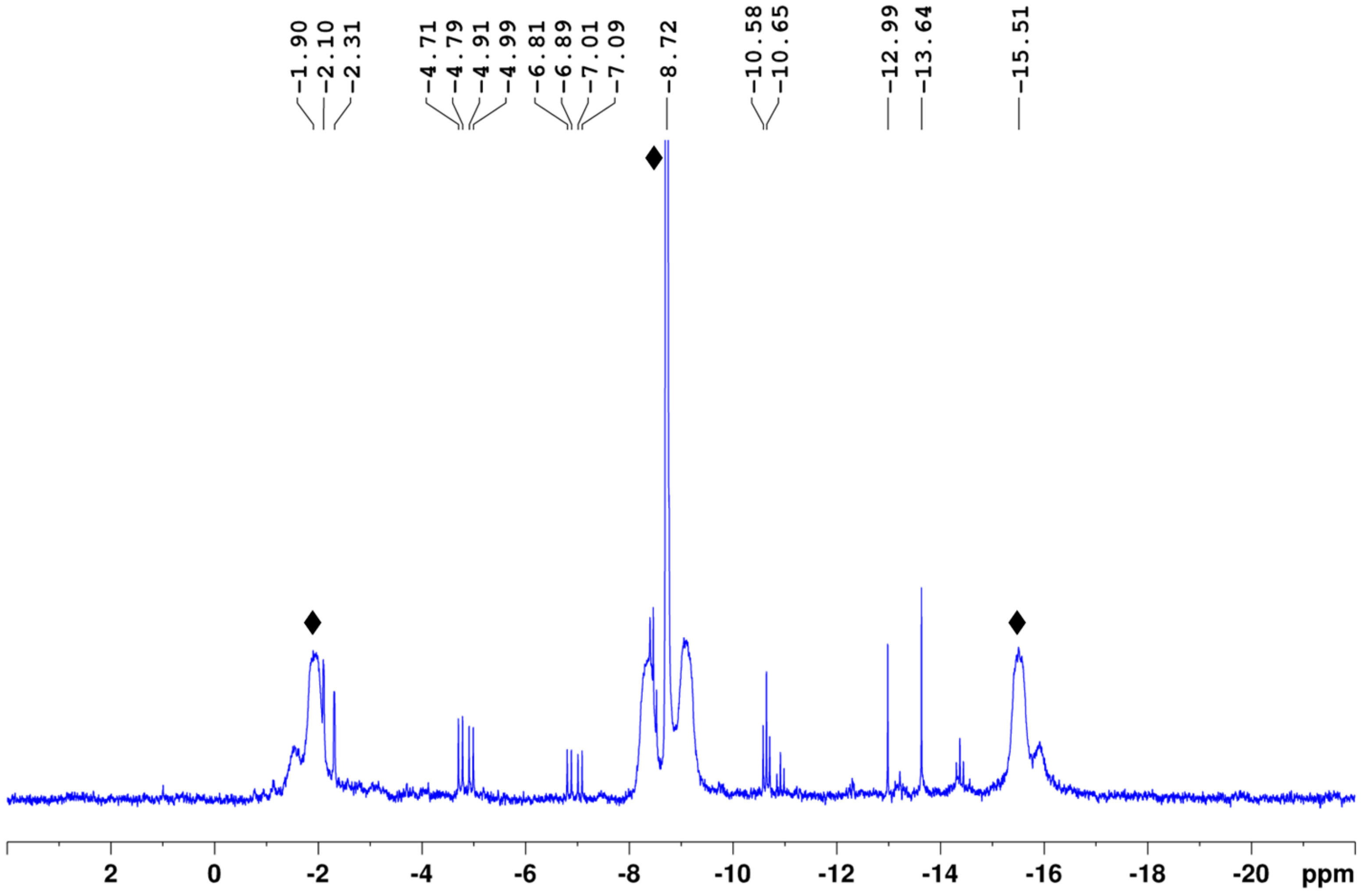

Figure S21. Zoom-in on the $^{31}P\{^{1}H\}$ NMR resonance of **2^{CCl2}-Cl** (♦) in $CDCl_3$ after 1 h at rt.

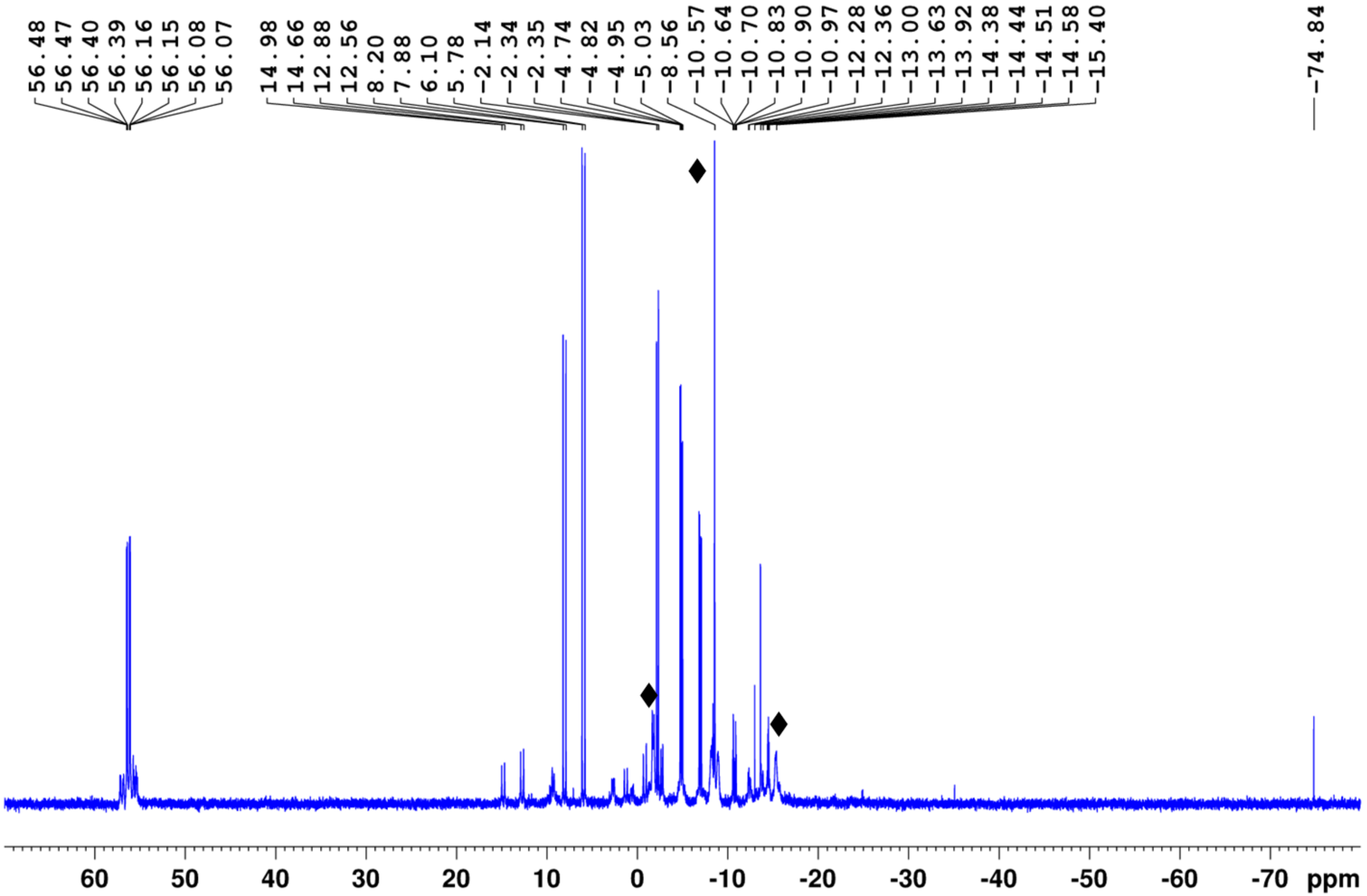

Figure S22. $^{31}P\{^{1}H\}$ NMR spectrum of **2^{CCl2}-Cl** (♦) after 16 h at rt in $CDCl_3$, showing ca. 68% decomposition to unidentified products.

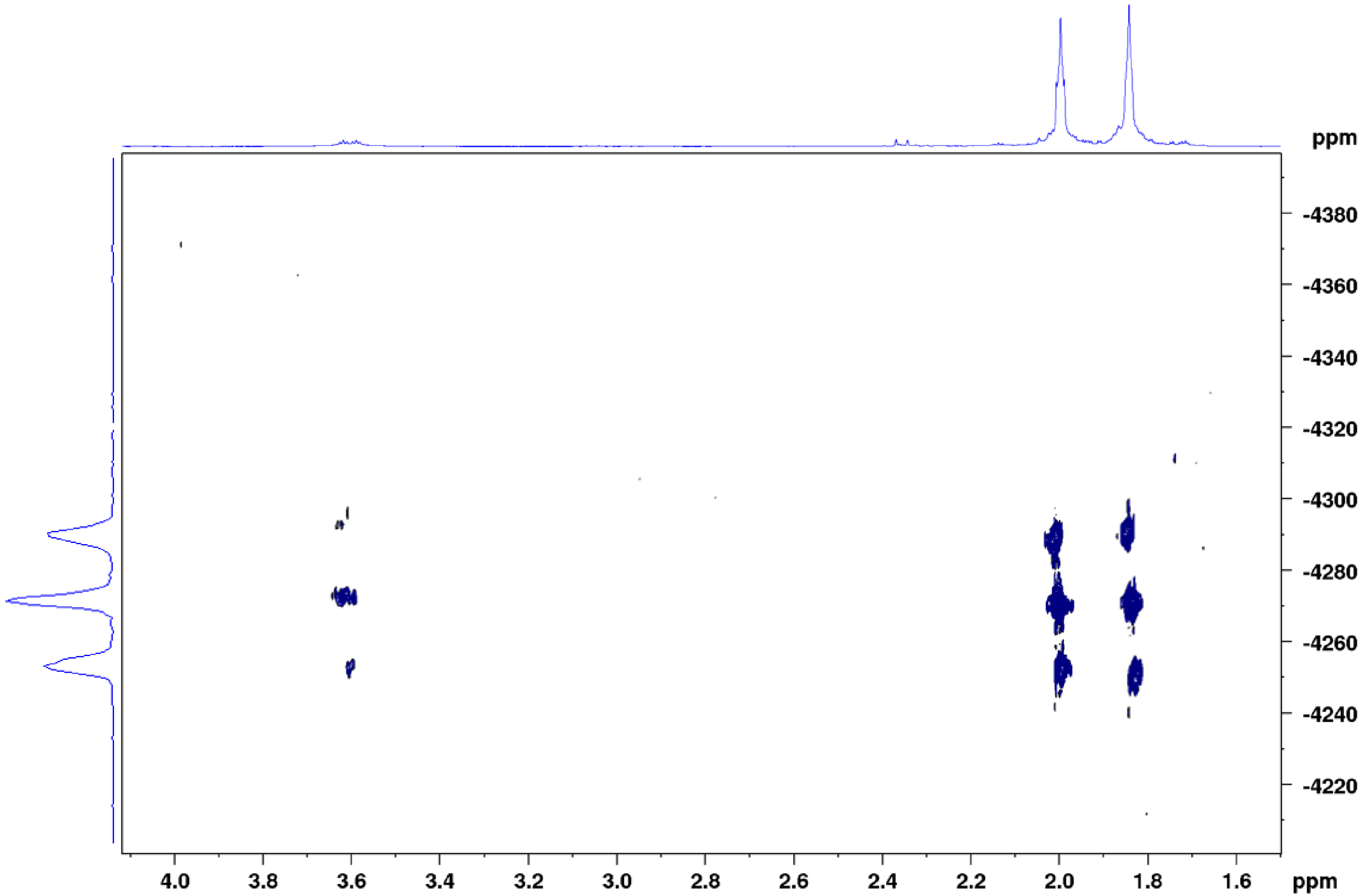

Figure S23. Zoom-in on the ^{1}H-$^{195}Pt\{^{1}H\}$ HMQC spectrum of $\mathbf{2^{CCl2}}$**-Cl** in $CDCl_3$ after 2 h at rt.

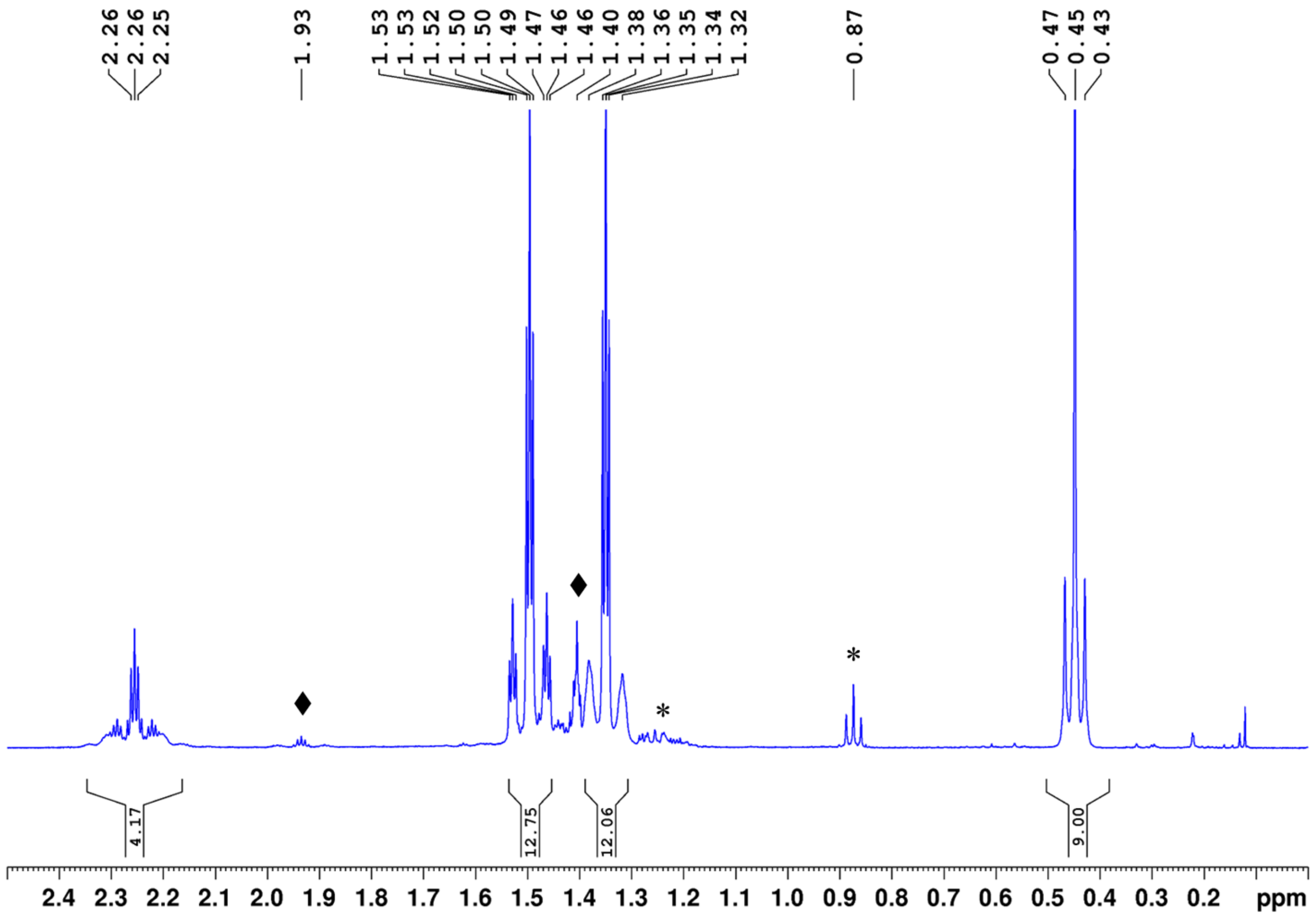

Figure S24. ^{1}H NMR spectrum of **3^{SiMe3}-Cl** in C_6D_6. The additional resonances marked ♦ correspond to the decomposition product [(μ–dmpm)$_2$Pt$_2$Cl$_2$], those marked * to residual pentane.

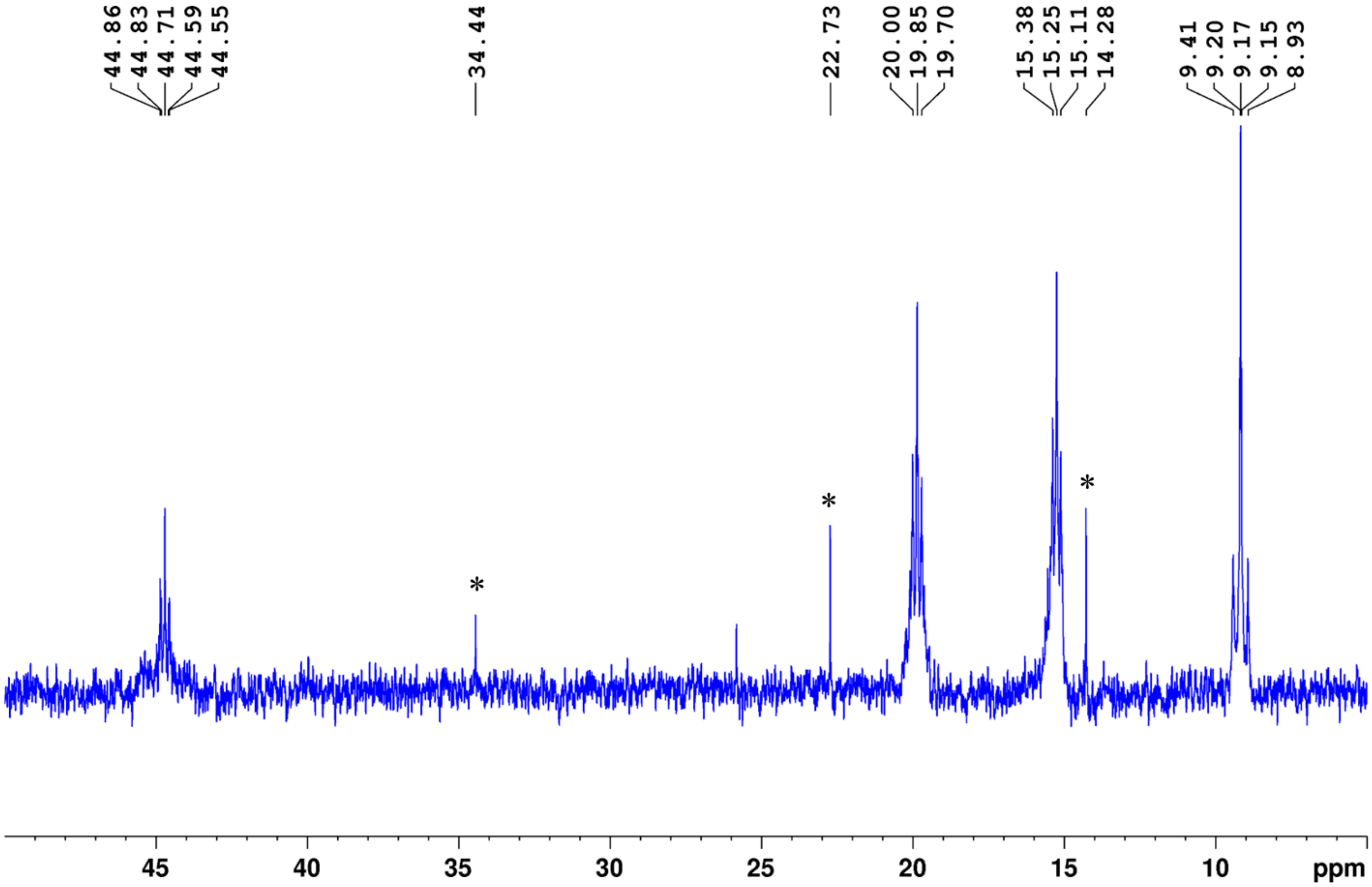

Figure S25. $^{13}C\{^1H\}$ NMR spectrum of $\mathbf{3^{SiMe3}}$**-Cl** in C_6D_6. The additional resonances marked * correspond to residual pentane.

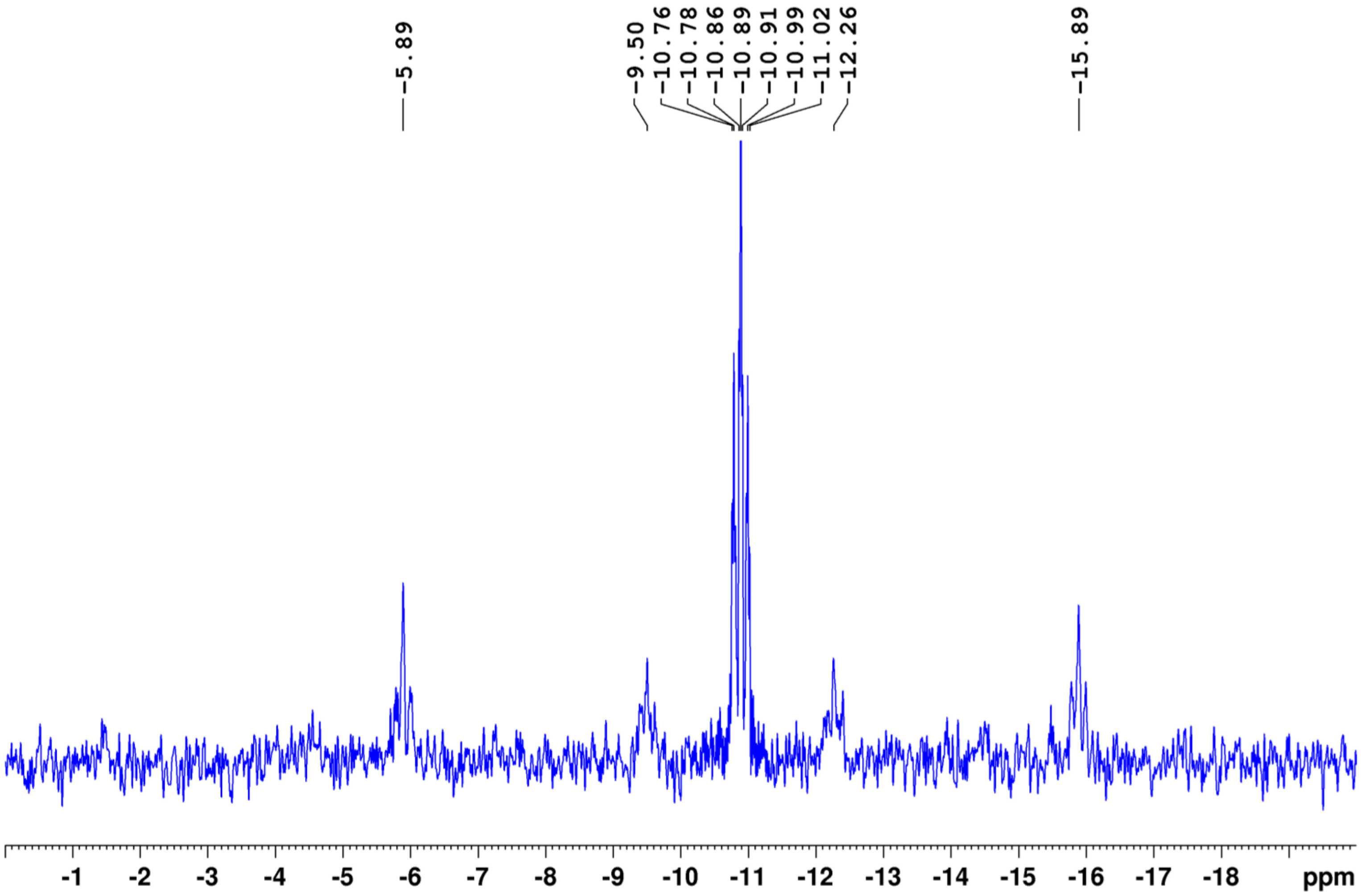

Figure S26. $^{29}Si\{^1H\}$ NMR spectrum of **3^{SiMe3}-Cl** in C_6D_6.

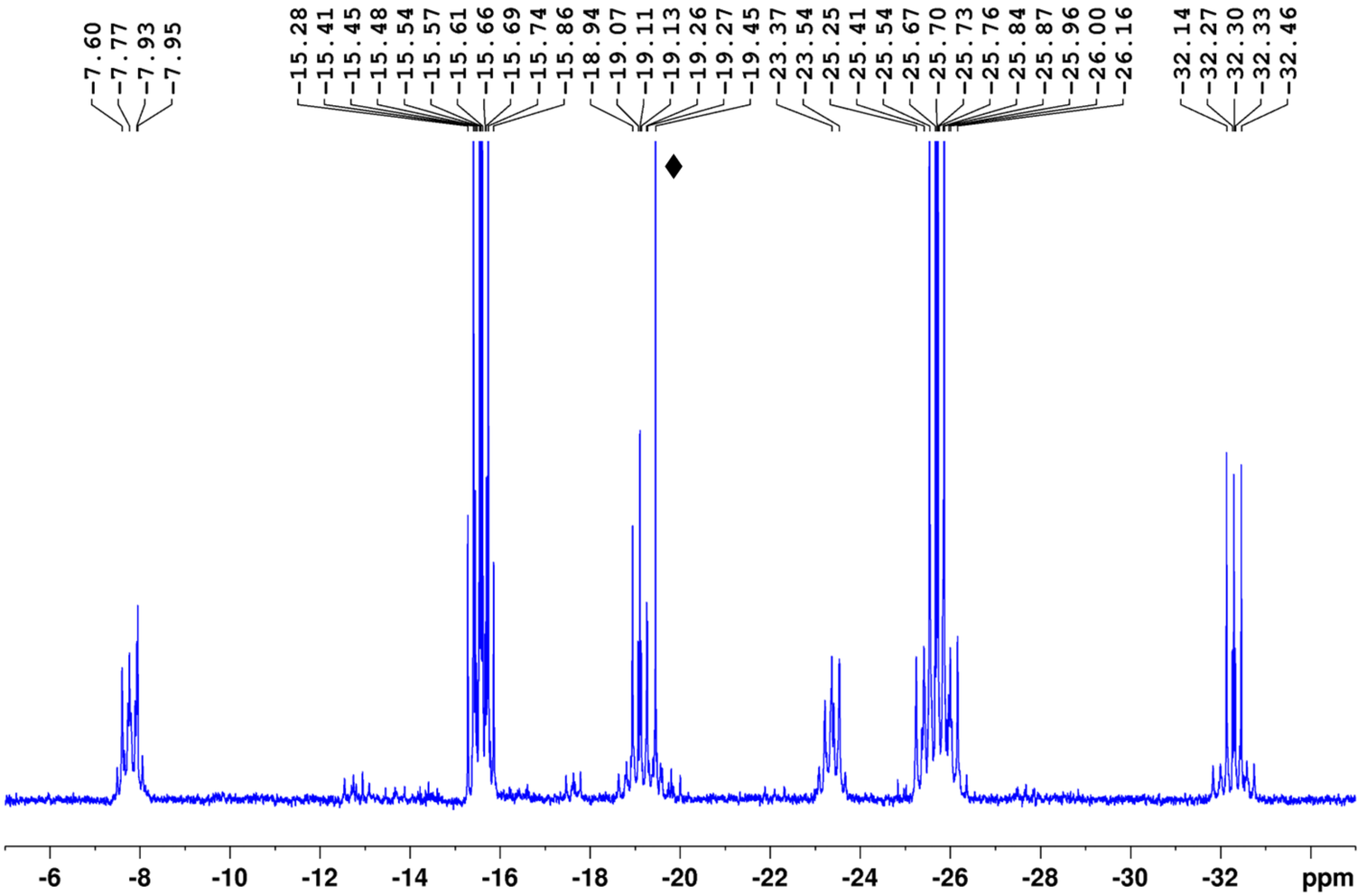

Figure S27. $^{31}P\{^{1}H\}$ NMR spectrum of **3^{SiMe3}-Cl** in C_6D_6. The additional resonance marked ♦ correspond to the decomposition product [(*μ*–dmpm)$_2$Pt$_2$Cl$_2$] (ca. 4%).

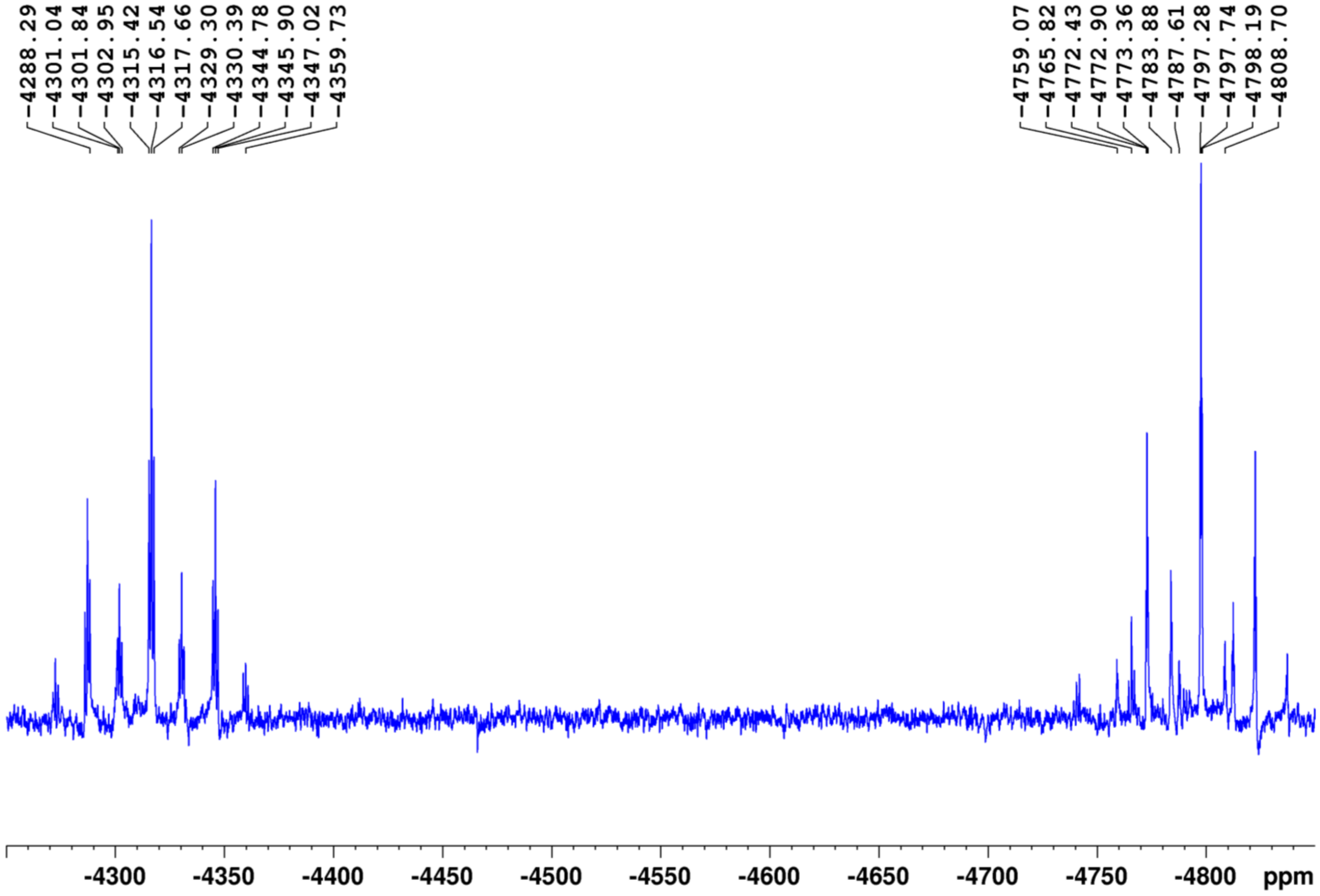

Figure S28. $^{195}Pt\{^{1}H\}$ NMR spectrum of $\mathbf{3^{SiMe3}}$**-Cl** in C_6D_6.

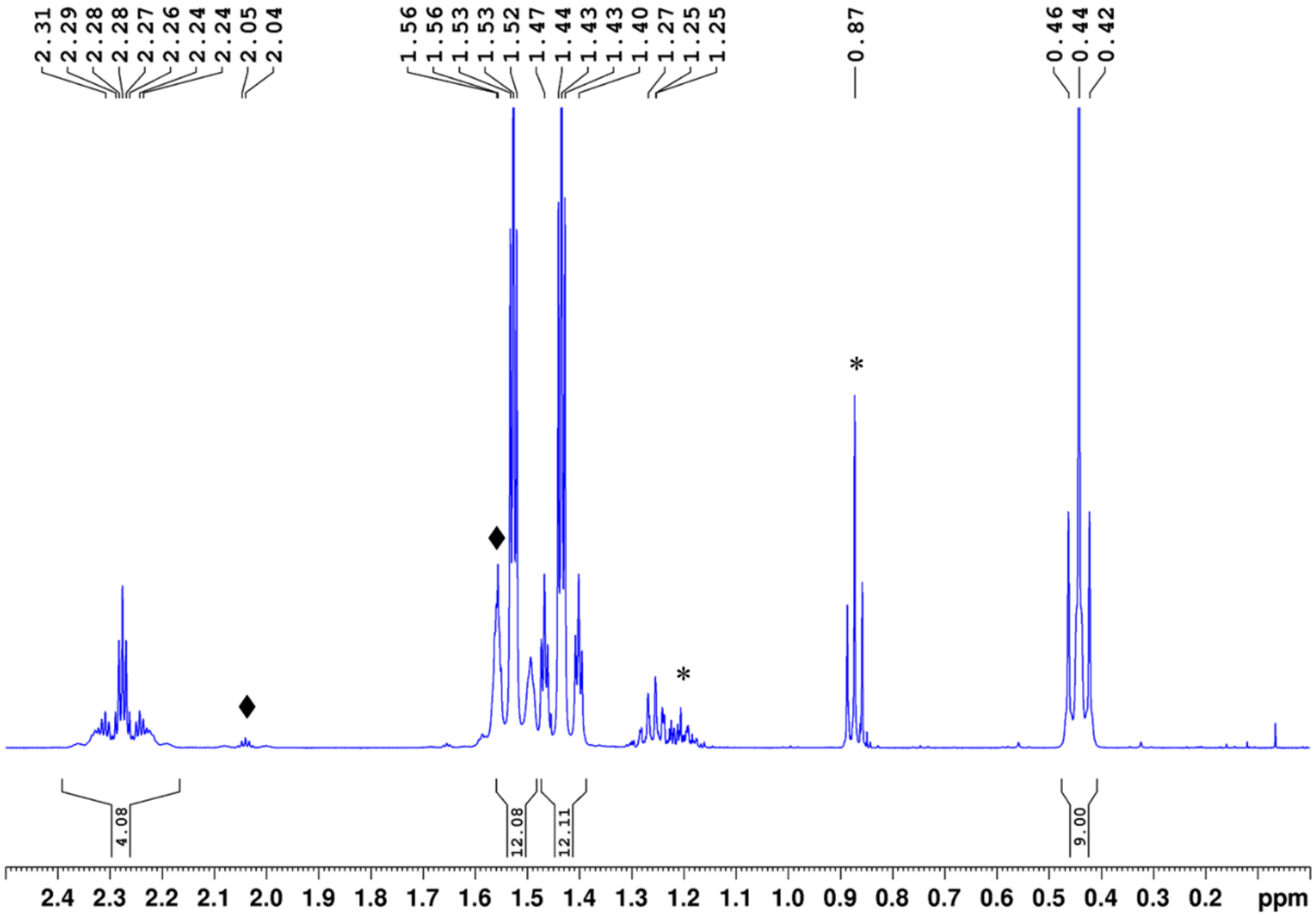

Figure S29. ^{1}H NMR spectrum of **3^{SiMe3}-I** in C_6D_6. The additional resonances marked ♦ correspond to the decomposition product [(*μ*–dmpm)$_2$Pt$_2$I$_2$], those marked * to residual pentane.

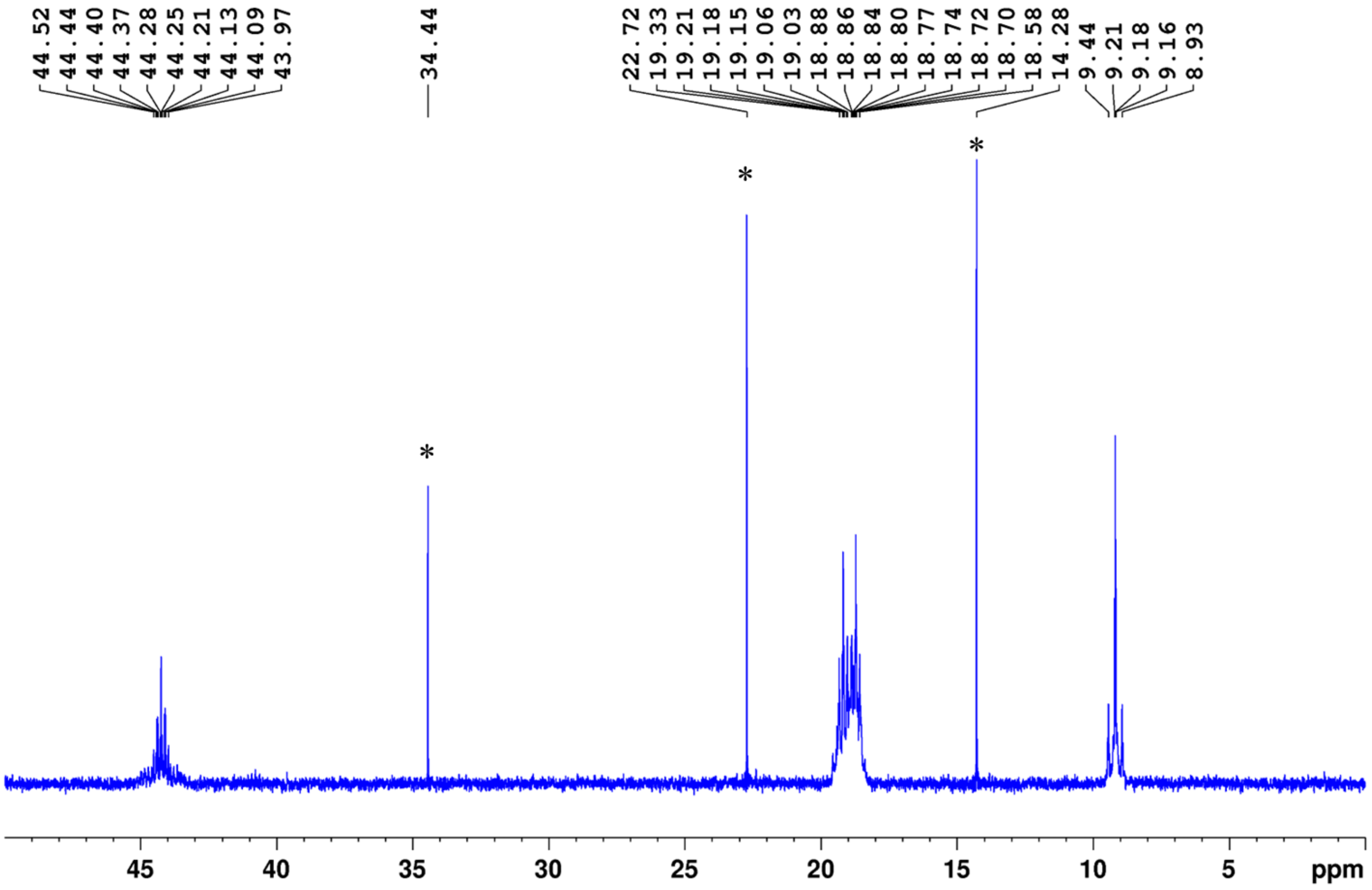

Figure S30. $^{13}C\{^{1}H\}$ NMR spectrum of $\mathbf{3^{SiMe3}}$**-I** in C_6D_6. The additional resonances marked * correspond to residual pentane.

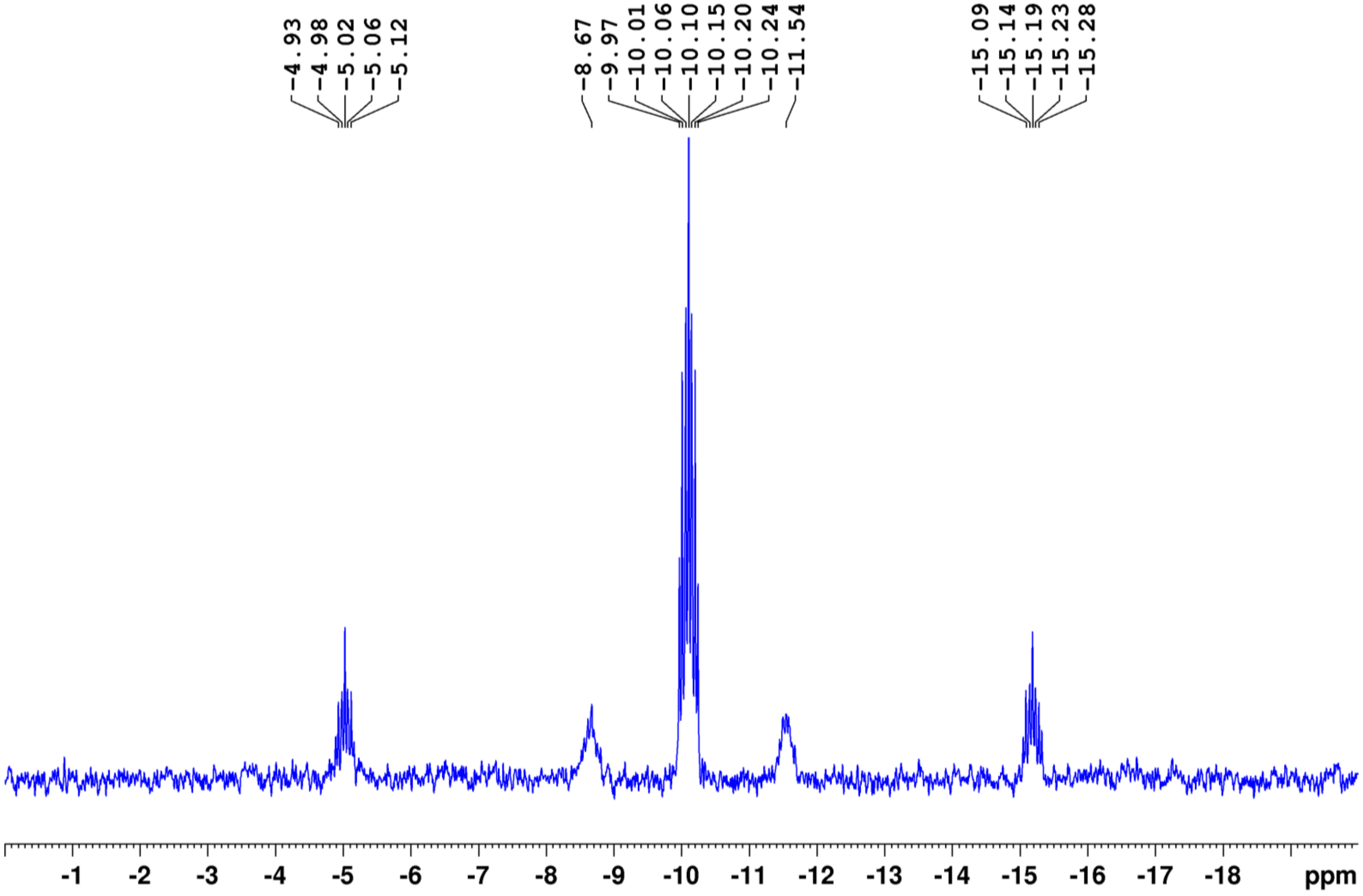

Figure S31. $^{29}Si\{^1H\}$ NMR spectrum of **3^{SiMe3}-I** in C_6D_6.

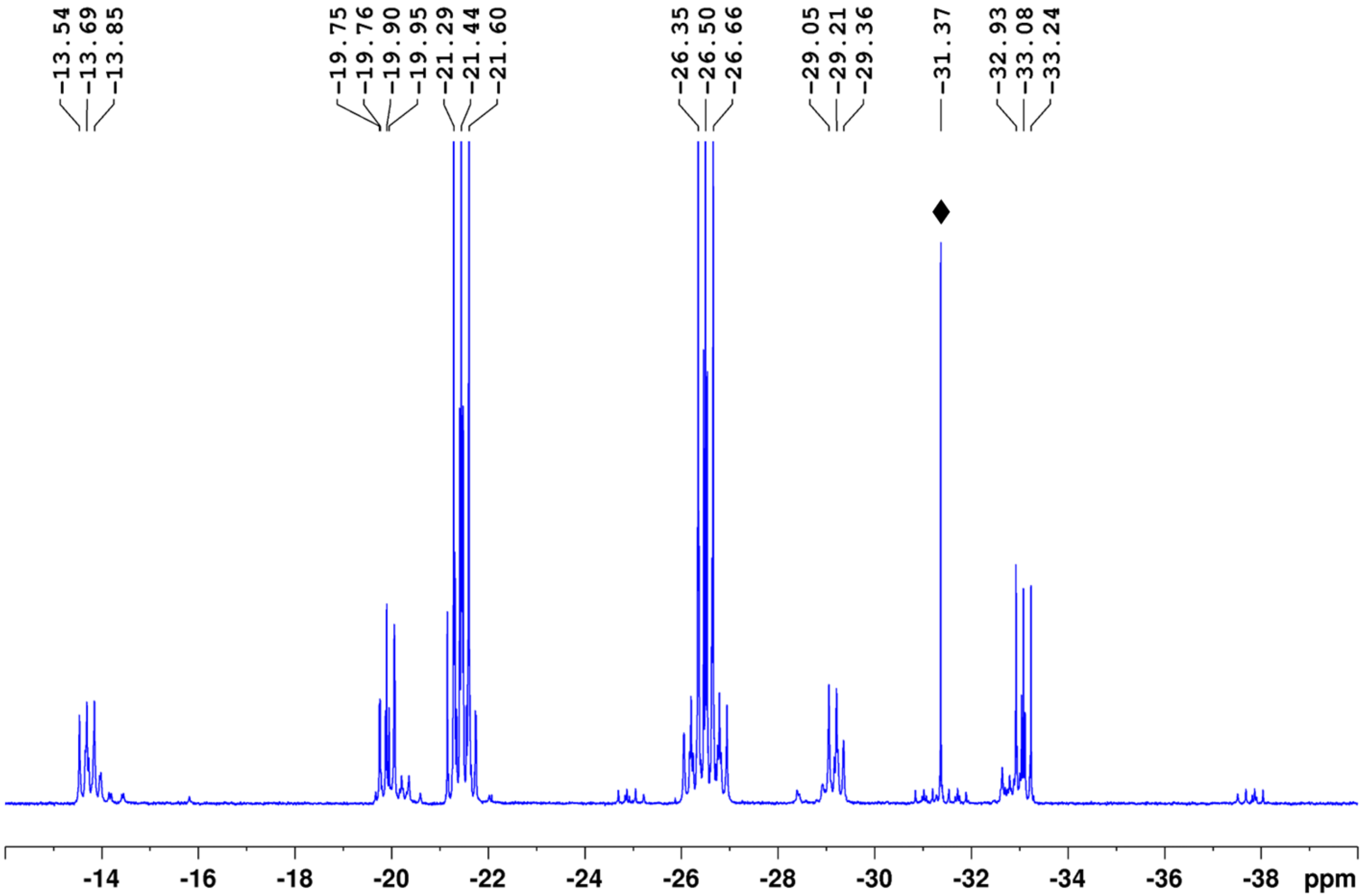

Figure S32. $^{31}P\{^{1}H\}$ NMR spectrum of $\mathbf{3^{SiMe3}}$**-I** in C_6D_6. The additional resonance marked ♦ corresponds to the decomposition product [(μ–dmpm)$_2Pt_2I_2$] (ca. 5%).

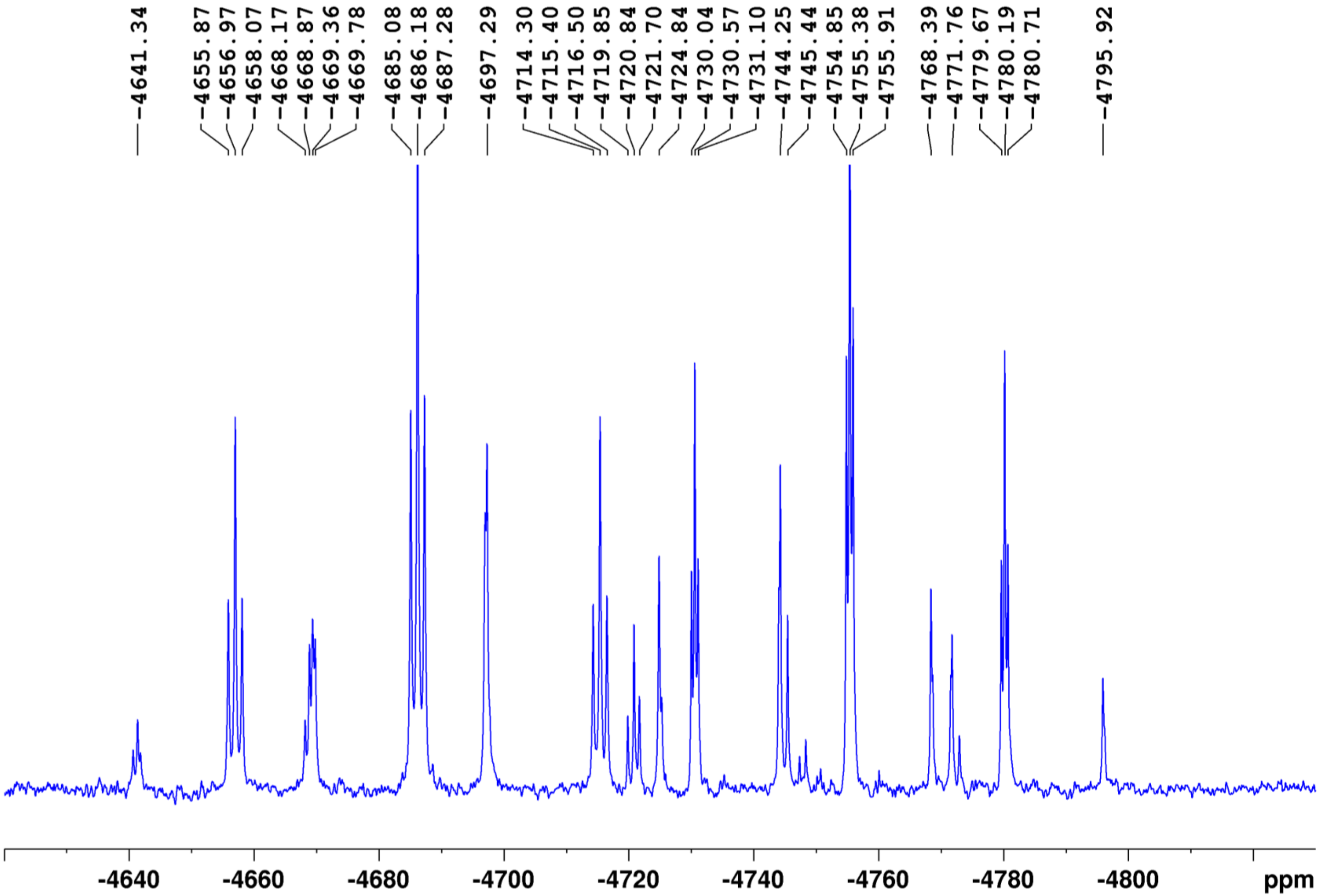

Figure S33. $^{195}Pt\{^{1}H\}$ NMR spectrum of $\mathbf{3^{SiMe3}}$**-I** in C_6D_6.

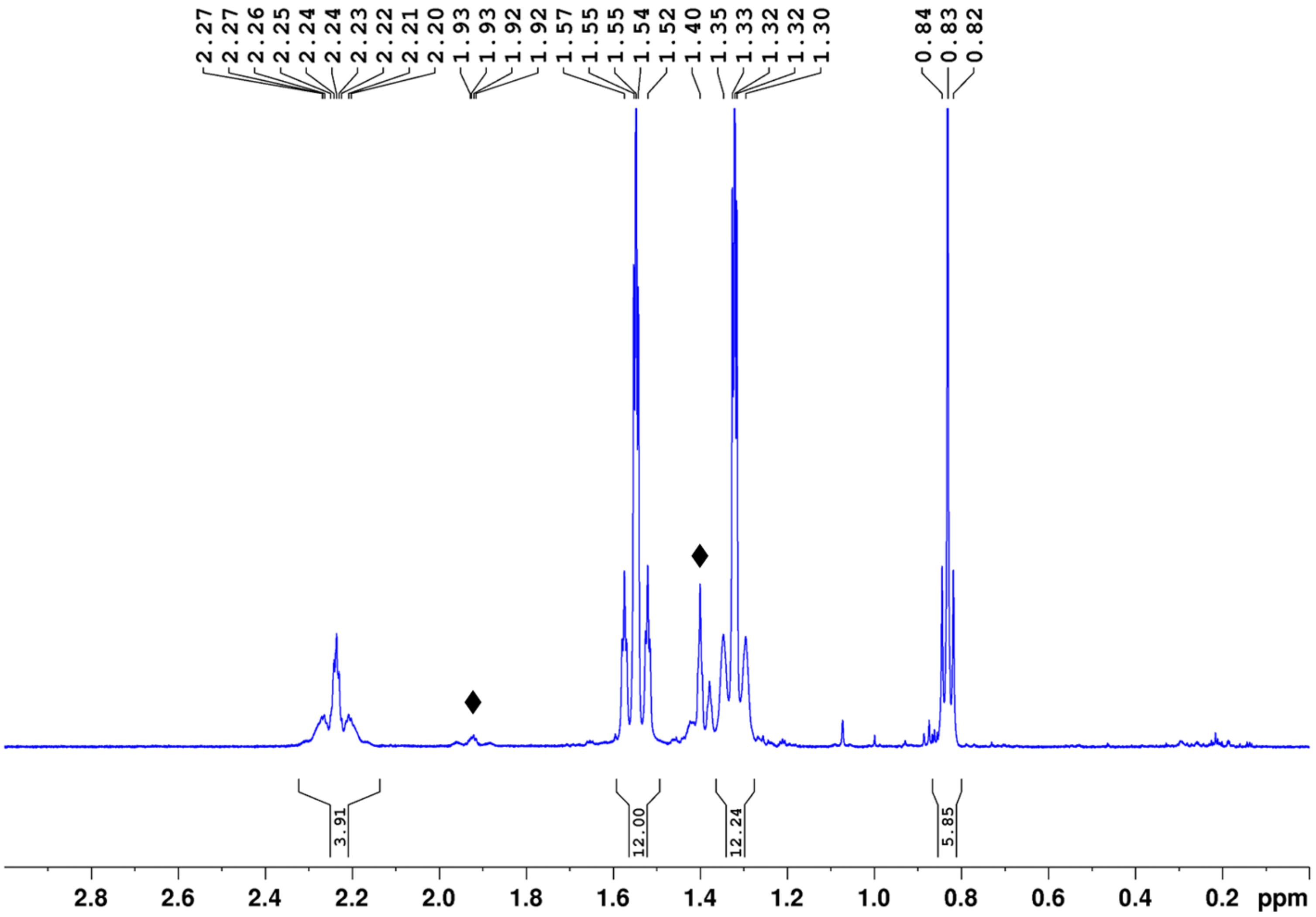

Figure S34. ^{1}H NMR spectrum of **3^{SiMe2Cl}-Cl** in C_6D_6. The additional resonances marked ♦ correspond to the decomposition product [(*μ*–dmpm)$_2$Pt$_2$Cl$_2$].

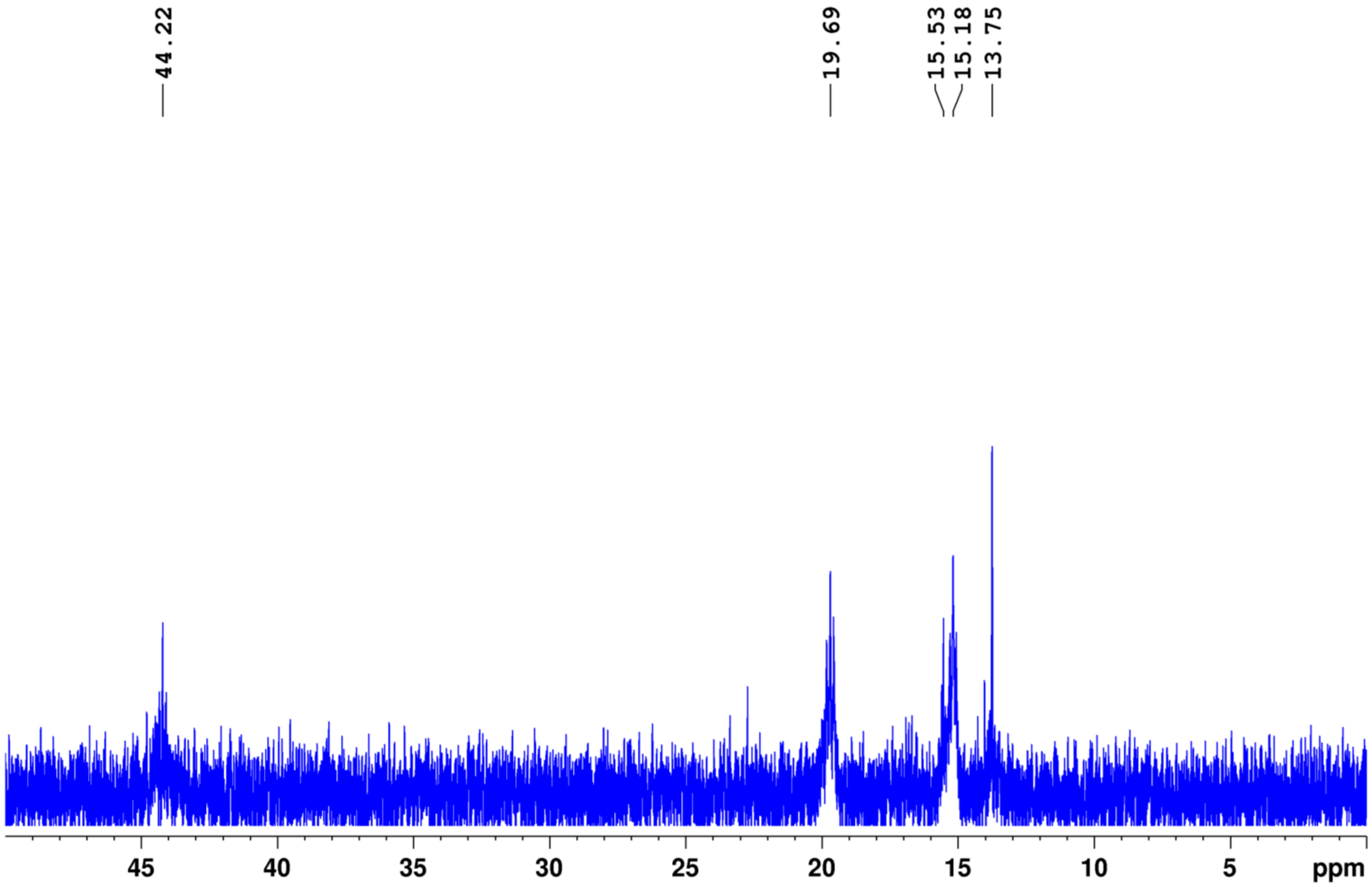

Figure S35. $^{13}C\{^{1}H\}$ NMR spectrum of $\mathbf{3^{SiMe2Cl}}$**-Cl** in C_6D_6.

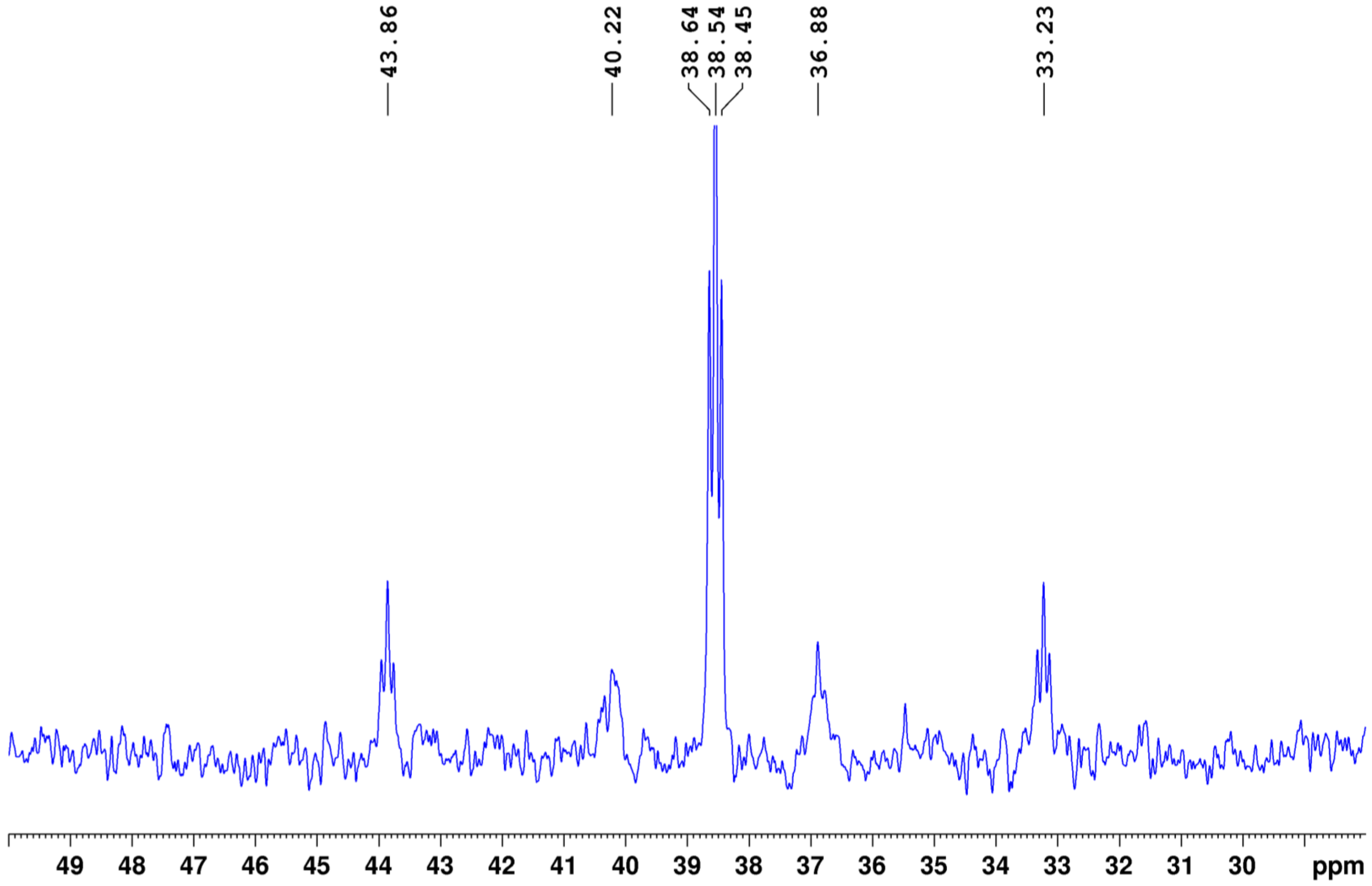

Figure S36. $^{29}Si\{^1H\}$ NMR spectrum of $\mathbf{3^{SiMe2Cl}}$**-Cl** in C_6D_6.

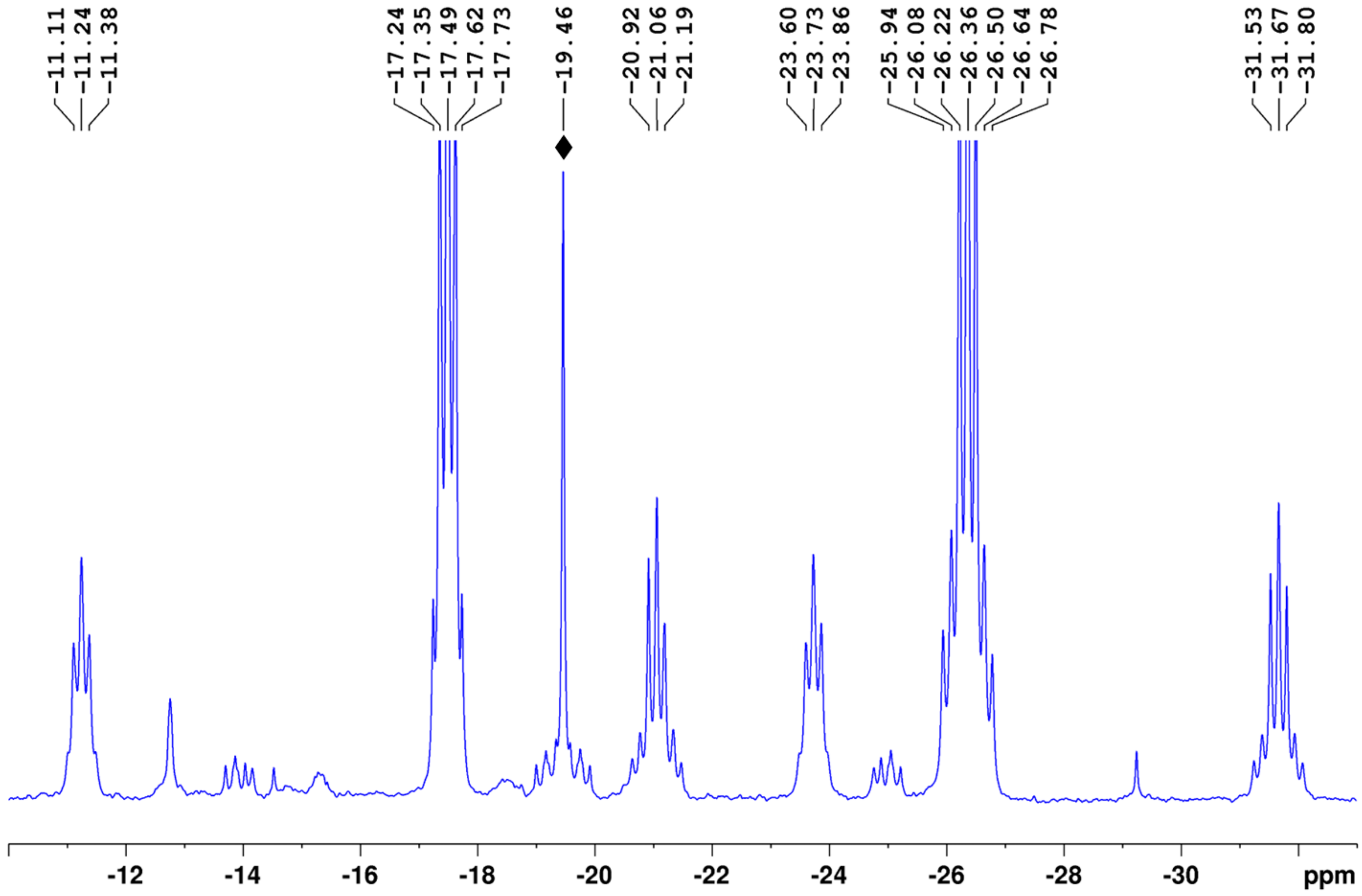

Figure S37. $^{31}P\{^{1}H\}$ NMR spectrum of $\mathbf{3^{SiMe2Cl}}$**-Cl** in C_6D_6. The additional resonance marked ♦ corresponds to the decomposition product [(*μ*–dmpm)$_2Pt_2Cl_2$] (ca. 9%).

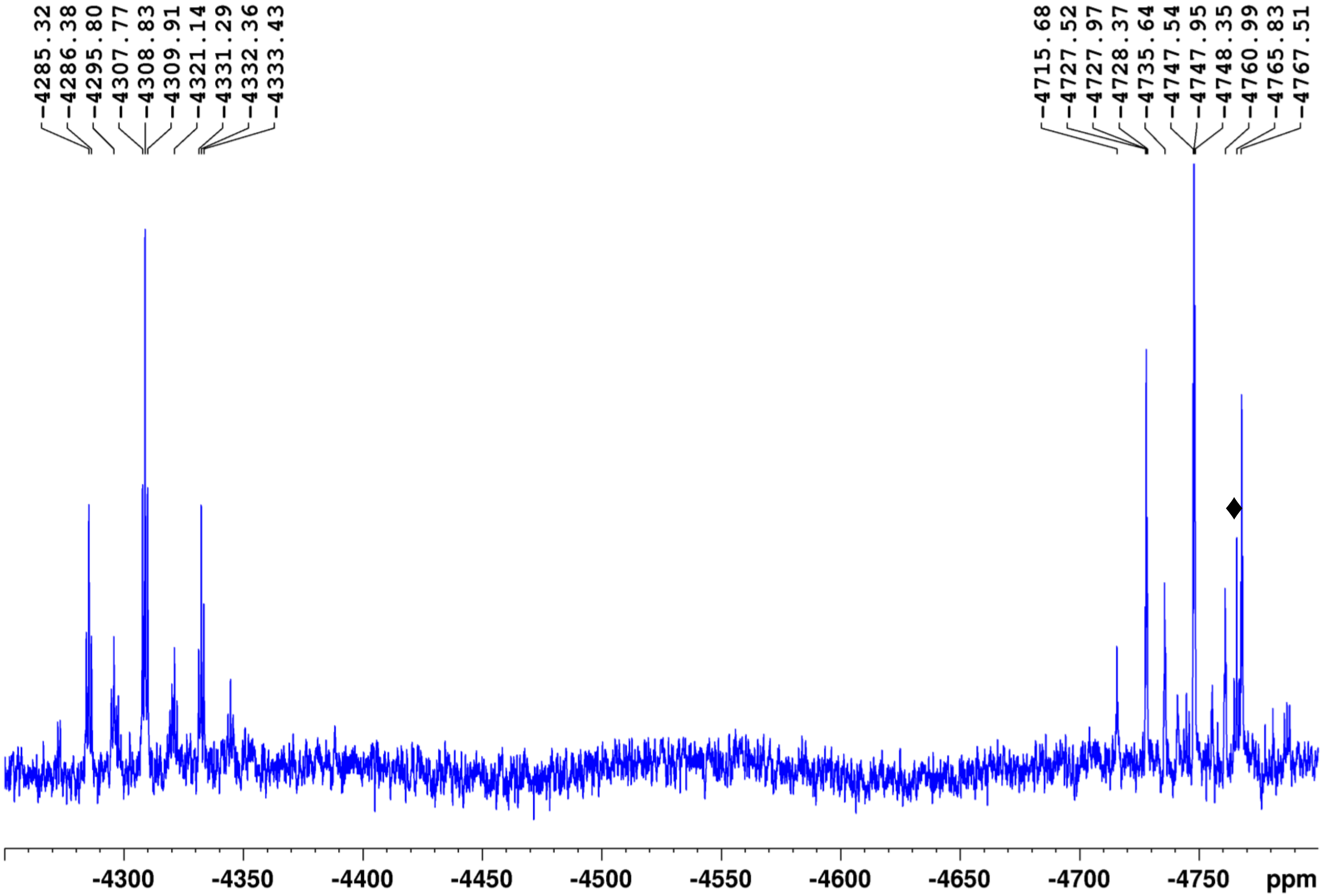

Figure S38. $^{195}Pt\{^1H\}$ NMR spectrum of $\mathbf{3^{SiMe2Cl}}$**-Cl** in C_6D_6. The additional resonance marked ♦ corresponds to the decomposition product [(*μ*–dmpm)$_2$Pt$_2$Cl$_2$]. The additional resonance marked ♦ correspond to the decomposition product [(*μ*–dmpm)$_2$Pt$_2$Cl$_2$].

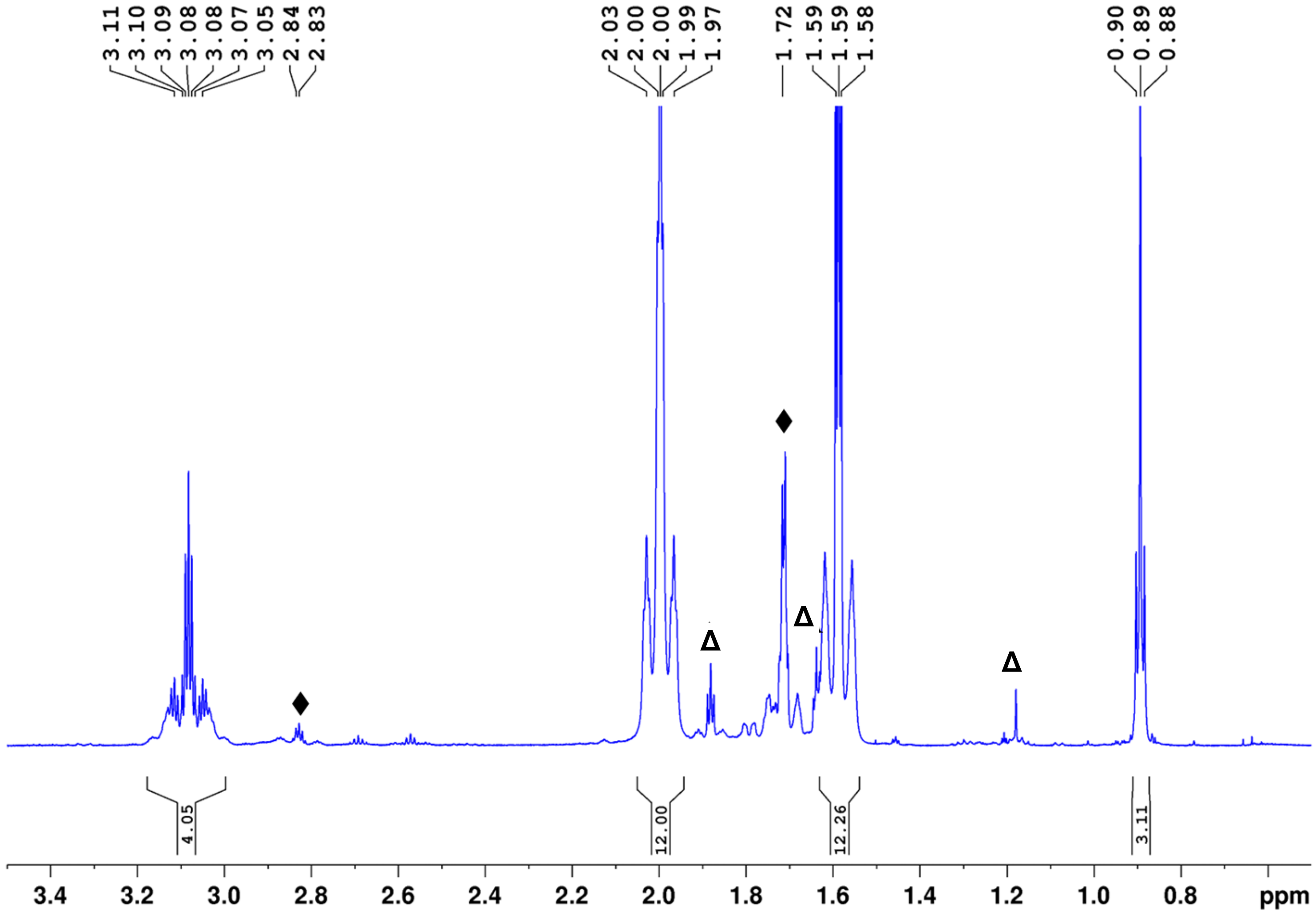

Figure S39. ^{1}H NMR spectrum of **3^{SiMeCl2}-Cl** in CD_2Cl_2. The additional resonances marked ♦ correspond to the decomposition product [(*μ*–dmpm)$_2$$Pt_2Cl_2$], those marked **Δ** to the HCl hydrolysis complex **4^{SiMeCl2}-HCl**.

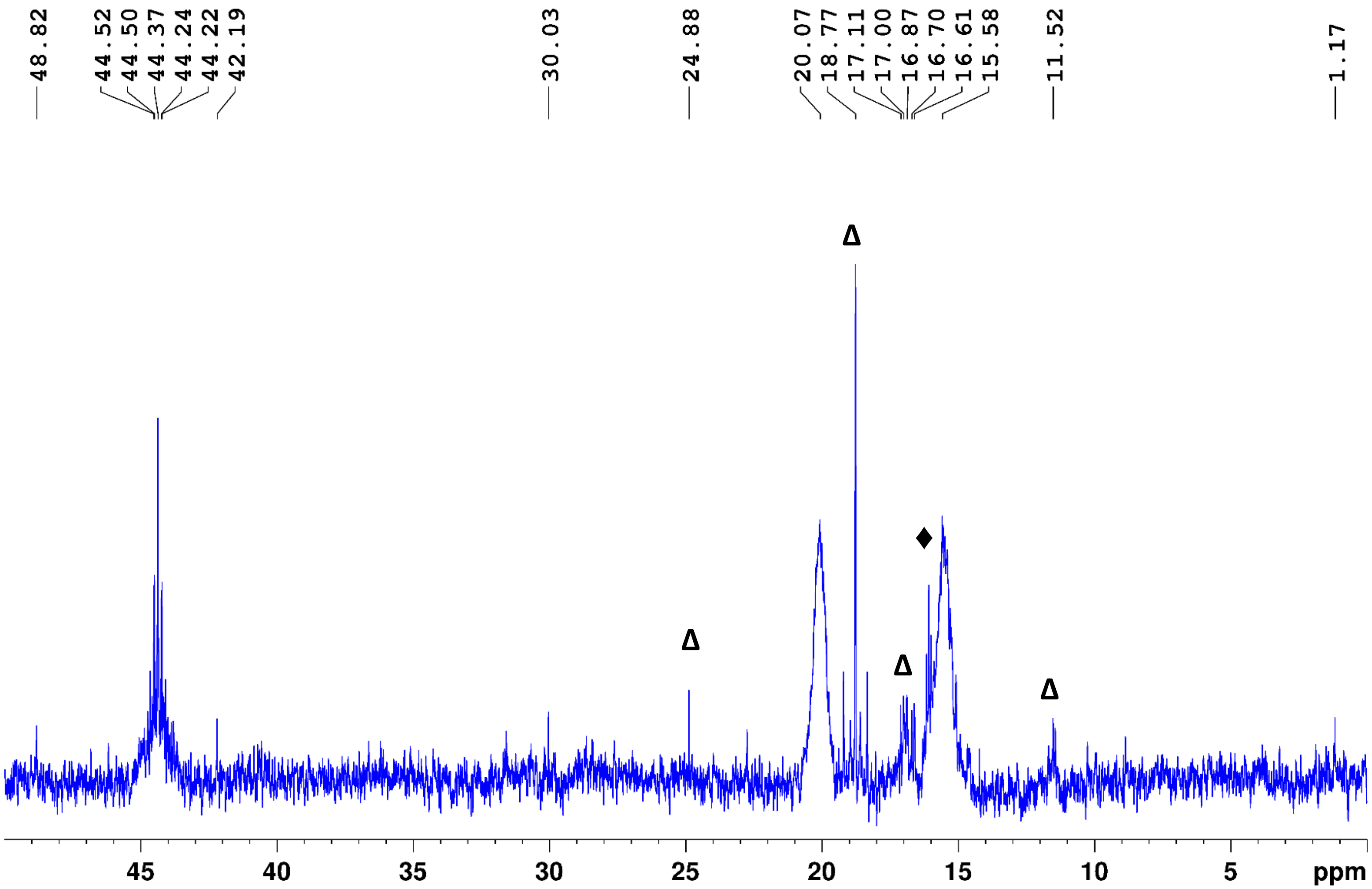

Figure S40. $^{13}C\{^{1}H\}$ NMR spectrum of **$3^{SiMeCl2}$-Cl** in CD_2Cl_2. The additional resonances marked ♦ correspond to the decomposition product [(*μ*–dmpm)$_2$Pt$_2$Cl$_2$], those marked **Δ** to the HCl hydrolysis complex **$4^{SiMeCl2}$-HCl**.

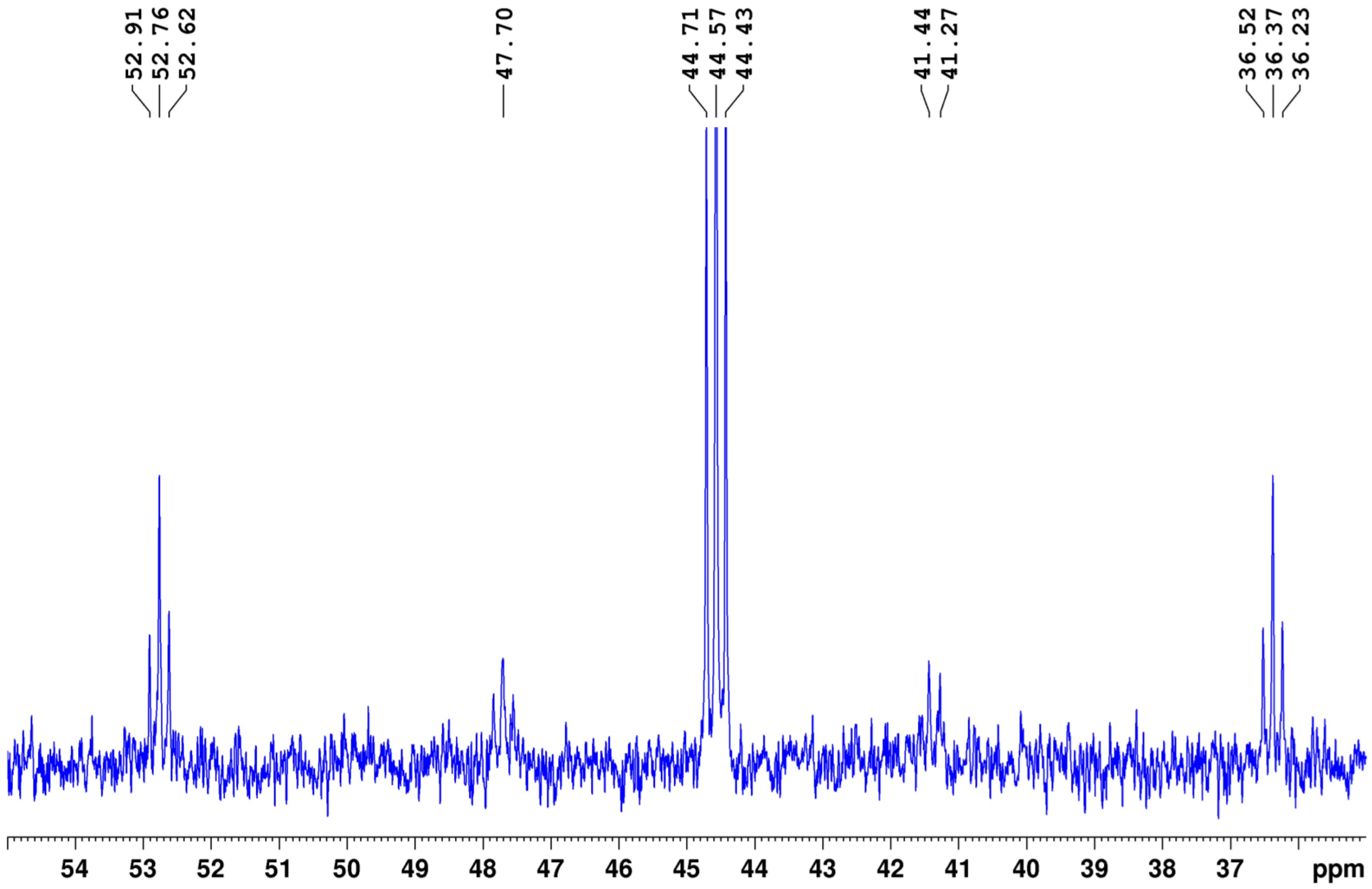

Figure S41. $^{29}Si\{^{1}H\}$ NMR spectrum of **3^{SiMeCl2}-Cl** in CD_2Cl_2.

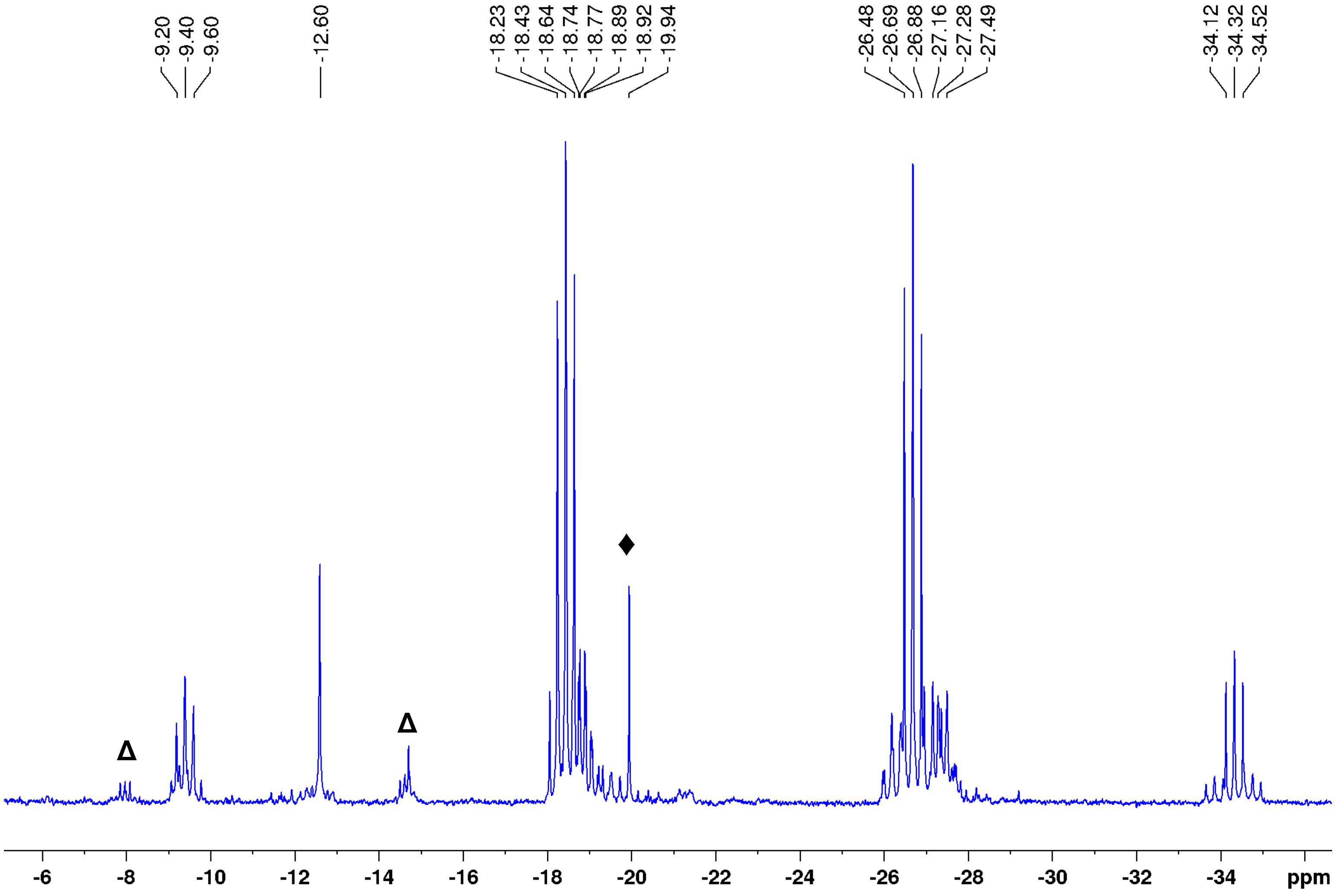

Figure S42. $^{31}P\{^{1}H\}$ NMR spectrum of **$3^{SiMeCl2}$-Cl** in CD_2Cl_2. The additional resonances marked ♦ correspond to the decomposition product [(μ–dmpm)$_2Pt_2Cl_2$] (ca. 2%), those marked **Δ** to the HCl hydrolysis complex **$4^{SiMeCl2}$-HCl** (ca. 2%).

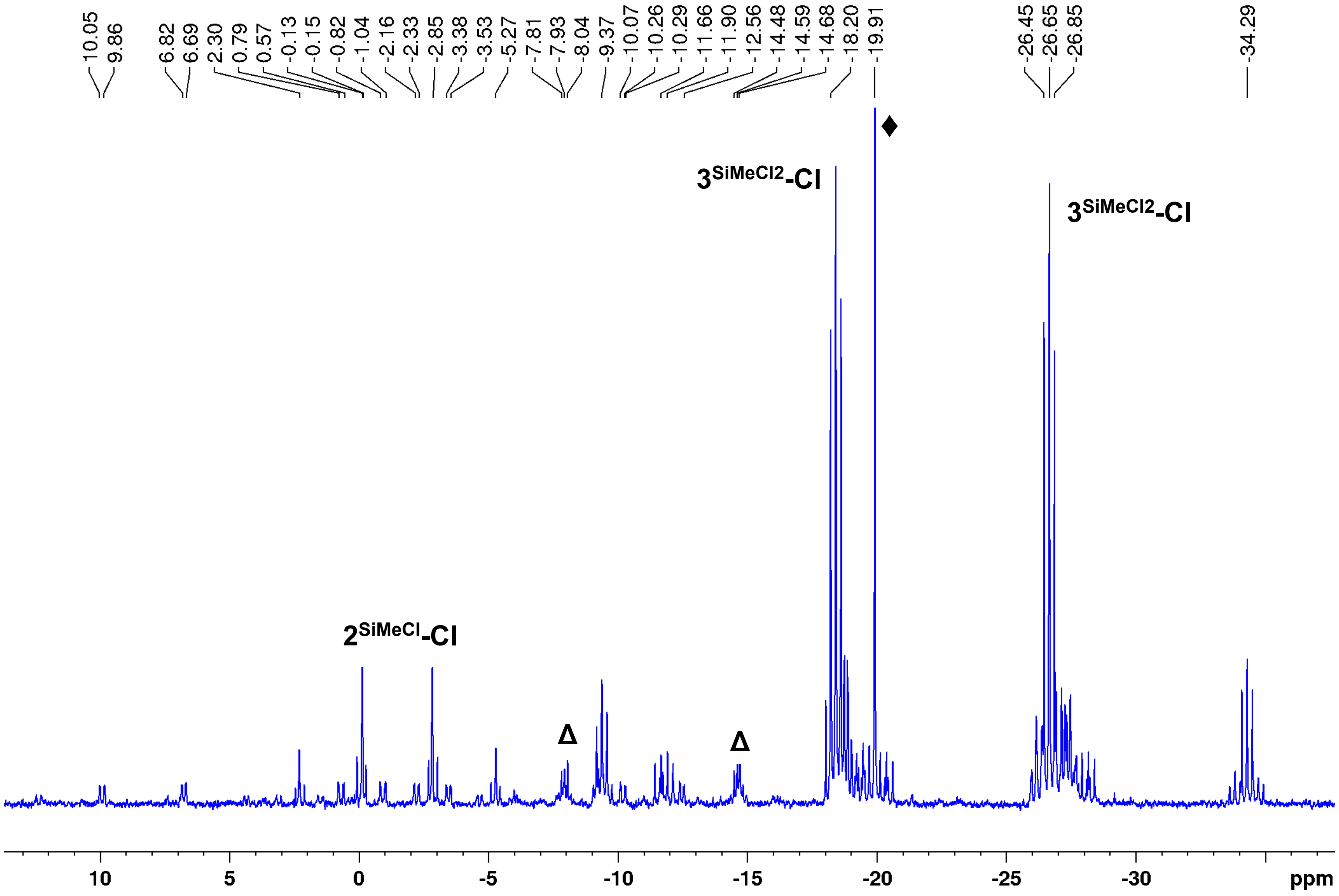

Figure S43. $^{31}P\{^{1}H\}$ NMR spectrum after 2 days of heating $\mathbf{3}^{SiMeCl2}$**-Cl** at 60 °C in CD_2Cl_2, showing the formation of $\mathbf{2}^{SiMeCl}$**-Cl** (ca. 16%). The additional resonances marked ♦ correspond to the decomposition product $[(\mu\text{–dmpm})_2Pt_2Cl_2]$ (ca. 17%), those marked **Δ** to the HCl hydrolysis complex $\mathbf{4}^{SiMeCl2}$**-HCl** (ca. 4%).

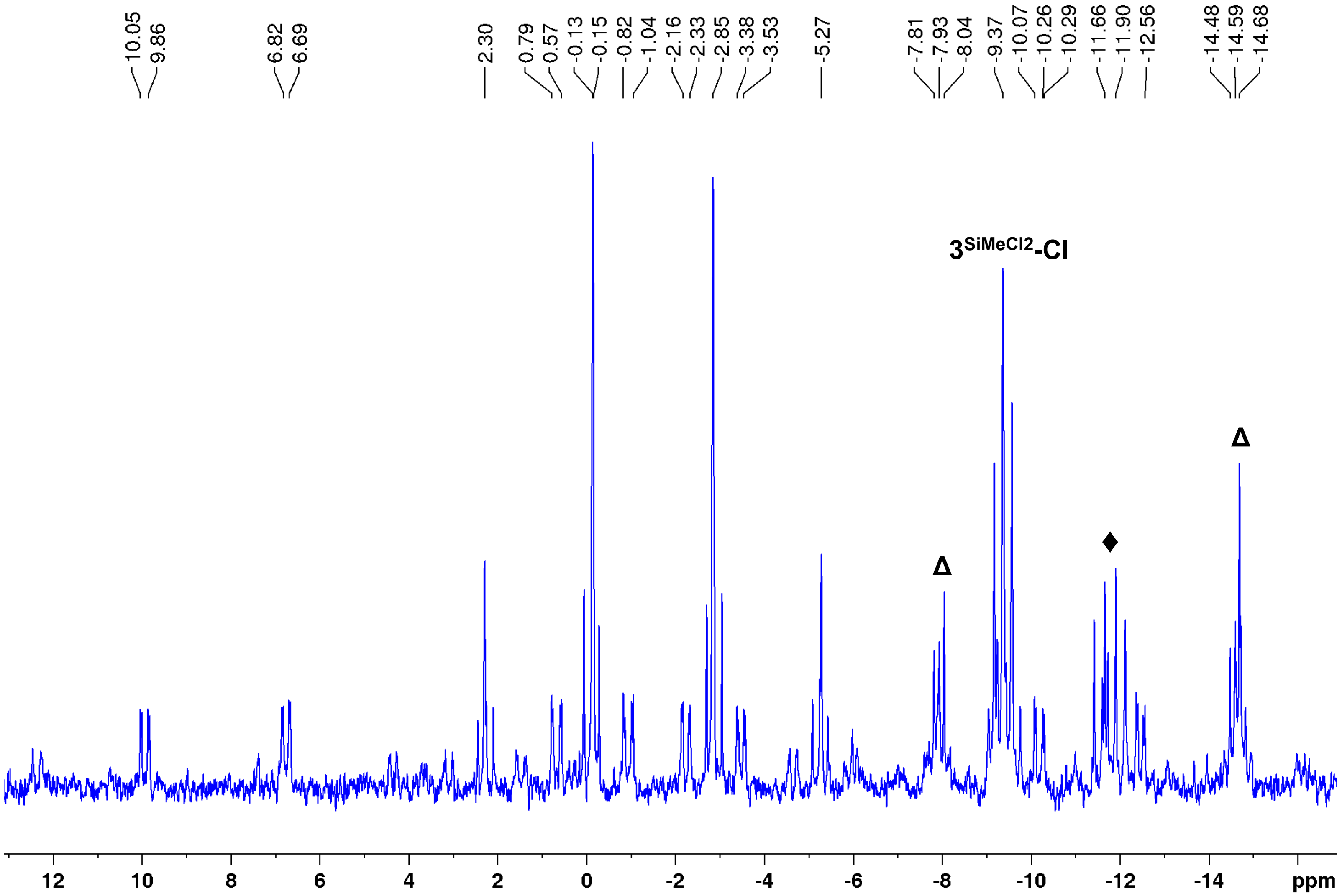

Figure S44. Expansion of the $^{31}P\{^{1}H\}$ NMR resonances of $\mathbf{2^{SiMeCl}}$**-Cl**. The additional resonances marked ♦ correspond to the decomposition product $[(\mu\text{–dmpm})_2Pt_2Cl_2]$, those marked **Δ** to the HCl hydrolysis complex $\mathbf{4^{SiMeCl2}}$**-HCl** (ca. 4%).

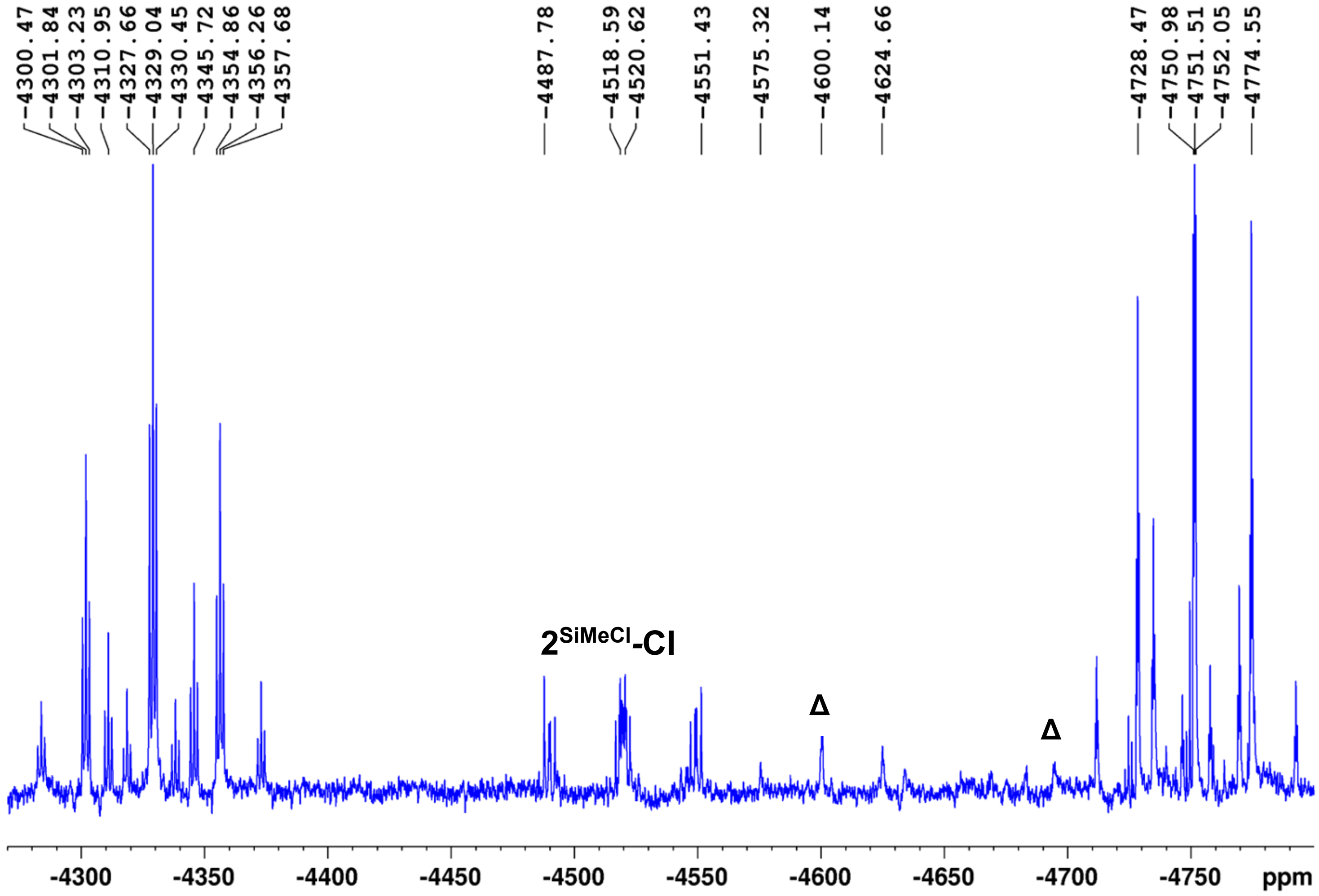

Figure S45. $^{195}Pt\{^{1}H\}$ NMR spectrum of $\mathbf{3^{SiMeCl2}}$**-Cl** in CD_2Cl_2. The additional resonance marked **Δ** correspond to the HCl hydrolysis complex $\mathbf{4^{SiMeCl2}}$**-HCl**.

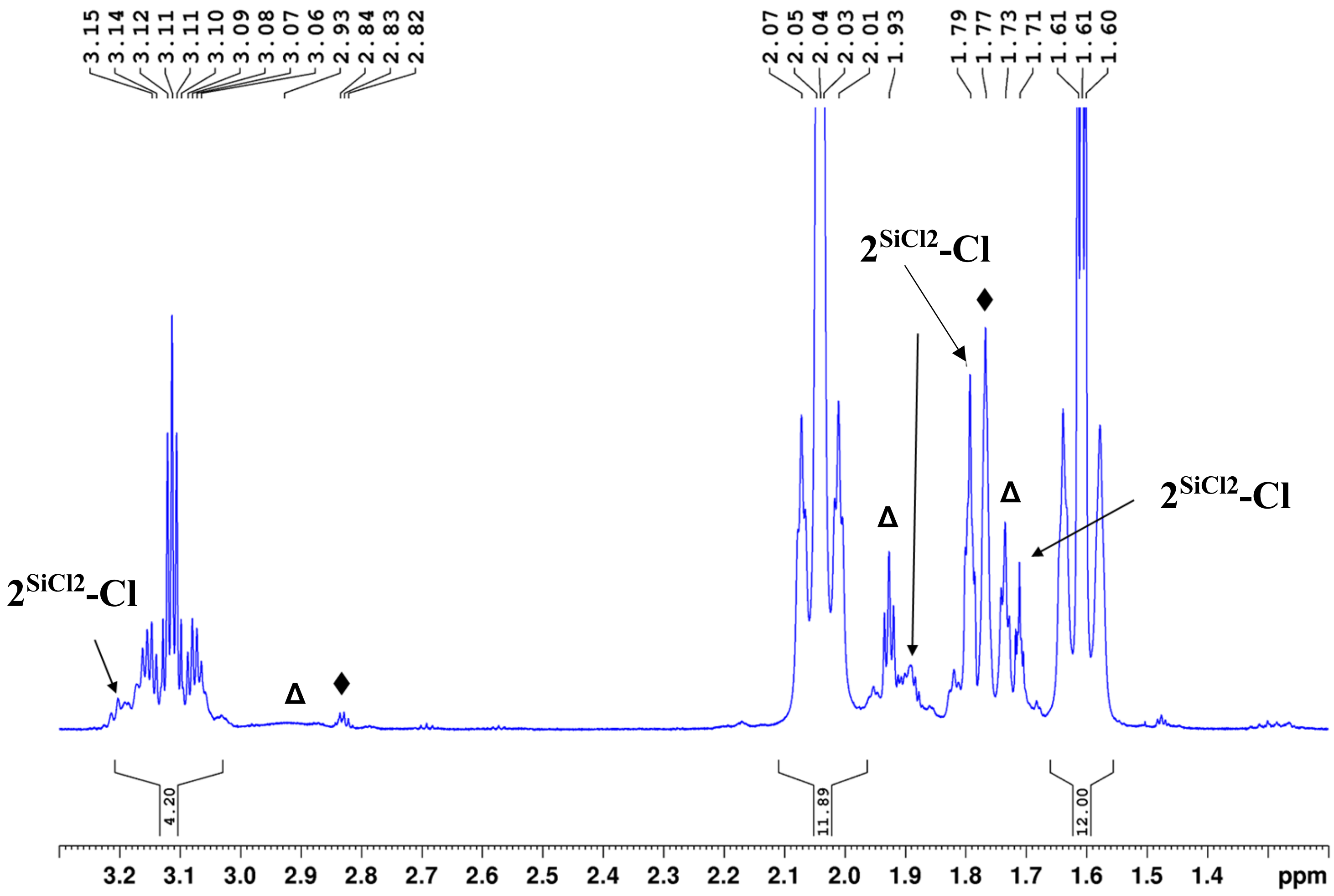

Figure S46. ^{1}H NMR spectrum of $\mathbf{3^{SiCl3}}$**-Cl** in CD_2Cl_2, already showing rearrangement to $\mathbf{2^{SiCl2}}$**-Cl**. The additional resonances marked ♦ correspond to the decomposition product [(μ–dmpm)$_2$Pt$_2$Cl$_2$], those marked **Δ** to the HCl hydrolysis complex $\mathbf{4^{SiCl3}}$**-HCl**.

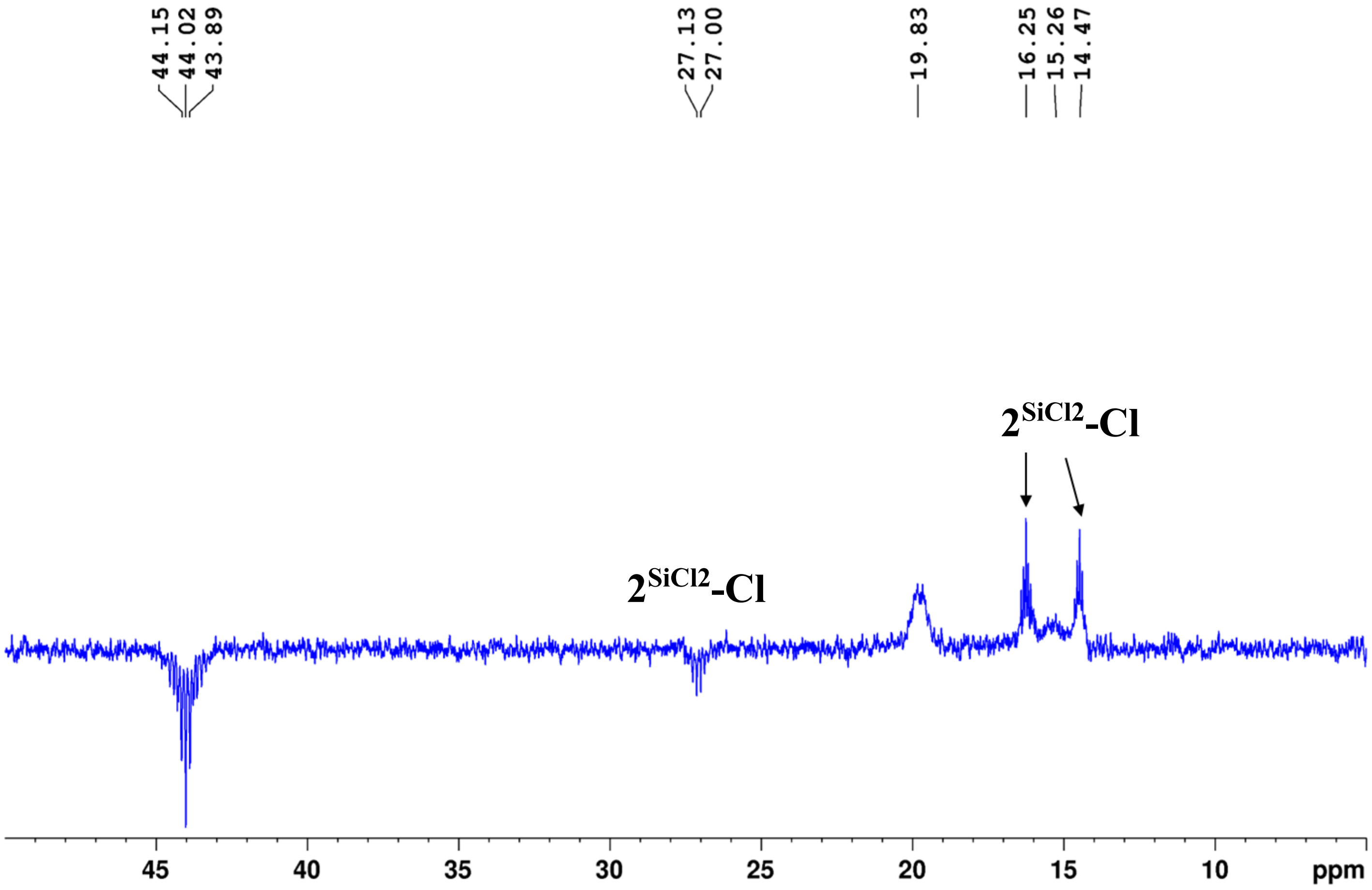

Figure S47. DEPT135-$^{13}C\{^{1}H\}$ NMR spectrum of **3^{SiCl3}-Cl** and its rearrangement product **2^{SiCl2}-Cl** in CD_2Cl_2.

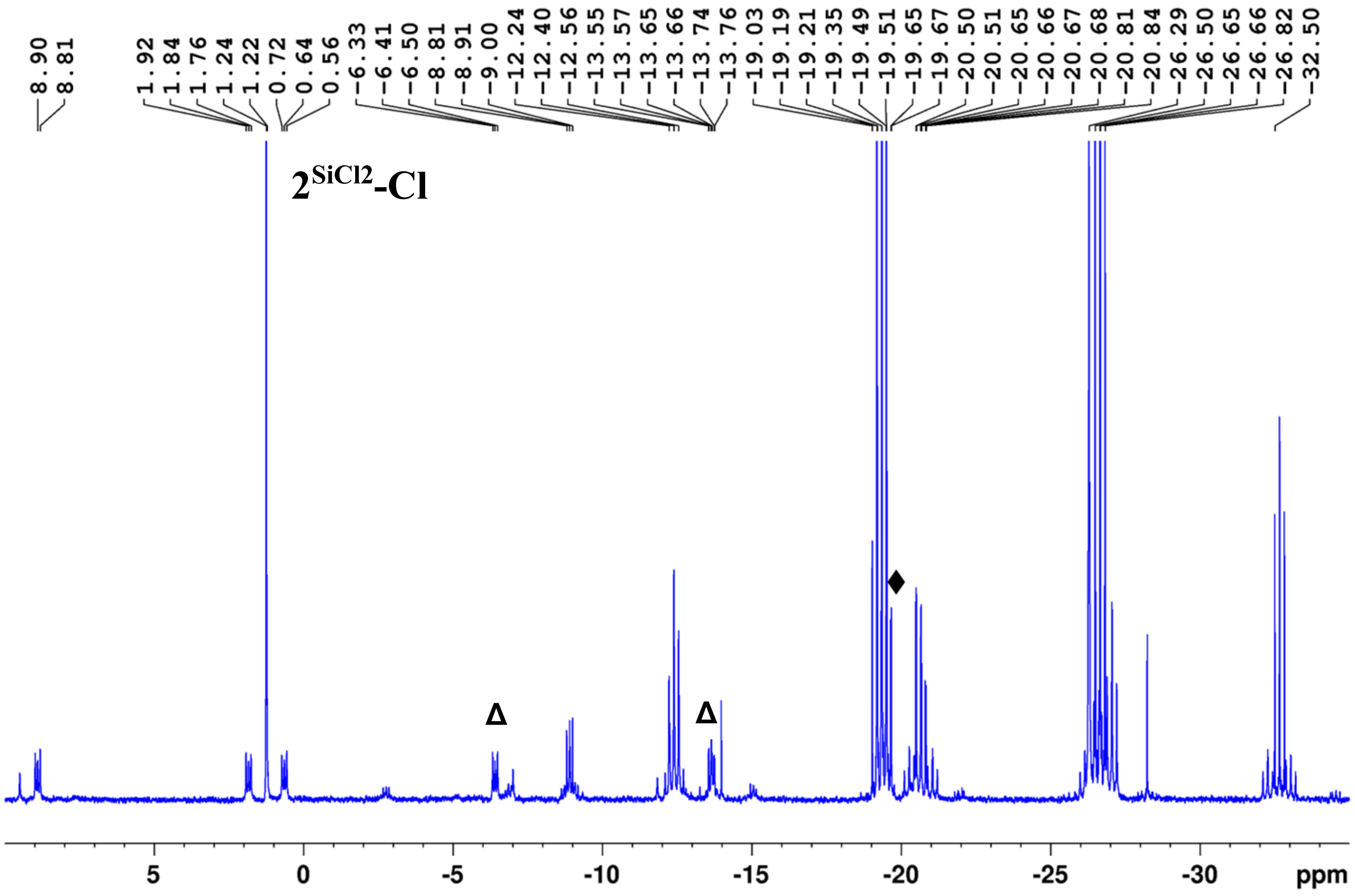

Figure S48. $^{31}P\{^1H\}$ NMR spectrum of **3^{SiCl3}-Cl** and its rearrangement product **2^{SiCl2}-Cl** in CD_2Cl_2. The additional resonances marked ♦ correspond to the decomposition product [(μ–dmpm)$_2$Pt$_2$Cl$_2$] (ca. 1%), those marked **Δ** to the HCl hydrolysis complex **4^{SiCl3}-HCl** (ca. 1%).

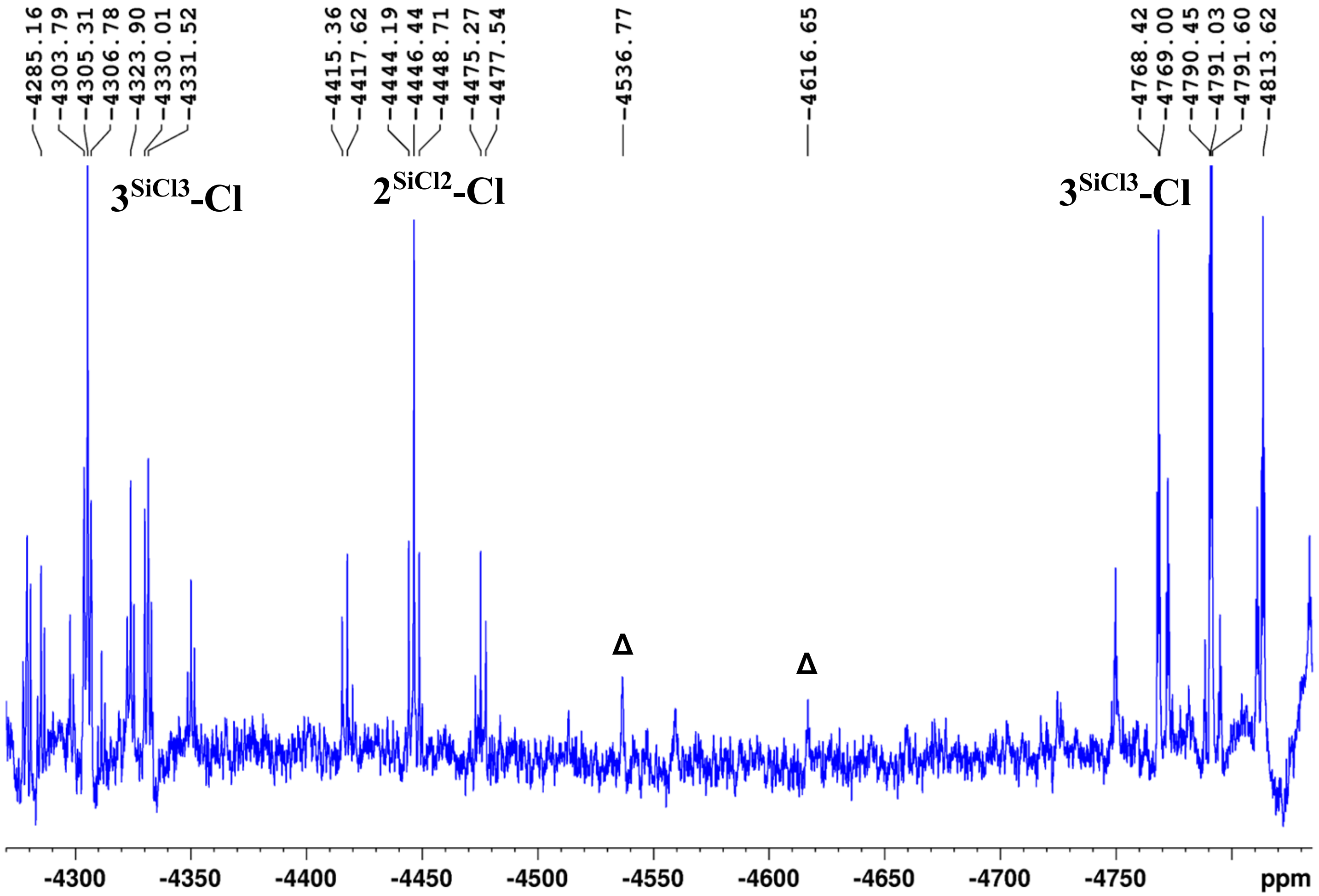

Figure S49. ^{195}Pt{^{1}H} NMR spectrum of **3^{SiCl3}-Cl** and its rearrangement product **2^{SiCl2}-Cl** in CD_2Cl_2. The additional resonances marked **Δ** correspond to the HCl hydrolysis complex **4^{SiCl3}-HCl**.

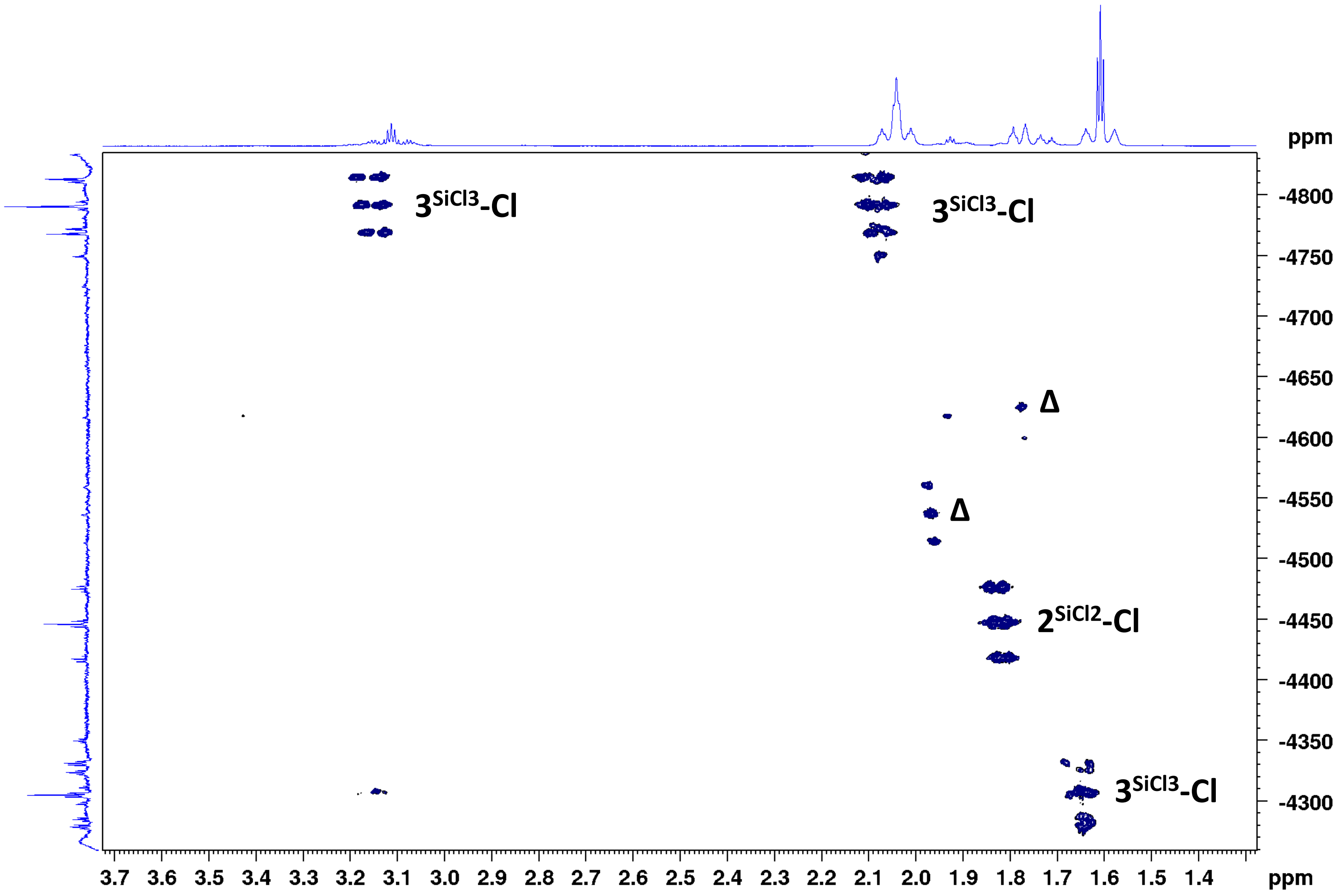

Figure S50. ^{1}H-^{195}Pt HMQC plot of **3^{SiCl3}-Cl** and its rearrangement product **2^{SiCl2}-Cl** in CD_2Cl_2. The additional resonances marked **Δ** correspond to the HCl hydrolysis complex **4^{SiCl3}-HCl**.

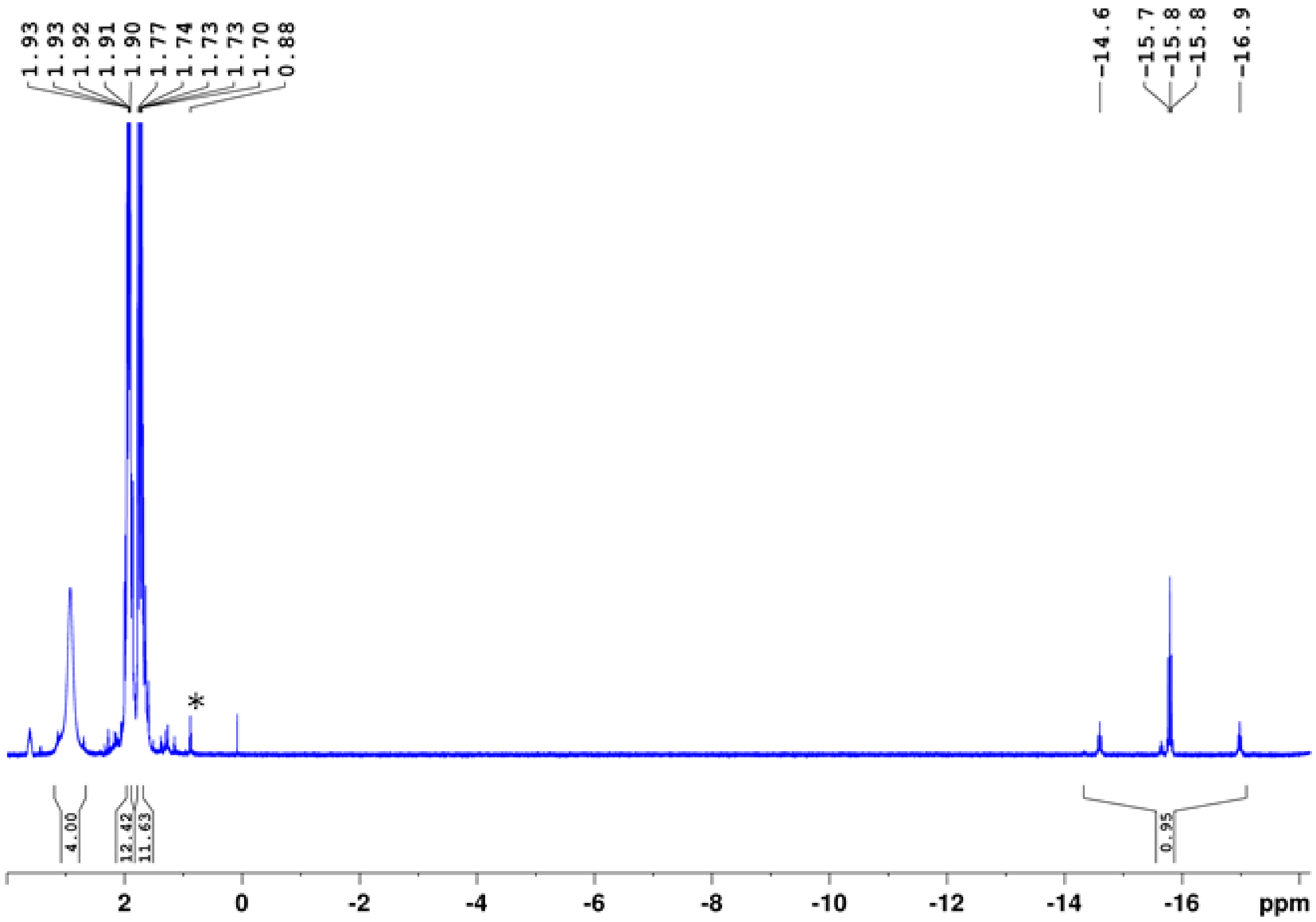

Figure S51. ^{1}H NMR spectrum of **4^{SiCl3}-HCl** in CD_2Cl_2. The additional resonance marked * corresponds to residual pentane.

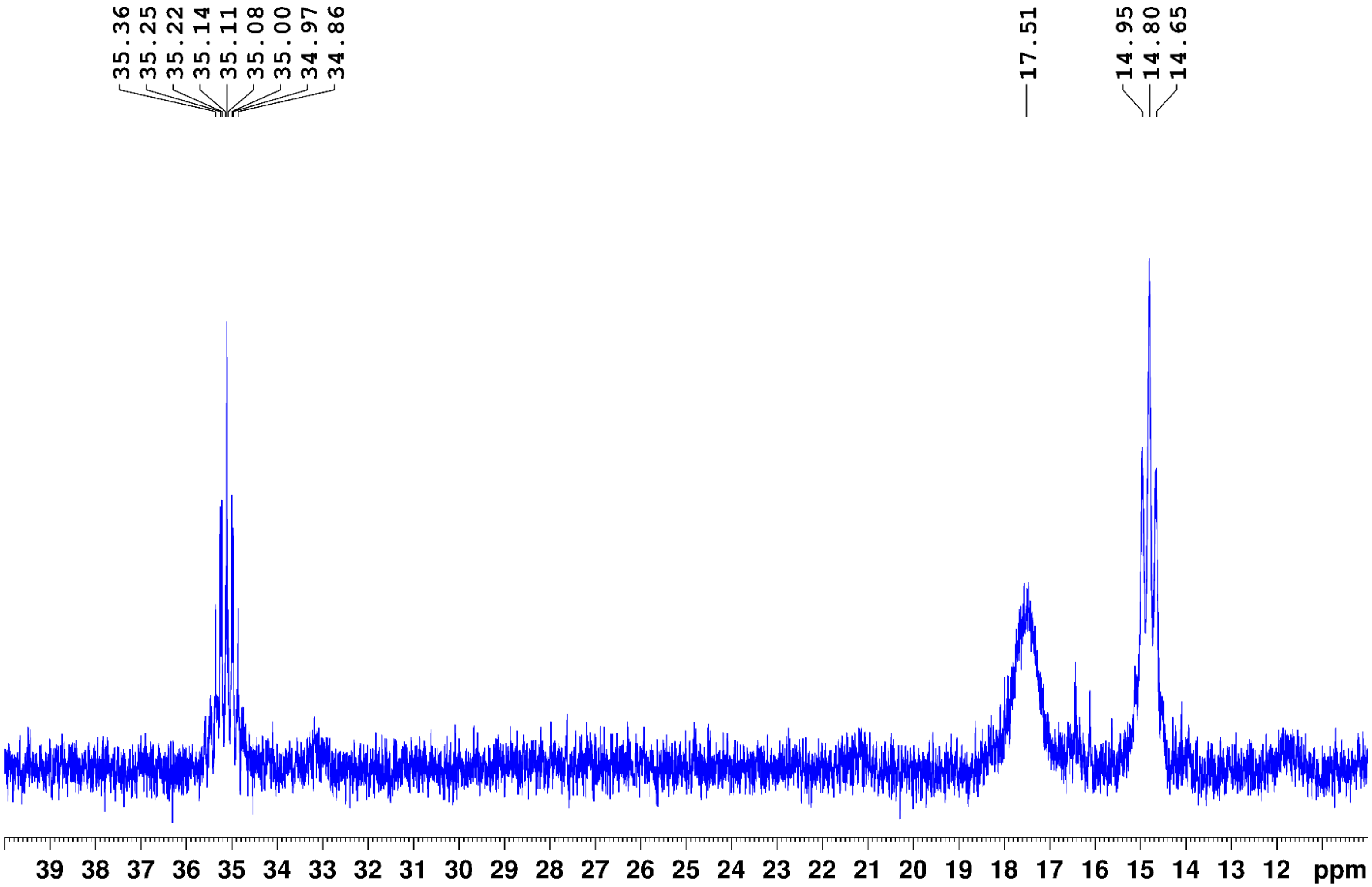

Figure S52. $^{13}C\{^{1}H\}$ NMR spectrum of $\mathbf{4^{SiCl3}}$**-HCl** in CD_2Cl_2.

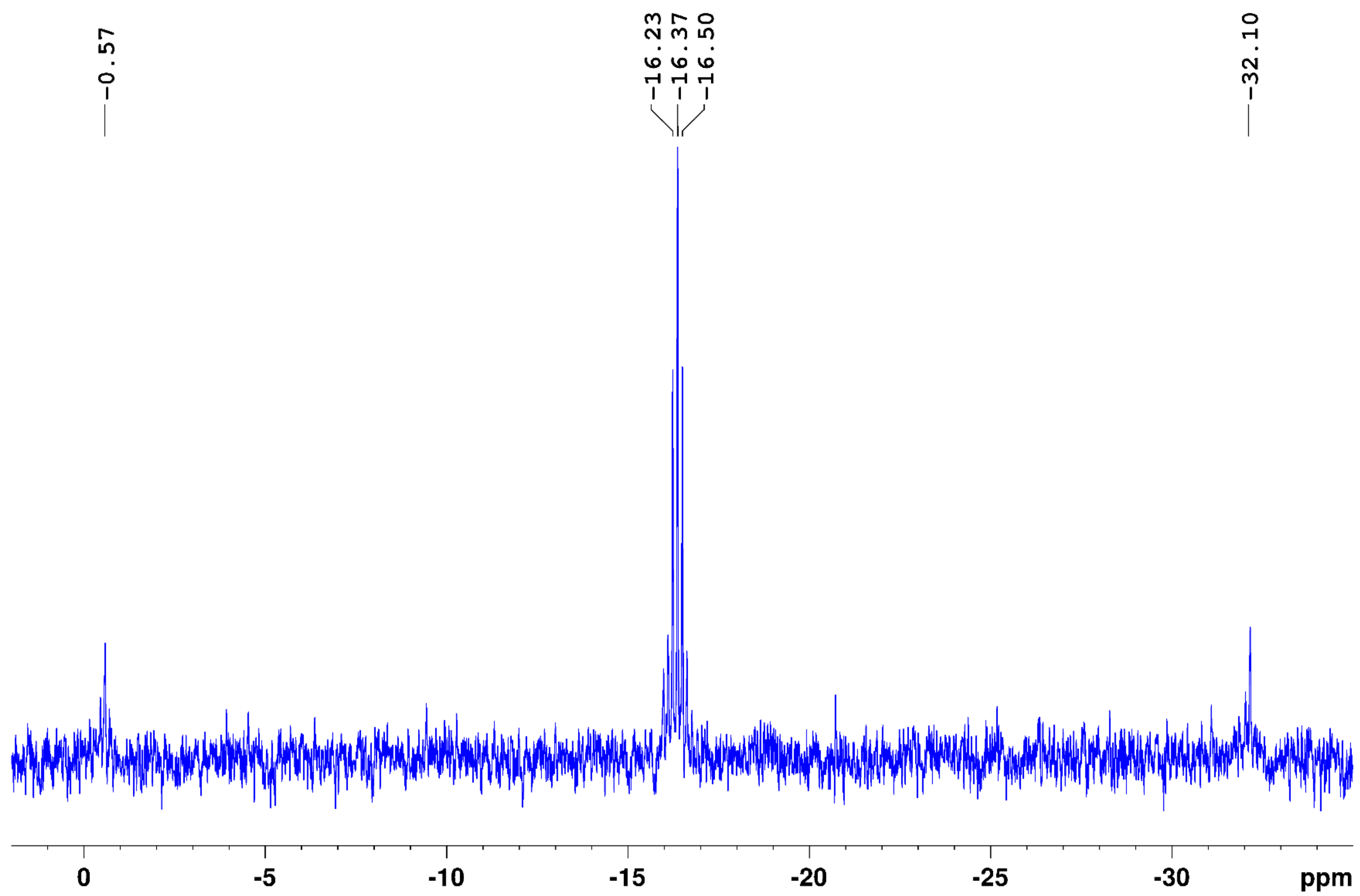

Figure S53. $^{29}Si\{^1H\}$ NMR spectrum of $\mathbf{4^{SiCl3}}$**-HCl** in CD_2Cl_2.

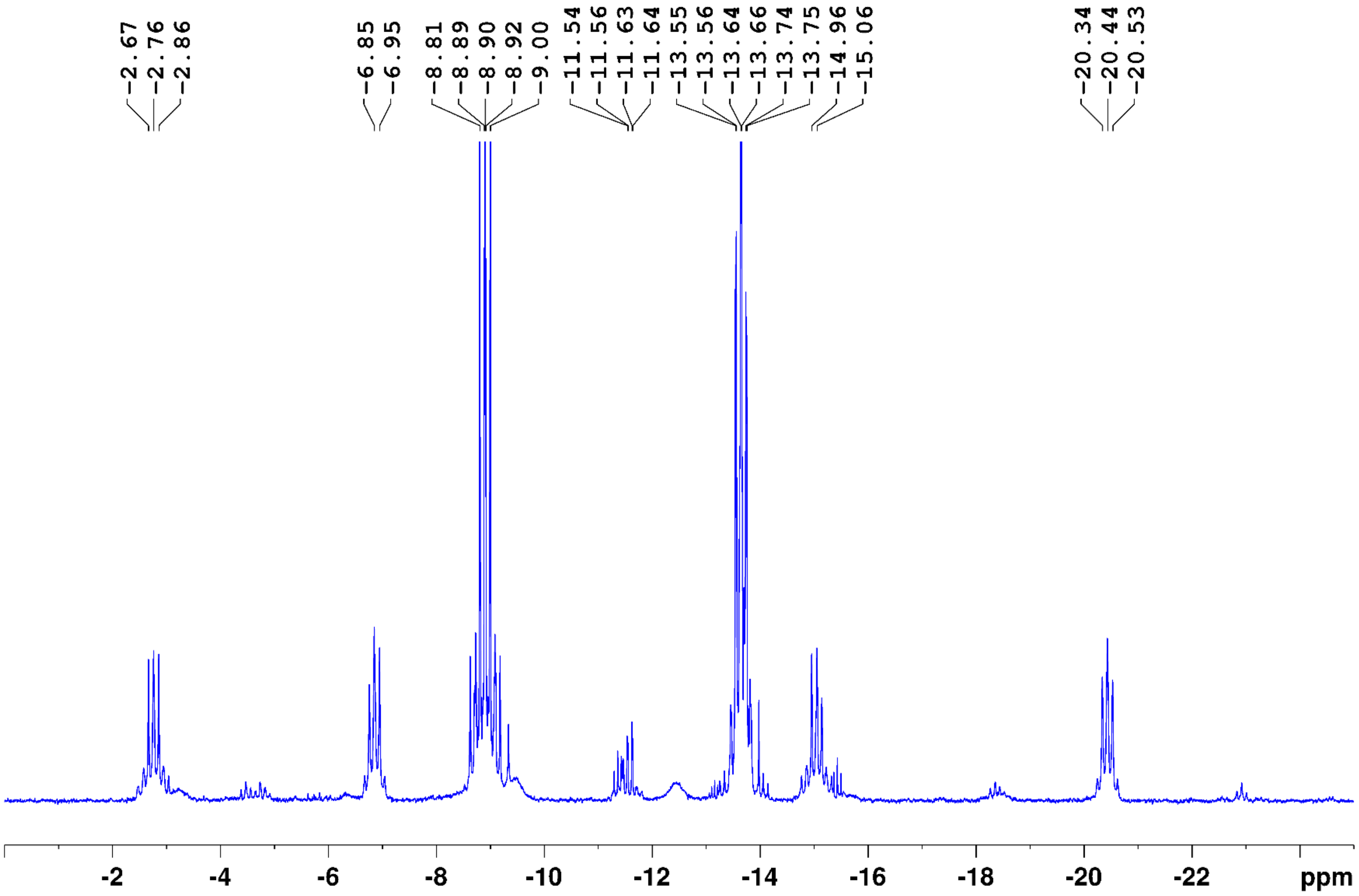

Figure S54. $^{31}P\{^{1}H\}$ NMR spectrum of **4^{SiCl3}-HCl** in CD_2Cl_2.

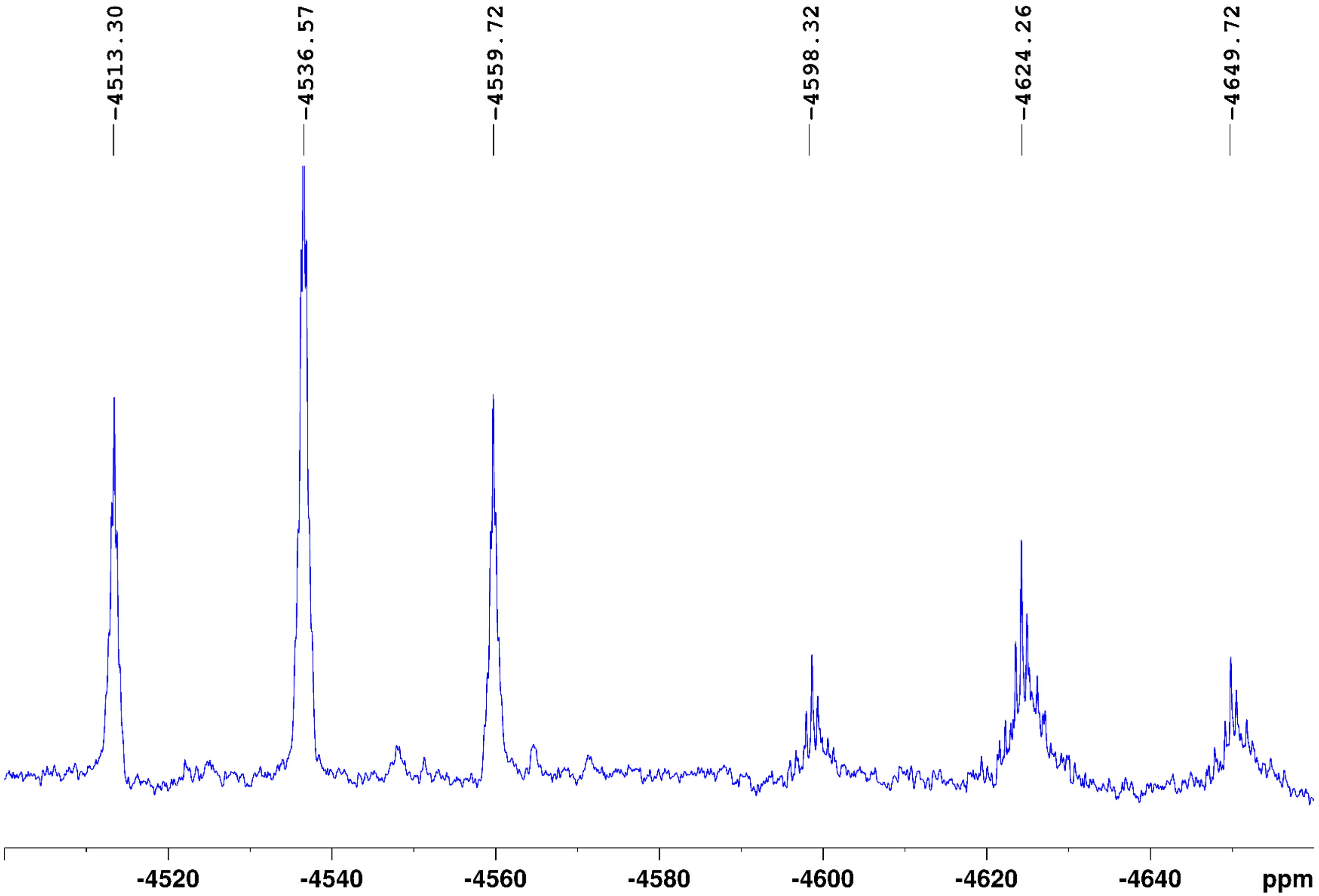

Figure S55. $^{195}Pt\{^{1}H\}$ NMR spectrum of $\mathbf{4^{SiCl3}}$**-HCl** in CD_2Cl_2.

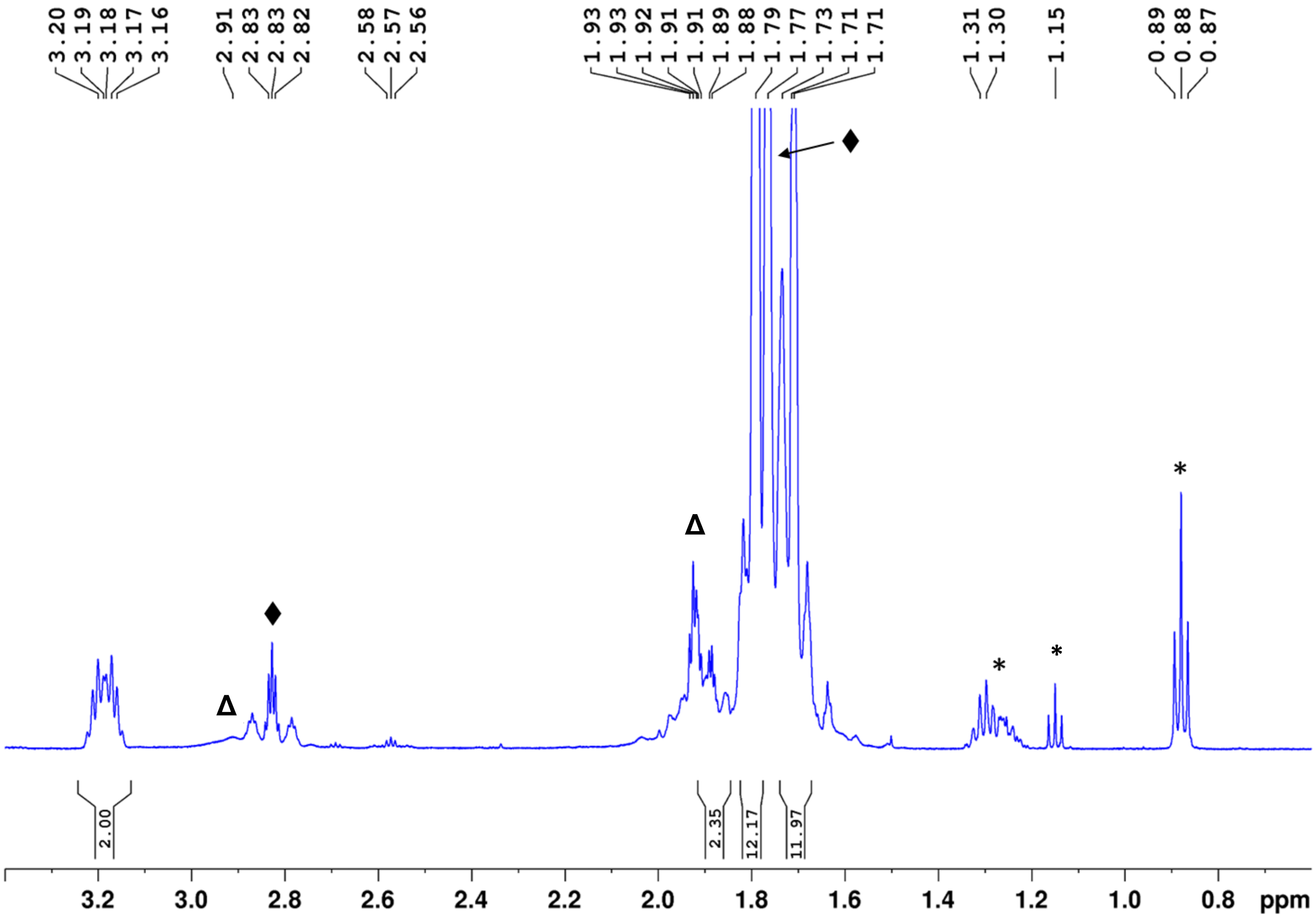

Figure S56. ^{1}H NMR spectrum of **2^{SiCl2}-Cl** in CD_2Cl_2. The additional resonances marked ♦ correspond to the decomposition product [(*μ*–dmpm)$_2$Pt$_2$Cl$_2$], those marked **Δ** to the HCl hydrolysis complex **4^{SiCl3}-HCl**, those marked * to residual pentane/hexane from recrystallisation.

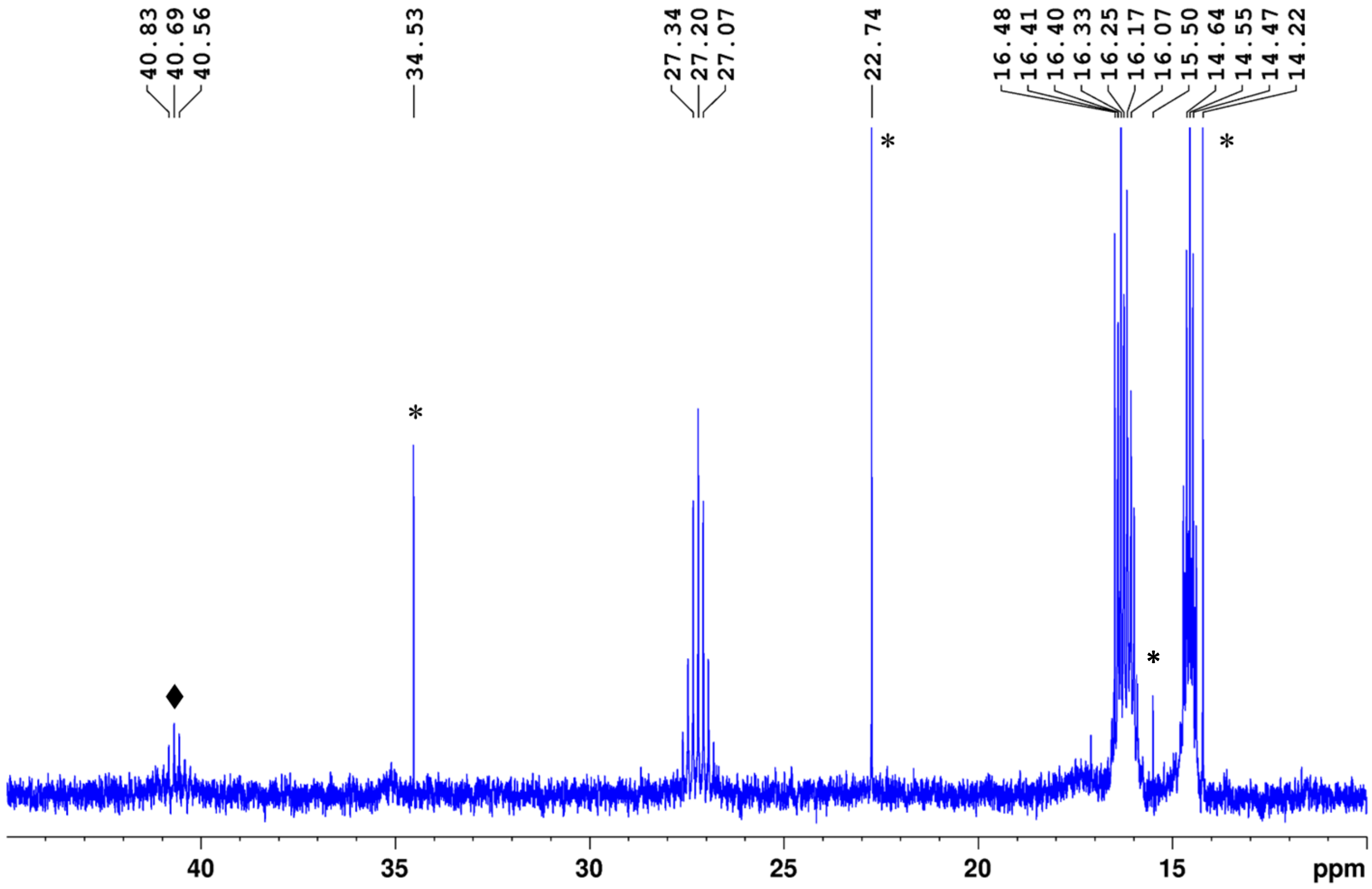

Figure S57. $^{13}C\{^1H\}$ NMR spectrum of $\mathbf{2^{SiCl2}}$**-Cl** in CD_2Cl_2. The additional resonance marked ♦ corresponds to the decomposition product [(*μ*–dmpm)$_2$Pt$_2$Cl$_2$], those marked * to residual pentane/hexane from recrystallisation.

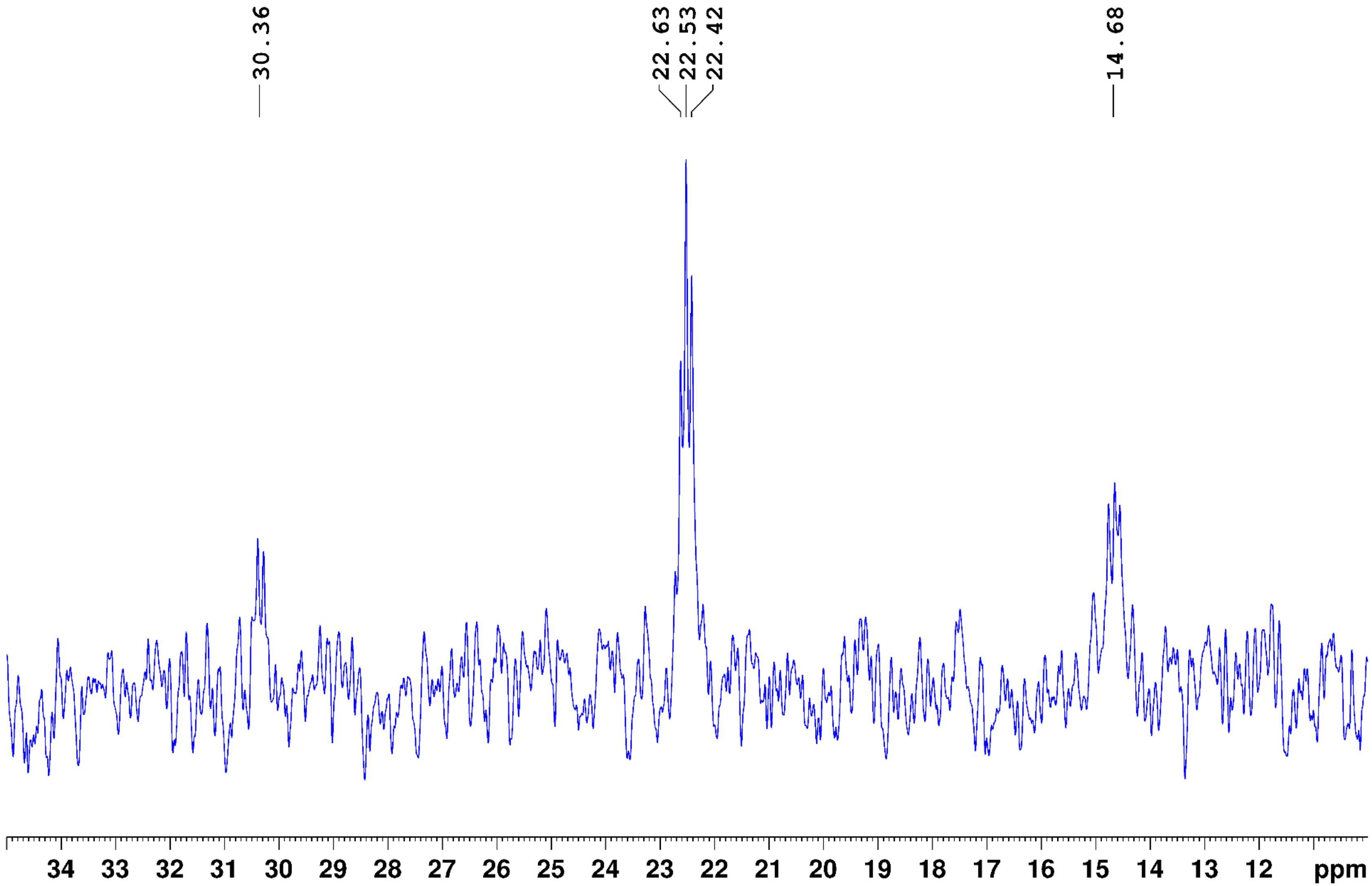

Figure S58. $^{29}Si\{^1H\}$ NMR spectrum of $\mathbf{2^{SiCl2}\text{-}Cl}$ in CD_2Cl_2.

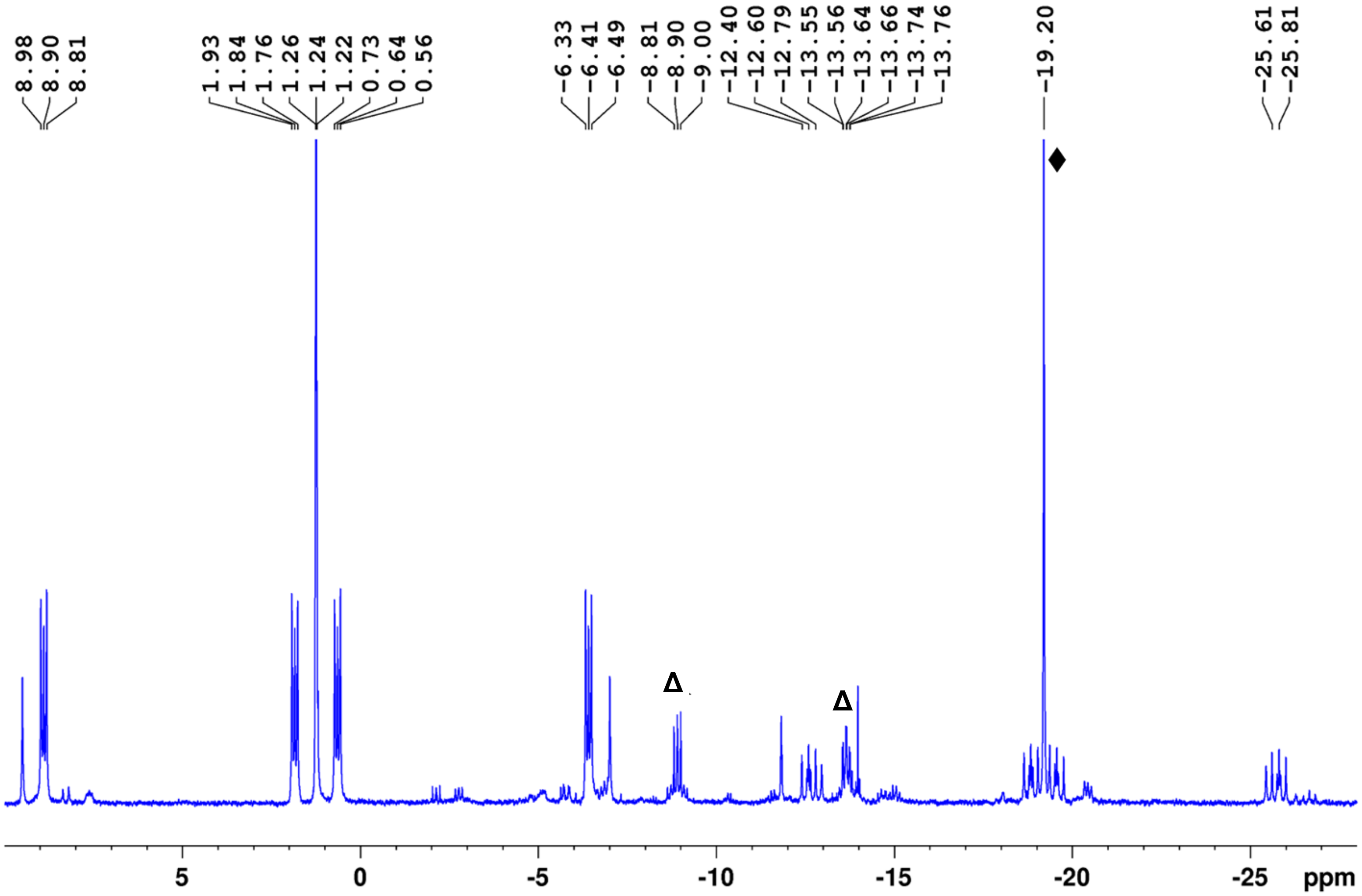

Figure S59. $^{31}P\{^1H\}$ NMR spectrum of **2^SiCl2^-Cl** in CD_2Cl_2. The additional resonances marked ♦ correspond to the decomposition product [(*μ*–dmpm)$_2$Pt$_2$Cl$_2$] (ca. 22%), those marked **Δ** to the HCl hydrolysis complex **4^SiMeCl2^-HCl** (ca. 8%).

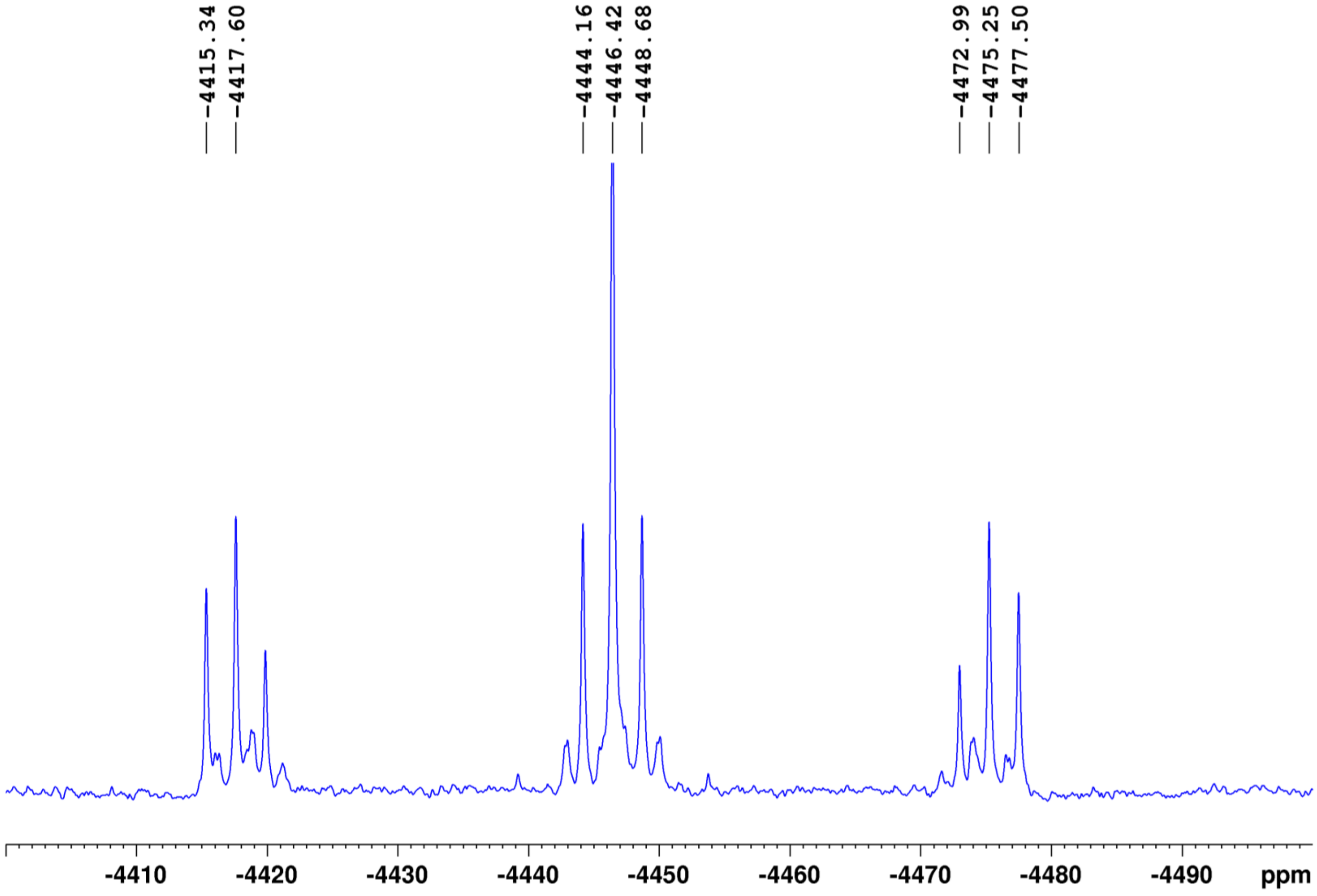

Figure S60. $^{195}Pt\{^{1}H\}$ NMR spectrum of $\mathbf{2^{SiCl2}}$**-Cl** in CD_2Cl_2.

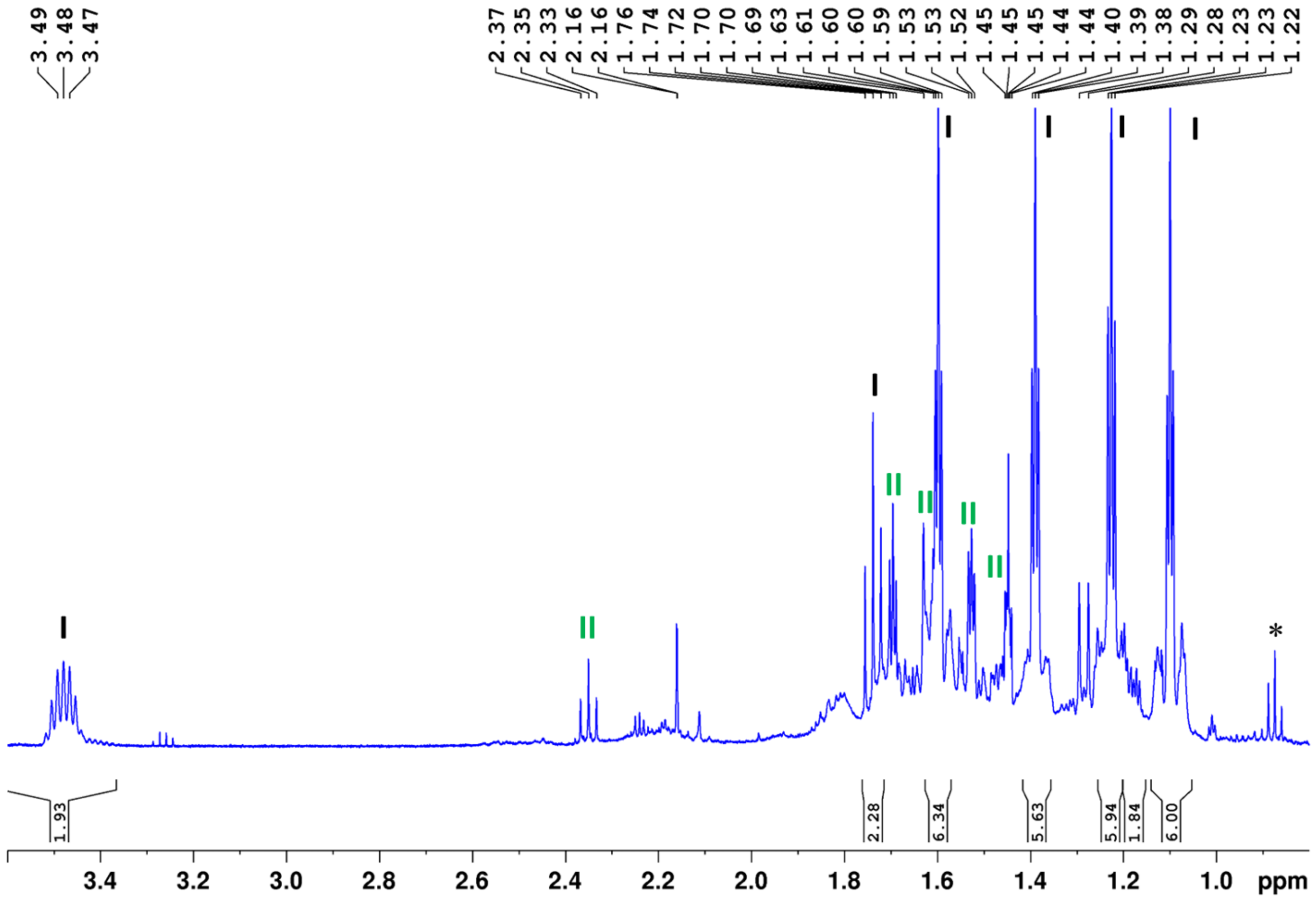

Figure S61. ^{1}H NMR spectrum of the mixture of **5^{SiCl3}-Br** (**I**, 80%) and **5^{SiCl3}-Cl** (**II**, 20%) in C_6D_6. The additional resonance marked * corresponds to residual pentane.

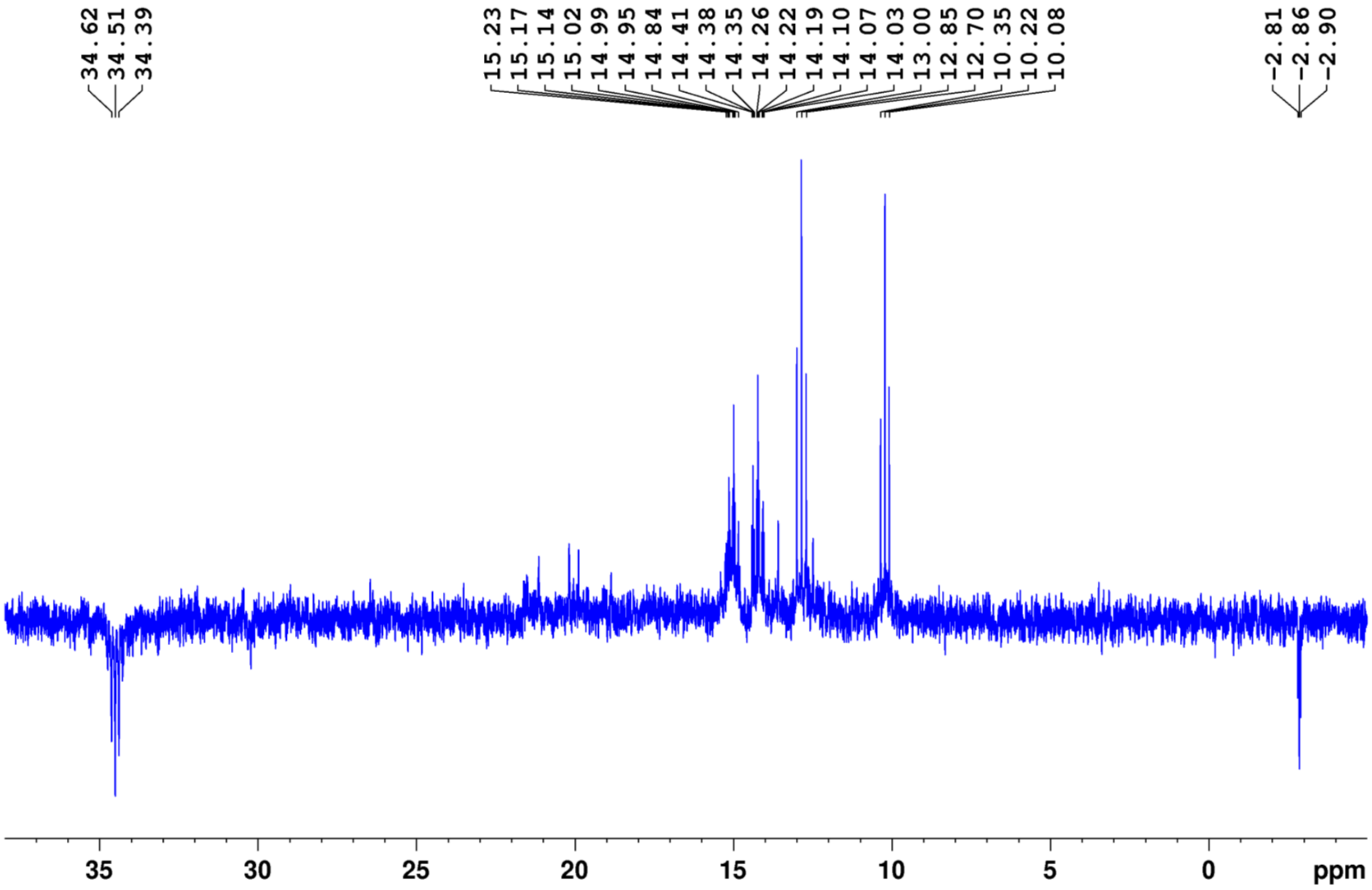

Figure S62. DEPT135-$^{13}C\{^1H\}$ NMR spectrum of **5^{SiCl3}-Br** and **5^{SiCl3}-Cl** in C_6D_6.

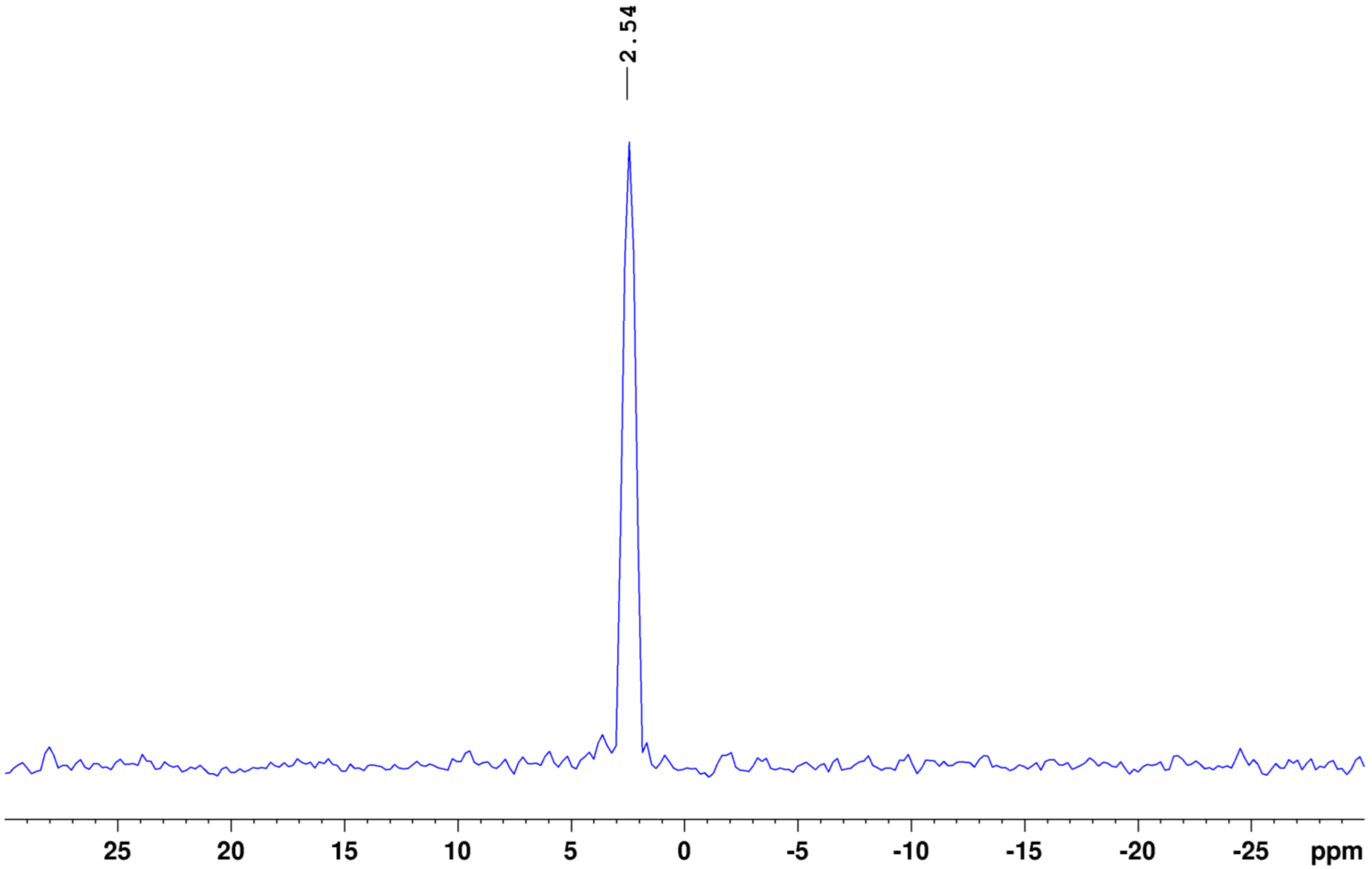

Figure S63. $^{29}Si\{^{1}H\}$ NMR spectrum of **5^{SiCl3}-Br/Cl** in C_6D_6.

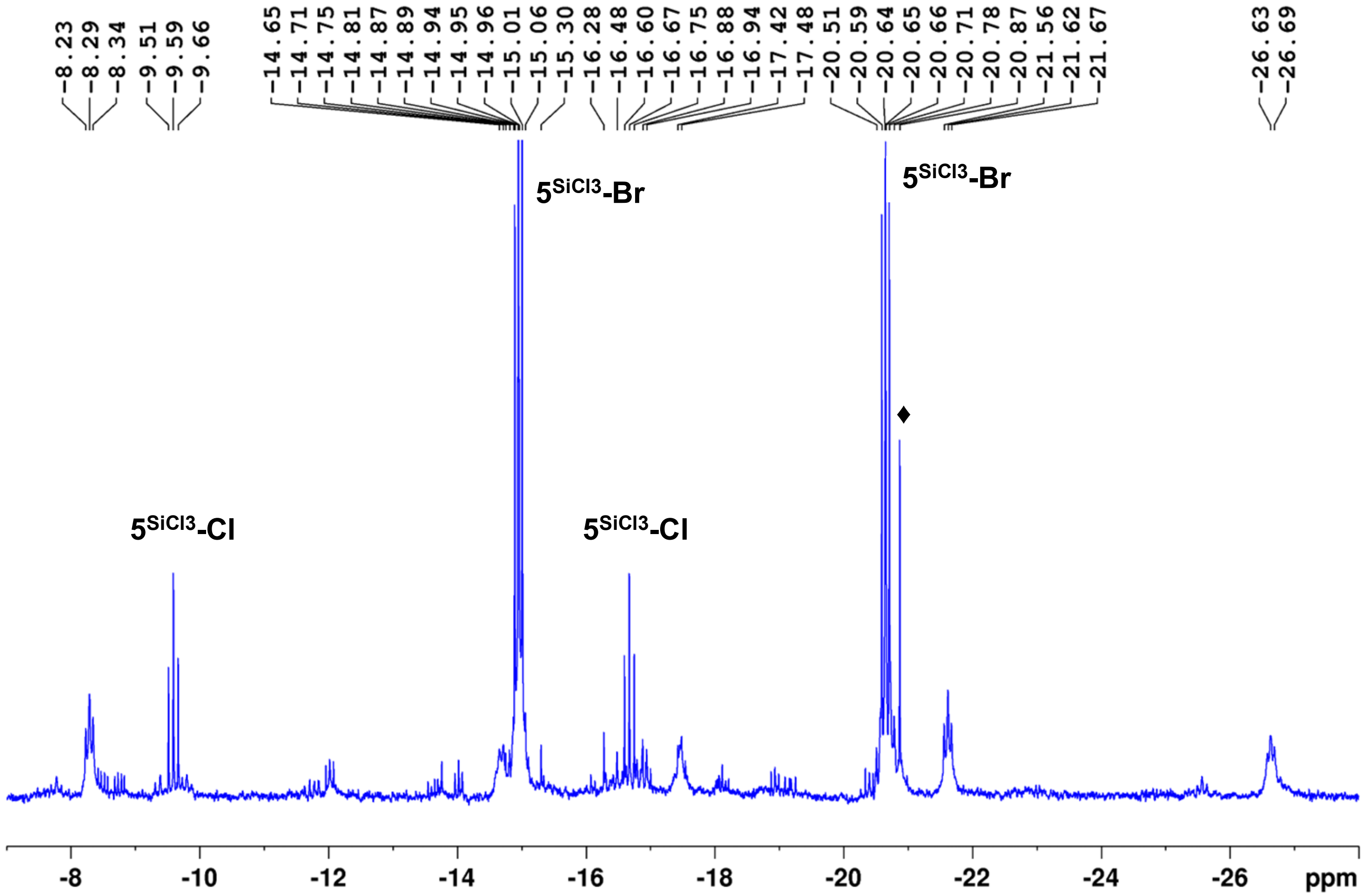

Figure S64. $^{31}P\{^{1}H\}$ NMR spectrum of **5^{SiCl3}-Br** and **5^{SiCl3}-Cl** in C_6D_6. The additional resonances marked ♦ correspond to the decomposition product [(μ–dmpm)$_2$Pt$_2$Br$_2$] (ca. 22%).

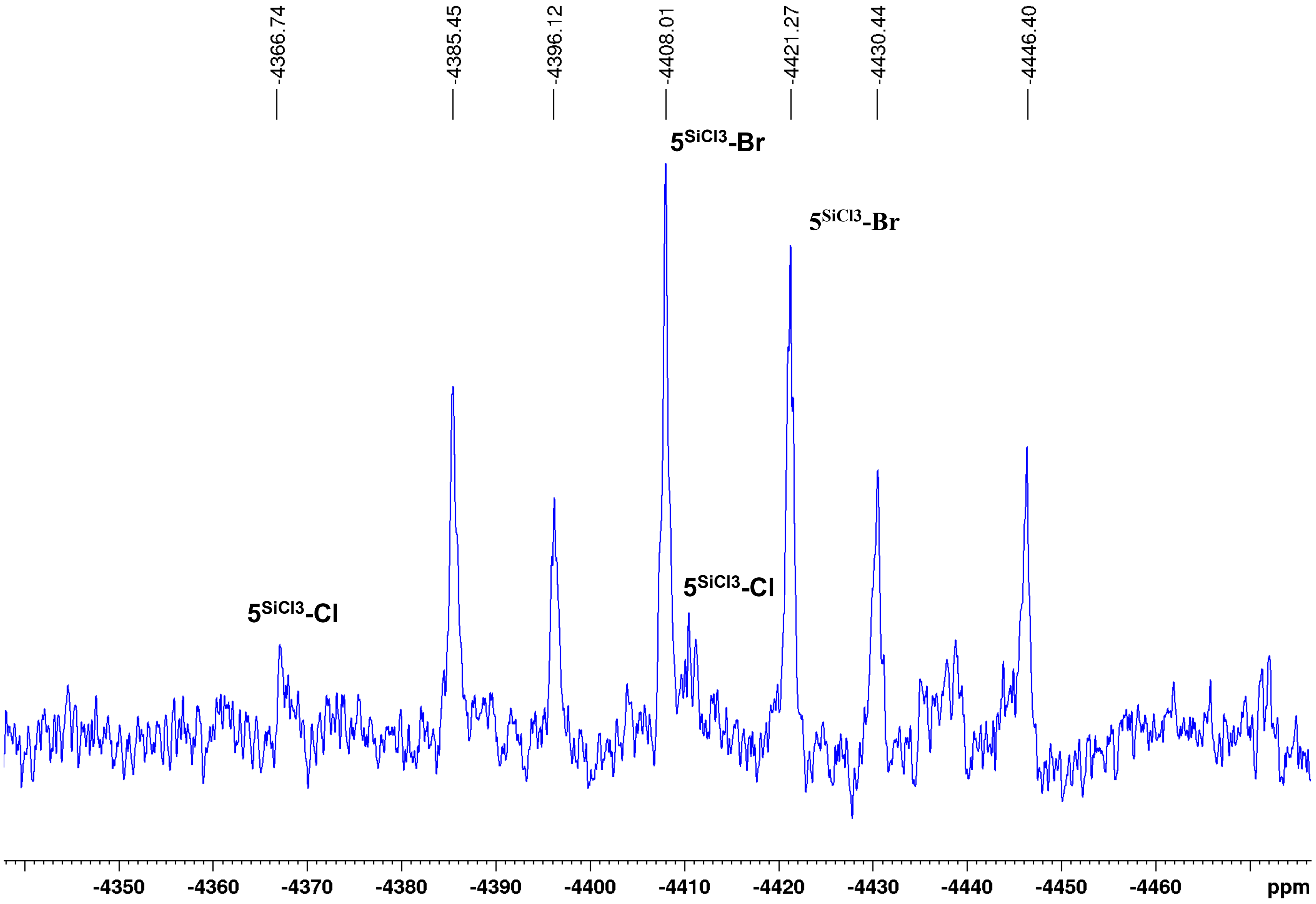

Figure S65. ^{195}Pt{^{1}H} NMR spectrum of **5^{SiCl3}-Br** and **5^{SiCl3}-Cl** in C_6D_6. The resonances for **5^{SiCl3}-Cl** were assigned by ^{1}H-^{195}Pt HMBC.

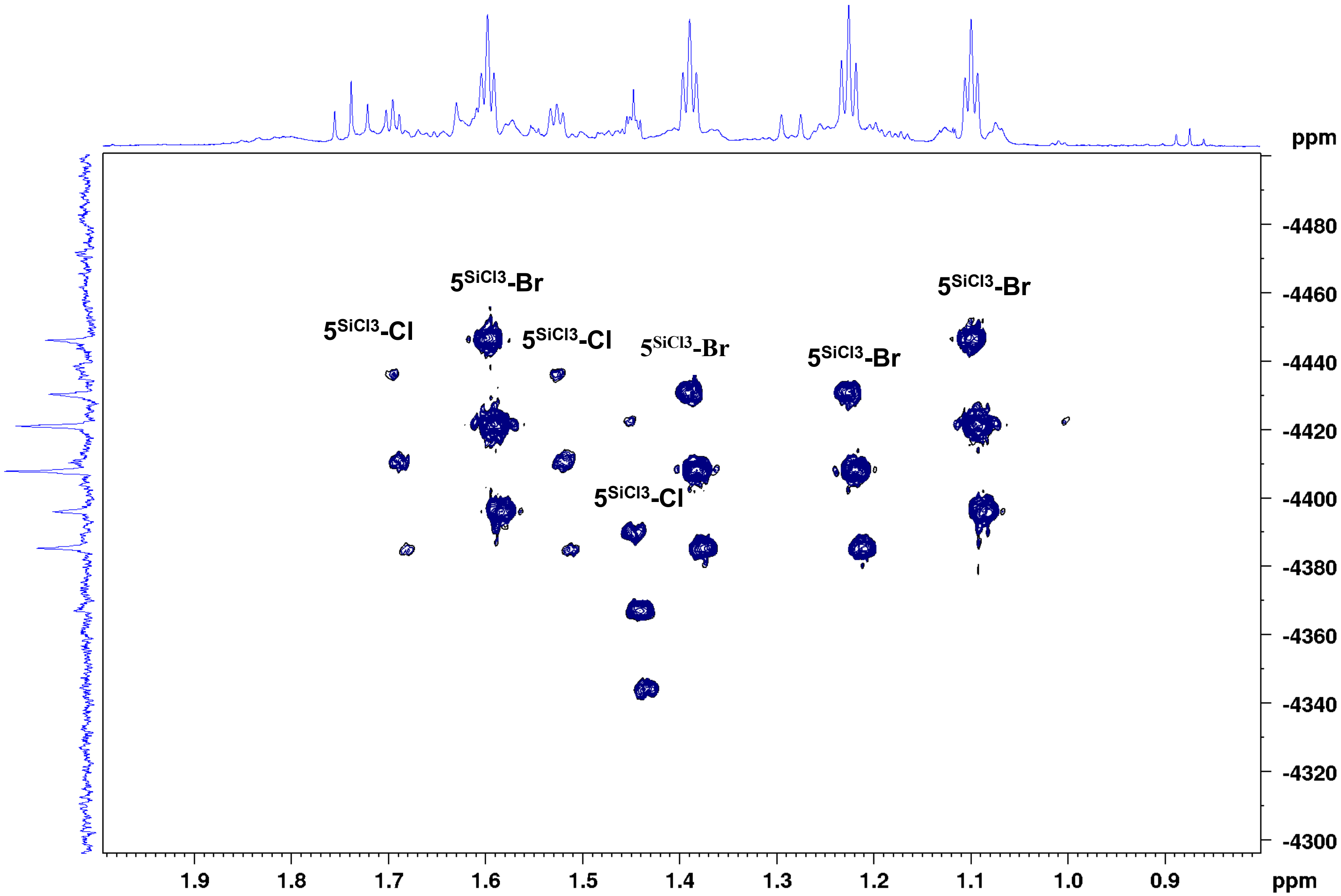

Figure S66. ^{1}H-^{195}Pt HMBC plot of **5^{SiCl3}-Br** and **5^{SiCl3}-Cl** in C_6D_6.

Crystallographic data

The crystal data of **3^{SiCl3}-Cl** was collected on a XTALAB Synergy, DualFlex, HyPix diffractometer with a hybrid pixel array detector and multi-layer mirror monochromated $Cu_{K\alpha}$ radiation. All other crystal data were collected on a Bruker D8 QUEST diffractometer equipped with a CMOS area detector and multi-layer mirror monochromated $Mo_{K\alpha}$ radiation. Structures were solved using the intrinsic phasing method,[5] refined with the SHELXL program[6] and expanded using Fourier techniques. All non-hydrogen atoms were refined anisotropically. Hydrogen atoms were included in structure factor calculations. All hydrogen atoms were assigned to idealised geometric positions, except where stated otherwise in the refinement details.

Crystallographic data have been deposited with the Cambridge Crystallographic Data Center as supplementary publication no. 2406490 (**2^{CH2}-Cl**), 2406494 (**2^{CH2}-Br**), 2406498 (**2^{CH2}-I**), 2406509 (**2^{CH2}-Br'**), 2406550 (**3^{SiMe3}-I**), 2406556 (**3^{SiMe2Cl}-Cl**), 2406558 (**3^{SiMeCl2}-Cl**), 2406564 (**3^{SiCl3}-Cl**), 2505905 (**4^{SiCl3}-HCl**), 2406843 (**2^{SiCl2}-Cl**), 2406901 (**5^{SiCl3}-Br**). These data can be obtained free of charge from The Cambridge Crystallographic Data Centre *via* www.ccdc.cam.ac.uk/data_request/cif.

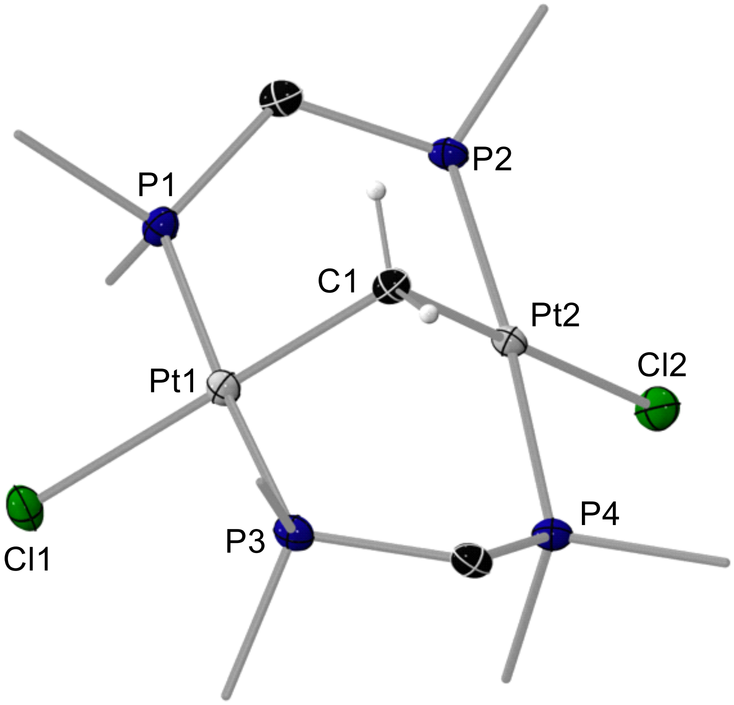

Figure S67. Solid-state structure of $\mathbf{2^{CH2}}$**-Cl**. Atomic displacement ellipsoids are represented at 50%. Ellipsoids of methyl groups and hydrogen atoms are omitted for clarity, except for methylene protons at C1.

Refinement details for $\mathbf{2^{CH2}}$-Cl: Refined as an inversion twin, with BASF = 2.8%. One reflection was omitted as an outlier (0 2 2).

Crystal data for $\mathbf{2^{CH2}}$-Cl: $C_{11}H_{30}Cl_2P_4Pt_2$, M_r = 747.31, orange plate, 0.203×0.088×0.061 mm^3, monoclinic space group *Pn*, a = 6.5571(19) Å, b = 12.974(2) Å, c = 12.042(2) Å, β = 97.096(11)°, V = 1016.6(4) Å^3, Z = 2, ρ_{calcd} = 2.441 g·cm^{-3}, μ = 14.310 mm^{-1}, F(000) = 692, T = 100(2) K, R_1 = 0.0174, wR_2 = 0.0371, Flack parameter = 0.028(8), 3974 independent reflections [$2\theta \leq 52.044°$] and 181 parameters.

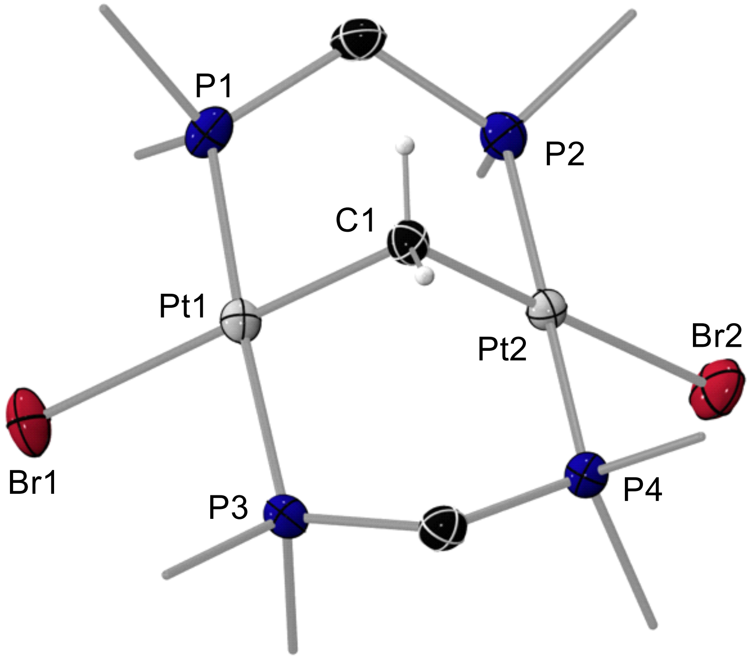

Figure S68. Solid-state structure of **2^{CH2}-Br**. Atomic displacement ellipsoids are represented at 50%. Ellipsoids of methyl groups and hydrogen atoms are omitted for clarity, except for methylene protons at C1.

Refinement details for 2^{CH2}-Br: Refined as an inversion twin, with BASF = 1.2%. The asymmetric unit contains two benzene molecules, one of which was modelled as twofold disordered (RESI 2/3 BENZ) in a 47:53 ratio. All benzene solvent rings were idealised with AFIX 66 and their ADPs restrained to similarity with SIMU 0.004.

Crystal data for 2^{CH2}-Br: $C_{11}H_{30}Br_2P_4Pt_2{\cdot}(C_6H_6)_2$, M_r = 992.44, red block, 0.106×0.103×0.034 mm^3, orthorhombic space group $P2_12_12_1$, a = 8.8989(15) Å, b = 14.2185(17) Å, c = 24.703(5) Å, V = 3125.6(9) Å^3, Z = 4, ρ_{calcd} = 2.109 g·cm^{-3}, μ = 11.714 mm^{-1}, F(000) = 1864, T = 100(2) K, R_1 = 0.0309, wR_2 = 0.0437, Flack parameter = 0.012(7), 9541 independent reflections [$2\theta \leq 61.038°$] and 308 parameters.

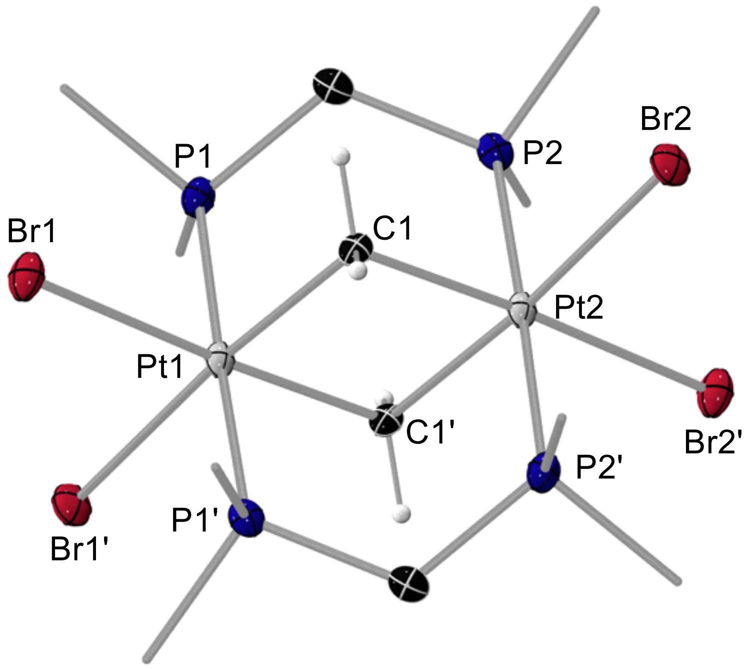

Figure S69. Solid-state structure of **2^{CH2}-Br'**. Atomic displacement ellipsoids are represented at 50%. Ellipsoids of methyl groups and hydrogen atoms are omitted for clarity, except for methylene protons at C1.

Refinement details for 2^{CH2}-Br': none.

Crystal data for 2^{CH2}-Br': $C_{12}H_{32}Br_4P_4Pt_2$, M_r = 1010.07, yellow plate, 0.179×0.133×0.055 mm^3, monoclinic space group $C2/c$, a = 18.672(4) Å, b = 10.955(4) Å, c = 13.051(5) Å, α = 90°, β = 117.21(3)°, γ = 90°, V = 2374.2(14) Å^3, Z = 4, ρ_{calcd} = 2.826 g·cm^{-3}, μ = 18.768 mm^{-1}, F(000) = 1840, T = 100(2) K, R_1 = 0.0710, wR_2 = 0.0801, 5339 independent reflections [$2\theta \leq 73.042°$] and 104 parameters.

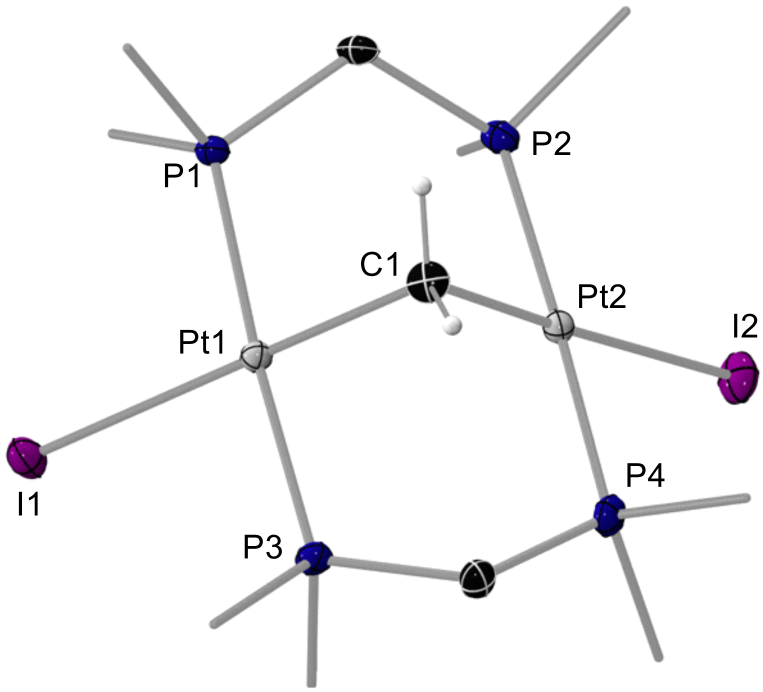

Figure S70. Solid-state structure of $\mathbf{2^{CH2}}$**-I**. Atomic displacement ellipsoids are represented at 50%. Ellipsoids of methyl groups and hydrogen atoms are omitted for clarity, except for methylene protons at C1.

Refinement details for $\mathbf{2^{CH2}}$-I: Refined as an inversion twin, with BASF = 4.5%. One PMe_2 group was modelled as twofold disordered (RESI 11/12 PMe2) in a 71:29 ratio. ADPs within the disorder were restrained to similarity with SIMU 0.003 and approximated to isotropy with ISOR 0.005.

Crystal data for $\mathbf{2^{CH2}}$-I: $C_{11}H_{30}I_2P_4Pt_2{\cdot}C_6H_6$, M_r = 1008.32, yellow plate, 0.130×0.084×0.036 mm^3, monoclinic space group *Cc*, a = 13.9003(12) Å, b = 16.8782(15) Å, c = 12.3088(10) Å, β = 109.790(3)°, V = 2717.2(4) Å^3, Z = 4, ρ_{calcd} = 2.465 g·cm^{-3}, μ = 12.795 mm^{-1}, $F(000)$ = 1840, T = 100(2) K, R_1 = 0.0240, wR_2 = 0.0436, Flack parameter = 0.045(5), 5927 independent reflections [$2\theta \leq 54.212°$] and 274 parameters.

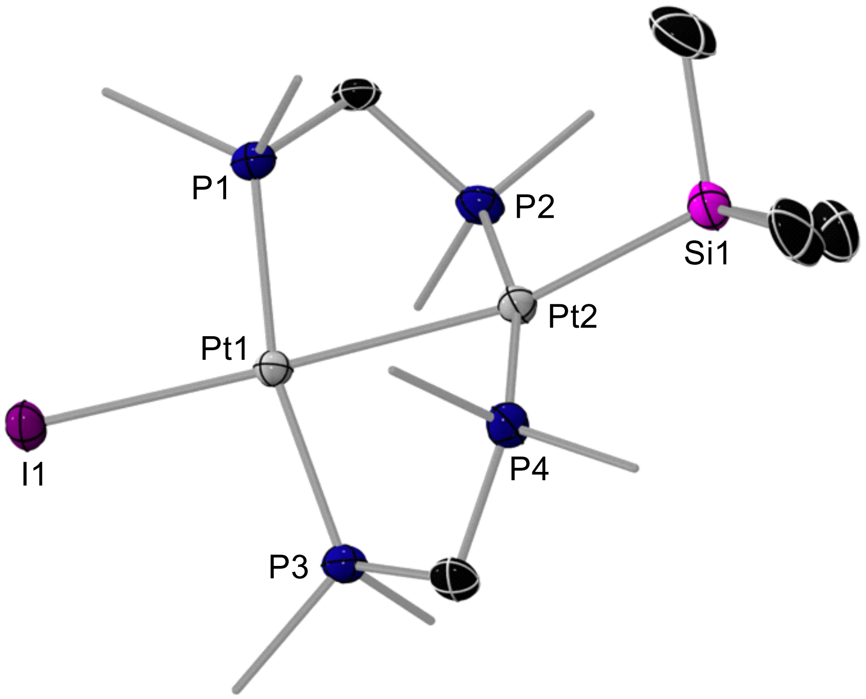

Figure S71. Solid-state structure of $\mathbf{3^{SiMe3}}$**-I**. Atomic displacement ellipsoids are represented at 50%. Ellipsoids of dmpm methyl groups and hydrogen atoms are omitted for clarity.

Refinement details for $\mathbf{3^{SiMe3}}$-I: Some reflections were removed from refinement as outliers. The unit cell contains two rotationally disordered half benzene molecules, one positioned on an inversion center and the other bisected by a C_2 axis, which were modelled with PARTs -1 10.5 and idealised rings (AFIX 6). ADPs within these disorders were restrained to similarity with SIMU 0.005 and approximated to isotropy with ISOR 0.01. The unit cell contains partially occupied and highly disordered pentane molecules positioned around symmetry operators, which could not be modelled adequately and have been treated as a diffuse contribution to the overall scattering without specific atom positions by the Platon program Squeeze.[7] 194 electrons were thus removed from the unit cell, corresponding to 4.6 pentane molecules, i.e. ca. 0.5 molecules of pentane per asymmetric unit.

Crystal data for $\mathbf{3^{SiMe3}}$-I: $C_{13}H_{34}IP_4Pt_2Si\cdot(C_6H_6)\cdot[(C_5H_{12})_{0.5}]_{squeezed}$, $M_r = 973.66$, yellow needle , 0.301×0.096×0.078 mm^3, monoclinic space group $C2/c$, $a = 32.531(2)$ Å, $b = 16.2293(15)$ Å, $c = 12.3818(11)$ Å, $\beta = 94.934(3)°$, $V = 6512.8(10)$ Å^3, $Z = 8$, $\rho_{calcd} = 1.986$ g·cm^{-3}, $\mu = 9.768$ mm^{-1}, $F(000) = 3496$, $T = 100(2)$ K, $R_1 = 0.0512$, $wR_2 = 0.0793$, 6666 independent reflections [$2\theta \leq 52.762°$] and 255 parameters.

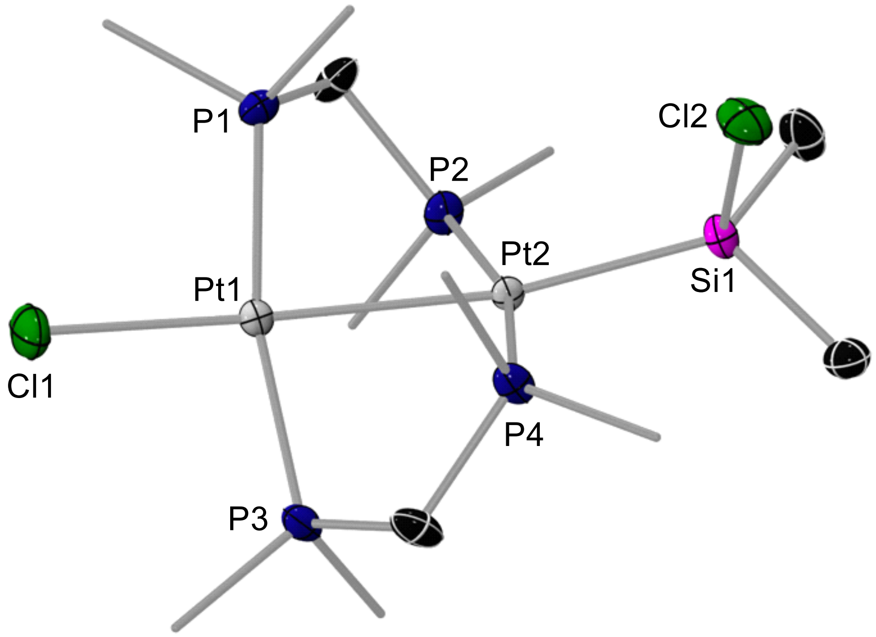

Figure S72. Solid-state structure of **$3^{SiMe2Cl}$-Cl**. Atomic displacement ellipsoids are represented at 50%. Ellipsoids of dmpm methyl groups and hydrogen atoms are omitted for clarity.

Refinement details for $3^{SiMe2Cl}$-Cl: The asymmetric unit contains a benzene solvent molecule, which was modelled as twofold rotationally disordered (RESI 3/33 BENZ) in a 78:22 ratio. The benzene rings within the disorder were idealised with AFIX 6. ADPs within this disorder were restrained to similarity with SIMU 0.005. The $SiMe_2Cl$ substituent at PT1 was modelled as twofold rotationally disordered (RESI 2/22 SI) in a 73:27 ratio in C1 and CL1. The SI1_1–C1_2 and SI1_1–C1_22 distances were restrained to similarity using SADI.

Crystal data for $3^{SiMe2Cl}$-Cl: $C_{12}H_{34}Cl_2P_4Pt_2Si\cdot C_6H_6$, $M_r = 869.55$, colourless block, $0.229\times0.178\times0.142$ mm^3, monoclinic space group $P2_1/n$, $a = 9.639(4)$ Å, $b = 29.672(11)$ Å, $c = 9.783(4)$ Å, $\beta = 94.077(13)°$, $V = 2791(2)$ Å^3, $Z = 4$, $\rho_{calcd} = 2.070$ g·cm^{-3}, $\mu = 10.483$ mm^{-1}, $F(000) = 1648$, $T = 100(2)$ K, $R_1 = 0.0416$, $wR_2 = 0.0740$, 5691 independent reflections [$2\theta \leq 52.744°$] and 332 parameters.

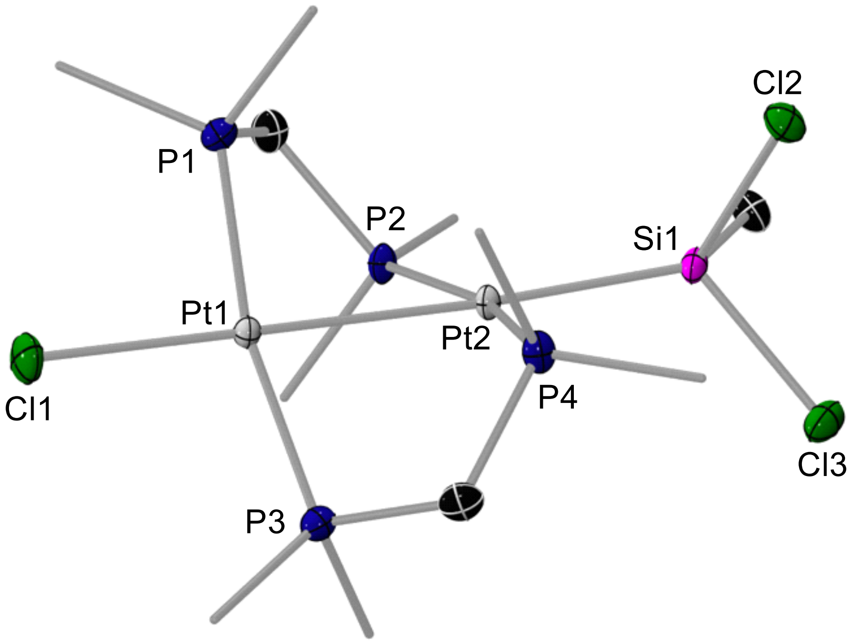

Figure S73. Solid-state structure of $\mathbf{3^{SiMeCl2}}$**-Cl**. Atomic displacement ellipsoids are represented at 50%. Ellipsoids of dmpm methyl groups and hydrogen atoms are omitted for clarity.

Refinement details for $\mathbf{3^{SiMeCl2}}$-Cl: The asymmetric unit contains one molecule of *o*-DFB, modelled as threefold rotationally disordered (RESI 21/22/23 DFB) in a 54:11:35 ratio using SUMP and there FVAR. Benzene rings within the disorder were idealised with AFIX 66, ADPs were restrained to similarity with SIMU 0.01, and 1,2 and 1,3 distances with SAME.

Crystal data for $\mathbf{3^{SiMeCl2}}$-Cl: $C_{11}H_{31}Cl_3P_4Pt_2Si{\cdot}C_6H_4F_2$, M_r = 925.95, yellow block, 0.128×0.114×0.047 mm^3, monoclinic space group $P2_1/n$, a = 10.199(3) Å, b = 9.446(2) Å, c = 29.217(8) Å, β = 90.426(10)°, V = 2814.7(12) Å^3, Z = 4, ρ_{calcd} = 2.185 g·cm^{-3}, μ = 10.503 mm^{-1}, F(000) = 1744, T = 100(2) K, R_1 = 0.0370, wR_2 = 0.0712, 5547 independent reflections [$2\theta \leq 52.04°$] and 382 parameters.

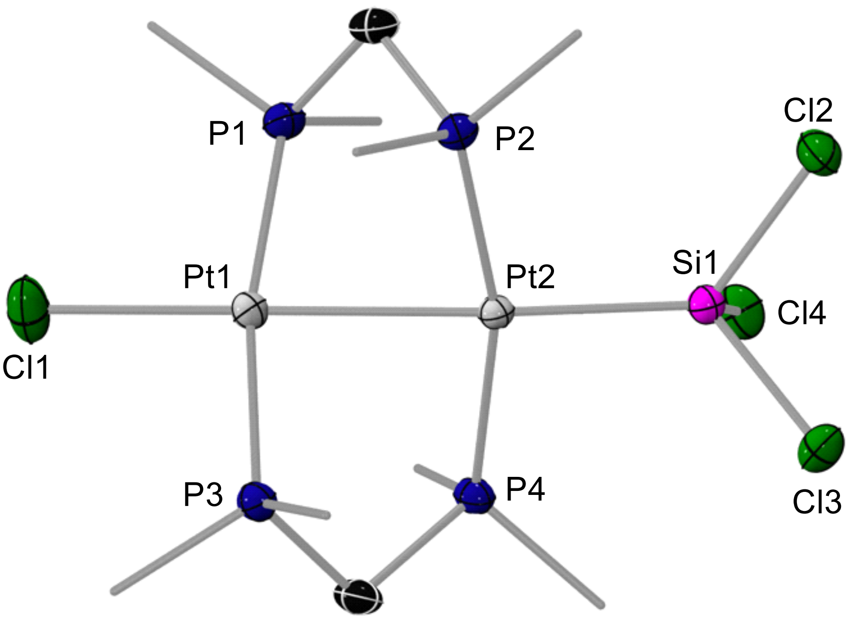

Figure S74. Solid-state structure of **3^{SiCl3}-Cl**. Atomic displacement ellipsoids are represented at 50%. Ellipsoids of methyl groups and hydrogen atoms are omitted for clarity.

Refinement details for 3^{SiCl3}-Cl: The asymmetric unit contains one benzene molecule, which was modelled as twofold rotationally disordered (RESI 21/22 BENZ) in a 53:47 ratio. The benzene rings within the disorder were idealised with AFIX 66 and ADPs restrained to similarity with AFIX 0.01.

Crystal data for 3^{SiCl3}-Cl: $C_{10}H_{28}Cl_4P_4Pt_2Si{\cdot}C_6H_6$, M_r = 910.38, colourless block, 0.200×0.050×0.030 mm^3, monoclinic space group $P2_1/n$, a = 9.58710(10) Å, b = 29.4441(2) Å, c = 9.73830(10) Å, β = 94.3660(10)°, V = 2740.98(4) Å^3, Z = 4, ρ_{calcd} = 2.206 g·cm^{-3}, μ = 25.058 mm^{-1}, F(000) = 1712, T = 100(2) K, R_1 = 0.0188, wR_2 = 0.0440, 5026 independent reflections [$2\theta \leq 136.5°$] and 283 parameters.

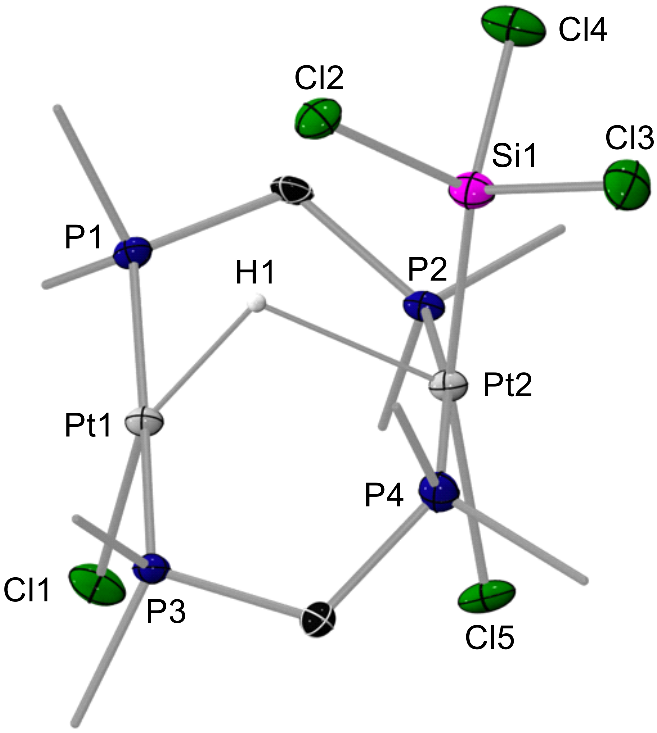

Figure S75. Solid-state structure of **4^{SiCl3}-HCl**. Atomic displacement ellipsoids are represented at 50%. Ellipsoids of methyl groups and hydrogen atoms are omitted for clarity, except for bridging hydride H1.

Refinement details for 4^{SiCl3}-HCl: The bridging hydride H1 was detected in the inverse Fourier map and freely refined.

Crystal data for 4^{SiCl3}-HCl: $C_{10}H_{29}Cl_5P_4Pt_2Si$, M_r = 204.41, yellow plate, 0.264×0.103×0.072 mm^3, monoclinic space group $P2_1/c$, a = 16.867(4) Å, b = 9.601(3) Å, c = 16.707(4) Å, β = 116.809(8)°, V = 2414.9(11) Å^3, Z = 17, ρ_{calcd} = 2.389 g·cm^{-3}, μ = 12.435 mm^{-1}, F(000) = 1616, T = 100(2) K, R_1 = 0.0526, wR_2 = 0.0944, 4752 independent reflections [$2\theta \leq 52.04°$] and 210 parameters.

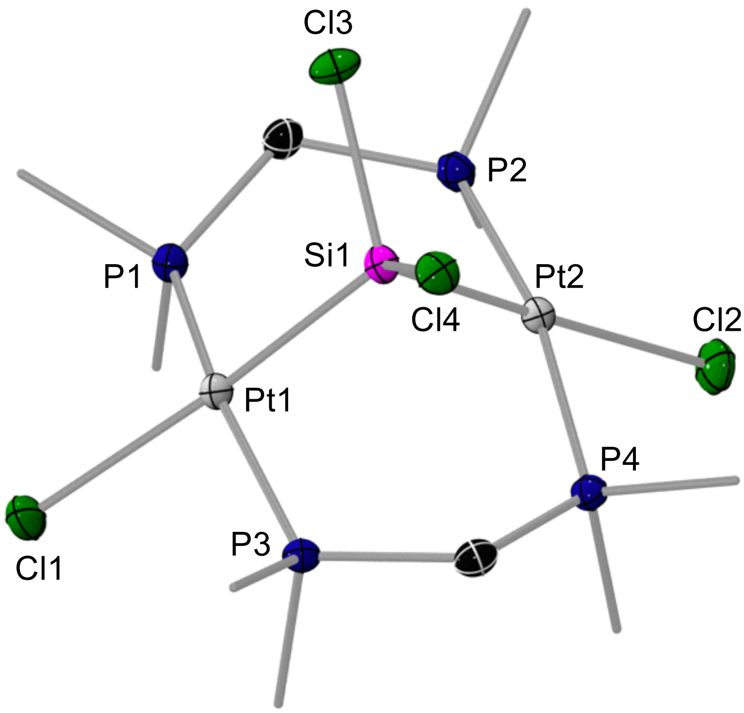

Figure S76. Solid-state structure of **2^{SiCl2}-Cl**. Atomic displacement ellipsoids are represented at 50%. Ellipsoids of methyl groups and hydrogen atoms are omitted for clarity.

Refinement details for 2^{SiCl2}-Cl: The large residual electron density (7.12 e Å^{-3} at 0.89 Å from Pt2) results from a second unidentified diplatinum complex. However, as this byproduct represents less than 2% of the sample, attempts to model the disorder failed. The data may only serve as proof of connectivity for **7** and may not be discussed in detail.

Crystal data for 2^{SiCl2}-Cl: $C_{13}H_{31}Cl_4P_4Pt_2Si$, M_r = 871.33, green block, 0.113×0.104×0.066 mm^3, monoclinic space group $P2_1/c$, a = 16.205(3) Å, b = 12.4401(17) Å, c = 12.880(3) Å, β = 103.351(9)°, V = 2526.3(8) Å^3, Z = 4, ρ_{calcd} = 2.291 g·cm^{-3}, μ = 11.785 mm^{-1}, F(000) = 1628, T = 100(2) K, R_1 = 0.0318, wR_2 = 0.0752, 4976 independent reflections [2θ≤52.044°] and 226 parameters.

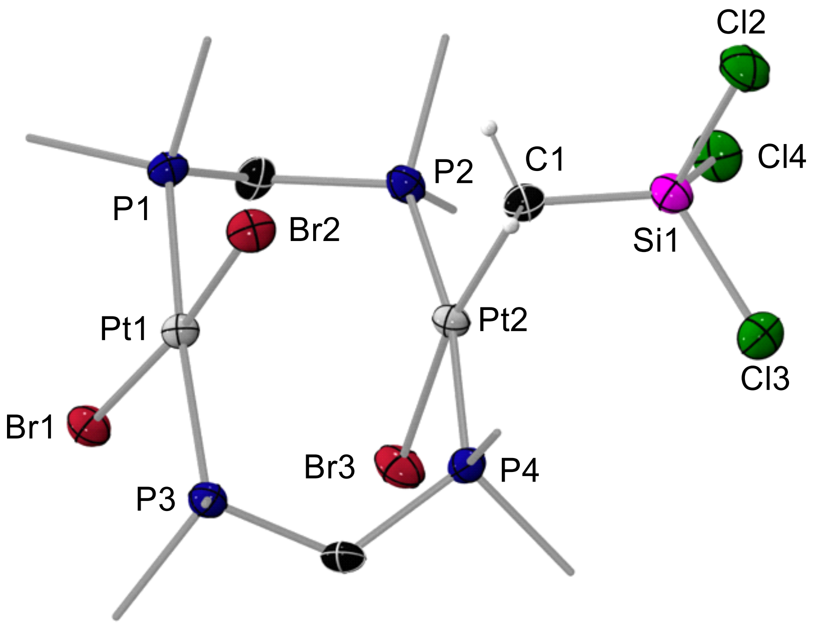

Figure S77. Solid-state structure of **5^{SiCl3}-Br**. Atomic displacement ellipsoids are represented at 50%. Ellipsoids of methyl groups and hydrogen atoms are omitted for clarity, except for methylene protons at C1.

Refinement details for 5^{SiCl3}-Br: Two low-resolution reflections, that were affected by beamstop, were removed from refinement: (-1 2 0) and (0 2 1). The unit cell contains pentane molecules, disordered and positioned around symmetry operators, which could not be adequately modelled and have been treated as a diffuse contribution to the overall scattering without specific atom positions by the Platon program Squeeze.[7] 393 electrons were thus removed from the unit cell, corresponding to 9.4 pentane molecules, i.e. ca. 0.5 per asymmetric unit.

Crystal data for 5^{SiCl3}-Br: $C_{11}H_{30}Br_3Cl_3P_4Pt_2Si\cdot[(C_5H_{10})_{0.5}]_{squeezed}$, M_r = 1085.64, colourless block, 0.159×0.143×0.089 mm^3, hexagonal space group $R\bar{3}$:H, a = 36.1930(9) Å, b = 36.1930(9) Å, c = 11.8809(5) Å, V = 13478.1(9) Å^3, Z = 18, ρ_{calcd} = 3.210 g·cm^{-3}, μ = 18.476 mm^{-1}, F(000) = 8676, T = 100(2) K, R_1 = 0.0458, wR_2 = 0.0709, 6624 independent reflections [$2\theta \leq 54.236°$] and 225 parameters.

Computational details

Optimisations and reaction coordinates

All calculations were performed using the Amsterdam Density Functional (ADF, version 2023.1) program as implemented in the Amsterdam Modeling Suite (AMS, version 2023.1).[8] All stationary points and energies were obtained using relativistic, dispersion-corrected density functional theory computations at the ZORA-BLYP-D3(BJ)/TZ2P level (see Cartesian coordinate list below). This approach comprises the BLYP level of the generalised gradient approximation (GGA), the exchange functional developed by Becke (B), and the GGA correlation functional developed by Lee, Yang, and Parr (LYP).[9] In addition, nonlocal dispersion interactions have been accounted for by the empirical DFT-D3(BJ) correction developed by Grimme and co-workers, which contains the damping function proposed by Becke and Johnson.[10] Scalar relativistic effects are accounted for using the zeroth-order regular approximation (ZORA).[11] This level has been proven to accurately describe bond activation by Group 10 metals[12] and bond energies of metal–metal complexes.[13] Solvation in benzene was simulated using the conductor-like screening model (COSMO).[14] Molecular orbitals (MO) were expanded into a large, uncontracted set of Slater-type orbitals (STOs): TZ2P.[15] This basis set is of triple-ζ quality for all atoms, augmented with polarisation functions, *i.e.,* one 2p and one 3d set on H; one 3d and one 4f set on C, Si, P, Cl; one 6p and one 5f set on Pt. All electrons were included in the variational process, *i.e.,* no frozen core approximation was used. For all calculations, the accuracies of both the Zlm fitting and the Becke integration grid were set to “Verygood”.[16] No symmetry constraints were used for the computations. Gibbs free energies G are calculated by adding thermal corrections computed at 298 K to the total electronic energy E; zero-point vibrational energies and vibrational partition functions are computed using the vibrational frequencies computed at the same level.[17] All calculated stationary points have been verified by performing a vibrational analysis calculation to be energy minima (no imaginary frequencies) or transition states (only one imaginary frequency). The character of the normal mode associated with the imaginary frequency of the transition state has been inspected and the transition-state structures have been checked through an intrinsic reaction coordinate (IRC) calculation[18] to ensure that the transition state is associated with the reaction of interest. A list of the Cartesian coordinates and energies of all optimised structures can be found in Table S2. The ball-and-stick figures of the optimised structures were created using the CYLview20 software.[19]

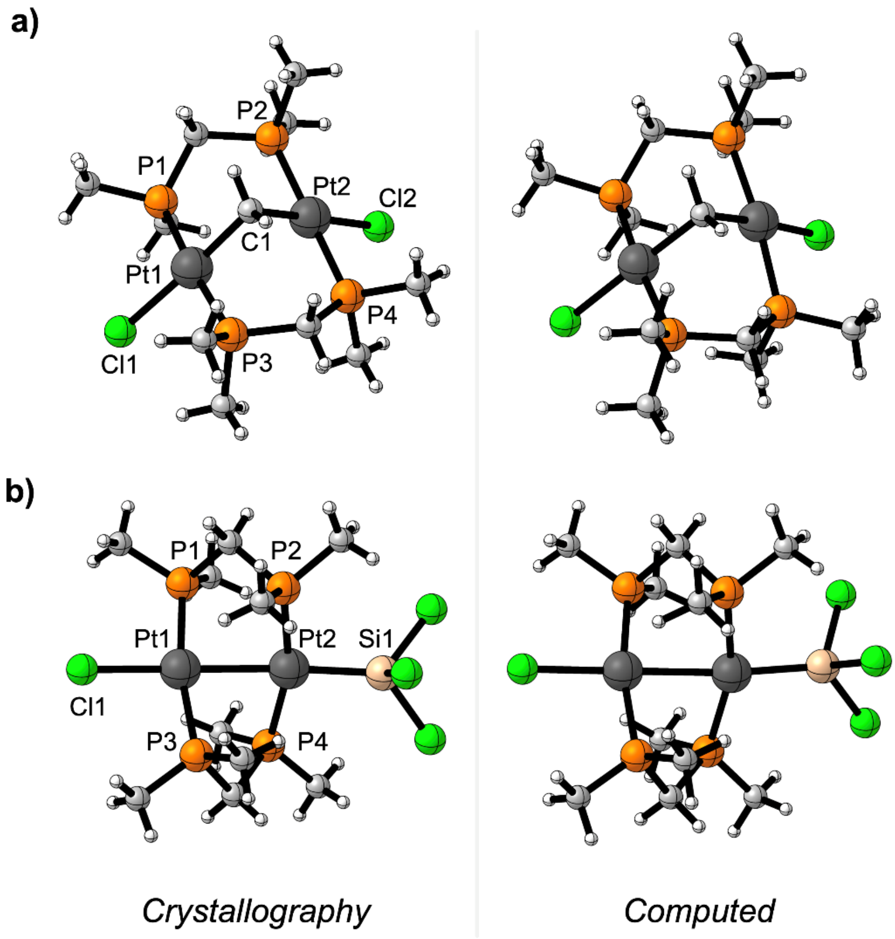

Figure S78. Agreement between SCXRD-derived (left) and computed (right) structures of **a) 2^{CH2}-Cl** and **b) 3^{SiCl3}-Cl**. Selected experimental (red) bond lengths (Å) and angles (°), with computed (blue) values at the COSMO(benzene)-ZORA-BLYP-D3(BJ)/TZ2P level in parentheses: **2^{CH2}-Cl** | Pt1–Cl1 2.44 (2.51); Pt1–C1 2.05 (2.09); Pt1–Pt2 3.15 (3.19); Pt1–P1 2.29 (2.32); Pt1–P3 2.27 (2.29); Cl1–C1–Cl2 100 (96); P1–Pt1–P3 175 (174); P1–Pt1–Cl1 89 (87); P3–Pt1–Cl1 94 (94). **3^{SiCl3}-Cl** | Pt1–Pt2 2.67 (2.73); Pt1–Cl1 2.42 (2.49); Pt2–Si1 2.29 (2.32); Pt1–P1 2.26 (2.29); Pt1–P3 2.27 (2.29); Pt2–P2 2.29 (2.33); Pt2–P4 2.29 (2.32); Cl1–Pt1–P1 96 (96); Cl1–Pt1–P3 95 (96); P1–Pt1–P3 169 (168); P2–Pt2–P4 164 (162); P2–Pt2–Si1 93 (95); P4–Pt2–Si1 103 (103).

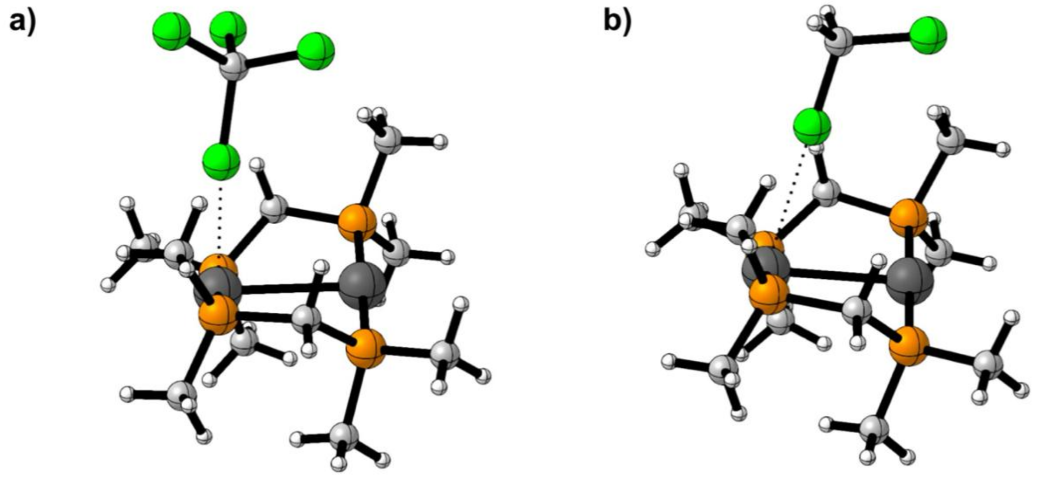

Figure S79. Ball-and-stick structures of the reactant complex (RC) for (a) CCl_4 and (b) CH_2Cl_2 interacting with **1**, computed at the COSMO(benzene)-ZORA-BLYP-D3(BJ)/TZ2P level. Atom colours: H = white; C = grey; P = orange; Cl = green; Pt = anthracite.

Energy decomposition analysis

The interaction energy ΔE_{int} between the $SiCl_4$ and $[(\mu\text{-dmpm})_2Pt_2]$ molecules in the reactant complex (RC) and the first transition state (TS1) geometry of the A-frame (AF) and transannular (TA) pathway (see Figure 7 in the main text) was partitioned into four physically meaningful terms using a quantitative energy decomposition analysis (EDA):[20] (i) the classical electrostatic interaction (ΔV_{elstat}) between the molecules, (ii) the steric Pauli repulsion (ΔE_{Pauli}) arising from the repulsion between overlapping closed-shell orbitals on the interacting molecules, iii) the orbital interaction (ΔE_{oi}) which accounts for charge transfer between the molecules (*i.e.,* donor–acceptor interactions, including HOMO–LUMO interactions) and the mutual polarisation of the molecules due to the presence of the other molecule, and iv) the dispersion energy (ΔE_{disp}) (see Equation (S1) and the results in Table S1).

$$\Delta E_{int} = \Delta V_{elstat} + \Delta E_{Pauli} + \Delta E_{oi} + \Delta E_{disp} \qquad \text{(S1)}$$

Table S1. Energy decomposition analysis of the interaction energy ΔE_{int} (in kcal mol^{-1}) between the $SiCl_4$ and $[(\mu\text{-dmpm})_2Pt_2]$ molecules in the reactant complex (RC) and the first transition state (TS1) geometry of the A-frame (**AF**) and transannular (**TA**) pathway (see Figure 7 in the main text).[a,b]

	ΔE_{int}	ΔV_{elstat}	ΔE_{Pauli}	ΔE_{oi}	ΔE_{disp}
**AF-RC**	–108.4	–157.1	250.5	–163.8	–38.0
**TA-RC**	–112.9	–154.6	229.7	–159.9	–28.1
**AF-TS1**	–125.6	–193.5	300.9	–194.2	–38.8
**TA-TS1**	–121.4	–170.0	251.5	–175.0	–27.9

[a] $\Delta E_{int} = \Delta V_{elstat} + \Delta E_{Pauli} + \Delta E_{oi} + \Delta E_{disp}$. [b] Computed at ZORA-BLYP-D3(BJ)/TZ2P//COSMO(benzene)-ZORA-BLYP-D3(BJ)/TZ2P.

Cartesian coordinates

Table S2. Cartesian coordinates (in Å), ADF total bond energies *E*, enthalpies *H*, Gibbs free energies *G* (in kcal mol^{-1}), and the number of imaginary frequencies (N_{imag}) of all reported structures, optimised at the COSMO(benzene)-ZORA-BLYP-D3(BJ)/TZ2P level of theory.

2^{CH2}-Cl

E: -5595.70

H: -5333.52

G: -5393.54

N_{imag} = 0

Pt	2.73370593	9.49641768	6.72163965
Pt	3.09093720	10.26172323	3.64719298
Cl	2.43725284	7.37990718	8.03859478
Cl	3.28566098	9.00764610	1.48135752
P	0.55114781	9.21383086	5.98258789
P	0.88481049	10.80166884	3.32219515
P	5.24398570	9.54175756	4.13229111
P	4.96206779	9.69239211	7.23206490
C	0.39380577	7.67010555	5.00111111
H	-0.62696007	7.53861510	4.62579569
H	0.66535282	6.83712171	5.65558525
H	1.10400273	7.70551457	4.17021724
C	-0.75300215	9.06013629	7.26745075
H	-1.72345054	8.81710575	6.82060693
H	-0.82575770	10.00065266	7.82199344
H	-0.44605567	8.26748980	7.95611656
C	-0.12391770	10.53196519	4.86440547
H	-1.16131170	10.30207671	4.59498464
H	-0.12372449	11.46566053	5.43642735
C	0.51620419	12.55423500	2.89863794
H	-0.56424408	12.72908474	2.84646942
H	0.97204558	12.77982723	1.92961181
H	0.96172068	13.20601751	3.65564674
C	-0.02288165	9.85554438	2.03886898
H	-1.06828439	10.17944334	1.99259531
H	0.02949170	8.78818356	2.26455467
H	0.47003162	10.01899458	1.07707401
C	6.55518496	9.94461697	2.91012091
H	7.50442324	9.46538217	3.17430902

H	6.68753688	11.02999525	2.86750943
H	6.21438504	9.59211149	1.93219323
C	5.30229895	7.71113030	4.26099705
H	6.30896902	7.36110402	4.51440996
H	4.99400705	7.30507293	3.29352057
H	4.58383161	7.38701601	5.01911748
C	5.97552800	10.13143996	5.73248993
H	6.03495440	11.22314893	5.67225514
H	6.99476413	9.74422926	5.84587453
C	5.79678045	8.20091543	7.89902885
H	6.85822962	8.40427964	8.07771801
H	5.68672779	7.37321942	7.19495490
H	5.30093697	7.91618470	8.83058268
C	5.42306331	11.00939081	8.43204746
H	6.51083551	11.07756637	8.54498270
H	4.96758584	10.77242588	9.39852311
H	5.02533625	11.96647176	8.08250010
C	2.95122215	11.18119770	5.51304222
H	2.09359707	11.85505241	5.61582419
H	3.84658404	11.77393084	5.73023028

3^{CH2Cl}-Cl

E: -5573.17

H: -5311.47

G: -5374.07

$N_{imag} = 0$

Pt	3.61928078	9.96983151	4.84528927
Pt	2.16135471	11.78844454	6.27323836
C	0.99261485	13.07713086	7.44408857
Cl	4.95995679	8.25941254	3.54349062
H	1.53261307	13.82012444	8.03255070
Cl	0.08224193	12.08098803	8.79472606
H	0.16908027	13.56889921	6.92222269
P	4.29496871	9.30549704	6.92411774
P	4.17443162	12.28598482	7.24241966
P	0.39718142	10.95405343	5.06912173
P	2.62097427	10.95494730	3.04768928
C	2.94474817	8.68553725	8.01366709
H	2.19659036	9.47706390	8.12024135
H	3.33627944	8.40001363	8.99685855

H	2.47621884	7.81839313	7.53908510
C	5.60107926	8.02116421	7.09247342
H	5.24102912	7.09802627	6.62849786
H	5.84955957	7.84171086	8.14456459
H	6.48956463	8.34595050	6.54353453
C	4.96811941	10.74159819	7.90213044
H	6.05615842	10.79909781	7.78541204
H	4.74108389	10.62370565	8.96777111
C	5.43407477	12.94426947	6.07460875
H	5.56143630	12.21002287	5.27349322
H	6.38673739	13.12225370	6.58632669
H	5.06679830	13.87971345	5.64210821
C	4.26422137	13.45307224	8.66648965
H	3.94651774	14.44834045	8.33911892
H	5.28445910	13.51204809	9.06102055
H	3.58581371	13.11167529	9.45459018
C	-1.26377215	11.74189013	5.18514486
H	-1.19182579	12.79001084	4.87848055
H	-1.59668685	11.70321219	6.22717051
H	-1.98906479	11.22489518	4.54749816
C	0.04755376	9.18793983	5.44003080
H	-0.78357273	8.81719651	4.82959989
H	-0.20198528	9.09562070	6.50114689
H	0.95685771	8.61520136	5.23584455
C	0.76811290	10.95712483	3.24832035
H	0.32635870	11.83899978	2.77031890
H	0.34314326	10.06293831	2.77863201
C	3.00178687	12.74251791	2.81635551
H	4.07761049	12.85568490	2.65212403
H	2.72887866	13.26848184	3.73651421
H	2.45307696	13.15659081	1.96252544
C	2.86654572	10.24966384	1.36635233
H	2.27653526	10.78978779	0.61727863
H	2.58341271	9.19338655	1.38210329
H	3.93070883	10.30607747	1.11825139

2^{CCl2}-Cl

E: -5523.80

H: -5271.60

G: -5335.32

$N_{imag} = 0$

Pt	2.03433589	8.58743482	5.99136815
Pt	2.98327592	10.77370169	3.86163037
Cl	2.94430707	7.28898248	7.90290583
Cl	4.87636335	12.33149094	3.46437493
P	3.62997091	7.35375515	4.73846588
P	3.83724089	9.30444003	2.29029461
P	2.62412188	12.12189057	5.78285692
P	0.97859376	10.03770955	7.45375134
C	5.29878256	7.84106457	5.33388498
H	6.08010353	7.28726971	4.80213719
H	5.35268169	7.63723755	6.40570489
H	5.42483397	8.91564484	5.17150015
C	3.57759089	5.52634482	4.90936326
H	4.44099814	5.06396068	4.41872539
H	2.65319114	5.15753582	4.45438630
H	3.57298740	5.28390513	5.97484615
C	3.79914329	7.54733082	2.89759462
H	4.72691905	7.04952841	2.59167180
H	2.95597660	7.03526455	2.43390592
C	3.07367188	9.24936249	0.62066821
H	3.52737389	8.45504240	0.01774589
H	3.24609615	10.21777985	0.14038912
H	2.00010752	9.07925731	0.71890151
C	5.61813310	9.53929754	1.91039222
H	5.94767395	8.75281055	1.22268521
H	6.20171594	9.49856078	2.83282658
H	5.76291185	10.52312346	1.46016556
C	2.47539379	13.93039842	5.50276983
H	2.46509189	14.47298562	6.45433546
H	1.54742582	14.12553769	4.95650928
H	3.32334959	14.25156354	4.89296237
C	4.08590968	11.92641486	6.87906610
H	3.98505668	12.54424667	7.77795632
H	4.97643403	12.21738495	6.31734102
H	4.17208555	10.87181506	7.15726772
C	1.21371790	11.81317523	6.95366939
H	0.30181003	12.16414466	6.47061009
H	1.38929595	12.41189005	7.85524879
C	1.65239808	10.02864103	9.16160228
H	1.14559925	10.79822655	9.75402148

H	2.72700308	10.22285731	9.13628421
H	1.49581153	9.04335101	9.60462970
C	-0.82760026	9.85446555	7.73280726
H	-1.19894096	10.65641830	8.38030765
H	-0.99624225	8.88638859	8.21512088
H	-1.34633050	9.87659743	6.77283963
C	1.40921115	9.57925362	4.33242141
Cl	0.74392015	8.28434339	3.05083816
Cl	-0.19694122	10.66707819	4.38744865

3^{CCl3}-Cl

E: -5498.31

H: -5247.53

G: -5310.88

N_{imag} = 0

Pt	3.51628201	10.02813865	4.92624446
Pt	2.02687028	11.83218342	6.35527223
C	0.99651499	13.20228570	7.58588865
Cl	4.88092215	8.38614451	3.64233817
Cl	1.64154702	14.96318476	7.39633976
Cl	1.22534431	12.75622556	9.41199847
Cl	-0.84415445	13.40786147	7.43684910
P	4.15667585	9.28386859	6.99701708
P	4.14911778	12.28123213	7.21599148
P	0.35309064	10.87982191	5.05508549
P	2.63428723	11.08006402	3.09917571
C	2.80401746	8.70973134	8.10355885
H	2.07813796	9.52193341	8.20750581
H	3.19868763	8.42863871	9.08635899
H	2.30992230	7.84778430	7.64587734
C	5.42939417	7.96790801	7.14129744
H	5.03407642	7.04890029	6.69835636
H	5.69710676	7.79387127	8.18932989
H	6.31183464	8.26436628	6.56771920
C	4.87487032	10.72319219	7.92244003
H	5.96482679	10.73922391	7.80968181
H	4.63864124	10.66043149	8.99058925
C	5.35868793	12.79481463	5.92686712
H	5.40418019	12.01798522	5.15835284
H	6.34627448	12.95278140	6.37432845

H	5.00871342	13.72696976	5.47361621
C	4.51755167	13.49654159	8.55309346
H	4.29459071	14.50557145	8.20088259
H	5.57916943	13.42027472	8.81423253
H	3.90022088	13.28843958	9.42830251
C	-1.42181767	11.38670482	5.00956393
H	-1.50400668	12.45386252	4.79386190
H	-1.89213631	11.18849807	5.97493746
H	-1.92648635	10.80828367	4.22689459
C	0.18963530	9.06370905	5.31995453
H	-0.57506407	8.64842858	4.65415574
H	-0.09533124	8.88628618	6.36125157
H	1.15959247	8.59513279	5.13058658
C	0.78767526	11.02633764	3.25117344
H	0.33066383	11.93029697	2.83279844
H	0.39142087	10.15938151	2.71023704
C	2.99265924	12.87850117	2.95440905
H	4.07159278	13.01962129	2.84248483
H	2.66501806	13.36838941	3.87642803
H	2.47416403	13.31067554	2.09118516
C	2.97382251	10.43027564	1.41535101
H	2.40443918	10.98150860	0.65880581
H	2.71441238	9.36840876	1.38698509
H	4.04584595	10.51954220	1.21563088

2^{SiCl2}-Cl

E: -5538.09

H: -5286.25

G: -5350.78

N_{imag} = 0

Pt	2.03963015	8.50025416	6.10364108
Pt	3.06797193	10.86921351	3.79939918
Cl	3.13524079	7.31086093	8.04067538
Cl	5.09958973	12.34532352	3.55278252
P	3.62366668	7.36248603	4.77661528
P	3.87196964	9.31960941	2.29156097
P	2.65518987	12.11466061	5.75977633
P	0.99904807	10.02795314	7.48380086
C	5.28452657	7.92360629	5.32463151
H	6.07013119	7.41741287	4.75336100

H	5.38482643	7.70210339	6.38943015
H	5.35355627	9.00550611	5.17837248
C	3.65687684	5.53451048	4.93892530
H	4.53267855	5.11128233	4.43541416
H	2.74297605	5.12287916	4.49987318
H	3.68089153	5.29468697	6.00530800
C	3.72325330	7.57569948	2.93083563
H	4.58825160	7.00255482	2.57680326
H	2.81960088	7.13635317	2.50485443
C	3.14092548	9.24121273	0.60881818
H	3.58674235	8.42260363	0.03335170
H	3.34343496	10.19360922	0.10891781
H	2.06166697	9.09710074	0.68721094
C	5.66920979	9.46887777	1.95018109
H	5.98438474	8.65602450	1.28691927
H	6.22989668	9.43004696	2.88616326
H	5.85931407	10.43760831	1.48303696
C	2.54901046	13.93579808	5.55774353
H	2.54143900	14.43927548	6.53042994
H	1.63433106	14.17870605	5.00839024
H	3.41424598	14.25741411	4.97191384
C	4.08328215	11.83746064	6.88109601
H	3.95997046	12.40509195	7.80972044
H	4.99100885	12.15060792	6.36063612
H	4.15108030	10.76827050	7.10230910
C	1.20506463	11.77737179	6.87609737
H	0.30279620	12.05220815	6.32713119
H	1.29652044	12.43735329	7.74680010
C	1.70186168	10.10739835	9.17750271
H	1.20361366	10.90203534	9.74341730
H	2.77557121	10.29834435	9.12591879
H	1.55409886	9.14152787	9.66541834
C	-0.80390279	9.87281768	7.79594136
H	-1.15883534	10.69935495	8.42114410
H	-0.97556813	8.92288969	8.31184641
H	-1.33908172	9.86857885	6.84449267
Si	1.22841728	9.56344746	4.23522839
Cl	0.48015813	8.15823370	2.77290336
Cl	-0.58691943	10.73477153	4.31273193

3^{SiCl3}-Cl

E: -5530.50

H: -5280.06

G: -5344.97

N_{imag} = 0

Pt	3.54722746	9.98675809	4.89954614
Pt	2.06636800	11.79940812	6.30744131
Si	0.92030523	13.29369452	7.66547446
Cl	4.89416874	8.31947912	3.63127544
Cl	1.59137757	15.33196122	7.53527075
Cl	1.15149760	12.83141063	9.75689067
Cl	-1.19644947	13.59537602	7.59234207
P	4.17964733	9.27465275	6.98307102
P	4.16334470	12.27016820	7.19820828
P	0.37045963	10.87631845	5.02284920
P	2.65491100	11.02897791	3.07095278
C	2.81888280	8.72182676	8.09093971
H	2.09827620	9.53991031	8.18747565
H	3.20801557	8.44562498	9.07726542
H	2.31859705	7.86057000	7.63884704
C	5.44492766	7.95445633	7.15046175
H	5.04634339	7.03222053	6.71724712
H	5.70730323	7.79200138	8.20164769
H	6.33135283	8.23900312	6.57693251
C	4.90127793	10.71860276	7.90118878
H	5.98969623	10.74107794	7.77597597
H	4.67811226	10.65463749	8.97202086
C	5.36836202	12.81256313	5.91946035
H	5.43615647	12.03599730	5.15245477
H	6.34829176	12.99240615	6.37522581
H	5.00167972	13.73665492	5.46319258
C	4.46589805	13.48793982	8.54300944
H	4.18902830	14.48693558	8.19825015
H	5.52749991	13.46914758	8.81314000
H	3.85478161	13.23956365	9.41313697
C	-1.36959678	11.47093257	5.01909088
H	-1.39769712	12.54162405	4.80432950
H	-1.81892828	11.30611107	6.00073960
H	-1.93377969	10.92258270	4.25616335
C	0.15793980	9.07332735	5.32031105
H	-0.63023340	8.67147627	4.67402830

H	-0.11347865	8.91935067	6.36878347
H	1.11070902	8.57473433	5.12087826
C	0.80531483	10.98757292	3.21888479
H	0.35488620	11.88530871	2.78064888
H	0.40512135	10.11215494	2.69494316
C	3.01647678	12.82638499	2.91672329
H	4.09631545	12.96530163	2.81158003
H	2.68381522	13.32527427	3.83232734
H	2.50469661	13.25375012	2.04716102
C	2.98937034	10.37097361	1.38941160
H	2.41972449	10.91937502	0.63108200
H	2.72848977	9.30931683	1.36676283
H	4.06123828	10.45775220	1.18764493

$SiCl_4$

E: -409.91

H: -400.89

G: -425.88

N_{imag} = 0

Cl	-1.67406544	-0.00000000	-0.58837899
Si	0.00000000	-0.00000000	0.59542965
Cl	0.00000000	-1.67404494	1.77926093
Cl	-0.00000000	1.67404494	1.77926093
Cl	1.67406544	0.00000000	-0.58837899

1

E: -9471.90

H: -9036.15

G: -9107.43

N_{imag} = 0

Pt	5.42674799	4.45340225	4.07981613
C	3.23237674	7.24170143	4.11946564
H	2.36408530	6.77926945	4.60480283
H	2.87797575	8.07300176	3.49765248
P	4.37475941	7.92038446	5.42480866
C	4.66507306	7.05710713	1.66618838
H	3.84825745	7.65307611	1.24126920
H	5.11159477	6.43878802	0.88228566
H	5.42844645	7.72178571	2.07742018

C	3.42860175	9.41956660	5.96708537
H	3.92797342	9.85391752	6.83964802
H	2.39079365	9.17631941	6.22617244
H	3.44679798	10.15794810	5.16009848
C	4.00222804	6.76101690	6.81782201
H	4.54417235	7.08095746	7.71230794
H	4.34325335	5.76404532	6.52899771
H	2.92678990	6.73462211	7.03151629
C	2.57507820	5.20132423	2.22173950
H	1.91644018	5.95518651	1.77313948
H	2.02372287	4.62407624	2.96977152
H	2.92010836	4.51206274	1.44374138
P	4.05359965	5.97069486	3.03340169
C	5.53219653	2.72459402	5.36135798
H	6.07038609	2.87127152	6.29871525
C	5.61018962	1.32846526	4.72319780
H	6.61139779	0.98374219	4.44731396
C	4.84418744	0.36575989	5.69405054
H	5.20062453	0.46354754	6.72549806
H	4.97701135	-0.68051313	5.39110519
C	3.34547144	0.81632747	5.52701272
H	2.72817322	-0.00444411	5.14051897
H	2.90034387	1.15512752	6.46912352
C	3.44332867	1.97985073	4.48164719
H	2.49658793	2.22064659	3.98861749
C	4.13892202	3.14349437	5.20595508
H	3.59155239	3.61652973	6.02231178
C	4.57793285	1.44133575	3.56938102
H	4.87919893	2.15737942	2.79771812
H	4.33657542	0.47096140	3.11763869
Pt	6.53107393	8.37556144	4.77219343
C	8.72544611	5.58726656	4.73255996
H	9.59373811	6.04969741	4.24722271
H	9.07984620	4.75596672	5.35437429
P	7.58306503	4.90858672	3.42721377
C	7.29275540	5.77187900	7.18584369
H	8.10957238	5.17591422	7.61076613
H	6.84623559	6.39020515	7.96974195
H	6.52938122	5.10719651	6.77461992
C	8.52922532	3.40940890	2.88492963
H	8.02985593	2.97506291	2.01236321

H	9.56703371	3.65265877	2.62584626
H	8.51102812	2.67102229	3.69191183
C	7.95559840	6.06796507	2.03420986
H	7.41365611	5.74803068	1.13972051
H	7.61457181	7.06493444	2.32304065
H	9.03103694	6.09436226	1.82051792
C	9.38274807	7.62765606	6.63027065
H	10.04138746	6.87379759	7.07887506
H	9.93410131	8.20489815	5.88223252
H	9.03771971	8.31692372	7.40826409
P	7.90422471	6.85827854	5.81861849
C	6.42561821	10.10435523	3.49063351
H	5.88742112	9.95766803	2.55328205
C	6.34763161	11.50049118	4.12877900
H	5.34642584	11.84521695	4.40466848
C	7.11362614	12.46318478	3.15790836
H	6.75717991	12.36538504	2.12646519
H	6.98080676	13.50946185	3.46084156
C	8.61234318	12.01261733	3.32493825
H	9.22964556	12.83339326	3.71141646
H	9.05746077	11.67380561	2.38282687
C	8.51449415	10.84910646	4.37031848
H	9.46123894	10.60831559	4.86334290
C	7.81889354	9.68545494	3.64603029
H	8.36625596	9.21240914	2.82967473
C	7.37989834	11.38763306	5.28258808
H	7.07863829	10.67159848	6.05426174
H	7.62126040	12.35801241	5.73431722

nbe

E: -2186.36

H: -2089.54

G: -2111.49

$N_{imag} = 0$

C	5.39295722	2.47205323	5.37969741
C	5.54058715	1.10560875	4.71377454
H	6.16580969	2.95921913	5.96815746
C	4.11227041	2.85760127	5.23764084
C	4.52308960	1.25296514	3.54608598
C	4.76351673	0.08722254	5.63623201

H	6.55698324	0.79632738	4.45941273
C	3.38016823	1.75597353	4.47415857
H	3.63124306	3.72221685	5.68703980
H	4.83005006	2.00051582	2.80735297
H	4.29259712	0.30357080	3.04757766
C	3.27081408	0.53658832	5.47068124
H	5.10990599	0.13607326	6.67286442
H	4.91164724	-0.93715607	5.27606668
H	2.43576643	2.03693233	4.00235833
H	2.80727400	0.82926898	6.41749498
H	2.66355292	-0.26042093	5.02678618

TA-RC

E: -5507.85

H: -5256.74

G: -5323.2

$N_{i\text{mag}} = 0$

Pt	2.45867485	12.60823860	4.88206230
Pt	2.86875979	10.32267920	6.44405872
Cl	1.55381290	7.93241299	9.39721057
Si	3.06471996	8.69694372	8.10955823
Cl	4.90323719	7.89568074	8.84842371
H	0.21454973	15.45385164	5.95974142
Cl	3.29451285	10.61106398	9.56640603
P	4.18836814	11.86287883	3.60719239
P	5.16884882	10.41840806	6.12484633
P	0.57530854	10.11369350	6.13458635
P	0.79390891	13.13012237	6.37003484
C	3.93642064	10.25527785	2.74736174
H	3.55857224	9.52631482	3.46967784
H	4.86898670	9.89589476	2.29844847
H	3.18399800	10.39119853	1.96455214
C	4.89331282	12.94185263	2.29035603
H	4.12901209	13.11815302	1.52670521
H	5.76890924	12.47182621	1.82792432
H	5.18050910	13.90297364	2.72702259
C	5.62397492	11.57090939	4.73243247
H	5.86840524	12.54276479	5.17402013
H	6.50470063	11.19431773	4.19792108
C	6.23830971	11.00887704	7.49173950

H	5.89357040	12.00069589	7.79685628
H	7.28300507	11.05381599	7.16509929
H	6.13462710	10.33018923	8.34045057
C	5.93020218	8.84171015	5.56758345
H	5.79677712	8.08312111	6.33994136
H	6.99711471	8.99180152	5.36881045
H	5.42682785	8.50300122	4.65892857
C	-0.47642594	8.80298027	6.86874874
H	-0.52424046	8.92447854	7.95155674
H	-0.03275677	7.82849488	6.64875302
H	-1.48113897	8.86766313	6.43667034
C	0.24531944	9.89876314	4.33645389
H	-0.83345561	9.86452908	4.14716997
H	0.70704122	8.96072248	4.01487644
H	0.70445721	10.73117376	3.79447714
C	-0.33842958	11.66917223	6.57492990
H	-0.68083890	11.60292444	7.61353820
H	-1.21501124	11.78037773	5.92665807
C	1.35801541	13.47435586	8.08125357
H	2.01162017	14.35193991	8.06848355
H	1.92903909	12.61454648	8.44722339
H	0.50088533	13.66208041	8.73796800
C	-0.35656232	14.52056290	5.99362874
H	-1.13469141	14.59867174	6.76148096
H	-0.81937724	14.35624606	5.01593192
Cl	2.79616730	6.97363433	6.59282307

TA-TS1

E: -5507.52

H: -5257.01

G: -5322.33

$N_{i\text{mag}}$ = 1 (-62.3775 cm^{-1})

Pt	1.27663297	10.08057049	6.29904631
Pt	3.48944620	11.63786516	5.63595200
Si	5.37077404	12.97549991	5.49278376
Cl	4.11947150	13.80136456	7.77234517
Cl	5.34820036	15.00451567	4.86960895
Cl	6.26890420	12.15339563	3.63681599
Cl	7.09533682	12.81013344	6.70310358
P	0.46553067	10.63601670	4.24606149

P	2.16507605	13.17025788	4.48221252
P	4.59810652	9.71596037	6.31346564
P	2.30873373	9.67714875	8.31159680
C	1.44746873	10.05986818	2.80032885
H	2.49317473	10.33949358	2.95686521
H	1.07186919	10.49759768	1.86874833
H	1.38314684	8.96904568	2.74290957
C	-1.26210844	10.18034053	3.79618501
H	-1.35089537	9.08933990	3.78028817
H	-1.52749093	10.58171784	2.81136384
H	-1.94783737	10.57687192	4.55082812
C	0.47547637	12.47617922	4.10643978
H	-0.21088566	12.84660870	4.87506630
H	0.12717604	12.82302336	3.12601299
C	1.76100904	14.76886646	5.28006100
H	1.28730863	14.56023314	6.24300179
H	1.09053472	15.35362056	4.64063661
H	2.68668923	15.31520406	5.46791704
C	2.77073728	13.60730470	2.80213306
H	3.73663703	14.10815144	2.88384963
H	2.05157591	14.26589984	2.30276644
H	2.90111022	12.69128938	2.22034403
C	6.42190746	9.51279025	6.34069736
H	6.86179004	10.17699271	7.08511286
H	6.82468515	9.77781145	5.35927516
H	6.66279947	8.47024192	6.57547670
C	4.08702991	8.28300480	5.27629164
H	4.54149852	7.35931121	5.65218015
H	4.41971764	8.46618881	4.25039644
H	2.99514741	8.20954883	5.29123164
C	4.09845373	9.23392034	8.03182209
H	4.72519993	9.78540538	8.74086825
H	4.25609724	8.16064723	8.18791339
C	2.38548465	11.09120801	9.47222906
H	1.36505114	11.39335176	9.72799924
H	2.89195191	11.92716688	8.97425873
H	2.92741116	10.81121642	10.38286871
C	1.67589800	8.28181636	9.33748506
H	2.29292173	8.15097020	10.23373693
H	1.68415214	7.36072583	8.74700269
H	0.64529368	8.49963805	9.63523286

TA-I

E: -5512.67

H: -5262.32

G: -5325.28

N_{imag} = 0

Pt	2.42664803	12.29226589	4.67833136
Pt	2.83124684	9.93958916	6.45611370
Si	2.72190992	7.73710365	6.98855908
Cl	3.43643506	12.10983065	8.06402174
Cl	4.38442676	6.43324568	6.75599690
Cl	1.19856533	6.58342430	6.04065064
Cl	2.33211056	7.44853115	9.05953221
P	4.53583539	12.08449407	3.89537744
P	5.11673925	9.91695495	5.99132571
P	0.51705168	10.15606118	6.74257719
P	0.60276067	13.03915636	5.81453048
C	4.98452203	11.03942089	2.44111753
H	4.54157978	10.04699225	2.54050571
H	6.07060290	10.95431062	2.32206046
H	4.55941201	11.51418717	1.55079607
C	5.29993715	13.70127552	3.44070567
H	4.76592846	14.12016531	2.58213580
H	6.35874482	13.57307310	3.18565770
H	5.20429383	14.38985822	4.28477587
C	5.63544082	11.53434321	5.27968452
H	5.49054179	12.27235107	6.07691281
H	6.69156375	11.49929874	4.98391551
C	6.18143267	9.73181167	7.47077485
H	5.88241234	10.49585105	8.19324674
H	7.23733569	9.84635830	7.20286792
H	6.01583644	8.74029580	7.90100556
C	5.81086867	8.71361397	4.77793141
H	6.02582389	7.77270014	5.28206576
H	6.73265103	9.11997676	4.34991151
H	5.08866129	8.52090154	3.98302992
C	-0.38997400	9.22833174	8.04364040
H	0.05720084	9.44281698	9.01707831
H	-0.33508213	8.15489027	7.85703631
H	-1.43760748	9.54837653	8.03704205

C	-0.42008294	9.77817049	5.21010602
H	-1.48079927	10.02129677	5.33641008
H	-0.30793661	8.71325606	4.99398510
H	0.01100962	10.35279837	4.38575173
C	0.07912844	11.89175880	7.16894794
H	0.67522869	12.14227788	8.05292257
H	-0.98939524	11.98268810	7.39615920
C	0.92863182	14.61045626	6.71254196
H	1.15015588	15.39940487	5.98729232
H	1.80114290	14.44922105	7.35175032
H	0.06198440	14.89962728	7.31845426
C	-0.97500388	13.39074390	4.92096368
H	-1.74075900	13.76262916	5.61173295
H	-1.33485049	12.48298380	4.42985174
H	-0.78091020	14.14617054	4.15303231

TA-TS2

E: -5510.35

H: -5259.59

G: -5325.03

$N_{i\text{mag}}$ = 1 (-28.9197 cm^{-1})

Pt	3.66040443	9.93360841	4.98152626
Pt	1.93279913	11.63676032	6.35035796
Si	0.66066719	12.96759060	7.70262043
Cl	1.30473738	8.09894251	6.09461272
Cl	1.27223706	15.00238491	7.71708299
Cl	0.71164207	12.34352191	9.72686876
Cl	-1.43242721	13.19068168	7.43547370
P	4.85377544	9.30491333	6.84870380
P	3.88650239	12.08007481	7.56995838
P	0.18932037	11.24391943	4.83245130
P	2.58146228	10.57077102	3.10222617
C	4.51895582	7.66281498	7.59292477
H	3.43555950	7.56989095	7.71630336
H	5.04532582	7.54834667	8.54714237
H	4.85014402	6.88847200	6.89332132
C	6.69101243	9.33606334	6.67717048
H	6.98487484	8.62080396	5.90265674
H	7.17590442	9.06687352	7.62285386
H	7.01379978	10.33388306	6.36816337

C	4.55497274	10.49671879	8.23873336
H	5.45837227	10.66002234	8.83802675
H	3.77144491	10.08419091	8.88370760
C	5.18832456	12.79779497	6.48888422
H	5.28135569	12.17670033	5.59323170
H	6.14061900	12.86437565	7.02674099
H	4.86466500	13.80173869	6.19643405
C	3.99281828	13.18001844	9.04081607
H	3.65913421	14.18615862	8.78016293
H	5.03793697	13.21160586	9.36731776
H	3.36512416	12.79704151	9.84629701
C	-0.47799488	12.78056298	4.06476408
H	0.33666385	13.35501495	3.61871349
H	-0.96662771	13.39074729	4.82537739
H	-1.20477939	12.51570412	3.28890169
C	-1.28718614	10.30990463	5.37449567
H	-1.96900936	10.18851088	4.52529248
H	-1.78963008	10.84859135	6.17900933
H	-0.93345371	9.33794924	5.73118341
C	0.77653909	10.29949935	3.35793598
H	0.16642227	10.53203230	2.47699211
H	0.68213537	9.24047043	3.62588349
C	2.75203300	12.30888886	2.51939480
H	3.78244596	12.45538283	2.18094579
H	2.56861487	12.98700727	3.35656303
H	2.06239593	12.52729407	1.69614283
C	2.94139931	9.58345426	1.59000941
H	2.30052791	9.90174300	0.75941073
H	2.76401517	8.52584751	1.80565476
H	3.99174856	9.71736004	1.31324734

AF-RC

E: -5481.06

H: -5230.32

G: -5296.24

$N_{imag} = 0$

Pt	-1.69557035	0.37433772	1.28399741
Pt	-0.81716474	1.18049507	-1.33423083
Cl	1.39108205	1.15027817	2.74394222
Cl	1.86643730	3.02596804	-0.05058174

P	-3.39361214	1.96526289	0.81670179
P	-1.72734764	3.23897708	-1.49131311
P	-0.78821446	-1.16970650	-1.66116591
P	-0.85424131	-1.71512621	1.40940414
C	-4.70664217	1.12310167	-0.17393759
H	-5.54886168	1.79488969	-0.37540134
H	-5.06491376	0.24497838	0.37218502
H	-4.26657675	0.79002125	-1.11821469
C	-4.33775171	2.67205533	2.23339588
H	-5.15427023	3.31711526	1.88858475
H	-3.64617276	3.24755532	2.85501149
H	-4.74661123	1.85020292	2.83001746
C	-3.06388350	3.47958882	-0.21467328
H	-3.99595106	3.78819770	-0.70242111
H	-2.74256700	4.26932649	0.46528095
C	-0.81507405	4.83317527	-1.47822102
H	-1.51232771	5.66942906	-1.60311102
H	-0.09186259	4.82613922	-2.29921392
H	-0.27631249	4.92375751	-0.53382540
C	-2.67188923	3.33786196	-3.07772637
H	-3.23272041	4.27821928	-3.13575509
H	-3.36618210	2.49487600	-3.14312791
H	-1.96958330	3.27980817	-3.91453513
C	0.22869724	-1.84432372	-3.04268002
H	0.08592137	-2.92543269	-3.15434073
H	1.28002276	-1.62750838	-2.83385480
H	-0.05731178	-1.33885908	-3.97070750
C	-2.49958477	-1.70169702	-2.11153384
H	-2.54099290	-2.77511153	-2.32907305
H	-2.83411895	-1.13996188	-2.98917757
H	-3.16476025	-1.46909694	-1.27503230
C	-0.38407303	-2.34964407	-0.27900276
H	0.69527386	-2.50490014	-0.29256303
H	-0.88298297	-3.30677749	-0.47097684
C	-2.21820303	-2.86343041	1.89903295
H	-1.88235209	-3.90596355	1.84925849
H	-3.07225957	-2.72331555	1.22980774
H	-2.53093882	-2.62940859	2.92092757
C	0.52732402	-2.26669564	2.48686797
H	0.69791953	-3.34213782	2.36273619
H	0.26476671	-2.04930139	3.52653505

H	1.42543468	-1.70543858	2.22471869
Si	0.48813836	1.56942761	0.80447106
Cl	-0.62760923	3.34738395	1.81085199
Cl	1.84338743	-0.09978971	-0.08765138

AF-TS1

E: -5471.59

H: -5222.08

G: -5286.07

N_{imag} = 1 (-100.9625 cm^{-1})

Pt	-1.87169416	0.40182147	1.22161204
Pt	-0.98879192	0.99507706	-1.43799244
Cl	0.20359161	1.28576442	3.20527432
Cl	1.78112826	2.30920129	-0.96378024
P	-3.23053980	2.26492244	1.29877478
P	-2.19701783	2.95415288	-1.57367547
P	-0.20663073	-1.13813620	-1.82933639
P	-1.25458175	-1.81408217	1.04089913
C	-4.99030693	1.74047531	1.09661328
H	-5.66635364	2.60143599	1.15883494
H	-5.24240550	1.02331803	1.88373649
H	-5.10797630	1.24791809	0.12698934
C	-3.26346559	3.26113167	2.84227105
H	-3.95581315	4.10635389	2.75652710
H	-2.24960315	3.61589426	3.04221187
H	-3.57416106	2.60981096	3.66517554
C	-3.04798543	3.55315211	-0.03188667
H	-4.03031288	3.96743181	-0.28474682
H	-2.43021503	4.35062828	0.38293967
C	-1.35774996	4.46128405	-2.20474441
H	-2.04338344	5.31586602	-2.22921159
H	-0.98730046	4.25373833	-3.21337420
H	-0.50594853	4.67632218	-1.55489378
C	-3.60735616	2.72583722	-2.74394960
H	-4.19222630	3.64854649	-2.83464918
H	-4.24947294	1.91820222	-2.38034408
H	-3.21567389	2.44511960	-3.72625411
C	1.46608403	-1.33990447	-2.56092296
H	1.71092966	-2.39846242	-2.70414906
H	2.19108568	-0.86957695	-1.89215443

H	1.48703885	-0.82054508	-3.52396245
C	-1.31173921	-1.98521918	-3.04258420
H	-0.95457358	-3.00002929	-3.25302235
H	-1.33373811	-1.40716351	-3.97129976
H	-2.32604295	-2.03005371	-2.63513561
C	-0.19402476	-2.32778906	-0.39956093
H	0.83269140	-2.37566971	-0.03453403
H	-0.49043606	-3.32567104	-0.74144253
C	-2.76731203	-2.84688733	0.80085206
H	-2.51094064	-3.91127255	0.74170267
H	-3.27010232	-2.53641150	-0.11971301
H	-3.44887680	-2.68377113	1.64124171
C	-0.42429741	-2.60999226	2.47379615
H	-0.19667147	-3.66223989	2.26872582
H	-1.08872069	-2.53683303	3.34048585
H	0.49211991	-2.05542247	2.68935876
Si	0.27496709	1.47957624	0.63102800
Cl	-0.03333837	3.58458175	1.13454631
Cl	1.75619806	-0.10442063	0.90678597

AF-I

E: -5517.36

H: -5266.80

G: -5330.90

$N_{imag} = 0$

Pt	-0.22799846	0.01012918	0.21481165
Pt	-2.88387338	1.08437523	-0.14917539
Cl	1.89995860	-0.84622781	-0.76206354
Cl	-3.71330717	1.40159432	2.85012415
P	-1.12992550	-2.10508229	-0.15451868
P	-3.98813843	-0.92293441	-0.37480117
P	-2.28543264	3.29721487	-0.35731370
P	0.59192347	2.16049773	-0.14211907
C	-0.96353449	-2.46774086	-1.94920468
H	-1.43838645	-3.42368167	-2.19544008
H	0.10040623	-2.49532049	-2.19557096
H	-1.42979044	-1.66137191	-2.52135235
C	-0.32921858	-3.53489843	0.66796722
H	-0.75374824	-4.48005407	0.31237973
H	-0.47040993	-3.44343067	1.74782767

H	0.74008913	-3.49588630	0.44038584
C	-2.93720495	-2.36478245	0.15334078
H	-3.26354034	-3.29969450	-0.31765822
H	-3.05729287	-2.45717797	1.23632724
C	-5.49597667	-1.16266124	0.65228074
H	-5.87364357	-2.18790158	0.56729134
H	-6.26259105	-0.45665848	0.31937960
H	-5.24654949	-0.93828215	1.69303704
C	-4.55266379	-1.40212652	-2.06549015
H	-4.99416651	-2.40556849	-2.06391951
H	-3.70822472	-1.37465611	-2.75857538
H	-5.29756775	-0.67348488	-2.39889947
C	-3.20339516	4.50817544	0.68033189
H	-2.76412189	5.50911958	0.60186681
H	-3.17747081	4.16595877	1.71857240
H	-4.24587204	4.53399031	0.34945775
C	-2.36052530	4.04712188	-2.04221129
H	-1.98354704	5.07650397	-2.03263016
H	-3.40238787	4.04041704	-2.37603065
H	-1.77039001	3.44754105	-2.73975022
C	-0.52744627	3.60188649	0.17132860
H	-0.54905163	3.74824443	1.25474456
H	-0.11260677	4.50244636	-0.29695204
C	0.96550648	2.31502969	-1.93563125
H	1.29016812	3.33327367	-2.17584167
H	0.06989545	2.06392228	-2.50998456
H	1.74885868	1.59571839	-2.18540117
C	2.16025164	2.62804973	0.68471010
H	2.51261815	3.60418890	0.33410702
H	2.90210798	1.85764104	0.45477682
H	1.99357126	2.65550369	1.76448251
Si	-1.81072840	0.64348023	1.95154565
Cl	-1.81423239	-1.15772230	3.16738999
Cl	-0.57234063	1.94333184	3.17513086

AF-TS2

E: -5510.37

H: -5260.18

G: -5323.77

N_{imag} = 1 (-34.3459 cm^{-1})

Pt	1.62541572	0.53892153	-0.02102627
Pt	-1.60400636	0.18703101	0.03756810
Cl	3.87051154	-0.56484024	0.00907231
Cl	-3.33217245	2.40376361	-0.09535380
P	1.52994600	0.10915576	2.27069166
P	-1.61169077	-0.06396512	2.34032180
P	-1.52654690	-0.27978112	-2.23944825
P	1.57933674	0.30240169	-2.33630623
C	1.79289472	-1.69275299	2.51184965
H	1.69455020	-1.95266848	3.57136141
H	2.78792610	-1.95041903	2.14355237
H	1.05135747	-2.23864149	1.92252941
C	2.79940200	0.91492125	3.32026020
H	2.75639051	0.54150092	4.34907575
H	2.63022490	1.99567700	3.31041129
H	3.78005141	0.70223040	2.88492892
C	-0.03797165	0.41086324	3.20585047
H	0.02578644	-0.10263784	4.17279875
H	-0.09864003	1.48553290	3.38990635
C	-2.85609228	0.93720521	3.24703217
H	-2.74830150	0.82012579	4.33096925
H	-3.85344395	0.61624554	2.93241421
H	-2.73289291	1.98347497	2.95601706
C	-1.93718937	-1.77871286	2.93899058
H	-1.93936536	-1.81734390	4.03469084
H	-1.17798710	-2.45856603	2.54554455
H	-2.91510339	-2.09497603	2.56302495
C	-2.92673589	0.39786260	-3.21763074
H	-2.79210685	0.22091965	-4.29041359
H	-3.00110952	1.46820062	-3.00533039
H	-3.85093891	-0.07771396	-2.87541611
C	-1.52574501	-2.07165357	-2.67669013
H	-1.47941104	-2.21541521	-3.76267137
H	-2.44560042	-2.52079450	-2.28931589
H	-0.67550456	-2.56387670	-2.19871286
C	-0.07184903	0.40012930	-3.17122249
H	-0.28707130	1.45953149	-3.32753875
H	-0.00161430	-0.08624749	-4.15168097
C	2.16444063	-1.37322003	-2.80932262
H	2.06664860	-1.51148797	-3.89140897
H	1.57245711	-2.12645893	-2.28374318

H	3.20599592	-1.47907586	-2.50008118
C	2.62588844	1.43869110	-3.32449490
H	2.58179853	1.18817022	-4.38994077
H	3.65508500	1.34806020	-2.96400318
H	2.28008887	2.46372138	-3.16717274
Si	-0.18233144	1.97951141	-0.00321606
Cl	-0.22728133	3.34786052	1.61698516
Cl	-0.23749320	3.35954009	-1.61370577

References

1. C. Brunecker, M. Arrowsmith, J. H. Müssig, J. Böhnke, A. Stoy, M. Heß, A. Hofmann, C. Lenczyk, C. Lichtenberg, J. Ramler, A. Rempel and H. Braunschweig, *Dalton Trans.,* 2021, **50**, 3506–3515.
2. L. Manojlović-Muir, K. W. Muir and T. Solomun, *Acta Cryst. B*, 1979, **35**, 1237–1239.
3. C. Brunecker, J. H. Müssig, M. Arrowsmith, F. Fantuzzi, A. Stoy, J. Böhnke, A. Hofmann, R. Bertermann, B. Engels and H. Braunschweig, *Chem. Eur. J.,* 2020, **26**, 8518–8523.
4. a) M. C. Grossel, J. R. Batson, R. P. Moulding and K. R. Seddon, *J. Organomet. Chem.*, 1986, **304**, 391–423; b) M. P. Brown, R. J. Puddephatt, L. Rashidi and K. R. Seddon, *J. Chem. Soc., Dalton Trans.*, 1978, 516–522.
5. G. M. Sheldrick, *Acta Cryst. A*, 2015, **71**, 3–8.
6. G. M. Sheldrick, *Acta Cryst. A*, 2008, **64**, 112–122.
7. A.L. Spek, *Acta Cryst. C*, 2015, **71**, 9–18.
8. a) G. te Velde, F. M. Bickelhaupt, E. J. Baerends, C. Fonseca Guerra, S. J. A. van Gisbergen, J. G. Snijders and T. Ziegler, *J. Comput. Chem.*, 2001, **22**, 931–967; b) C. Fonseca Guerra, J. G. Snijders, G. te Velde and E. J. Baerends, *Theor. Chem. Acc.*, 1998, **99**, 391–403; c) ADF 2023.1, SCM, Theoretical Chemistry, Vrije Universiteit Amsterdam, Amsterdam, The Netherlands, www.scm.com; d) AMS 2023.1, SCM, Theoretical Chemistry, Vrije Universiteit Amsterdam, Amsterdam, The Netherlands, www.scm.com.
9. a) A. D. Becke, *Phys. Rev. A*, 1988, **38**, 3098–3100; b) C. Lee, W. Yang and R. G. Parr, *Phys. Rev. B*, 1988, **37**, 785–789; c) Q. Wu and W. Yang, *J. Chem. Phys.*, 2002, **116**, 515–524.
10. a) S. Grimme, *J. Comput. Chem.*, 2004, **25**, 1463–1473; b) S. Grimme, *J. Comput. Chem.*, 2006, **27**, 1787–1799; c) S. Grimme, J. Antony, S. Ehrlich and H. Krieg, *J. Chem. Phys.*, 2010, **132**, Article 154104; d) S. Grimme, S. Ehrlich and L. Goerigk, *J. Comput. Chem.*, 2011, **32**, 1456; e) A. D. Becke and E. R. Johnson, *J. Chem. Phys.*, 2005, **122**, Article 154101; f) E. R. Johnson and A. D. Becke, *J. Chem. Phys.*, 2006, **124**, Article 024101.
11. a) A. E. van Lenthe, A. Ehlers and E. J. Baerends, *J. Chem. Phys.*, 1999, **110**, 8943–8953; b) E. van Lenthe, E. J. Baerends and J. G. Snijders, *J. Chem. Phys.*, 1994, **101**, 9783–9792.

12. a) A. Diefenbach and F. M. Bickelhaupt, *J. Phys. Chem. A*, 2004, **108**, 8460–8466; b) G. T. de Jong, M. Solà, L. Visscher and F. M. Bickelhaupt, *J. Chem. Phys.*, 2004, **121**, 9982–9991; c) T. Hansen, X. Sun, M. Dalla Tiezza, W-. J. van Zeist, J. N. P. van Stralen, D. P. Geerke, L. P. Wolters, J. Poater, T. A. Hamlin and F. M. Bickelhaupt, *Chem. Eur. J.*, 2022, **28**, e202201093; d) J. N. P. van Stralen and F. M. Bickelhaupt, *Organometallics*, 2006, **25**, 4260–4264; e) N. Arnold, R. Bertermann, F. M. Bickelhaupt, H. Braunschweig, M. Drisch, M. Finze, F. Hupp, J. Poater and J. A. P. Sprenger, *Chem. Eur. J.*, 2017, **23**, 5948–5955.
13. M. B. Brands, J. Nitsch and C. Fonseca Guerra, *Inorg. Chem.*, 2018, **57**, 2603–2613.
14. a) A. Klamt and G. Schüürmann, *J. Chem. Soc. Perkin Trans. 2*, 1993, 799–805; b) A. Klamt, *J. Phys. Chem.*, 1995, **99**, 2224–2235; c) C. C. Pye and T. Ziegler, *Theor. Chem. Acc.*, 1999, **101**, 396–408.
15. E. van Lenthe and J. Baerends, *J. Comput. Chem.*, 2003, **24**, 1142–1156.
16. a) M. Franchini, P. H. T. Philipsen, E. van Lenthe and L. Visscher, *J. Chem. Theory Comput.* 2014, **10**, 1994–2004; b) A. D. Becke, *J. Chem. Phys.*, 1993, **88**, 2547–2553; c) M. Franchini, P. H. T. Philipsen and L. Visscher, *J. Comput. Chem.*, 2013, **34**, 1819–1827.
17. a) S. K. Wolff, *Int. J. Quantum Chem.*, 2005, **104**, 645–659; b) A. Bérces, R. M. Dickson, L. Fan, H. Jacobsen, D. P. Swerhone and T. Ziegler, *Comput. Phys. Commun.*, 1997, **100**, 247–262; c) H. Jacobsen, A. Bérces, D. P. Swerhone and T. Ziegler, *Comput. Phys. Commun.*, 1997, **100**, 263–284.
18. a) K. Fukui, *Acc. Chem. Res.*, 1981, **14**, 363–368; b) L. Deng, T. Ziegler and L. A. Fan, *J. Chem. Phys.*, 1993, **99**, 3823 3828; c) L. Deng and T. Ziegler, *Int. J. Quantum Chem.*, 1994, **52**, 731–738.
19. C. Y. Legault, CYLview20, Université de Sherbrooke, Sherbrooke, Quebec, Canada, 2020, www.cylview.org.
20. a) F. M. Bickelhaupt and E. J. Baerends, in *Reviews in Computational Chemistry*, ed. K. B. Lipkowitz and D. B. Boyd, Wiley-VCH, New York, 2000, **15**, pp. 1–86; b) T. A. Hamlin, P. Vermeeren, C. Fonseca Guerra and F. M. Bickelhaupt, in *Complementary Bonding Analysis*, ed. S. Grabowsky, De Gruyter, Berlin, 2021, **8**, pp. 199–212.
